# Global, regional, and national burden of hepatitis B, 1990–2019: a systematic analysis for the Global Burden of Disease Study 2019

**DOI:** 10.1016/S2468-1253(22)00124-8

**Published:** 2022-06-21

**Authors:** GBD 2019 Hepatitis B Collaborators, Brittney S Sheena, Lindsey Hiebert, Hannah Han, Helen Ippolito, Mohsen Abbasi-Kangevari, Zeinab Abbasi-Kangevari, Hedayat Abbastabar, Amir Abdoli, Hiwa Abubaker Ali, Mesafint Molla Adane, Oyelola A Adegboye, Qorinah Estiningtyas Sakilah Adnani, Shailesh M Advani, Muhammad Sohail Afzal, Saira Afzal, Mohamad Aghaie Meybodi, Bahman Ahadinezhad, Bright Opoku Ahinkorah, Sajjad Ahmad, Tauseef Ahmad, Sepideh Ahmadi, Haroon Ahmed, Muktar Beshir Ahmed, Tarik Ahmed Rashid, Gizachew Taddesse Akalu, Addis Aklilu, Tayyaba Akram, Hanadi Al Hamad, Fares Alahdab, Adugnaw Zeleke Alem, Dejene Tsegaye Alem, Fadwa Alhalaiqa Naji Alhalaiqa, Robert Kaba Alhassan, Liaqat Ali, Muhammad Ashar Ali, Yousef Alimohamadi, Vahid Alipour, Motasem Alkhayyat, Sami Almustanyir, Rajaa M Al-Raddadi, Haya Altawalah, Saeed Amini, Hubert Amu, Robert Ancuceanu, Catalina Liliana Andrei, Tudorel Andrei, Amir Anoushiravani, Adnan Ansar, Anayochukwu Edward Anyasodor, Jalal Arabloo, Morteza Arab-Zozani, Ayele Mamo Argaw, Zeleke Gebru Argaw, Muhammad Arshad, Anton A Artamonov, Tahira Ashraf, Daniel Atlaw, Floriane Ausloos, Marcel Ausloos, Sina Azadnajafabad, Mohammadreza Azangou-Khyavy, Amirhossein Azari Jafari, Ghasem Azarian, Sayna Bagheri, Saeed Bahadory, Atif Amin Baig, Maciej Banach, Nastaran Barati, Amadou Barrow, Abdul-Monim Mohammad Batiha, Diana Fernanda Bejarano Ramirez, Uzma Iqbal Belgaumi, Alemshet Yirga Berhie, Devidas S Bhagat, Nikha Bhardwaj, Pankaj Bhardwaj, Krittika Bhattacharyya, Vijayalakshmi S Bhojaraja, Ali Bijani, Antonio Biondi, Belay Boda Abule Bodicha, Hunduma Amensisa Bojia, Archith Boloor, Cristina Bosetti, Dejana Braithwaite, Nikolay Ivanovich Briko, Zahid A Butt, Luis Alberto Cámera, Raja Chandra Chakinala, Promit Ananyo Chakraborty, Jaykaran Charan, Shu Chen, Jee-Young Jasmine Choi, Sonali Gajanan Choudhari, Fazle Rabbi Chowdhury, Dinh-Toi Chu, Sheng-Chia Chung, Paolo Angelo Cortesi, Benjamin C Cowie, Garland T Culbreth, Omid Dadras, Xiaochen Dai, Lalit Dandona, Rakhi Dandona, Fernando Pio De la Hoz, Sisay Abebe Debela, Mohammed Gebre Dedefo, Feleke Mekonnen Demeke, Takele Gezahegn G Demie, Getu Debalkie Demissie, Meseret Derbew Molla, Abebaw Alemayehu Desta, Deepak Dhamnetiya, Mandira Lamichhane Dhimal, Meghnath Dhimal, Mojtaba Didehdar, Linh Phuong Doan, Fariba Dorostkar, Thomas M Drake, Fatemeh Eghbalian, Michael Ekholuenetale, Iman El Sayed, Maysaa El Sayed Zaki, Muhammed Elhadi, Mohamed A Elmonem, Aisha Elsharkawy, Shymaa Enany, Daniel Berhanie Enyew, Ryenchindorj Erkhembayar, Sharareh Eskandarieh, Firooz Esmaeilzadeh, Sayeh Ezzikouri, Hossein Farrokhpour, Getahun Fetensa, Florian Fischer, Masoud Foroutan, Mohamed M Gad, Abhay Motiramji Gaidhane, Shilpa Gaidhane, Natalie C Galles, Silvano Gallus, Teferi Gebru Gebremeskel, Eyob Alemayehu Gebreyohannes, Keyghobad Ghadiri, Kazem Ghaffari, Mansour Ghafourifard, Seyyed-Hadi Ghamari, Ahmad Ghashghaee, Ali Gholami, Abdolmajid Gholizadeh, Aima Gilani, Amit Goel, Mahaveer Golechha, Pouya Goleij, Davide Golinelli, Giuseppe Gorini, Yitayal Ayalew Goshu, Max G Griswold, Mohammed Ibrahim Mohialdeen Gubari, Bhawna Gupta, Sapna Gupta, Veer Bala Gupta, Vivek Kumar Gupta, Rasool Haddadi, Rabih Halwani, Saeed S Hamid, Samer Hamidi, Asif Hanif, Shafiul Haque, Harapan Harapan, Arief Hargono, Sanam Hariri, Ahmed I Hasaballah, S M Mahmudul Hasan, Soheil Hassanipour, Hadi Hassankhani, Simon I Hay, Khezar Hayat, Golnaz Heidari, Claudiu Herteliu, Demisu Zenbaba Heyi, Kamal Hezam, Ramesh Holla, Mohammad-Salar Hosseini, Mostafa Hosseini, Mehdi Hosseinzadeh, Mihaela Hostiuc, Mowafa Househ, Junjie Huang, Nawfal R Hussein, Ivo Iavicoli, Segun Emmanuel Ibitoye, Olayinka Stephen Ilesanmi, Irena M Ilic, Milena D Ilic, Lalu Muhammad Irham, Jessica Y Islam, Nahlah Elkudssiah Ismail, Kathryn H Jacobsen, Farhad Jadidi-Niaragh, Amirreza Javadi Mamaghani, Shubha Jayaram, Ranil Jayawardena, Rime Jebai, Ravi Prakash Jha, Nitin Joseph, Farahnaz Joukar, Billingsley Kaambwa, Ali Kabir, Zubair Kabir, Rohollah Kalhor, Himal Kandel, Tesfaye K Tesfaye Kanko, Rami S Kantar, Ibraheem M Karaye, Bekalu Getnet Kassa, Phillip M Kemp Bohan, Mohammad Keykhaei, Yousef Saleh Khader, Himanshu Khajuria, Gulfaraz Khan, Imteyaz A Khan, Junaid Khan, Moien AB Khan, Javad Khanali, Amir M Khater, Mahalaqua Nazli Khatib, Mahmoud Khodadost, Abdullah T Khoja, Omid Khosravizadeh, Jagdish Khubchandani, Gyu Ri Kim, Hanna Kim, Min Seo Kim, Yun Jin Kim, Jonathan M Kocarnik, Ali-Asghar Kolahi, Rajasekaran Koteeswaran, G Anil Kumar, Carlo La Vecchia, Dharmesh Kumar Lal, Iván Landires, Savita Lasrado, Jeffrey V Lazarus, Caterina Ledda, Doo Woong Lee, Sang-woong Lee, Yeong Yeh Lee, Miriam Levi, Jiarui Li, Stephen S Lim, Stany W Lobo, Platon D Lopukhov, Joana A Loureiro, Jennifer H MacLachlan, Hassan Magdy Abd El Razek, Muhammed Magdy Abd El Razek, Azeem Majeed, Alaa Makki, Mohammad-Reza Malekpour, Reza Malekzadeh, Ahmad Azam Malik, Fariborz Mansour-Ghanaei, Mohammad Ali Mansournia, Francisco Rogerlândio Martins-Melo, Philippa C Matthews, Walter Mendoza, Ritesh G Menezes, Tuomo J Meretoja, Amanual Getnet Mersha, Tomislav Mestrovic, Ted R Miller, Le Huu Nhat Minh, Andreea Mirica, Seyyedmohammadsadeq Mirmoeeni, Erkin M Mirrakhimov, Sanjeev Misra, Prasanna Mithra, Babak Moazen, Ashraf Mohamadkhani, Mokhtar Mohammadi, Shafiu Mohammed, Nagabhishek Moka, Ali H Mokdad, Jalal Moludi, Sara Momtazmanesh, Lorenzo Monasta, Ghobad Moradi, Maliheh Moradzadeh, Rahmatollah Moradzadeh, Paula Moraga, Ebrahim Mostafavi, Sumaira Mubarik, Malaisamy Muniyandi, Christopher J L Murray, Mohsen Naghavi, Mukhammad David Naimzada, Sreenivas Narasimha Swamy, Zuhair S Natto, Biswa Prakash Nayak, Javad Nazari, Ionut Negoi, Serban Mircea Negru, Seyed Aria Nejadghaderi, Sandhya Neupane Kandel, Huong Lan Thi Nguyen, Che Henry Ngwa, Robina Khan Niazi, Chukwudi A Nnaji, Jean Jacques Noubiap, Ali Nowroozi, Virginia Nuñez-Samudio, Bogdan Oancea, Chimedsuren Ochir, Oluwakemi Ololade Odukoya, In-Hwan Oh, Andrew T Olagunju, Babayemi Oluwaseun Olakunde, Ahmed Omar Bali, Emad Omer, Stanislav S Otstavnov, Bilcha Oumer, Jagadish Rao Padubidri, Adrian Pana, Anamika Pandey, Eun-Cheol Park, Fatemeh Pashazadeh Kan, Urvish K Patel, Uttam Paudel, Ionela-Roxana Petcu, Zahra Zahid Piracha, Richard Charles G Pollok, Maarten J Postma, Akram Pourshams, Hossein Poustchi, Mohammad Rabiee, Navid Rabiee, Alireza Rafiei, Sima Rafiei, Pavan Manibettu Raghuram, Mosiur Rahman, Amir Masoud Rahmani, Setyaningrum Rahmawaty, Aashish Rajesh, Priyanga Ranasinghe, Chythra R Rao, Sowmya J Rao, Mahsa Rashidi, Mohammad-Mahdi Rashidi, David Laith Rawaf, Salman Rawaf, Reza Rawassizadeh, Negar Rezaei, Aziz Rezapour, Sahba Rezazadeh-Khadem, Jefferson Antonio Buendia Rodriguez, Godfrey M Rwegerera, Siamak Sabour, Basema Saddik, Mohammad Reza Saeb, Umar Saeed, Amirhossein Sahebkar, KM Saif-Ur-Rahman, Sarvenaz Salahi, Hamideh Salimzadeh, Chethan Sampath, Abdallah M Samy, Juan Sanabria, Francesco Sanmarchi, Milena M Santric-Milicevic, Arash Sarveazad, Brijesh Sathian, Monika Sawhney, Abdul-Aziz Seidu, Sadaf G Sepanlou, Allen Seylani, Saeed Shahabi, Masood Ali Shaikh, Elaheh Shaker, Murad Ziyaudinovich Shakhmardanov, Mohammed Shannawaz, Suchitra M Shenoy, Jeevan K Shetty, Pavanchand H Shetty, Kenji Shibuya, Jae Il Shin, Parnian Shobeiri, Migbar Mekonnen Sibhat, Achintya Dinesh Singh, Jasvinder A Singh, Surjit Singh, Valentin Yurievich Skryabin, Anna Aleksandrovna Skryabina, Amir Ali Sohrabpour, Suhang Song, Seidamir Pasha Tabaeian, Eyayou Girma Tadesse, Majid Taheri, Mircea Tampa, Ker-Kan Tan, Ahmad Tavakoli, Abdelghani Tbakhi, Belay Negash Tefera, Arash Tehrani-Banihashemi, Habtamu Molla Tesfaw, Rekha Thapar, Aravind Thavamani, Seyed Abolfazl Tohidast, Daniel Nigusse Tollosa, Maria Elena Tosti, Marcos Roberto Tovani-Palone, Eugenio Traini, Mai Thi Ngoc Tran, Indang Trihandini, Biruk Shalmeno Tusa, Irfan Ullah, Marco Vacante, Sahel Valadan Tahbaz, Pascual R Valdez, Shoban Babu Varthya, Bay Vo, Yasir Waheed, Adisu Birhanu Weldesenbet, Melat Woldemariam, Suowen Xu, Seyed Hossein Yahyazadeh Jabbari, Mehdi Yaseri, Yigizie Yeshaw, Vahit Yiğit, Birhanu Wubale Yirdaw, Naohiro Yonemoto, Chuanhua Yu, Ismaeel Yunusa, Mazyar Zahir, Leila Zaki, Mohammad Zamani, Maryam Zamanian, Mikhail Sergeevich Zastrozhin, Theo Vos, John W Ward, M Ashworth Dirac

## Abstract

**Background:**

Combating viral hepatitis is part of the UN Sustainable Development Goals (SDGs), and WHO has put forth hepatitis B elimination targets in its Global Health Sector Strategy on Viral Hepatitis (WHO-GHSS) and Interim Guidance for Country Validation of Viral Hepatitis Elimination (WHO Interim Guidance). We estimated the global, regional, and national prevalence of hepatitis B virus (HBV), as well as mortality and disability-adjusted life-years (DALYs) due to HBV, as part of the Global Burden of Diseases, Injuries, and Risk Factors Study (GBD) 2019. This included estimates for 194 WHO member states, for which we compared our estimates to WHO elimination targets.

**Methods:**

The primary data sources were population-based serosurveys, claims and hospital discharges, cancer registries, vital registration systems, and published case series. We estimated chronic HBV infection and the burden of HBV-related diseases, defined as an aggregate of cirrhosis due to hepatitis B, liver cancer due to hepatitis B, and acute hepatitis B. We used DisMod-MR 2.1, a Bayesian mixed-effects meta-regression tool, to estimate the prevalence of chronic HBV infection, cirrhosis, and aetiological proportions of cirrhosis. We used mortality-to-incidence ratios modelled with spatiotemporal Gaussian process regression to estimate the incidence of liver cancer. We used the Cause of Death Ensemble modelling (CODEm) model, a tool that selects models and covariates on the basis of out-of-sample performance, to estimate mortality due to cirrhosis, liver cancer, and acute hepatitis B.

**Findings:**

In 2019, the estimated global, all-age prevalence of chronic HBV infection was 4·1% (95% uncertainty interval [UI] 3·7 to 4·5), corresponding to 316 million (284 to 351) infected people. There was a 31·3% (29·0 to 33·9) decline in all-age prevalence between 1990 and 2019, with a more marked decline of 76·8% (76·2 to 77·5) in prevalence in children younger than 5 years. HBV-related diseases resulted in 555 000 global deaths (487 000 to 630 000) in 2019. The number of HBV-related deaths increased between 1990 and 2019 (by 5·9% [–5·6 to 19·2]) and between 2015 and 2019 (by 2·9% [–5·9 to 11·3]). By contrast, all-age and age-standardised death rates due to HBV-related diseases decreased during these periods. We compared estimates for 2019 in 194 WHO locations to WHO-GHSS 2020 targets, and found that four countries achieved a 10% reduction in deaths, 15 countries achieved a 30% reduction in new cases, and 147 countries achieved a 1% prevalence in children younger than 5 years. As of 2019, 68 of 194 countries had already achieved the 2030 target proposed in WHO Interim Guidance of an all-age HBV-related death rate of four per 100 000.

**Interpretation:**

The prevalence of chronic HBV infection declined over time, particularly in children younger than 5 years, since the introduction of hepatitis B vaccination. HBV-related death rates also decreased, but HBV-related death counts increased as a result of population growth, ageing, and cohort effects. By 2019, many countries had met the interim seroprevalence target for children younger than 5 years, but few countries had met the WHO-GHSS interim targets for deaths and new cases. Progress according to all indicators must be accelerated to meet 2030 targets, and there are marked disparities in burden and progress across the world. HBV interventions, such as vaccination, testing, and treatment, must be strategically supported and scaled up to achieve elimination.

**Funding:**

Bill & Melinda Gates Foundation.

## Introduction

Hepatitis B is a major global public health concern.^1^, ^2^, ^3^ Hepatitis B virus (HBV) damages the liver through acute and chronic infection, with the majority of the burden coming from long-term consequences of chronic infection, principally cirrhosis and hepatocellular carcinoma.^4^, ^5^ Approximately 80–90% of infants infected in the first year of life and 30–50% of children infected in the first 5 years of life will develop chronic infection, in comparison with only 5% of adults infected later in life.^6^ One in four people with chronic HBV infection are at risk of premature death from cirrhosis or liver cancer.^7^

There have been several initiatives to control and eliminate hepatitis in recent years. In 2015, the UN called on all nations to “combat hepatitis” in target 3.3 of the UN Sustainable Development Goals (SDGs).^8^ In 2016, the World Health Assembly adopted the WHO Global Health Sector Strategy on Viral Hepatitis (WHO-GHSS) goal to eliminate viral hepatitis as a public health threat.^9^ The WHO-GHSS suggested impact targets of a 30% reduction in new hepatitis B cases and a 10% reduction in HBV-related deaths by 2020, and a 95% reduction in new cases and a 65% reduction in deaths by 2030 compared to the baseline year of 2015. The WHO-GHSS also suggested modelled proxies for incidence goals of a HBsAg prevalence in children younger than 5 years of less than 1·0% by 2020 and less than 0·1% by 2030. In their later Interim Guidance for Country Validation of Viral Hepatitis Elimination (WHO Interim Guidance), WHO put forth an absolute mortality rate target of less than or equal to four deaths per 100 000 people per year.^10^

**Research in context**

**Evidence before this study**

Comprehensive and timely estimation of hepatitis B prevalence and mortality is crucial to assess disease patterns, develop policies and programmes, and evaluate progress towards elimination of hepatitis. Several research groups have produced estimates on various measures of hepatitis B. In 2018, the Center for Disease Analysis Foundation produced estimates on prevalence and mortality for 120 locations by use of a compartmental model. In 2015, Schweitzer and colleagues generated the first large-scale systematic review of HBsAg studies and modelled broad time-period and all-age HBsAg estimates for 161 countries. In 2017, WHO reported prevalence estimates produced in collaboration with the London School of Hygiene and Tropical Medicine, and mortality estimates based on data from GLOBOCAN and a meta-analysis by the International Agency for Research on Cancer for WHO member states.

**Added value of this study**

The Global Burden of Diseases, Injuries, and Risk Factors Study (GBD) 2019 provides comparable, detailed, and internally consistent estimates of hepatitis B virus (HBV) prevalence and HBV-related deaths and disability-adjusted life-years (DALYs) for 204 locations and territories, 21 age groups, and by Socio-demographic Index, for 29 years, within a framework that allows direct comparison with 368 other diseases and injuries. Our statistical modelling approach allowed us to generate estimates for all quantities of interest even when there are no or sparse data by incorporating a range of predictive covariates and spatiotemporal techniques. Although estimates in areas without data have high uncertainty, comprehensive estimation of all measures of interest can guide stakeholders on research priorities, inform health agendas, and monitor health progress. GBD 2019 used methods for redistributing vaguely characterised codes in vital registration data (so-called “garbage codes”) to liver cancer, cirrhosis, and acute hepatitis. In particular, we redistributed the International Classification of Diseases, 10th revision (ICD-10) code C22.9, assigning only a proportion of these deaths to primary liver cancer. Furthermore, our compartmental framework synthesises data for multiple non-fatal and fatal measures and we account for heterogeneous data sources. We used detailed estimates for location and time to evaluate progress towards the WHO Global Health Sector Strategy on Viral Hepatitis (WHO-GHSS) 2020 elimination targets and the proposed targets from the WHO Interim Guidance for Country Validation of Viral Hepatitis Elimination. We also evaluated what progress is required over the next decade to achieve the WHO-GHSS 2030 elimination targets.

**Implications of all the available evidence**

Chronic HBV prevalence has declined globally since the introduction of hepatitis B vaccination, and HBV-related death rates have also fallen in the past three decades. HBV-related death counts, however, are increasing in many countries due to population growth and ageing. We conclude that HBV infection continues to be a major cause of premature mortality, despite impressive progress in prevention of chronic infection and reduction in death rates. This suggests that hepatitis B vaccination and other strategies to prevent chronic HBV infection have been effective and must be sustained globally, with more targeted action where there are disparities in access to preventive services. To meet the WHO-GHSS mortality targets for 2030, however, efforts to diagnose and treat existing prevalent cases must also be stepped up, particularly in locations where population growth, ageing, and historically high rates of HBV infection present extra barriers to reducing mortality.

Tools and technologies, such as vaccines, testing, and antiviral therapies, already exist to prevent the transmission of HBV and HBV-related disease progression.^11^, ^12^ In 1992, WHO recommended countries include hepatitis B three-dose primary series (HepB3) in national immunisation schedules. Coverage of HepB3 vaccination increased through the 1990s and 2000s with the declining cost of the vaccine, and support from Gavi, the Vaccine Alliance increased the feasibility of routine vaccination of infants in resource-constrained countries.^13^, ^14^ Mother-to-child transmission, which is responsible for many infections in children, can be reduced through newborn vaccination and administration of hepatitis B immunoglobulin within the first 24 h after birth and administration of antivirals to pregnant women when appropriate.^2^, ^15^ In 2009, WHO recommended all countries introduce universal hepatitis B birth dose vaccination into their schedules,^2^ but more than 50 countries to date have not yet introduced a birth dose policy.^16^ WHO recommends the use of oral antivirals, such as tenofovir, to suppress HBV infection and slow disease progression. Improvements in blood safety,^17^ injection safety,^18^, ^19^ and infection control^20^, ^21^ have aided in hepatitis elimination efforts and strengthened health systems broadly. One simulation study found that new cases of hepatitis B have already been avoided since vaccination efforts have increased, but mortality due to liver disease is expected to rise under the current pace of testing and treatment interventions.^22^

Research groups including the Center for Disease Analysis Foundation,^23^ London School of Hygiene and Tropical Medicine,^11^ WHO,^11^ and Schweitzer and colleagues^24^ have published estimates of various HBV-related measures with different levels of geographical, temporal, and age granularity. The Global Burden of Diseases, Injuries, and Risk Factors Study (GBD), however, produces the most granular, comprehensive, and comparable estimates of the burden of hepatitis B, producing estimates for 21 age groups, in 204 countries and territories (including 194 WHO member states), for every year from 1990 to 2019.^25^ Thus, GBD offers a unique tool to evaluate current epidemiology, trends over time, inequality in burden, and progress towards elimination goals across locations. Here, we provide a detailed account of estimates for the hepatitis B disease burden from GBD 2019, and the methods used to produce them, to improve our understanding of disease burden over time and across locations for priority-setting and health service planning and delivery, and to increase the accessibility of findings for hepatology stakeholders. Our approach of generating estimates for all locations and demographics catalyses strategic global, regional, and national planning and policy action. This GBD analysis improves upon the estimates from previous rounds of GBD with additional data sources and enhanced methods for adjusting non-standard data sources, redistributing deaths attributed to vaguely characterised liver pathologies, estimating mortality of acute infections, and incorporating the impact of infant vaccination programmes on the prevalence of chronic infection.

This manuscript was produced as part of the GBD Collaborator Network and in accordance with the GBD Protocol.

## Methods

### Overview

GBD provides a systematic, comparable method of quantifying morbidity and mortality for 369 diseases by age, sex, year, and geographical location.^25^ As part of GBD 2019, we estimated the non-fatal and fatal health loss due to hepatitis B resulting from three diseases: cirrhosis due to hepatitis B, liver cancer due to hepatitis B, and acute hepatitis B. This Article refers to the aggregate of these diseases as HBV-related diseases. We did not account for health loss due to extra-hepatic manifestations of HBV infection. All estimates computed in GBD were carried out 1000 times at the draw level to account for uncertainty from input data, data adjustments, and model selection. The bounds of the 95% uncertainty intervals (UIs) were taken as the 25th and 975th of the 1000 ordered draws.^25^ Detailed descriptions of the overall GBD methodology have been published previously,^25^ and the complete protocol can be accessed online. Websites cited in this Article were last accessed on Jan 9, 2022, unless otherwise stated. Summaries of previously published methods and additional details specific to HBV-related disease estimation can be found in the appendix (sections 1–13). All demographic inputs to the analyses below were estimated in GBD and have been separately reported.^26^

### HBsAg seroprevalence in chronic HBV infection

The input data to the GBD chronic hepatitis B estimation model were HBsAg seroprevalence data from population-based surveys. In GBD 2019, we expanded the number of studies included in our model from the systematic review published by Schweitzer and colleagues,^24^ building on the systematic review that was done as part of GBD 2013.^1^ The details of the GBD 2013 systematic review and inclusion and exclusion criteria for studies from all sources are described in the appendix (section 3.2).

In GBD 2019, unlike previous rounds, we generated a data subset of only unvaccinated population samples to generate an initial counterfactual model of HBsAg seroprevalence in the absence of vaccination. In brief, we excluded seroprevalence measurements made on samples in which participants were born after the location-specific year of vaccine introduction (appendix section 3.3). We then processed this subset of seroprevalence data using a meta-regression tool developed for GBD, meta-regression Bayesian, regularised, trimmed (MR-BRT),^25^, ^27^ to split non-sex-specific datapoints into sex-specific points. Bias adjustment factors were estimated and applied to data collected from non-reference study populations (pregnant women and blood donors) based on an analysis of pairs of data (matched by age, sex, location, and year) from general and alternative populations, also done in MR-BRT (appendix section 3.5). We used an age pattern estimated in DisMod to split datapoints reported for broad age ranges.

We estimated the age-sex-year-location-specific counterfactual HBsAg seroprevalence using DisMod-MR 2.1,^25^, ^28^ a Bayesian mixed-effects meta-regression tool. DisMod-MR uses a steady-state compartmental model to generate internally consistent estimates of prevalence, incidence, cause-specific mortality, and remission, estimated at 5-year intervals and subsequently interpolated to produce annual estimates. DisMod uses a geographical cascade in which the estimated fit at one level of the location hierarchy is used as the Bayesian prior to fit a model at the next level in conjunction with data specific to that geographical level.^25^, ^28^ Additional details are provided in the appendix (section 3.6).

After generating counterfactual estimates of what HBsAg seroprevalence would be in the absence of vaccination, these estimates were multiplied by HepB3 vaccine coverage estimates^29^ and 95% vaccine efficacy^30^, ^31^, ^32^, ^33^ to estimate cases averted by vaccination. These were subtracted from the counterfactual seroprevalence estimates from DisMod to estimate true seroprevalence in which the effects of vaccination have been accounted for (appendix section 3.7).

### Cirrhosis

We separately estimated the incidence, prevalence, and mortality of cirrhosis, regardless of aetiology, and the proportions of cases of cirrhosis due to five aetiologies: hepatitis B, hepatitis C, alcohol, non-alcoholic fatty liver disease (NAFLD), and other causes (eg, cryptogenic cirrhosis and haemochromatosis). Details of the data sources and modelling approach for aetiological proportions are provided in the appendix (sections 4.2, 4.6, and 4.10). Estimated proportions were applied to cirrhosis estimates at the draw level to estimate aetiology-specific prevalence, incidence, and mortality.

We estimated the incidence and prevalence of total cirrhosis (decompensated and compensated combined) in a compartmental DisMod-MR 2.1 model^25^, ^28^ with inputs for prevalence, cause-specific mortality rate, excess mortality rate, and zero remission (appendix section 4.8). Prevalence inputs came from hospital and claims data, processed with previously described methodologies (appendix section 4.7).^25^ Cause-specific mortality rate inputs were generated with the Cause of Death Ensemble model (CODEm), as described below.^25^ Excess mortality rate (EMR) inputs were modelled by age, sex, and the Healthcare Access and Quality (HAQ) Index by use of MR-BRT (appendix section 4.5).^25^, ^27^

We estimated the incidence and prevalence of decompensated cirrhosis in another compartmental DisMod-MR 2.1 model (appendix section 4.9).^25^, ^28^ The prevalence and incidence of compensated cirrhosis were estimated by subtracting the prevalence and incidence of decompensated cirrhosis from the respective estimates of total cirrhosis at the draw level (appendix section 4.11).

Vital registration and verbal autopsy data from the cause of death (CoD) database were used to estimate mortality from cirrhosis. Processing of the CoD data has been described in detail elsewhere.^25^, ^34^ In brief, mortality data were mapped from their own cause of death classification systems (most often the International Classification of Diseases 9th revision [ICD-9] or ICD-10) to GBD causes of death (mapping shown in appendix section 5.2). They were processed to account for differences in age and sex reporting, coding discrepancies, and misclassifications. Deaths assigned to invalid causes of death (so-called “garbage codes”) were redistributed to GBD-defined causes. The cause-specific mortality for cirrhosis due to any aetiology was estimated with the CODEm tool, an automated tool that chooses an ensemble of models and predictive covariates that best fit with the observed data (appendix section 5.3).^25^, ^35^

We estimated the proportion of cases of cirrhosis attributable to the five aetiologies outlined above using data from published case series (appendix section 4.2). The proportion of cases due to each aetiology was estimated in single-parameter DisMod models (appendix section 4.10). The proportion estimates were used to split estimates of prevalence and incidence for decompensated and compensated cirrhosis, and mortality due to cirrhosis, to derive aetiology-specific estimates (ie, cirrhosis due to hepatitis B).

### Liver cancer

We separately estimated mortality due to primary liver cancer as well as the prevalence and incidence of primary liver cancer using mortality-to-incidence ratios (MIRs), irrespective of aetiology, and the proportions of cases of liver cancer due to the same five aetiologies as for cirrhosis.

Cancer mortality sources included vital registration, verbal autopsy, and cancer registry data (appendix section 6.2). Mortality and incidence cancer registry data were matched by cancer type, location, age, year, and sex to calculate MIRs. MIRs were modelled with spatiotemporal Gaussian process regression (ST-GPR), which has been previously described.^36^ The cancer registry incidence data were multiplied by the estimated MIRs to generate interim mortality estimates, which were then combined with vital registration and verbal autopsy data to estimate mortality due to primary liver cancer using CODEm (appendix section 6.3). Details of processing vital registration and verbal autopsy data have been previously published.^25^, ^34^ In GBD 2019, we redistributed deaths due to “malignant neoplasm of liver, not specified as primary or secondary” (ICD-10 code C22.9) proportionately to both primary liver cancer and other primary cancers that metastasise to the liver.

For non-fatal burden estimation, the MIRs modelled above were multiplied by mortality estimates produced with CODEm to generate incidence estimates of liver cancer. Survival data were used to estimate prevalence from incidence (appendix section 7.2).

Proportions of cases of liver cancer due to hepatitis B, hepatitis C, alcohol, NAFLD, and other causes were estimated via a similar strategy to that used for aetiological proportions of cases of cirrhosis described above (appendix sections 6.2, 6.4, and 7.3–7.5). Estimates of liver cancer prevalence, incidence, and mortality were multiplied by proportion estimates to produce aetiology-specific estimates (ie, liver cancer due to hepatitis B).

### Acute hepatitis B

We converted the incidence of chronic HBV infection into the incidence of acute hepatitis B infection by dividing the incidence of chronic infection by age-specific estimates of the probability of a new infection becoming chronic (appendix section 8.2).^37^ We generated estimates of acute hepatitis B prevalence from acute hepatitis B incidence by multiplying by an assumed 6-week duration.

Mortality due to acute hepatitis was modelled with vital registration and verbal autopsy data (appendix section 9.2). Cause-specific mortality was estimated with CODEm, as described above. Deaths from acute hepatitis were modelled by encompassing all hepatitis virus types (A, B, C, and E) in a parent CODEm model (appendix section 9.3). We then produced separate CODEm models in which data were limited to each specific virus. Virus-specific deaths due to acute hepatitis were rescaled to fit within the parent model (appendix section 9.4).

### Disability-adjusted life-years

We estimated years of life lost (YLLs) by multiplying estimates of deaths due to HBV infection by the reference life expectancy at each age group. Years lived with disability (YLDs) for HBV-related diseases were estimated by multiplying the prevalence of each non-fatal sequela of these diseases by corresponding disability weights.^38^, ^39^, ^40^ A list of non-fatal sequelae and their disability weights is provided in the appendix (sections 3.8, 4.13, 7.6, and 8.3). Disability-adjusted life-years (DALYs) were generated by summing YLDs and YLLs.

### Socio-demographic Index

Socio-demographic Index (SDI) is a summary measure that quantifies where countries fall on the development spectrum. SDI is a composite of lag-distributed income per capita, average educational attainment for individuals aged 15 years and older, and total fertility rate for women younger than 25 years of age. Additional details about SDI methodology have been described elsewhere.^26^

### Additional analyses

We evaluated progress towards WHO-GHSS 2020 interim targets and proxies^9^ and the WHO Interim Guidance mortality targets for 2030.^10^ For each target—a 10% reduction in mortality and 30% reduction in new cases from 2015, less than 1% prevalence of HBsAg in infants and children younger than 5 years, and an all-age mortality rate less than or equal to four deaths per 100 000 people per year—we calculated the probability of attainment in 2019 based on the percentage of draws meeting the target. A high certainty of goal achievement was defined as 95% of draws at or better than the target value (appendix section 11.1).

We assessed the percentage change over time to highlight changes in estimates since 1990, the earliest year for which GBD produces estimates, and since 2015, the baseline year of the WHO-GHSS targets. We examined death counts and death rates, both by aggregating all age groups and by age standardising, to illustrate how perspectives on progress over time can differ according to the metric assessed.

We calculated the annualised rate of change to determine the extent of the change since setting the baseline goals in 2015, and the progress needed to achieve the WHO-GHSS 2030 targets for viral hepatitis, both as originally described^9^ and on the basis of more recent WHO Interim Guidance.^10^ The annualised rate of change was calculated as the difference in the natural log of the values at the start and end of the time period of interest divided by the number of years in the interval. For the two relative targets, we determined what the target values would be in 2030 if there was a 65% reduction in deaths and a 95% reduction in new cases since 2015, as defined by the WHO-GHSS 2030 goals. We then calculated the annualised rate of change between 2015 and 2019, and between 2019 and 2030, to ascertain what the annualised rates of change have been and what they need to be to meet the proposed goals (appendix section 11.2).

As an analysis of GBD 2019, this study is compliant with the Guidelines for Accurate and Transparent Health Estimates Reporting (GATHER) recommendations (appendix section 14).^41^

### Role of the funding source

Funding was obtained from the Bill & Melinda Gates Foundation. The funder of this study had no role in study design, data collection, data analysis, data interpretation, or the writing of the report.

## Results

### HBsAg seroprevalence

**Figure 1:**
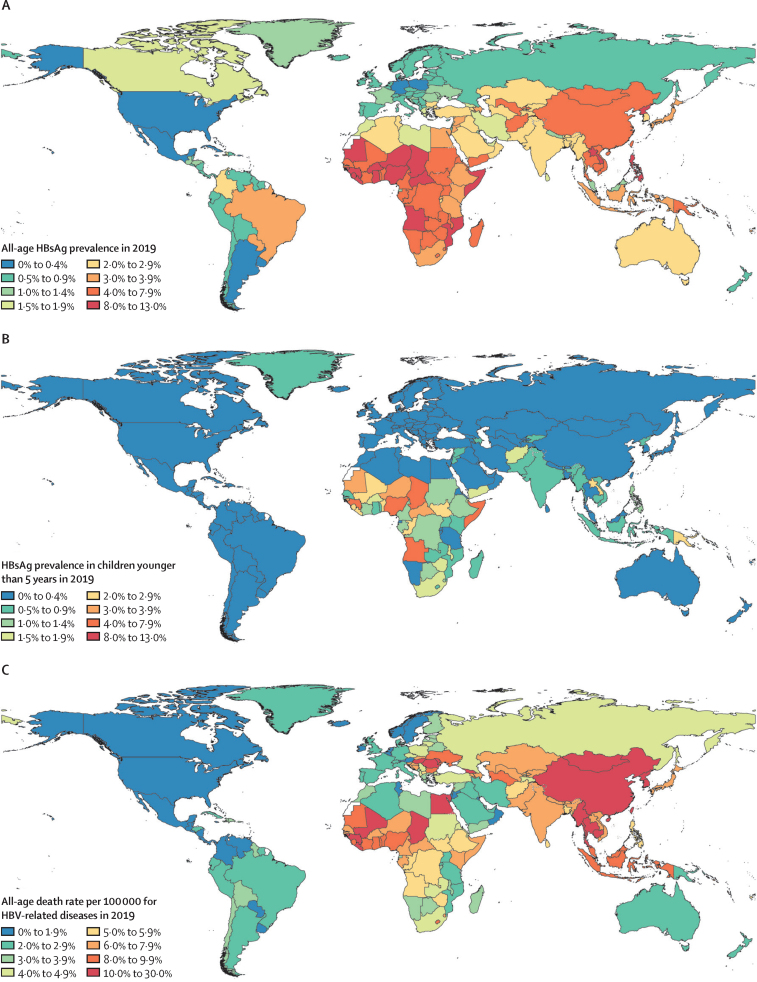
Geographical distribution of the prevalence and death rate of hepatitis B in 2019 (A) All-age HBsAg prevalence in 2019. (B) HBsAg prevalence in children younger than 5 years in 2019. (C) All-age death rate per 100 000 for HBV-related diseases in 2019. HBV=hepatitis B virus.

Globally in 2019, an estimated 316 million (95% UI 284–351) people had chronic HBV infection. The global all-age chronic HBV prevalence was 4·1% (3·7–4·5) in 2019 (table), a 31·3% (29·0–33·9) decrease from 1990 and a 6·8% (5·5–8·3) decline from 2015 (appendix table S2). Counterfactual estimation suggests the global HBsAg prevalence in 2019 would have been 5·2% (4·6–5·8) in the absence of the HepB3 vaccine; this would correspond to a total of 402 million (357–449) cases of HBV infection, suggesting that 85 million (51–118) cases were averted. The chronic HBV all-age prevalence decreased across all WHO regions from 1990 to 2019 and from 2015 to 2019. The highest prevalence in 2019 was in the Western Pacific region (7·1% [6·3–7·9]), followed by the African region (6·5% [5·8–7·3]). The lowest prevalence in 2019 was 1·1% (1·0–1·2), in the European region (table). Country-level variation in all-age chronic HBV prevalence in 2019 is shown in figure 1A.

**Table:** HBsAg prevalence (%) in all ages and children younger than 5 years, all-age death counts, and all-age death rates per 100 000 in 1990, 2015, and 2019, by location

		**HBsAg seroprevalence, all ages (95% UI)**			**HBsAg seroprevalence, children younger than 5 years (95% UI)**			**Death counts, all ages (95% UI)**			**Death rate per 100 000, all ages (95% UI)**		
		1990	2015	2019	1990	2015	2019	1990	2015	2019	1990	2015	2019
**Global**		**6·0% (5·3 to 6·6)**	**4·4% (3·9 to 4·8)**	**4·1% (3·7 to 4·5)**	**4·4% (3·6 to 5·2)**	**1·2% (1·0 to 1·4)**	**1·0% (0·8 to 1·2)**	**524 000 (468 000 to 585 000)**	**540 000 (486 000 to 599 000)**	**555 000 (487 000 to 630 000)**	**9·8 (8·8 to 10·9)**	**7·3 (6·6 to 8·1)**	**7·2 (6·3 to 8·1)**
African region		10·2% (8·9 to 11·4)	7·1% (6·3 to 8·0)	6·5% (5·8 to 7·3)	7·8% (6·4 to 9·3)	3·0% (2·4 to 3·5)	2·7% (2·2 to 3·2)	52 400 (41 600 to 64 800)	69 700 (57 200 to 84 500)	71 000 (57 500 to 87 300)	10·3 (8·2 to 12·7)	7·0 (5·8 to 8·5)	6·5 (5·2 to 7·9)
Eastern Mediterranean region		4·9% (4·4 to 5·5)	3·3% (3·0 to 3·6)	3·1% (2·8 to 3·4)	3·0% (2·4 to 3·6)	1·0% (0·8 to 1·2)	0·8% (0·6 to 0·9)	39 700 (30 000 to 51 800)	46 300 (37 400 to 55 900)	49 700 (39 300 to 62 900)	10·5 (7·9 to 13·7)	6·9 (5·6 to 8·3)	6·8 (5·4 to 8·7)
European region		1·6% (1·4 to 1·8)	1·2% (1·1 to 1·3)	1·1% (1·0 to 1·2)	1·4% (1·1 to 1·7)	0·2% (0·2 to 0·2)	0·1% (0·1 to 0·2)	39 000 (32 900 to 45 800)	40 700 (34 100 to 48 500)	39 700 (33 000 to 47 700)	4·5 (3·8 to 5·3)	4·4 (3·7 to 5·3)	4·3 (3·5 to 5·1)
Region of the Americas		1·7% (1·5 to 2·0)	1·3% (1·1 to 1·4)	1·2% (1·1 to 1·4)	0·8% (0·6 to 1·0)	0·09% (0·07 to 0·10)	0·08% (0·06 to 0·11)	15 100 (13 400 to 17 100)	17 500 (15 300 to 20 100)	18 500 (16 000 to 21 500)	2·1 (1·9 to 2·4)	1·8 (1·6 to 2·1)	1·8 (1·6 to 2·1)
South-East Asia region		4·0% (3·5 to 4·6)	3·2% (2·8 to 3·6)	3·1% (2·7 to 3·4)	2·4% (2·0 to 3·0)	0·6% (0·5 to 0·8)	0·5% (0·4 to 0·6)	110 000 (91 600 to 135 000)	172 000 (153 000 to 194 000)	169 000 (143 000 to 201 000)	8·5 (7·0 to 10·3)	8·9 (7·9 to 10·1)	8·4 (7·1 to 10·0)
Western Pacific region		10·8% (9·7 to 11·9)	7·7% (6·9 to 8·5)	7·1% (6·3 to 7·9)	8·3% (6·9 to 9·7)	1·0% (0·8 to 1·1)	0·5% (0·4 to 0·6)	266 000 (231 000 to 309 000)	192 000 (170 000 to 215 000)	206 000 (173 000 to 240 000)	17·0 (14·8 to 19·7)	10·1 (9·0 to 11·4)	10·7 (9·0 to 12·4)
Low SDI		7·9% (6·9 to 8·9)	6·0% (5·3 to 6·7)	5·5% (4·8 to 6·2)	5·9% (4·8 to 7·1)	2·5% (2·0 to 3·0)	2·3% (1·8 to 2·7)	50 300 (41 200 to 61 600)	69 900 (59 400 to 81 900)	70 900 (59 100 to 84 300)	9·5 (7·8 to 11·7)	6·8 (5·8 to 8·0)	6·3 (5·2 to 7·5)
Low-middle SDI		5·5% (4·8 to 6·2)	4·1% (3·7 to 4·6)	3·9% (3·5 to 4·3)	3·7% (3·0 to 4·4)	1·1% (0·9 to 1·3)	0·9% (0·7 to 1·1)	106 000 (92 200 to 122 000)	143 000 (128 000 to 161 000)	141 000 (121 000 to 163 000)	9·4 (8·2 to 10·8)	8·5 (7·6 to 9·6)	8·0 (6·9 to 9·2)
Middle SDI		8·0% (7·1 to 8·8)	5·3% (4·7 to 5·8)	4·8% (4·3 to 5·4)	5·4% (4·5 to 6·4)	0·9% (0·7 to 1·0)	0·6% (0·5 to 0·7)	214 000 (191 000 to 240 000)	203 000 (183 000 to 226 000)	213 000 (185 000 to 243 000)	12·5 (11·1 to 14·0)	8·8 (7·9 to 9·8)	8·9 (7·7 to 10·1)
High-middle SDI		5·4% (4·8 to 5·9)	4·1% (3·7 to 4·5)	3·8% (3·4 to 4·3)	4·0% (3·3 to 4·7)	0·3% (0·2 to 0·3)	0·3% (0·2 to 0·3)	117 000 (103 000 to 134 000)	84 900 (75 900 to 95 400)	89 400 (76 700 to 102 000)	10·2 (8·9 to 11·6)	6·1 (5·4 to 6·8)	6·2 (5·4 to 7·1)
High SDI		2·0% (1·8 to 2·1)	1·5% (1·4 to 1·7)	1·5% (1·4 to 1·6)	0·6% (0·5 to 0·7)	0·2% (0·2 to 0·2)	0·07% (0·06 to 0·09)	36 300 (32 200 to 41 100)	38 200 (33 400 to 43 400)	40 500 (35 200 to 46 500)	4·4 (3·9 to 5·0)	3·9 (3·4 to 4·4)	4·0 (3·5 to 4·6)
**Central Europe, eastern Europe, and central Asia**		**1·8% (1·5 to 2·0)**	**1·4% (1·3 to 1·6)**	**1·3% (1·2 to 1·5)**	**1·6% (1·2 to 2·0)**	**0·3% (0·2 to 0·3)**	**0·2% (0·1 to 0·2)**	**23 800 (20 000 to 27 800)**	**28 000 (23 300 to 33 300)**	**27 100 (22 200 to 32 900)**	**5·7 (4·8 to 6·7)**	**6·7 (5·6 to 8·0)**	**6·5 (5·3 to 7·9)**
Central Asia		4·8% (4·1 to 5·5)	3·5% (3·1 to 3·9)	3·2% (2·8 to 3·6)	3·8% (2·6 to 4·8)	0·4% (0·3 to 0·5)	0·4% (0·3 to 0·5)	4950 (4050 to 5960)	7580 (5940 to 9470)	7570 (5740 to 9850)	7·2 (5·8 to 8·6)	8·5 (6·7 to 10·7)	8·1 (6·1 to 10·5)
	Armenia	3·0% (2·5 to 3·5)	2·2% (1·8 to 2·5)	2·1% (1·7 to 2·4)	1·9% (1·4 to 2·5)	0·2% (0·1 to 0·2)	0·2% (0·1 to 0·2)	121 (92·6 to 152)	244 (191 to 307)	232 (173 to 306)	3·5 (2·7 to 4·5)	8·0 (6·3 to 10·1)	7·7 (5·7 to 10·1)
	Azerbaijan	3·9% (3·3 to 4·5)	3·3% (2·9 to 3·9)	3·2% (2·8 to 3·6)	2·7% (2·0 to 3·4)	0·6% (0·4 to 0·8)	0·6% (0·4 to 0·7)	469 (366 to 586)	778 (579 to 1020)	698 (497 to 959)	6·4 (5·0 to 8·0)	7·9 (5·9 to 10·3)	6·8 (4·8 to 9·3)
	Georgia	1·9% (1·9 to 2·0)	1·6% (1·6 to 1·6)	1·5% (1·5 to 1·5)	1·4% (1·1 to 1·6)	0·3% (0·2 to 0·3)	0·2% (0·2 to 0·3)	494 (368 to 633)	377 (290 to 484)	367 (268 to 493)	9·0 (6·7 to 11·5)	9·9 (7·6 to 12·8)	10·0 (7·3 to 13·5)
	Kazakhstan	3·8% (3·1 to 4·4)	2·4% (2·0 to 2·8)	2·3% (1·8 to 2·6)	2·9% (2·2 to 3·7)	0·3% (0·2 to 0·3)	0·2% (0·2 to 0·3)	772 (619 to 934)	1370 (1040 to 1810)	1340 (969 to 1810)	4·7 (3·8 to 5·7)	7·9 (5·9 to 10·4)	7·3 (5·3 to 9·8)
	Kyrgyzstan	4·1% (3·3 to 4·8)	2·8% (2·3 to 3·3)	2·5% (2·1 to 2·9)	3·1% (2·3 to 3·9)	0·7% (0·5 to 0·8)	0·6% (0·5 to 0·8)	325 (256 to 399)	403 (301 to 534)	404 (292 to 548)	7·3 (5·7 to 8·9)	6·6 (4·9 to 8·7)	6·2 (4·5 to 8·4)
	Mongolia	6·9% (5·7 to 8·1)	4·6% (4·1 to 5·2)	4·3% (3·9 to 4·7)	3·3% (2·2 to 4·5)	0·3% (0·2 to 0·4)	0·3% (0·2 to 0·4)	515 (403 to 647)	882 (636 to 1200)	987 (689 to 1350)	23·9 (18·7 to 30·0)	28·0 (20·2 to 38·1)	29·1 (20·3 to 39·9)
	Tajikistan	4·1% (3·3 to 4·9)	2·8% (2·3 to 3·3)	2·5% (2·1 to 2·9)	2·9% (2·1 to 3·9)	0·4% (0·3 to 0·4)	0·3% (0·2 to 0·4)	353 (267 to 491)	406 (310 to 527)	445 (313 to 615)	6·6 (5·0 to 9·1)	4·7 (3·6 to 6·0)	4·7 (3·3 to 6·5)
	Turkmenistan	4·4% (3·4 to 5·3)	2·9% (2·4 to 3·4)	2·6% (2·2 to 3·0)	3·6% (2·3 to 4·7)	0·4% (0·3 to 0·6)	0·4% (0·3 to 0·5)	320 (252 to 396)	504 (382 to 662)	502 (356 to 695)	8·6 (6·8 to 10·7)	10·4 (7·9 to 13·6)	9·9 (7·0 to 13·7)
	Uzbekistan	7·2% (5·9 to 8·4)	4·9% (4·3 to 5·5)	4·3% (3·9 to 4·9)	5·6% (3·5 to 7·4)	0·5% (0·3 to 0·7)	0·4% (0·3 to 0·5)	1580 (1280 to 1910)	2610 (2000 to 3330)	2600 (1900 to 3540)	7·6 (6·1 to 9·1)	8·2 (6·3 to 10·5)	7·7 (5·6 to 10·5)
Central Europe		1·3% (1·1 to 1·4)	0·8% (0·8 to 0·9)	0·8% (0·7 to 0·9)	0·7% (0·5 to 0·8)	0·08% (0·06 to 0·10)	0·08% (0·06 to 0·10)	11 500 (9430 to 13 600)	7720 (6140 to 9570)	7670 (5920 to 9850)	9·3 (7·7 to 11·0)	6·7 (5·3 to 8·3)	6·7 (5·2 to 8·6)
	Albania	1·0% (0·8 to 1·2)	0·7% (0·6 to 0·8)	0·6% (0·6 to 0·7)	0·5% (0·4 to 0·7)	0·03% (0·02 to 0·04)	0·03% (0·02 to 0·04)	160 (132 to 192)	115 (79·6 to 160)	123 (83·3 to 173)	4·8 (4·0 to 5·8)	4·1 (2·9 to 5·7)	4·5 (3·1 to 6·4)
	Bosnia and Herzegovina	0·7% (0·6 to 0·8)	0·6% (0·5 to 0·7)	0·6% (0·5 to 0·7)	0·3% (0·2 to 0·4)	0·07% (0·05 to 0·09)	0·08% (0·06 to 0·10)	300 (238 to 367)	223 (176 to 281)	210 (148 to 284)	6·6 (5·2 to 8·1)	6·4 (5·0 to 8·0)	6·4 (4·5 to 8·6)
	Bulgaria	4·0% (3·9 to 4·2)	2·7% (2·6 to 2·7)	2·4% (2·3 to 2·5)	2·4% (2·0 to 2·9)	0·3% (0·3 to 0·4)	0·3% (0·2 to 0·3)	882 (701 to 1080)	631 (482 to 816)	635 (443 to 868)	10·2 (8·1 to 12·4)	8·8 (6·7 to 11·4)	9·2 (6·4 to 12·5)
	Croatia	1·7% (1·5 to 1·8)	1·2% (1·1 to 1·3)	1·1% (1·0 to 1·2)	1·0% (0·8 to 1·2)	0·08% (0·06 to 0·10)	0·09% (0·07 to 0·11)	611 (469 to 766)	276 (209 to 355)	263 (184 to 363)	12·5 (9·6 to 15·6)	6·4 (4·9 to 8·2)	6·2 (4·3 to 8·5)
	Czech Republic	1·0% (0·8 to 1·1)	0·8% (0·7 to 0·9)	0·7% (0·6 to 0·8)	0·5% (0·3 to 0·7)	0·03% (0·02 to 0·04)	0·03% (0·02 to 0·04)	793 (664 to 947)	503 (376 to 652)	506 (367 to 680)	7·7 (6·4 to 9·2)	4·8 (3·6 to 6·2)	4·7 (3·4 to 6·4)
	Hungary	0·7% (0·7 to 0·8)	0·7% (0·6 to 0·8)	0·7% (0·6 to 0·7)	0·3% (0·2 to 0·4)	0·2% (0·2 to 0·3)	0·2% (0·2 to 0·3)	2010 (1590 to 2450)	1150 (880 to 1440)	1110 (830 to 1480)	19·3 (15·3 to 23·5)	11·7 (9·0 to 14·7)	11·5 (8·6 to 15·3)
	Montenegro	0·7% (0·6 to 0·9)	0·7% (0·5 to 0·8)	0·6% (0·5 to 0·7)	0·4% (0·3 to 0·6)	0·09% (0·06 to 0·10)	0·09% (0·06 to 0·12)	19·7 (15 to 24·7)	28·8 (22·9 to 35·8)	21·6 (15·7 to 28·8)	3·1 (2·4 to 3·9)	4·6 (3·7 to 5·7)	3·5 (2·5 to 4·6)
	North Macedonia	1·0% (0·9 to 1·2)	0·9% (0·7 to 1·0)	0·8% (0·7 to 0·9)	0·5% (0·4 to 0·7)	0·08% (0·05 to 0·10)	0·08 (0·05 to 0·10)	103 (82·2 to 126)	133 (105 to 164)	124 (86·7 to 168)	5·1 (4·1 to 6·3)	6·2 (4·9 to 7·6)	5·8 (4·0 to 7·8)
	Poland	0·9% (0·8 to 1·0)	0·5% (0·4 to 0·5)	0·4% (0·4 to 0·5)	0·4% (0·3 to 0·5)	0·02% (0·02 to 0·03)	0·02% (0·02 to 0·03)	2540 (2310 to 2790)	1760 (1520 to 2030)	1750 (1380 to 2200)	6·7 (6·1 to 7·3)	4·6 (3·9 to 5·3)	4·6 (3·6 to 5·7)
	Romania	1·4% (1·2 to 1·6)	1·0% (0·8 to 1·1)	1·0% (0·8 to 1·1)	0·9% (0·7 to 1·2)	0·1% (0·1 to 0·2)	0·1% (0·1 to 0·2)	2650 (2040 to 3280)	1950 (1410 to 2550)	2000 (1400 to 2810)	11·3 (8·7 to 14·0)	9·9 (7·1 to 12·9)	10·4 (7·3 to 14·6)
	Serbia	0·9% (0·8 to 1·1)	0·7% (0·6 to 0·8)	0·7% (0·6 to 0·8)	0·6% (0·4 to 0·8)	0·06% (0·04 to 0·08)	0·06% (0·04 to 0·08)	614 (465 to 780)	441 (346 to 567)	406 (289 to 553)	6·5 (5·0 to 8·3)	5·0 (3·9 to 6·4)	4·6 (3·3 to 6·3)
	Slovakia	1·1% (1·0 to 1·3)	0·9% (0·7 to 1·0)	0·8% (0·7 to 0·9)	0·6% (0·4 to 0·8)	0·04% (0·03 to 0·05)	0·04% (0·03 to 0·05)	551 (434 to 689)	364 (271 to 484)	359 (248 to 507)	10·4 (8·2 to 13·0)	6·7 (5·0 to 8·9)	6·6 (4·6 to 9·3)
	Slovenia	1·1% (1·0 to 1·3)	0·9% (0·8 to 1·0)	0·8% (0·7 to 1·0)	0·5% (0·3 to 0·7)	0·07% (0·05 to 0·09)	0·06% (0·04 to 0·08)	249 (170 to 344)	142 (110 to 182)	147 (103 to 209)	12·6 (8·6 to 17·4)	6·9 (5·3 to 8·8)	7·1 (4·9 to 10·1)
Eastern Europe		1·1% (1·0 to 1·2)	0·8% (0·8 to 0·9)	0·8% (0·7 to 0·8)	0·9% (0·8 to 1·1)	0·2% (0·2 to 0·3)	0·08% (0·07 to 0·10)	7350 (6380 to 8410)	12 700 (10 900 to 14 800)	11 900 (9640 to 14 200)	3·2 (2·8 to 3·7)	6·0 (5·2 to 7·0)	5·7 (4·6 to 6·8)
	Belarus	0·9% (0·7 to 1·0)	0·6% (0·5 to 0·7)	0·6% (0·5 to 0·6)	0·8% (0·6 to 1·0)	0·04% (0·03 to 0·05)	0·04% (0·03 to 0·05)	231 (180 to 293)	374 (282 to 492)	354 (237 to 506)	2·2 (1·7 to 2·8)	3·9 (2·9 to 5·1)	3·7 (2·5 to 5·3)
	Estonia	0·7% (0·6 to 0·9)	0·6% (0·5 to 0·6)	0·5% (0·4 to 0·6)	0·5% (0·3 to 0·7)	0·03% (0·02 to 0·04)	0·03% (0·02 to 0·04)	43·6 (34·1 to 54·9)	50·7 (38·3 to 66·9)	49·3 (34·5 to 69·3)	2·8 (2·2 to 3·5)	3·9 (2·9 to 5·1)	3·8 (2·6 to 5·3)
	Latvia	0·9% (0·7 to 1·0)	0·6% (0·5 to 0·7)	0·5% (0·5 to 0·6)	1·0% (0·8 to 1·2)	0·05% (0·04 to 0·07)	0·05% (0·04 to 0·06)	87·3 (70·6 to 106)	71·4 (54·2 to 93·1)	67 (47·8 to 92·2)	3·3 (2·7 to 4·0)	3·6 (2·7 to 4·7)	3·5 (2·5 to 4·8)
	Lithuania	0·8% (0·6 to 0·9)	0·6% (0·5 to 0·6)	0·5% (0·4 to 0·6)	0·5% (0·4 to 0·7)	0·03% (0·02 to 0·04)	0·03% (0·02 to 0·04)	101 (79·5 to 127)	157 (118 to 206)	132 (94 to 185)	2·8 (2·2 to 3·5)	5·4 (4·1 to 7·1)	4·7 (3·4 to 6·6)
	Moldova	1·4% (1·2 to 1·6)	1·2% (1·0 to 1·4)	1·2% (1·0 to 1·3)	1·0% (0·8 to 1·3)	0·3% (0·3 to 0·4)	0·3% (0·2 to 0·4)	658 (483 to 870)	406 (285 to 552)	374 (261 to 525)	14·8 (10·9 to 19·6)	10·8 (7·6 to 14·6)	10·2 (7·1 to 14·2)
	Russia	0·9% (0·8 to 1·0)	0·7% (0·6 to 0·8)	0·6% (0·5 to 0·7)	0·7% (0·6 to 0·9)	0·03% (0·03 to 0·04)	0·03% (0·03 to 0·04)	4260 (3750 to 4840)	8040 (6940 to 9290)	7100 (5730 to 8650)	2·8 (2·5 to 3·2)	5·5 (4·7 to 6·4)	4·8 (3·9 to 5·9)
	Ukraine	1·7% (1·5 to 1·9)	1·4% (1·3 to 1·6)	1·3% (1·1 to 1·5)	1·5% (1·2 to 1·8)	1·0% (0·8 to 1·2)	0·3% (0·2 to 0·4)	1960 (1700 to 2290)	3600 (3070 to 4230)	3800 (2970 to 4760)	3·7 (3·2 to 4·4)	7·9 (6·8 to 9·3)	8·6 (6·7 to 10·8)
**High income**		**1·4% (1·3 to 1·6)**	**1·1% (1·0 to 1·2)**	**1·1% (1·0 to 1·2)**	**0·4% (0·3 to 0·5)**	**0·2% (0·1 to 0·2)**	**0·06% (0·04 to 0·07)**	**34 400 (30 000 to 39 300)**	**35 600 (31 000 to 40 400)**	**37 100 (31 900 to 42 700)**	**3·8 (3·3 to 4·3)**	**3·3 (2·9 to 3·8)**	**3·4 (2·9 to 3·9)**
Australasia		2·5% (2·3 to 2·8)	2·1% (1·8 to 2·3)	1·9% (1·8 to 2·1)	0·8% (0·6 to 1·0)	0·08% (0·06 to 0·10)	0·07% (0·06 to 0·09)	385 (310 to 472)	611 (497 to 752)	626 (505 to 768)	1·9 (1·5 to 2·3)	2·2 (1·8 to 2·7)	2·2 (1·7 to 2·6)
	Australia	2·8% (2·5 to 3·1)	2·3% (2·0 to 2·5)	2·1% (1·9 to 2·3)	0·9% (0·7 to 1·2)	0·08% (0·06 to 0·10)	0·07% (0·06 to 0·09)	333 (262 to 417)	527 (419 to 662)	537 (427 to 674)	2·0 (1·6 to 2·5)	2·3 (1·8 to 2·8)	2·2 (1·7 to 2·7)
	New Zealand	1·3% (1·1 to 1·5)	0·9% (0·8 to 1·0)	0·9% (0·8 to 1·0)	0·2% (0·2 to 0·3)	0·07% (0·05 to 0·09)	0·07% (0·05 to 0·08)	52·2 (46·8 to 58·4)	83·4 (73·7 to 93·4)	88·2 (76·7 to 99·3)	1·5 (1·4 to 1·7)	1·9 (1·7 to 2·1)	2·0 (1·7 to 2·2)
High-income Asia Pacific		3·8% (3·5 to 4·1)	3·1% (2·8 to 3·3)	3·0% (2·7 to 3·3)	1·0% (0·8 to 1·3)	0·8% (0·6 to 1·0)	0·09% (0·07 to 0·11)	17 600 (16 000 to 19 200)	18 300 (16 100 to 20 300)	19 800 (17 100 to 22 400)	10·1 (9·2 to 11·1)	9·8 (8·6 to 10·8)	10·6 (9·1 to 12·0)
	Brunei	4·7% (3·9 to 5·5)	2·5% (2·2 to 2·8)	2·1% (1·8 to 2·4)	0·5% (0·4 to 0·6)	0·2% (0·2 to 0·3)	0·2% (0·2 to 0·2)	15·5 (12·7 to 18·6)	27·6 (22·9 to 32·9)	31·2 (25·2 to 38·4)	6·0 (4·9 to 7·2)	6·6 (5·5 to 7·9)	7·1 (5·8 to 8·8)
	Japan	3·3% (2·9 to 3·6)	3·1% (2·7 to 3·4)	3·0% (2·7 to 3·4)	1·2% (0·9 to 1·5)	1·1% (0·9 to 1·4)	0·08% (0·06 to 0·10)	6970 (6440 to 7570)	7120 (6100 to 8050)	7650 (6470 to 8750)	5·5 (5·1 to 6·0)	5·5 (4·7 to 6·2)	6·0 (5·1 to 6·8)
	Singapore	3·9% (3·6 to 4·3)	2·6% (2·5 to 2·8)	2·7% (2·5 to 2·9)	0·4% (0·3 to 0·6)	0·1% (0·1 to 0·1)	0·1% (0·1 to 0·1)	240 (217 to 262)	444 (378 to 508)	502 (420 to 588)	7·9 (7·1 to 8·6)	8·0 (6·8 to 9·2)	8·9 (7·4 to 10·4)
	South Korea	5·3% (4·9 to 5·7)	3·2% (3·0 to 3·3)	2·9% (2·8 to 3·1)	0·8% (0·6 to 1·1)	0·1% (0·1 to 0·1)	0·1% (0·1 to 0·1)	10 300 (9060 to 11 700)	10 700 (9340 to 12 000)	11 600 (9860 to 13 500)	23·3 (20·4 to 26·3)	20·6 (18·0 to 23·1)	21·7 (18·5 to 25·2)
High-income North America		0·7% (0·6 to 0·7)	0·5% (0·5 to 0·6)	0·5% (0·4 to 0·5)	0·2% (0·1 to 0·2)	0·06% (0·05 to 0·08)	0·05% (0·04 to 0·07)	2700 (2400 to 3010)	5040 (4480 to 5630)	4930 (4250 to 5700)	1·0 (0·9 to 1·1)	1·4 (1·3 to 1·6)	1·4 (1·2 to 1·6)
	Canada	1·9% (1·8 to 2·1)	1·8% (1·6 to 1·9)	1·7% (1·6 to 1·9)	0·8% (0·6 to 1·1)	0·3% (0·2 to 0·4)	0·3% (0·2 to 0·3)	197 (149 to 259)	450 (340 to 583)	445 (332 to 575)	0·7 (0·5 to 0·9)	1·3 (1·0 to 1·6)	1·2 (0·9 to 1·6)
	Greenland	1·5% (1·2 to 1·8)	1·4% (1·1 to 1·7)	1·4% (1·1 to 1·7)	0·6% (0·4 to 0·9)	0·6% (0·4 to 0·8)	0·6% (0·4 to 0·8)	0·614 (0·452 to 0·837)	1·1 (0·775 to 1·54)	1·12 (0·762 to 1·58)	1·1 (0·8 to 1·5)	2·0 (1·4 to 2·7)	2·0 (1·4 to 2·8)
	USA	0·5% (0·5 to 0·6)	0·4% (0·3 to 0·4)	0·3% (0·3 to 0·4)	0·1% (0·1 to 0·2)	0·03% (0·02 to 0·04)	0·03% (0·02 to 0·04)	2500 (2240 to 2780)	4580 (4110 to 5100)	4490 (3870 to 5180)	1·0 (0·9 to 1·1)	1·4 (1·3 to 1·6)	1·4 (1·2 to 1·6)
Southern Latin America		0·4% (0·4 to 0·5)	0·4% (0·3 to 0·4)	0·4% (0·3 to 0·4)	0·2% (0·1 to 0·2)	0·02% (0·01 to 0·03)	0·02% (0·01 to 0·03)	1800 (1310 to 2370)	1630 (1220 to 2150)	1670 (1250 to 2210)	3·6 (2·7 to 4·8)	2·5 (1·9 to 3·3)	2·5 (1·9 to 3·3)
	Argentina	0·4% (0·3 to 0·5)	0·4% (0·3 to 0·4)	0·3% (0·3 to 0·4)	0·1% (0·1 to 0·2)	0·02% (0·01 to 0·03)	0·02% (0·01 to 0·03)	1020 (744 to 1330)	999 (744 to 1310)	1010 (755 to 1350)	3·1 (2·2 to 4·0)	2·3 (1·7 to 3·0)	2·2 (1·7 to 3·0)
	Chile	0·5% (0·4 to 0·6)	0·5% (0·4 to 0·6)	0·5% (0·4 to 0·6)	0·2% (0·1 to 0·2)	0·02% (0·01 to 0·02)	0·02% (0·01 to 0·03)	682 (498 to 904)	569 (420 to 751)	598 (444 to 797)	5·1 (3·8 to 6·8)	3·3 (2·4 to 4·3)	3·3 (2·4 to 4·4)
	Uruguay	0·4% (0·3 to 0·4)	0·3% (0·2 to 0·3)	0·3% (0·2 to 0·3)	0·1% (0·1 to 0·2)	0·02% (0·01 to 0·02)	0·01% (0·01 to 0·02)	94·5 (69 to 125)	62·2 (47·7 to 79·4)	64·2 (48·7 to 82·9)	3·0 (2·2 to 4·0)	1·8 (1·4 to 2·3)	1·9 (1·4 to 2·4)
Western Europe		1·0% (0·9 to 1·1)	0·8% (0·7 to 0·9)	0·8% (0·7 to 0·8)	0·4% (0·3 to 0·5)	0·1% (0·1 to 0·1)	0·06% (0·04 to 0·07)	11 900 (9710 to 14 600)	10 000 (8200 to 12 100)	10 100 (8220 to 12 200)	3·1 (2·5 to 3·8)	2·3 (1·9 to 2·8)	2·3 (1·9 to 2·8)
	Andorra	1·2% (1·0 to 1·4)	1·0% (0·8 to 1·1)	0·9% (0·7 to 1·1)	0·6% (0·4 to 0·8)	0·05% (0·04 to 0·07)	0·04% (0·03 to 0·06)	1·74 (1·17 to 2·51)	2·53 (1·82 to 3·41)	2·79 (1·95 to 3·87)	3·2 (2·2 to 4·6)	3·3 (2·3 to 4·4)	3·4 (2·3 to 4·7)
	Austria	1·0% (0·8 to 1·1)	0·8% (0·6 to 0·9)	0·8% (0·6 to 0·9)	0·4% (0·3 to 0·6)	0·04% (0·03 to 0·06)	0·06% (0·04 to 0·08)	273 (199 to 361)	173 (128 to 228)	167 (124 to 218)	3·5 (2·6 to 4·6)	2·0 (1·5 to 2·6)	1·9 (1·4 to 2·4)
	Belgium	0·7% (0·7 to 0·8)	0·6% (0·5 to 0·6)	0·5% (0·5 to 0·6)	0·3% (0·2 to 0·4)	0·02% (0·01 to 0·02)	0·02% (0·01 to 0·02)	206 (166 to 252)	199 (154 to 255)	188 (143 to 243)	2·1 (1·7 to 2·5)	1·8 (1·4 to 2·3)	1·7 (1·3 to 2·1)
	Cyprus	1·2% (1·1 to 1·2)	0·7% (0·7 to 0·8)	0·7% (0·7 to 0·7)	0·2% (0·2 to 0·3)	0·02% (0·01 to 0·02)	0·02% (0·01 to 0·02)	14·9 (10·4 to 20·9)	14·9 (11·4 to 19·3)	16·1 (12·1 to 21·1)	1·9 (1·3 to 2·7)	1·2 (0·9 to 1·6)	1·2 (0·9 to 1·6)
	Denmark	0·9% (0·7 to 1·1)	0·9% (0·7 to 1·1)	0·9% (0·7 to 1·0)	0·4% (0·3 to 0·5)	0·4% (0·3 to 0·5)	0·4% (0·2 to 0·5)	113 (83·5 to 148)	153 (116 to 199)	156 (118 to 203)	2·2 (1·6 to 2·9)	2·7 (2·0 to 3·5)	2·7 (2·0 to 3·5)
	Finland	1·0% (0·8 to 1·1)	0·9% (0·7 to 1·1)	0·9% (0·7 to 1·0)	0·4% (0·3 to 0·6)	0·4% (0·2 to 0·5)	0·4% (0·3 to 0·5)	103 (78·2 to 132)	197 (146 to 263)	190 (143 to 251)	2·1 (1·6 to 2·6)	3·6 (2·6 to 4·8)	3·4 (2·6 to 4·5)
	France	1·7% (1·7 to 1·8)	1·4% (1·4 to 1·5)	1·4% (1·3 to 1·4)	0·6% (0·5 to 0·8)	0·10% (0·08 to 0·12)	0·07% (0·05 to 0·09)	2030 (1540 to 2630)	1870 (1430 to 2400)	1750 (1310 to 2260)	3·5 (2·7 to 4·5)	2·9 (2·2 to 3·7)	2·6 (2·0 to 3·4)
	Germany	0·4% (0·4 to 0·5)	0·4% (0·3 to 0·4)	0·4% (0·3 to 0·4)	0·1% (0·1 to 0·2)	0·02% (0·01 to 0·03)	0·02% (0·01 to 0·02)	2900 (2130 to 3840)	1960 (1490 to 2590)	1980 (1510 to 2610)	3·6 (2·7 to 4·8)	2·4 (1·8 to 3·1)	2·3 (1·8 to 3·1)
	Greece	2·2% (2·0 to 2·5)	1·8% (1·6 to 2·0)	1·8% (1·6 to 2·0)	0·8% (0·6 to 1·1)	0·05% (0·04 to 0·07)	0·05% (0·04 to 0·07)	324 (253 to 406)	370 (296 to 455)	372 (299 to 463)	3·1 (2·4 to 3·9)	3·5 (2·8 to 4·3)	3·6 (2·9 to 4·5)
	Iceland	0·7% (0·6 to 0·9)	0·7% (0·5 to 0·8)	0·6% (0·5 to 0·8)	0·4% (0·2 to 0·5)	0·3% (0·2 to 0·5)	0·3% (0·2 to 0·4)	2·59 (1·98 to 3·32)	4·1 (3·14 to 5·24)	4·55 (3·5 to 5·92)	1·0 (0·8 to 1·3)	1·2 (1·0 to 1·6)	1·3 (1·0 to 1·7)
	Ireland	0·8% (0·6 to 1·0)	0·7% (0·5 to 0·8)	0·6% (0·5 to 0·7)	0·4% (0·2 to 0·5)	0·04% (0·02 to 0·05)	0·03% (0·02 to 0·04)	28·8 (21·9 to 37·2)	76·6 (59·1 to 97·9)	61·5 (46·7 to 79·8)	0·8 (0·6 to 1·0)	1·6 (1·2 to 2·0)	1·3 (1·0 to 1·6)
	Israel	0·8% (0·7 to 1·0)	0·6% (0·5 to 0·7)	0·5% (0·4 to 0·6)	0·4% (0·3 to 0·5)	0·04% (0·02 to 0·05)	0·04% (0·02 to 0·05)	71·3 (54·5 to 90·9)	84·4 (64·6 to 109)	92·6 (70·9 to 120)	1·4 (1·1 to 1·8)	1·0 (0·7 to 1·3)	1·0 (0·8 to 1·3)
	Italy	1·2% (1·1 to 1·4)	0·8% (0·7 to 0·9)	0·7% (0·6 to 0·8)	0·2% (0·1 to 0·2)	0·03% (0·02 to 0·03)	0·03% (0·02 to 0·03)	2850 (2580 to 3130)	1640 (1460 to 1830)	1710 (1520 to 1930)	5·0 (4·5 to 5·5)	2·7 (2·4 to 3·0)	2·8 (2·5 to 3·2)
	Luxembourg	0·9% (0·8 to 1·1)	0·8% (0·7 to 0·9)	0·8% (0·6 to 0·9)	0·4% (0·2 to 0·5)	0·06% (0·04 to 0·08)	0·09% (0·06 to 0·13)	12·8 (9·41 to 17)	9·53 (7·22 to 12·4)	9·71 (7·25 to 12·8)	3·4 (2·5 to 4·4)	1·7 (1·3 to 2·2)	1·6 (1·2 to 2·1)
	Malta	0·8% (0·7 to 1·0)	0·6% (0·5 to 0·8)	0·6% (0·5 to 0·7)	0·4% (0·3 to 0·6)	0·07% (0·05 to 0·10)	0·06% (0·04 to 0·08)	5·18 (3·86 to 6·82)	5·01 (3·81 to 6·57)	5·31 (3·99 to 6·95)	1·4 (1·0 to 1·8)	1·2 (0·9 to 1·5)	1·2 (0·9 to 1·6)
	Monaco	0·9% (0·7 to 1·1)	0·7% (0·6 to 0·9)	0·7% (0·5 to 0·8)	0·4% (0·2 to 0·5)	0·02% (0·01 to 0·02)	0·02% (0·01 to 0·02)	0·988 (0·698 to 1·35)	1·29 (0·915 to 1·76)	1·29 (0·904 to 1·76)	3·2 (2·3 to 4·4)	3·5 (2·5 to 4·8)	3·4 (2·4 to 4·7)
	Netherlands	0·9% (0·8 to 1·1)	0·9% (0·8 to 1·1)	0·9% (0·7 to 1·0)	0·4% (0·3 to 0·6)	0·08% (0·06 to 0·11)	0·06% (0·04 to 0·07)	193 (148 to 249)	268 (206 to 343)	283 (218 to 362)	1·3 (1·0 to 1·7)	1·6 (1·2 to 2·0)	1·6 (1·3 to 2·1)
	Norway	0·7% (0·6 to 0·8)	0·6% (0·5 to 0·6)	0·5% (0·4 to 0·6)	0·3% (0·3 to 0·4)	0·04% (0·03 to 0·05)	0·03% (0·03 to 0·04)	55 (48 to 62·8)	51·9 (45·2 to 59·1)	51·5 (44·3 to 59·2)	1·3 (1·1 to 1·5)	1·0 (0·9 to 1·1)	1·0 (0·8 to 1·1)
	Portugal	1·3% (1·0 to 1·5)	1·0% (0·8 to 1·2)	0·9 (0·7 to 1·1)	0·6% (0·4 to 0·8)	0·04% (0·03 to 0·05)	0·04% (0·02 to 0·05)	444 (321 to 593)	250 (192 to 321)	258 (199 to 333)	4·4 (3·2 to 5·9)	2·3 (1·8 to 3·0)	2·4 (1·9 to 3·1)
	San Marino	0·9% (0·7 to 1·1)	0·7% (0·6 to 0·8)	0·7% (0·6 to 0·8)	0·4% (0·2 to 0·5)	0·1% (0·1 to 0·1)	0·1% (0·1 to 0·1)	0·656 (0·473 to 0·902)	0·726 (0·453 to 1·09)	0·795 (0·484 to 1·2)	2·8 (2·0 to 3·8)	2·2 (1·4 to 3·4)	2·4 (1·5 to 3·6)
	Spain	1·1% (0·9 to 1·2)	0·8% (0·7 to 0·9)	0·8% (0·7 to 0·8)	0·4% (0·3 to 0·5)	0·03% (0·02 to 0·04)	0·03% (0·02 to 0·04)	1480 (1160 to 1880)	953 (738 to 1210)	954 (732 to 1210)	3·8 (3·0 to 4·8)	2·0 (1·6 to 2·6)	2·1 (1·6 to 2·6)
	Sweden	0·6% (0·5 to 0·7)	0·6% (0·5 to 0·6)	0·5% (0·5 to 0·6)	0·3% (0·2 to 0·4)	0·1% (0·1 to 0·2)	0·08% (0·06 to 0·10)	100 (87 to 116)	127 (108 to 148)	129 (110 to 151)	1·2 (1·0 to 1·3)	1·3 (1·1 to 1·5)	1·3 (1·1 to 1·5)
	Switzerland	0·8% (0·7 to 1·0)	0·8% (0·7 to 0·9)	0·8% (0·7 to 0·9)	0·4% (0·2 to 0·5)	0·3% (0·2 to 0·4)	0·2% (0·1 to 0·2)	130 (97·5 to 167)	207 (158 to 265)	212 (163 to 273)	1·9 (1·4 to 2·4)	2·5 (1·9 to 3·1)	2·4 (1·9 to 3·1)
	UK	0·7% (0·6 to 0·8)	0·7% (0·6 to 0·8)	0·7% (0·6 to 0·8)	0·3% (0·3 to 0·4)	0·3% (0·2 to 0·4)	0·07% (0·05 to 0·09)	589 (511 to 673)	1400 (1220 to 1590)	1450 (1260 to 1660)	1·0 (0·9 to 1·2)	2·1 (1·8 to 2·4)	2·2 (1·9 to 2·5)
**Latin America and Caribbean**		**2·6% (2·3 to 3·0)**	**1·9% (1·6 to 2·1)**	**1·8% (1·5 to 2·0)**	**1·1% (0·9 to 1·4)**	**0·1% (0·1 to 0·1)**	**0·1% (0·1 to 0·1)**	**10 900 (9740 to 12 200)**	**10 900 (9560 to 12 600)**	**12 100 (10 400 to 14 100)**	**2·8 (2·5 to 3·1)**	**1·9 (1·7 to 2·2)**	**2·1 (1·8 to 2·4)**
Andean Latin America		0·6% (0·5 to 0·6)	0·5% (0·5 to 0·6)	0·5% (0·5 to 0·6)	0·2% (0·1 to 0·2)	0·04% (0·03 to 0·05)	0·04% (0·03 to 0·05)	1330 (1090 to 1670)	1620 (1270 to 2060)	1700 (1260 to 2240)	3·5 (2·8 to 4·4)	2·7 (2·1 to 3·5)	2·7 (2·0 to 3·5)
	Bolivia	0·6% (0·5 to 0·7)	0·6% (0·5 to 0·6)	0·6% (0·5 to 0·6)	0·2% (0·2 to 0·3)	0·03% (0·02 to 0·05)	0·03% (0·02 to 0·04)	256 (177 to 362)	355 (253 to 485)	396 (278 to 541)	4·0 (2·8 to 5·6)	3·2 (2·3 to 4·3)	3·3 (2·3 to 4·5)
	Ecuador	0·6% (0·5 to 0·7)	0·5% (0·5 to 0·6)	0·5% (0·4 to 0·5)	0·2% (0·1 to 0·2)	0·04% (0·03 to 0·05)	0·04% (0·03 to 0·06)	262 (208 to 327)	417 (330 to 523)	460 (332 to 629)	2·6 (2·1 to 3·3)	2·6 (2·0 to 3·2)	2·6 (1·9 to 3·6)
	Peru	0·5% (0·5 to 0·6)	0·5% (0·5 to 0·6)	0·5% (0·5 to 0·6)	0·2% (0·1 to 0·2)	0·04% (0·03 to 0·05)	0·04% (0·03 to 0·05)	815 (660 to 1020)	843 (630 to 1120)	846 (581 to 1190)	3·8 (3·0 to 4·7)	2·6 (2·0 to 3·5)	2·5 (1·7 to 3·5)
Caribbean		0·9% (0·7 to 1·0)	0·8% (0·7 to 0·9)	0·8% (0·7 to 0·9)	0·6% (0·4 to 0·7)	0·2% (0·1 to 0·2)	0·2% (0·1 to 0·2)	1230 (983 to 1540)	1130 (857 to 1500)	1270 (937 to 1680)	3·5 (2·8 to 4·4)	2·5 (1·9 to 3·3)	2·7 (2·0 to 3·6)
	Antigua and Barbuda	0·6% (0·5 to 0·8)	0·5% (0·4 to 0·6)	0·5% (0·4 to 0·5)	0·3% (0·2 to 0·4)	0·03% (0·02 to 0·04)	0·03% (0·02 to 0·04)	2·46 (1·99 to 3·02)	1·4 (1·09 to 1·8)	1·55 (1·18 to 2·02)	4·1 (3·3 to 5·0)	1·6 (1·2 to 2·1)	1·8 (1·3 to 2·3)
	The Bahamas	0·6% (0·5 to 0·8)	0·5% (0·4 to 0·6)	0·5% (0·4 to 0·6)	0·3% (0·2 to 0·4)	0·03% (0·02 to 0·04)	0·03% (0·02 to 0·04)	9·26 (7·32 to 11·5)	7·13 (5·42 to 9·32)	8·11 (5·7 to 10·9)	3·6 (2·9 to 4·5)	1·9 (1·5 to 2·5)	2·2 (1·5 to 2·9)
	Barbados	0·5% (0·4 to 0·6)	0·4% (0·4 to 0·5)	0·4% (0·4 to 0·5)	0·3% (0·2 to 0·4)	0·03% (0·02 to 0·05)	0·03% (0·02 to 0·05)	6·61 (5·12 to 8·37)	5·26 (4·02 to 6·83)	6·09 (4·46 to 7·95)	2·6 (2·0 to 3·3)	1·8 (1·4 to 2·3)	2·0 (1·5 to 2·7)
	Belize	0·8% (0·7 to 1·0)	0·5% (0·4 to 0·6)	0·6% (0·5 to 0·6)	0·5% (0·4 to 0·7)	0·08% (0·06 to 0·10)	0·07% (0·05 to 0·09)	4·07 (3·26 to 4·95)	6·26 (4·81 to 8·03)	7·48 (5·65 to 9·81)	2·2 (1·8 to 2·7)	1·7 (1·3 to 2·1)	1·8 (1·4 to 2·4)
	Bermuda	0·6% (0·5 to 0·7)	0·5% (0·4 to 0·6)	0·5% (0·4 to 0·6)	0·3% (0·2 to 0·4)	0·2% (0·2 to 0·3)	0·2% (0·2 to 0·3)	2·28 (1·79 to 2·87)	1·43 (1·09 to 1·85)	1·51 (1·13 to 2·01)	3·8 (3·0 to 4·8)	2·2 (1·7 to 2·8)	2·4 (1·8 to 3·1)
	Cuba	0·5% (0·5 to 0·6)	0·4% (0·3 to 0·4)	0·4% (0·3 to 0·5)	0·04% (0·03 to 0·06)	0·02% (0·02 to 0·03)	0·02% (0·02 to 0·03)	321 (258 to 397)	229 (175 to 292)	242 (174 to 338)	3·0 (2·4 to 3·7)	2·0 (1·5 to 2·6)	2·1 (1·5 to 3·0)
	Dominica	0·7% (0·6 to 0·8)	0·6% (0·5 to 0·7)	0·6% (0·5 to 0·7)	0·4% (0·3 to 0·5)	0·03% (0·02 to 0·05)	0·04% (0·02 to 0·05)	3·18 (2·5 to 4·03)	1·96 (1·46 to 2·57)	1·46 (1·07 to 1·93)	4·3 (3·4 to 5·4)	2·8 (2·1 to 3·7)	2·1 (1·6 to 2·8)
	Dominican Republic	1·0% (0·8 to 1·2)	0·7% (0·6 to 0·8)	0·7% (0·6 to 0·8)	0·6% (0·5 to 0·8)	0·2% (0·2 to 0·3)	0·2% (0·1 to 0·2)	230 (177 to 297)	297 (214 to 404)	361 (242 to 516)	3·2 (2·5 to 4·1)	2·9 (2·1 to 3·9)	3·3 (2·2 to 4·7)
	Grenada	0·8% (0·7 to 0·9)	0·6% (0·5 to 0·7)	0·6% (0·5 to 0·7)	0·5% (0·3 to 0·6)	0·05% (0·04 to 0·07)	0·06% (0·04 to 0·08)	3·88 (3·1 to 4·8)	2 (1·55 to 2·58)	2·14 (1·63 to 2·82)	4·5 (3·6 to 5·6)	1·9 (1·5 to 2·5)	2·1 (1·6 to 2·7)
	Guyana	0·9% (0·8 to 1·1)	0·8% (0·6 to 0·9)	0·7% (0·6 to 0·8)	0·5% (0·3 to 0·6)	0·06% (0·04 to 0·08)	0·05% (0·03 to 0·07)	37·2 (28 to 48·6)	23·7 (16·3 to 32·9)	24·5 (16·6 to 35)	4·8 (3·6 to 6·3)	3·2 (2·2 to 4·5)	3·2 (2·2 to 4·5)
	Haiti	1·9% (1·5 to 2·2)	1·7% (1·3 to 2·0)	1·6% (1·3 to 1·9)	1·2% (0·9 to 1·6)	0·3% (0·2 to 0·4)	0·3% (0·2 to 0·4)	260 (155 to 376)	339 (192 to 505)	374 (212 to 563)	4·1 (2·4 to 5·9)	3·0 (1·7 to 4·4)	3·0 (1·7 to 4·5)
	Jamaica	0·4% (0·3 to 0·5)	0·3% (0·3 to 0·3)	0·3% (0·2 to 0·3)	0·2% (0·2 to 0·3)	0·02% (0·01 to 0·02)	0·01% (0·01 to 0·02)	39·4 (31·6 to 48·5)	32 (25 to 41)	33·7 (24·1 to 45·4)	1·7 (1·3 to 2·0)	1·1 (0·9 to 1·5)	1·2 (0·9 to 1·6)
	Puerto Rico	0·6% (0·5 to 0·7)	0·4% (0·3 to 0·5)	0·4% (0·3 to 0·5)	0·1% (0·1 to 0·2)	0·04% (0·03 to 0·05)	0·04% (0·03 to 0·05)	199 (156 to 251)	104 (80·9 to 134)	108 (73·2 to 149)	5·5 (4·3 to 6·9)	2·8 (2·2 to 3·6)	3·1 (2·1 to 4·2)
	Saint Kitts and Nevis	0·8% (0·7 to 1·0)	0·6% (0·5 to 0·7)	0·6% (0·5 to 0·7)	0·4% (0·3 to 0·5)	0·03% (0·02 to 0·04)	0·03% (0·02 to 0·04)	2·86 (2·27 to 3·53)	1·18 (0·857 to 1·61)	1·59 (1·13 to 2·17)	6·9 (5·5 to 8·5)	2·1 (1·5 to 2·8)	2·7 (1·9 to 3·6)
	Saint Lucia	0·7% (0·6 to 0·8)	0·6% (0·5 to 0·7)	0·6% (0·5 to 0·7)	0·4% (0·3 to 0·5)	0·04% (0·03 to 0·05)	0·05% (0·04 to 0·07)	4·26 (3·42 to 5·33)	2·95 (2·25 to 3·8)	3·39 (2·53 to 4·54)	3·1 (2·5 to 3·9)	1·7 (1·3 to 2·2)	1·9 (1·5 to 2·6)
	Saint Vincent and the Grenadines	0·7% (0·6 to 0·8)	0·6% (0·5 to 0·6)	0·5% (0·4 to 0·6)	0·4% (0·3 to 0·5)	0·02% (0·01 to 0·03)	0·02% (0·01 to 0·03)	3·17 (2·52 to 3·88)	2·21 (1·71 to 2·81)	2·4 (1·82 to 3·13)	2·9 (2·3 to 3·5)	2·0 (1·5 to 2·5)	2·1 (1·6 to 2·8)
	Suriname	0·8% (0·7 to 1·0)	0·7% (0·5 to 0·8)	0·6% (0·5 to 0·7)	0·6% (0·4 to 0·7)	0·04% (0·02 to 0·05)	0·04% (0·03 to 0·05)	15·6 (12·5 to 19·3)	12·5 (9·65 to 16·3)	13·2 (9·55 to 17·6)	4·0 (3·2 to 5·0)	2·2 (1·7 to 2·9)	2·3 (1·7 to 3·1)
	Trinidad and Tobago	0·6% (0·5 to 0·7)	0·5% (0·5 to 0·6)	0·5% (0·4 to 0·6)	0·3% (0·2 to 0·5)	0·06% (0·04 to 0·08)	0·06% (0·04 to 0·08)	39·9 (32·8 to 47·8)	24·7 (17·2 to 34·8)	26·3 (18 to 36·9)	3·3 (2·7 to 4·0)	1·8 (1·2 to 2·5)	1·9 (1·3 to 2·7)
	Virgin Islands	0·6% (0·5 to 0·7)	0·4% (0·4 to 0·5)	0·4% (0·4 to 0·5)	0·2% (0·1 to 0·2)	0·09% (0·06 to 0·13)	0·1% (0·1 to 0·1)	3·61 (2·7 to 4·82)	3·45 (2·51 to 4·62)	3·52 (2·53 to 4·76)	3·4 (2·5 to 4·5)	3·3 (2·4 to 4·4)	3·4 (2·4 to 4·6)
	Central Latin America	1·3% (1·0 to 1·5)	0·9% (0·8 to 1·0)	0·8% (0·7 to 1·0)	0·6% (0·5 to 0·8)	0·1% (0·1 to 0·1)	0·10% (0·07 to 0·10)	2420 (2140 to 2750)	2930 (2510 to 3450)	3540 (2860 to 4300)	1·5 (1·3 to 1·7)	1·2 (1·0 to 1·4)	1·4 (1·1 to 1·7)
	Colombia	3·9% (3·1 to 4·8)	2·8% (2·3 to 3·3)	2·5% (2·1 to 2·9)	2·1% (1·4 to 2·8)	0·3% (0·2 to 0·4)	0·3% (0·2 to 0·3)	231 (184 to 287)	320 (248 to 413)	355 (245 to 500)	0·7 (0·6 to 0·9)	0·7 (0·5 to 0·9)	0·7 (0·5 to 1·0)
	Costa Rica	1·0% (0·8 to 1·2)	0·7% (0·6 to 0·9)	0·7% (0·6 to 0·9)	0·5% (0·4 to 0·7)	0·03% (0·02 to 0·04)	0·02% (0·02 to 0·03)	39·7 (32 to 48·9)	60·1 (46·6 to 76·7)	69 (47·7 to 95·4)	1·3 (1·1 to 1·6)	1·3 (1·0 to 1·7)	1·5 (1·0 to 2·0)
	El Salvador	1·1% (0·9 to 1·4)	0·7% (0·6 to 0·9)	0·7% (0·5 to 0·8)	0·6% (0·5 to 0·9)	0·07% (0·05 to 0·10)	0·07% (0·05 to 0·10)	78·6 (61·4 to 102)	63·8 (47·6 to 84·1)	67 (45·3 to 95·8)	1·5 (1·2 to 1·9)	1·0 (0·8 to 1·4)	1·1 (0·7 to 1·5)
	Guatemala	1·7% (1·3 to 2·0)	1·2% (1·0 to 1·5)	1·2% (1·0 to 1·4)	0·8% (0·6 to 1·1)	0·2% (0·1 to 0·2)	0·2% (0·1 to 0·2)	213 (159 to 278)	292 (217 to 390)	286 (203 to 401)	2·7 (2·0 to 3·5)	1·8 (1·3 to 2·4)	1·6 (1·1 to 2·3)
	Honduras	1·8% (1·4 to 2·1)	1·1% (0·9 to 1·3)	1·1% (0·9 to 1·3)	1·0% (0·7 to 1·3)	0·1% (0·1 to 0·2)	0·1% (0·1 to 0·2)	111 (72·9 to 152)	228 (140 to 337)	249 (151 to 371)	2·4 (1·5 to 3·2)	2·5 (1·6 to 3·7)	2·5 (1·5 to 3·8)
	Mexico	0·3% (0·3 to 0·3)	0·2% (0·2 to 0·2)	0·2% (0·2 to 0·2)	0·1% (0·1 to 0·1)	0·02% (0·02 to 0·03)	0·02% (0·01 to 0·02)	1440 (1310 to 1580)	1630 (1460 to 1820)	2130 (1710 to 2620)	1·7 (1·5 to 1·9)	1·4 (1·2 to 1·5)	1·7 (1·4 to 2·1)
	Nicaragua	1·5% (1·4 to 1·5)	0·8% (0·8 to 0·9)	0·7% (0·7 to 0·8)	0·8% (0·7 to 0·9)	0·03% (0·03 to 0·03)	0·03% (0·03 to 0·03)	40·7 (31·7 to 52·6)	69 (53·1 to 88·3)	75·3 (53·7 to 105)	1·0 (0·8 to 1·4)	1·1 (0·9 to 1·4)	1·2 (0·8 to 1·6)
	Panama	0·8% (0·7 to 1·0)	0·6% (0·5 to 0·7)	0·5% (0·4 to 0·6)	0·5% (0·3 to 0·7)	0·2% (0·1 to 0·2)	0·08% (0·05 to 0·11)	23 (18·4 to 28·5)	31·4 (24·6 to 39·8)	34·3 (24 to 47·7)	1·0 (0·8 to 1·2)	0·8 (0·6 to 1·0)	0·8 (0·6 to 1·1)
	Venezuela	0·9% (0·7 to 1·1)	0·8% (0·6 to 0·9)	0·7% (0·6 to 0·9)	0·5% (0·4 to 0·7)	0·1% (0·1 to 0·2)	0·1% (0·1 to 0·2)	242 (190 to 305)	230 (168 to 314)	283 (190 to 407)	1·3 (1·0 to 1·6)	0·8 (0·6 to 1·1)	1·0 (0·7 to 1·5)
Tropical Latin America		5·0% (4·4 to 5·7)	3·6% (3·1 to 4·0)	3·4% (3·0 to 3·8)	2·2% (1·8 to 2·8)	0·1% (0·1 to 0·2)	0·1% (0·1 to 0·2)	5870 (5320 to 6510)	5260 (4700 to 5890)	5570 (4960 to 6270)	3·8 (3·5 to 4·3)	2·4 (2·2 to 2·7)	2·5 (2·2 to 2·8)
	Brazil	5·1% (4·4 to 5·7)	3·6% (3·1 to 4·0)	3·4% (3·0 to 3·8)	2·2% (1·8 to 2·8)	0·1% (0·1 to 0·2)	0·1% (0·1 to 0·2)	5800 (5250 to 6420)	5160 (4600 to 5760)	5450 (4860 to 6140)	3·9 (3·5 to 4·3)	2·5 (2·2 to 2·8)	2·5 (2·2 to 2·8)
	Paraguay	3·7% (2·9 to 4·5)	3·2% (2·6 to 3·8)	3·0% (2·4 to 3·5)	1·9% (1·3 to 2·6)	0·2% (0·1 to 0·2)	0·1% (0·1 to 0·2)	73·3 (55·2 to 94·4)	106 (77·4 to 145)	118 (79 to 170)	1·8 (1·4 to 2·3)	1·6 (1·2 to 2·2)	1·7 (1·1 to 2·5)
**North Africa and Middle East**		**5·0% (4·5 to 5·4)**	**3·2% (2·9 to 3·4)**	**2·9% (2·7 to 3·2)**	**3·2% (2·6 to 3·8)**	**0·8% (0·6 to 0·9)**	**0·6% (0·4 to 0·7)**	**34 300 (26 800 to 43 800)**	**34 700 (27 300 to 43 300)**	**36 600 (27 100 to 48 200)**	**9·9 (7·8 to 12·7)**	**6·1 (4·8 to 7·6)**	**6·0 (4·4 to 7·9)**
Afghanistan		8·4% (6·8 to 10·2)	6·5% (5·0 to 7·9)	5·9% (4·6 to 7·2)	6·1% (4·4 to 8·0)	2·4% (1·7 to 3·2)	1·7% (1·2 to 2·2)	2090 (1200 to 4380)	2270 (1510 to 3390)	2250 (1480 to 3310)	18·3 (10·5 to 38·4)	6·8 (4·5 to 10·1)	5·9 (3·9 to 8·7)
Algeria		3·7% (3·0 to 4·4)	2·7% (2·2 to 3·1)	2·4% (2·0 to 2·8)	2·7% (2·0 to 3·5)	0·3% (0·2 to 0·4)	0·3% (0·2 to 0·4)	1070 (716 to 1620)	1070 (769 to 1440)	1130 (805 to 1570)	4·2 (2·8 to 6·4)	2·7 (2·0 to 3·7)	2·7 (1·9 to 3·7)
Bahrain		3·7% (3·0 to 4·4)	2·7% (2·3 to 3·2)	2·6% (2·3 to 3·0)	2·1% (1·5 to 2·8)	0·1% (0·1 to 0·2)	0·1% (0·1 to 0·2)	20·2 (15·7 to 25·6)	32·1 (24·3 to 42·7)	41·8 (30·1 to 57·4)	4·0 (3·1 to 5·0)	2·3 (1·7 to 3·0)	2·9 (2·1 to 4·0)
Egypt		5·5% (5·1 to 5·9)	3·9% (3·7 to 4·2)	3·6% (3·4 to 3·8)	1·6% (1·3 to 1·9)	0·1% (0·1 to 0·1)	0·1% (0·1 to 0·1)	17 600 (13 600 to 23 200)	18 000 (12 200 to 25 200)	19 200 (11 700 to 28 400)	31·5 (24·5 to 41·6)	19·5 (13·2 to 27·2)	19·4 (11·8 to 28·7)
Iran		2·5% (2·2 to 2·9)	1·6% (1·4 to 1·8)	1·5% (1·3 to 1·7)	1·6% (1·3 to 2·0)	0·10% (0·08 to 0·10)	0·09% (0·07 to 0·11)	1980 (1670 to 2300)	2260 (2060 to 2490)	2470 (2220 to 2770)	3·4 (2·9 to 3·9)	2·8 (2·5 to 3·1)	2·9 (2·6 to 3·3)
Iraq		3·8% (3·1 to 4·6)	2·2% (1·8 to 2·5)	2·0% (1·6 to 2·3)	2·0% (1·5 to 2·6)	0·8% (0·6 to 1·1)	0·4% (0·3 to 0·6)	611 (464 to 806)	1020 (743 to 1370)	1090 (801 to 1460)	3·5 (2·6 to 4·6)	2·6 (1·9 to 3·5)	2·6 (1·9 to 3·5)
Jordan		9·0% (8·7 to 9·3)	3·8% (3·7 to 3·9)	3·4% (3·3 to 3·5)	5·5% (5·1 to 5·9)	0·6% (0·5 to 0·6)	0·5% (0·5 to 0·6)	99·7 (75·4 to 130)	122 (90·9 to 159)	166 (121 to 217)	2·6 (2·0 to 3·5)	1·3 (0·9 to 1·6)	1·4 (1·0 to 1·9)
Kuwait		2·0% (1·6 to 2·5)	1·1% (0·9 to 1·3)	1·2% (1·0 to 1·4)	0·3% (0·2 to 0·4)	0·07% (0·05 to 0·09)	0·07% (0·05 to 0·09)	27·4 (22 to 33·1)	42·8 (34 to 53·1)	55·9 (41·8 to 74·3)	1·6 (1·3 to 1·9)	1·1 (0·9 to 1·4)	1·3 (0·9 to 1·7)
Lebanon		4·4% (3·7 to 5·1)	3·0% (2·6 to 3·5)	2·8% (2·4 to 3·2)	3·0% (2·2 to 3·9)	0·7% (0·5 to 0·9)	0·6% (0·4 to 0·8)	260 (187 to 337)	301 (212 to 454)	312 (219 to 470)	7·9 (5·7 to 10·3)	6·0 (4·2 to 9·1)	6·0 (4·2 to 9·1)
Libya		2·7% (2·3 to 3·1)	1·6% (1·4 to 1·8)	1·7% (1·5 to 1·9)	1·7% (1·2 to 2·2)	0·1% (0·1 to 0·2)	0·1% (0·1 to 0·2)	202 (126 to 307)	217 (165 to 282)	257 (187 to 352)	4·8 (3·0 to 7·2)	3·3 (2·5 to 4·3)	3·8 (2·8 to 5·2)
Morocco		2·8% (2·6 to 3·0)	1·6% (1·5 to 1·7)	1·5% (1·4 to 1·5)	2·3% (2·0 to 2·6)	0·2% (0·1 to 0·2)	0·1% (0·1 to 0·2)	1280 (791 to 1880)	1220 (828 to 1660)	1300 (913 to 1730)	5·0 (3·1 to 7·4)	3·5 (2·4 to 4·8)	3·6 (2·5 to 4·8)
Oman		5·3% (4·6 to 6·2)	2·5% (2·1 to 3·0)	2·5% (2·1 to 2·9)	0·2% (0·2 to 0·3)	0·09% (0·07 to 0·12)	0·09% (0·07 to 0·11)	61·7 (42·1 to 86·9)	82·9 (61·1 to 109)	83·6 (58 to 117)	3·2 (2·2 to 4·5)	2·0 (1·5 to 2·6)	1·8 (1·3 to 2·5)
Palestine		4·1% (3·3 to 5·0)	1·7% (1·4 to 2·0)	1·5% (1·2 to 1·7)	2·8% (2·0 to 3·8)	0·2% (0·2 to 0·3)	0·2% (0·1 to 0·2)	88·7 (62·3 to 122)	105 (84·6 to 128)	120 (92·3 to 153)	4·3 (3·0 to 5·9)	2·3 (1·8 to 2·8)	2·4 (1·9 to 3·1)
Qatar		2·2% (1·9 to 2·5)	1·7% (1·5 to 1·9)	1·4% (1·3 to 1·6)	0·2% (0·1 to 0·2)	0·10% (0·07 to 0·13)	0·10% (0·07 to 0·10)	17·6 (13·1 to 23·6)	54·8 (38·5 to 75·8)	70·9 (47·5 to 100)	4·0 (2·9 to 5·3)	2·2 (1·6 to 3·1)	2·5 (1·7 to 3·5)
Saudi Arabia		7·5% (6·8 to 8·1)	3·0% (2·6 to 3·4)	2·7% (2·3 to 3·1)	1·7% (1·4 to 2·0)	0·2% (0·1 to 0·2)	0·1% (0·1 to 0·2)	749 (505 to 1100)	671 (511 to 879)	784 (570 to 1060)	4·7 (3·1 to 6·9)	2·1 (1·6 to 2·7)	2·2 (1·6 to 3·0)
Sudan		9·2% (7·8 to 10·7)	5·1% (4·5 to 5·7)	4·6% (4·1 to 5·1)	7·5% (5·6 to 9·5)	1·5% (1·2 to 1·9)	1·2% (0·9 to 1·5)	2520 (1070 to 5620)	1800 (1000 to 3190)	1750 (990 to 3010)	12·5 (5·3 to 27·8)	4·8 (2·7 to 8·5)	4·3 (2·4 to 7·4)
Syria		6·7% (6·2 to 7·2)	3·2% (3·1 to 3·3)	3·1% (3·0 to 3·2)	4·5% (3·6 to 5·4)	1·6% (1·3 to 1·9)	0·8% (0·7 to 1·0)	507 (374 to 665)	514 (359 to 712)	553 (375 to 800)	3·9 (2·9 to 5·2)	3·4 (2·4 to 4·7)	3·8 (2·6 to 5·5)
Tunisia		4·7% (4·3 to 5·1)	2·4% (2·2 to 2·6)	2·4% (2·2 to 2·7)	3·6% (3·0 to 4·3)	0·3% (0·3 to 0·4)	0·3% (0·2 to 0·3)	202 (116 to 323)	201 (132 to 292)	211 (143 to 313)	2·4 (1·4 to 3·8)	1·8 (1·2 to 2·6)	1·8 (1·2 to 2·7)
Turkey		4·5% (4·2 to 4·8)	2·7% (2·5 to 2·8)	2·4% (2·3 to 2·6)	3·5% (2·9 to 4·2)	0·2% (0·2 to 0·3)	0·2% (0·1 to 0·2)	3820 (2970 to 4700)	3580 (2950 to 4340)	3530 (2640 to 4620)	6·4 (5·0 to 7·9)	4·5 (3·7 to 5·5)	4·3 (3·3 to 5·7)
United Arab Emirates		2·1% (1·7 to 2·4)	1·5% (1·3 to 1·7)	1·8% (1·5 to 2·1)	0·1% (0·1 to 0·2)	0·1% (0·1 to 0·2)	0·1% (0·1 to 0·2)	43·4 (28·9 to 62·3)	192 (119 to 296)	264 (161 to 430)	2·3 (1·5 to 3·3)	2·1 (1·3 to 3·3)	2·9 (1·7 to 4·6)
Yemen		10·4% (9·2 to 11·9)	5·7% (5·2 to 6·4)	5·5% (4·9 to 6·0)	8·0% (6·5 to 9·9)	2·2% (1·7 to 2·7)	1·6% (1·3 to 2·0)	1080 (478 to 2340)	869 (546 to 1290)	982 (633 to 1470)	7·8 (3·5 to 17·1)	3·0 (1·9 to 4·5)	3·1 (2·0 to 4·7)
**South Asia**		**3·6% (3·1 to 4·1)**	**2·9% (2·5 to 3·2)**	**2·8% (2·4 to 3·1)**	**2·2% (1·8 to 2·8)**	**0·6% (0·5 to 0·7)**	**0·5% (0·4 to 0·6)**	**83 700 (70 000 to 102 000)**	**142 000 (127 000 to 161 000)**	**137 000 (114 000 to 165 000)**	**7·6 (6·4 to 9·3)**	**8·3 (7·4 to 9·4)**	**7·6 (6·3 to 9·1)**
Bangladesh		3·1% (2·8 to 3·3)	2·3% (2·1 to 2·5)	2·2% (2·0 to 2·4)	1·7% (1·3 to 2·1)	0·2% (0·1 to 0·2)	0·1% (0·1 to 0·2)	11 400 (8160 to 14 700)	8630 (6150 to 11 600)	8690 (6070 to 12 100)	10·5 (7·5 to 13·4)	5·6 (4·0 to 7·6)	5·5 (3·8 to 7·6)
Bhutan		5·8% (5·2 to 6·1)	3·0% (2·8 to 3·1)	2·6% (2·5 to 2·7)	4·4% (3·6 to 5·0)	0·2% (0·1 to 0·2)	0·2% (0·1 to 0·2)	49 (30·7 to 76·9)	49·5 (30·9 to 88·8)	52·6 (32·9 to 94·9)	8·0 (5·0 to 12·6)	6·5 (4·1 to 11·6)	7·0 (4·4 to 12·6)
India		3·7% (3·2 to 4·2)	3·1% (2·7 to 3·4)	2·9% (2·6 to 3·3)	2·3% (1·9 to 2·9)	0·7% (0·5 to 0·8)	0·5% (0·4 to 0·6)	60 300 (49 500 to 76 600)	117 000 (104 000 to 133 000)	110 000 (89 400 to 135 000)	7·0 (5·8 to 9·0)	8·8 (7·8 to 10·0)	7·9 (6·4 to 9·7)
Nepal		1·7% (1·5 to 1·9)	1·2% (1·1 to 1·3)	1·1% (1·0 to 1·2)	1·1% (0·8 to 1·3)	0·2% (0·1 to 0·2)	0·1% (0·1 to 0·2)	2310 (1690 to 3240)	1680 (1180 to 2510)	2060 (1340 to 3040)	11·8 (8·6 to 16·6)	5·8 (4·0 to 8·6)	6·8 (4·4 to 10·0)
Pakistan		3·7% (3·2 to 4·3)	2·6% (2·3 to 2·9)	2·3% (2·0 to 2·6)	2·3% (1·8 to 3·0)	0·7% (0·5 to 0·8)	0·5% (0·4 to 0·7)	9660 (6100 to 13 700)	14 900 (10 800 to 19 600)	16 200 (11 900 to 21 700)	8·6 (5·4 to 12·2)	7·3 (5·2 to 9·5)	7·2 (5·3 to 9·7)
**Southeast Asia, east Asia, and Oceania**		**10·6% (9·4 to 11·7)**	**7·5% (6·7 to 8·2)**	**6·9% (6·2 to 7·7)**	**7·6% (6·3 to 9·0)**	**0·9% (0·8 to 1·1)**	**0·6% (0·5 to 0·7)**	**285 000 (249 000 to 330 000)**	**218 000 (194 000 to 243 000)**	**234 000 (198 000 to 270 000)**	**16·8 (14·7 to 19·4)**	**10·3 (9·2 to 11·5)**	**10·8 (9·2 to 12·5)**
East Asia		11·9% (10·6 to 13·2)	8·5% (7·6 to 9·4)	7·9% (7·0 to 8·8)	9·2% (7·6 to 10·8)	0·5% (0·4 to 0·5)	0·4% (0·3 to 0·5)	236 000 (201 000 to 278 000)	158 000 (139 000 to 181 000)	169 000 (140 000 to 203 000)	19·3 (16·4 to 22·7)	11·0 (9·6 to 12·6)	11·5 (9·5 to 13·8)
	China	11·9% (10·6 to 13·1)	8·4% (7·5 to 9·3)	7·8% (7·0 to 8·7)	9·2% (7·7 to 10·8)	0·5% (0·4 to 0·5)	0·4% (0·3 to 0·5)	229 000 (194 000 to 271 000)	151 000 (132 000 to 174 000)	162 000 (133 000 to 195 000)	19·3 (16·4 to 22·9)	10·9 (9·5 to 12·5)	11·4 (9·3 to 13·7)
	North Korea	14·9% (11·9 to 17·9)	11·4% (9·3 to 13·5)	10·7% (9·1 to 12·3)	11·2% (8·3 to 14·4)	0·7% (0·6 to 1·0)	0·6% (0·5 to 0·8)	3390 (2530 to 4480)	3680 (2740 to 4770)	3730 (2720 to 4880)	16·1 (12·0 to 21·3)	14·2 (10·6 to 18·4)	14·2 (10·4 to 18·6)
	Taiwan (province of China)	10·2% (9·7 to 10·7)	8·2% (7·9 to 8·6)	7·9% (7·5 to 8·2)	0·8% (0·6 to 0·9)	0·2% (0·1 to 0·2)	0·2% (0·1 to 0·2)	3730 (3330 to 4140)	3190 (2680 to 3720)	3680 (2710 to 4950)	18·3 (16·3 to 20·3)	13·6 (11·4 to 15·8)	15·6 (11·5 to 20·9)
Oceania		9·3% (7·8 to 10·3)	6·3% (5·5 to 6·8)	5·9% (5·5 to 6·2)	4·9% (3·2 to 6·2)	2·1% (1·2 to 2·7)	2·3% (1·7 to 2·9)	363 (285 to 463)	516 (393 to 667)	559 (422 to 727)	5·6 (4·4 to 7·2)	4·3 (3·3 to 5·5)	4·2 (3·2 to 5·5)
	American Samoa	5·7% (4·5 to 6·9)	5·6% (4·6 to 6·6)	5·4% (4·5 to 6·3)	2·8% (1·9 to 3·9)	2·6% (1·8 to 3·7)	2·6% (1·8 to 3·4)	2·65 (2·15 to 3·2)	4·45 (3·62 to 5·42)	4·71 (3·78 to 5·93)	5·5 (4·4 to 6·6)	7·9 (6·5 to 9·7)	8·5 (6·8 to 10·7)
	Cook Islands	5·0% (4·0 to 6·1)	3·8% (3·1 to 4·3)	3·3% (2·7 to 3·7)	1·1% (0·7 to 1·5)	0·3% (0·2 to 0·4)	0·2% (0·2 to 0·3)	1·68 (1·33 to 2·04)	1·71 (1·37 to 2·14)	1·84 (1·39 to 2·38)	8·8 (7·0 to 10·7)	9·5 (7·6 to 11·9)	10·2 (7·7 to 13·2)
	Federated States of Micronesia	5·7% (4·6 to 6·6)	3·9% (3·3 to 4·4)	3·6% (3·1 to 4·0)	1·2% (0·8 to 1·6)	0·9% (0·6 to 1·1)	0·8% (0·6 to 1·1)	11·6 (7·95 to 16·2)	10·1 (6·16 to 15·8)	10·8 (6·16 to 17·3)	11·1 (7·6 to 15·5)	9·8 (6·0 to 15·3)	10·6 (6·0 to 16·9)
	Fiji	7·1% (5·8 to 8·2)	4·4% (3·8 to 5·0)	3·6% (3·1 to 4·1)	2·0% (1·2 to 2·6)	0·6% (0·4 to 0·8)	0·5% (0·3 to 0·6)	37·2 (29 to 47)	48·9 (38 to 61·6)	50·7 (37·2 to 67·6)	4·9 (3·8 to 6·2)	5·4 (4·2 to 6·9)	5·6 (4·1 to 7·4)
	Guam	5·5% (4·5 to 6·7)	5·5% (4·6 to 6·4)	5·0% (4·3 to 5·9)	2·4% (1·6 to 3·3)	2·3% (1·5 to 3·0)	2·1% (1·4 to 2·8)	9·74 (8·05 to 11·7)	19·2 (16·1 to 23)	20·3 (16·4 to 25·2)	7·1 (5·9 to 8·6)	11·4 (9·6 to 13·7)	11·9 (9·6 to 14·8)
	Kiribati	10·4% (7·4 to 12·8)	8·5% (6·4 to 10·3)	7·5% (6·1 to 8·9)	6·4% (3·3 to 8·8)	3·1% (1·6 to 4·2)	2·8% (1·8 to 3·6)	16·2 (11·9 to 21·1)	15·6 (9·78 to 23·4)	16 (9·93 to 23·9)	21·8 (16·1 to 28·4)	13·9 (8·7 to 20·7)	13·5 (8·4 to 20·2)
	Marshall Islands	5·7% (4·5 to 6·9)	4·9% (4·1 to 5·7)	4·2% (3·5 to 4·9)	2·5% (1·7 to 3·4)	0·9% (0·7 to 1·3)	0·7% (0·5 to 1·0)	4·38 (3·38 to 5·64)	5·38 (3·45 to 7·82)	5·55 (3·53 to 8·03)	9·6 (7·4 to 12·3)	9·6 (6·2 to 14·0)	9·8 (6·2 to 14·1)
	Nauru	7·3% (5·6 to 8·9)	5·5% (4·3 to 6·7)	4·8% (3·9 to 5·7)	3·7% (2·3 to 5·0)	0·7% (0·4 to 1·0)	0·5% (0·4 to 0·7)	1·03 (0·584 to 1·52)	0·77 (0·441 to 1·23)	0·737 (0·427 to 1·15)	10·0 (5·7 to 14·8)	7·4 (4·2 to 11·8)	7·0 (4·0 to 10·9)
	Niue	5·9% (4·8 to 7·0)	4·2% (3·4 to 4·9)	3·6% (2·9 to 4·2)	0·5% (0·3 to 0·8)	0·3% (0·2 to 0·5)	0·3% (0·2 to 0·4)	0·309 (0·23 to 0·407)	0·181 (0·134 to 0·236)	0·185 (0·135 to 0·242)	13·3 (9·9 to 17·5)	11·0 (8·1 to 14·3)	11·1 (8·1 to 14·5)
	Northern Mariana Islands	6·5% (5·3 to 7·5)	7·3% (6·0 to 8·6)	7·1% (5·9 to 8·1)	2·4% (1·7 to 3·2)	2·6% (1·8 to 3·5)	2·6% (1·8 to 3·5)	5·06 (3·75 to 6·71)	7·73 (6·23 to 9·36)	8·07 (6·38 to 9·98)	11·1 (8·3 to 14·8)	16·7 (13·4 to 20·2)	19·0 (15·0 to 23·5)
	Palau	5·9% (4·4 to 7·2)	4·7% (3·9 to 5·7)	4·3% (3·6 to 5·0)	1·1% (0·7 to 1·5)	0·7% (0·4 to 0·9)	0·5% (0·3 to 0·7)	1·73 (1·19 to 2·41)	2·38 (1·67 to 3·32)	2·75 (1·91 to 3·85)	11·2 (7·7 to 15·6)	13·3 (9·3 to 18·5)	15·3 (10·6 to 21·4)
	Papua New Guinea	10·0% (8·4 to 10·9)	6·6% (5·8 to 7·0)	6·2% (5·8 to 6·5)	5·8% (3·9 to 7·2)	2·3% (1·3 to 3·0)	2·6% (1·9 to 3·1)	159 (109 to 231)	256 (173 to 363)	289 (200 to 410)	3·9 (2·7 to 5·7)	2·9 (2·0 to 4·1)	2·9 (2·0 to 4·2)
	Samoa	6·2% (5·0 to 7·6)	5·9% (4·7 to 7·0)	5·2% (4·3 to 6·3)	2·8% (1·8 to 3·8)	2·3% (1·5 to 3·2)	2·0% (1·3 to 2·8)	13·5 (9·71 to 18·3)	15·2 (11·2 to 19·8)	15·9 (11·7 to 21·2)	8·3 (5·9 to 11·2)	7·6 (5·6 to 10·0)	7·5 (5·5 to 10·0)
	Solomon Islands	12·2% (7·5 to 15·5)	6·8% (4·7 to 8·2)	6·3% (4·9 to 7·4)	4·3% (1·9 to 6·2)	1·5% (0·6 to 2·1)	1·3% (0·7 to 1·8)	41·5 (28·1 to 58·6)	58 (43·1 to 76·3)	59·3 (43·5 to 79·6)	12·2 (8·3 to 17·2)	9·5 (7·1 to 12·5)	9·0 (6·6 to 12·1)
	Tokelau	7·0% (5·7 to 8·4)	6·9% (5·6 to 8·4)	6·4% (5·2 to 7·7)	3·5% (2·3 to 4·8)	3·2% (2·1 to 4·6)	3·0% (2·0 to 4·1)	0·187 (0·14 to 0·248)	0·129 (0·0975 to 0·17)	0·138 (0·102 to 0·185)	11·1 (8·3 to 14·7)	9·8 (7·4 to 12·8)	9·8 (7·2 to 13·1)
	Tonga	8·0% (6·0 to 9·5)	4·4% (3·4 to 5·3)	4·0% (3·2 to 4·6)	0·9% (0·5 to 1·3)	0·4% (0·2 to 0·7)	0·4% (0·2 to 0·6)	18·9 (14·7 to 23·5)	18·3 (13·9 to 23·6)	18·3 (13·7 to 23·9)	19·5 (15·2 to 24·3)	17·6 (13·3 to 22·7)	17·9 (13·4 to 23·3)
	Tuvalu	8·0% (6·1 to 9·8)	6·5% (5·4 to 7·6)	5·8% (4·8 to 6·7)	4·1% (2·6 to 5·8)	1·8% (1·2 to 2·5)	1·5% (1·0 to 2·0)	1·43 (1·01 to 1·91)	1·24 (0·851 to 1·75)	1·27 (0·855 to 1·79)	15·3 (10·8 to 20·4)	11·0 (7·5 to 15·4)	10·8 (7·2 to 15·1)
	Vanuatu	8·8% (7·0 to 10·7)	5·9% (4·7 to 7·1)	5·2% (4·2 to 6·2)	5·0% (3·3 to 6·7)	1·6% (1·0 to 2·2)	1·5% (1·0 to 2·0)	16·9 (11·5 to 24·2)	25·6 (17 to 36·4)	27 (17·8 to 37·5)	11·1 (7·6 to 16·0)	9·4 (6·2 to 13·3)	9·2 (6·0 to 12·7)
Southeast Asia		7·1% (6·3 to 7·9)	5·2% (4·7 to 5·7)	4·8% (4·3 to 5·2)	4·4% (3·6 to 5·4)	1·5% (1·2 to 1·9)	0·7% (0·6 to 0·9)	49 300 (42 600 to 57 200)	58 900 (50 400 to 68 500)	63 700 (53 400 to 76 300)	10·6 (9·1 to 12·2)	9·1 (7·7 to 10·5)	9·4 (7·9 to 11·3)
	Cambodia	9·7% (7·9 to 11·8)	7·4% (6·1 to 8·6)	6·6% (5·5 to 7·6)	6·2% (4·6 to 8·1)	0·7% (0·5 to 1·0)	0·5% (0·4 to 0·7)	1870 (1440 to 2330)	2300 (1720 to 3030)	2450 (1770 to 3270)	18·0 (13·8 to 22·4)	14·7 (11·0 to 19·4)	14·7 (10·7 to 19·7)
	Indonesia	4·7% (4·1 to 5·3)	3·8% (3·3 to 4·2)	3·6% (3·2 to 4·0)	2·7% (2·1 to 3·3)	0·9% (0·7 to 1·0)	0·6% (0·5 to 0·8)	18 300 (14 700 to 22 100)	20 900 (16 900 to 25 300)	22 600 (18 400 to 27 500)	9·9 (8·0 to 11·9)	8·3 (6·7 to 10·0)	8·7 (7·1 to 10·6)
	Laos	11·7% (9·6 to 13·8)	9·4% (8·0 to 10·9)	8·7% (7·3 to 10·0)	9·0% (6·6 to 12·1)	2·5% (1·8 to 3·1)	2·1% (1·6 to 2·7)	526 (375 to 714)	519 (364 to 706)	519 (361 to 719)	12·7 (9·0 to 17·2)	7·6 (5·4 to 10·4)	7·3 (5·0 to 10·0)
	Malaysia	2·0% (1·8 to 2·2)	1·4% (1·2 to 1·5)	1·3% (1·1 to 1·4)	0·4% (0·3 to 0·5)	0·1% (0·1 to 0·1)	0·09% (0·07 to 0·12)	1260 (1110 to 1430)	2670 (2260 to 3120)	3000 (2300 to 3870)	7·2 (6·3 to 8·1)	8·9 (7·6 to 10·4)	9·6 (7·3 to 12·4)
	Maldives	4·7% (3·7 to 5·7)	2·8% (2·3 to 3·4)	2·9% (2·4 to 3·4)	3·6% (2·6 to 4·7)	0·6% (0·4 to 0·8)	0·5% (0·3 to 0·6)	12 (8·12 to 16)	12·1 (9·91 to 14·9)	15·1 (11·8 to 19)	5·4 (3·7 to 7·2)	2·8 (2·3 to 3·4)	3·0 (2·4 to 3·8)
	Mauritius	4·0% (3·4 to 4·7)	3·0% (2·5 to 3·6)	2·9% (2·4 to 3·3)	2·3% (1·7 to 3·1)	0·5% (0·3 to 0·7)	0·4% (0·3 to 0·6)	107 (85·8 to 127)	72·8 (57·5 to 91·4)	76·1 (55·8 to 103)	9·7 (7·8 to 11·6)	5·7 (4·5 to 7·2)	6·0 (4·4 to 8·0)
	Myanmar	3·2% (2·6 to 3·7)	2·6% (2·2 to 2·9)	2·4% (2·0 to 2·7)	2·2% (1·6 to 2·8)	0·8% (0·6 to 1·0)	0·6% (0·5 to 0·8)	6120 (4370 to 8380)	6590 (4760 to 8850)	6640 (4680 to 9380)	14·9 (10·6 to 20·4)	12·5 (9·0 to 16·7)	12·2 (8·6 to 17·2)
	Philippines	14·1% (12·2 to 16·3)	9·6% (8·4 to 10·8)	8·2% (7·2 to 9·4)	8·8% (7·2 to 10·7)	4·3% (3·5 to 5·2)	1·2% (1·0 to 1·4)	4960 (3900 to 6060)	5700 (4910 to 6690)	6060 (4760 to 7660)	7·8 (6·2 to 9·6)	5·4 (4·7 to 6·4)	5·4 (4·2 to 6·8)
	Seychelles	4·3% (3·5 to 5·1)	3·3% (2·7 to 3·7)	3·2% (2·7 to 3·8)	2·3% (1·7 to 3·1)	0·4% (0·3 to 0·5)	0·3% (0·2 to 0·4)	9·27 (7·47 to 11·3)	9·69 (7·69 to 12·1)	11·3 (8·7 to 14·4)	12·7 (10·2 to 15·4)	9·8 (7·8 to 12·3)	11·1 (8·5 to 14·1)
	Sri Lanka	2·3% (2·0 to 2·7)	1·6% (1·5 to 1·8)	1·5% (1·3 to 1·7)	1·6% (1·2 to 2·0)	0·08% (0·06 to 0·10)	0·07% (0·06 to 0·10)	1550 (1230 to 1870)	1260 (913 to 1690)	1270 (879 to 1800)	9·0 (7·1 to 10·9)	5·9 (4·3 to 7·9)	5·8 (4·0 to 8·2)
	Thailand	6·8% (6·3 to 7·5)	5·0% (4·6 to 5·3)	4·8% (4·4 to 5·1)	3·0% (2·4 to 3·8)	0·4% (0·3 to 0·5)	0·4% (0·3 to 0·4)	6980 (5860 to 8240)	12 000 (9590 to 14 700)	13 200 (9240 to 18 300)	12·3 (10·3 to 14·5)	17·2 (13·8 to 21·2)	18·9 (13·2 to 26·1)
	Timor-Leste	6·2% (5·0 to 7·5)	4·7% (3·8 to 5·5)	4·1% (3·3 to 4·9)	4·7% (3·3 to 6·2)	1·5% (1·1 to 2·0)	1·3% (1·0 to 1·8)	55 (38·4 to 81)	91·7 (46·9 to 140)	81·6 (46·1 to 122)	7·0 (4·9 to 10·3)	7·4 (3·8 to 11·4)	6·1 (3·5 to 9·2)
	Vietnam	11·4% (10·3 to 12·4)	7·2% (6·9 to 7·6)	6·6% (6·3 to 6·9)	7·6% (5·8 to 9·5)	0·8% (0·6 to 1·0)	0·7% (0·6 to 0·9)	7440 (5370 to 10 100)	6790 (5040 to 9090)	7600 (5420 to 10 500)	11·0 (7·9 to 14·9)	7·3 (5·4 to 9·7)	7·9 (5·6 to 10·9)
**Sub-Saharan Africa**		**10·5% (9·3 to 11·8)**	**7·4% (6·5 to 8·3)**	**6·7% (6·0 to 7·5)**	**8·0% (6·6 to 9·5)**	**3·1% (2·5 to 3·7)**	**2·8% (2·3 to 3·4)**	**52 000 (41 200 to 64 200)**	**70 100 (57 500 to 85 100)**	**71 500 (58 100 to 87 500)**	**10·6 (8·4 to 13·1)**	**7·2 (5·9 to 8·7)**	**6·6 (5·4 to 8·1)**
Central sub-Saharan Africa		8·7% (7·5 to 10·1)	7·1% (6·1 to 8·1)	6·4% (5·5 to 7·2)	6·7% (5·2 to 8·4)	3·1% (2·4 to 3·8)	2·7% (2·1 to 3·4)	5280 (3800 to 7200)	7730 (5530 to 10 300)	7030 (4880 to 9720)	9·5 (6·8 to 13·0)	6·6 (4·7 to 8·7)	5·3 (3·7 to 7·4)
	Angola	18·7% (16·2 to 21·3)	13·2% (11·4 to 14·9)	12·0% (10·4 to 13·6)	15·1% (11·3 to 18·9)	7·5% (5·7 to 9·4)	6·8% (5·2 to 8·6)	1160 (751 to 1690)	1800 (1290 to 2350)	1740 (1220 to 2390)	11·3 (7·3 to 16·4)	6·8 (4·9 to 8·9)	5·8 (4·1 to 7·9)
	Central African Republic	13·0% (12·6 to 13·5)	10·2% (9·8 to 10·5)	9·5% (9·2 to 9·8)	7·9% (7·0 to 9·0)	4·0% (3·5 to 4·5)	3·3% (2·9 to 3·7)	299 (186 to 448)	397 (225 to 652)	363 (207 to 601)	10·9 (6·8 to 16·3)	8·2 (4·6 to 13·4)	6·9 (3·9 to 11·3)
	Congo (Brazzaville)	13·0% (10·6 to 15·4)	8·1% (6·7 to 9·6)	7·4% (6·2 to 8·6)	10·4% (7·8 to 13·3)	2·8% (2·1 to 3·6)	2·6% (1·9 to 3·4)	240 (165 to 331)	349 (234 to 484)	312 (198 to 442)	9·8 (6·7 to 13·5)	7·2 (4·9 to 10·0)	5·9 (3·8 to 8·4)
	Democratic Republic of the Congo	5·6% (4·5 to 6·8)	4·9% (4·2 to 5·8)	4·3% (3·6 to 5·0)	4·3% (3·1 to 5·6)	1·5% (1·1 to 1·9)	1·2% (0·9 to 1·6)	3440 (2380 to 4810)	5030 (3400 to 6930)	4460 (2970 to 6410)	8·9 (6·2 to 12·5)	6·4 (4·3 to 8·8)	5·1 (3·4 to 7·3)
	Equatorial Guinea	2·2% (1·9 to 2·6)	1·4% (1·2 to 1·6)	1·3% (1·1 to 1·4)	1·7% (1·3 to 2·2)	0·8% (0·6 to 1·1)	0·5% (0·4 to 0·6)	37·9 (23·9 to 58·4)	39·2 (23·5 to 61·9)	46·7 (27·9 to 74·1)	8·8 (5·6 to 13·6)	3·1 (1·9 to 4·9)	3·3 (2·0 to 5·2)
	Gabon	9·8% (8·9 to 10·8)	5·9% (5·4 to 6·4)	5·2% (4·7 to 5·7)	7·6% (6·1 to 9·6)	1·5% (1·1 to 1·9)	1·2% (0·9 to 1·5)	105 (71·4 to 147)	115 (77·4 to 168)	111 (71·2 to 166)	10·6 (7·2 to 14·8)	7·0 (4·7 to 10·2)	6·3 (4·1 to 9·5)
Eastern sub-Saharan Africa		7·8% (6·7 to 8·8)	5·4% (4·7 to 6·0)	4·8% (4·2 to 5·4)	5·2% (4·2 to 6·4)	1·3% (1·1 to 1·6)	1·1% (0·9 to 1·4)	13 300 (9790 to 17 800)	17 900 (14 300 to 22 400)	18 800 (15 100 to 23 300)	7·0 (5·1 to 9·4)	4·8 (3·9 to 6·0)	4·6 (3·7 to 5·7)
	Burundi	4·0% (3·8 to 4·2)	2·5% (2·4 to 2·6)	2·1% (2·1 to 2·2)	2·5% (2·0 to 3·0)	0·2% (0·2 to 0·2)	0·2% (0·1 to 0·2)	362 (207 to 562)	350 (197 to 631)	358 (201 to 632)	6·5 (3·7 to 10·1)	3·4 (1·9 to 6·1)	3·0 (1·7 to 5·3)
	Comoros	6·3% (5·0 to 7·9)	4·9% (4·0 to 6·0)	4·5% (3·6 to 5·5)	4·2% (3·0 to 5·8)	0·9% (0·7 to 1·2)	0·7% (0·5 to 1·0)	25·1 (11·1 to 44·2)	34·3 (22·5 to 51)	34·9 (21·8 to 51·2)	5·4 (2·4 to 9·5)	5·0 (3·3 to 7·5)	4·9 (3·1 to 7·2)
	Djibouti	6·6% (5·2 to 8·2)	5·7% (4·6 to 6·9)	5·2% (4·2 to 6·4)	4·5% (3·2 to 6·2)	2·0% (1·5 to 2·7)	1·8% (1·2 to 2·5)	20·9 (13·5 to 35·8)	59·9 (35·1 to 98·2)	54·9 (32·7 to 88·9)	4·3 (2·8 to 7·4)	5·5 (3·3 to 9·1)	4·6 (2·7 to 7·4)
	Eritrea	7·9% (6·2 to 9·5)	5·1% (4·2 to 6·3)	4·3% (3·5 to 5·2)	5·0% (3·7 to 6·7)	0·5% (0·4 to 0·7)	0·4% (0·3 to 0·6)	181 (108 to 281)	277 (174 to 423)	307 (193 to 460)	6·0 (3·6 to 9·4)	4·5 (2·8 to 6·9)	4·6 (2·9 to 6·9)
	Ethiopia	5·5% (4·7 to 6·3)	3·9% (3·4 to 4·5)	3·8% (3·3 to 4·4)	3·7% (3·0 to 4·5)	1·5% (1·2 to 1·8)	1·3% (1·1 to 1·6)	4510 (2450 to 6610)	6360 (5130 to 8020)	6350 (5010 to 7990)	8·8 (4·8 to 12·9)	6·5 (5·3 to 8·2)	5·9 (4·7 to 7·4)
	Kenya	4·0% (3·4 to 4·5)	3·2% (2·8 to 3·6)	3·0% (2·6 to 3·3)	1·9% (1·5 to 2·3)	0·5% (0·4 to 0·6)	0·5% (0·4 to 0·7)	1580 (1010 to 2630)	2810 (2090 to 4010)	3090 (2220 to 4430)	6·8 (4·3 to 11·4)	6·1 (4·5 to 8·7)	6·2 (4·4 to 8·8)
	Madagascar	7·9% (6·7 to 9·2)	5·3% (4·6 to 6·0)	4·6% (4·0 to 5·2)	5·1% (3·7 to 6·8)	1·1% (0·8 to 1·4)	0·9% (0·6 to 1·1)	834 (605 to 1090)	865 (587 to 1240)	960 (642 to 1410)	7·0 (5·1 to 9·1)	3·6 (2·4 to 5·1)	3·6 (2·4 to 5·3)
	Malawi	11·5% (9·1 to 14·0)	9·1% (7·3 to 10·8)	7·9% (6·5 to 9·3)	8·4% (6·1 to 11·1)	1·6% (1·2 to 2·1)	1·6% (1·2 to 2·1)	850 (593 to 1170)	650 (446 to 919)	713 (480 to 996)	8·9 (6·2 to 12·2)	3·9 (2·7 to 5·6)	3·9 (2·6 to 5·4)
	Mozambique	15·2% (12·3 to 17·9)	9·3% (7·7 to 11·0)	8·0% (6·6 to 9·4)	11·4% (8·4 to 15·1)	1·8% (1·3 to 2·3)	0·8% (0·6 to 1·1)	609 (382 to 925)	706 (449 to 1050)	736 (458 to 1090)	4·7 (2·9 to 7·1)	2·7 (1·7 to 4·0)	2·5 (1·6 to 3·7)
	Rwanda	6·8% (5·4 to 8·4)	4·4% (3·6 to 5·4)	3·8% (3·1 to 4·5)	5·1% (3·8 to 6·7)	0·4% (0·3 to 0·5)	0·4% (0·3 to 0·5)	427 (287 to 596)	317 (218 to 440)	343 (235 to 480)	6·0 (4·0 to 8·3)	2·7 (1·9 to 3·8)	2·7 (1·9 to 3·8)
	Somalia	13·0% (11·9 to 14·2)	11·8% (10·8 to 12·9)	11·1% (10·1 to 12·0)	8·5% (7·1 to 10·2)	5·2% (4·3 to 6·2)	4·4% (3·6 to 5·3)	717 (456 to 1170)	1470 (889 to 2500)	1600 (993 to 2650)	10·0 (6·4 to 16·4)	8·4 (5·1 to 14·3)	7·8 (4·9 to 13·0)
	South Sudan	8·1% (6·4 to 9·9)	7·1% (5·6 to 8·7)	6·5% (5·2 to 8·1)	5·8% (4·2 to 7·6)	3·0% (2·2 to 4·0)	2·6% (1·9 to 3·4)	358 (229 to 561)	525 (315 to 854)	541 (327 to 865)	6·1 (3·9 to 9·6)	5·7 (3·4 to 9·3)	5·8 (3·5 to 9·3)
	Uganda	10·8% (9·5 to 12·2)	5·7% (5·1 to 6·2)	4·8% (4·3 to 5·2)	8·1% (6·4 to 10·2)	1·2% (0·9 to 1·5)	0·9% (0·7 to 1·1)	825 (573 to 1190)	1110 (781 to 1540)	1210 (821 to 1690)	4·8 (3·3 to 6·9)	3·0 (2·1 to 4·2)	2·9 (2·0 to 4·1)
	Tanzania	7·1% (6·8 to 7·5)	4·2% (4·1 to 4·4)	3·5% (3·4 to 3·7)	4·0% (3·3 to 5·0)	0·5% (0·4 to 0·6)	0·4% (0·3 to 0·5)	1370 (972 to 1850)	1520 (990 to 2150)	1600 (1030 to 2300)	5·3 (3·8 to 7·2)	3·0 (1·9 to 4·2)	2·8 (1·8 to 4·1)
	Zambia	11·2% (8·7 to 13·8)	7·3% (5·9 to 8·7)	6·7% (5·4 to 8·0)	7·9% (5·5 to 10·4)	1·2% (0·9 to 1·6)	0·9% (0·6 to 1·1)	623 (440 to 863)	879 (631 to 1200)	868 (600 to 1220)	7·8 (5·5 to 10·9)	5·4 (3·9 to 7·4)	4·8 (3·3 to 6·7)
Southern sub-Saharan Africa		8·4% (7·6 to 9·2)	4·7% (4·3 to 5·1)	4·5% (4·1 to 4·9)	7·5% (6·4 to 8·7)	1·8% (1·6 to 2·1)	1·8% (1·5 to 2·1)	3150 (2360 to 4110)	3500 (3040 to 4080)	3520 (3000 to 4150)	6·0 (4·5 to 7·8)	4·7 (4·0 to 5·4)	4·5 (3·8 to 5·3)
	Botswana	8·7% (6·8 to 10·6)	4·6% (3·8 to 5·5)	4·1% (3·3 to 5·0)	6·7% (4·9 to 9·0)	1·2% (0·9 to 1·6)	1·4% (1·0 to 1·8)	79·5 (45·9 to 128)	82·8 (53 to 127)	91·1 (56·6 to 138)	6·1 (3·5 to 9·8)	3·8 (2·4 to 5·8)	3·9 (2·4 to 5·9)
	Eswatini	10·8% (8·6 to 12·9)	5·6% (4·5 to 6·7)	4·9% (4·0 to 5·8)	8·7% (6·3 to 11·2)	1·2% (0·8 to 1·5)	1·1% (0·8 to 1·5)	46·8 (32·7 to 66·1)	90·1 (40 to 150)	89·8 (40·7 to 148)	5·8 (4·1 to 8·2)	8·1 (3·6 to 13·5)	7·9 (3·6 to 13·0)
	Lesotho	11·6% (9·2 to 13·8)	7·7% (6·3 to 9·3)	6·9% (5·5 to 8·3)	9·5% (7·1 to 12·5)	1·1% (0·8 to 1·5)	0·6% (0·4 to 0·8)	140 (82 to 217)	171 (93·2 to 265)	168 (91·5 to 258)	7·7 (4·5 to 12·0)	8·4 (4·6 to 13·1)	8·1 (4·4 to 12·3)
	Namibia	7·6% (7·5 to 7·8)	4·8% (4·8 to 4·9)	4·2% (4·1 to 4·2)	5·8% (5·2 to 6·4)	0·6% (0·5 to 0·6)	0·4% (0·4 to 0·5)	71·6 (45 to 108)	107 (72·7 to 150)	82·4 (55 to 120)	5·1 (3·2 to 7·7)	4·7 (3·2 to 6·6)	3·4 (2·3 to 5·0)
	South Africa	6·7% (5·9 to 7·6)	3·5% (3·1 to 3·9)	3·5% (3·1 to 3·9)	6·6% (5·5 to 7·9)	2·0% (1·6 to 2·4)	2·0% (1·6 to 2·3)	2010 (1540 to 2640)	2190 (1960 to 2450)	2220 (1930 to 2520)	5·5 (4·2 to 7·2)	4·1 (3·7 to 4·6)	4·0 (3·5 to 4·5)
	Zimbabwe	13·7% (13·0 to 14·2)	8·8% (8·5 to 9·1)	7·8% (7·5 to 8·0)	9·7% (8·2 to 11·5)	1·8% (1·5 to 2·1)	1·8% (1·5 to 2·1)	803 (570 to 1150)	857 (606 to 1170)	861 (592 to 1200)	7·8 (5·5 to 11·1)	6·1 (4·3 to 8·3)	5·7 (3·9 to 8·0)
Western sub-Saharan Africa		14·4% (12·8 to 16·1)	9·9% (8·7 to 11·0)	9·0% (8·0 to 10·0)	11·3% (9·3 to 13·3)	4·8% (3·9 to 5·7)	4·4% (3·6 to 5·3)	30 200 (22 600 to 39 900)	41 000 (32 400 to 51 700)	42 200 (32 800 to 53 900)	15·7 (11·7 to 20·7)	10·0 (7·9 to 12·7)	9·2 (7·2 to 11·8)
	Benin	13·9% (11·0 to 16·5)	8·6% (6·8 to 10·2)	7·6% (6·2 to 8·8)	12·2% (8·2 to 15·5)	3·3% (2·2 to 4·3)	2·8% (1·8 to 3·6)	737 (541 to 979)	994 (720 to 1300)	1020 (715 to 1380)	15·2 (11·1 to 20·2)	8·9 (6·5 to 11·7)	8·1 (5·6 to 10·9)
	Burkina Faso	16·6% (13·3 to 19·4)	14·0% (11·9 to 16·2)	12·2% (10·1 to 14·1)	13·7% (9·8 to 17·7)	1·9% (1·5 to 2·5)	1·8% (1·2 to 2·3)	1300 (1000 to 1670)	1460 (870 to 2060)	1400 (825 to 2040)	13·6 (10·5 to 17·5)	7·3 (4·4 to 10·3)	6·2 (3·6 to 9·0)
	Cameroon	7·4% (7·1 to 7·7)	4·7% (4·1 to 5·1)	4·2% (3·8 to 4·5)	5·7% (5·0 to 6·5)	1·4% (1·1 to 1·6)	1·0% (0·8 to 1·2)	1400 (973 to 2040)	1990 (1350 to 2770)	2090 (1400 to 2990)	13·5 (9·4 to 19·6)	7·7 (5·2 to 10·7)	7·2 (4·8 to 10·3)
	Cape Verde	10·5% (8·5 to 12·6)	7·2% (6·0 to 8·4)	6·5% (5·0 to 7·6)	8·6% (6·4 to 11·1)	0·8% (0·6 to 1·1)	0·7% (0·5 to 0·9)	36·9 (28·2 to 51·6)	63·7 (53·1 to 75·4)	73·4 (57·8 to 92·2)	10·5 (8·0 to 14·7)	11·8 (9·8 to 13·9)	13·0 (10·3 to 16·4)
	Chad	16·6% (13·2 to 19·8)	13·6% (10·1 to 16·3)	12·4% (9·9 to 14·5)	14·8% (10·2 to 19·5)	8·8% (5·6 to 11·2)	7·9% (5·7 to 10·0)	958 (647 to 1360)	1720 (1260 to 2300)	1790 (1300 to 2390)	15·9 (10·7 to 22·5)	12·2 (8·9 to 16·2)	10·9 (7·9 to 14·6)
	Côte d'Ivoire	14·1% (13·6 to 14·7)	8·1% (7·9 to 8·2)	6·9% (6·7 to 7·0)	10·9% (9·3 to 12·3)	1·8% (1·6 to 2·0)	1·2% (1·1 to 1·4)	1480 (1110 to 1970)	2110 (1470 to 2910)	2170 (1470 to 3020)	12·1 (9·1 to 16·1)	8·8 (6·1 to 12·1)	8·3 (5·6 to 11·5)
	The Gambia	7·4% (7·0 to 7·8)	3·1% (3·0 to 3·1)	3·0% (2·9 to 3·1)	1·3% (1·1 to 1·5)	0·5% (0·4 to 0·6)	0·5% (0·4 to 0·6)	162 (114 to 219)	314 (230 to 417)	386 (278 to 516)	16·4 (11·5 to 22·1)	15·4 (11·3 to 20·5)	17·2 (12·4 to 23·0)
	Ghana	17·1% (15·7 to 18·5)	10·5% (9·5 to 11·3)	9·1% (8·3 to 9·9)	13·0% (10·8 to 15·4)	1·2% (1·0 to 1·5)	0·6% (0·5 to 0·8)	2340 (1650 to 3290)	2910 (2220 to 3720)	3120 (2270 to 4150)	15·6 (11·0 to 21·9)	10·1 (7·7 to 12·9)	9·9 (7·2 to 13·2)
	Guinea	17·0% (13·3 to 19·8)	13·3% (10·5 to 15·5)	11·7% (9·5 to 13·6)	14·2% (9·0 to 18·1)	7·1% (4·5 to 9·2)	6·4% (4·3 to 8·1)	1560 (1230 to 1920)	2400 (1820 to 3140)	2270 (1670 to 3020)	25·2 (19·9 to 31·1)	21·1 (16·0 to 27·6)	17·9 (13·2 to 23·9)
	Guinea-Bissau	16·7% (13·4 to 20·1)	11·9% (9·8 to 14·0)	10·8% (9·0 to 12·4)	13·7% (9·7 to 17·7)	3·5% (2·5 to 4·5)	3·8% (2·8 to 4·7)	195 (134 to 272)	260 (191 to 346)	241 (172 to 331)	19·3 (13·3 to 27·0)	14·9 (10·9 to 19·8)	12·7 (9·1 to 17·4)
	Liberia	14·3% (11·7 to 16·9)	11·0% (8·8 to 13·2)	10·0% (8·2 to 11·8)	11·7% (8·6 to 14·7)	3·5% (2·5 to 4·7)	2·2% (1·5 to 2·8)	429 (327 to 558)	545 (397 to 740)	518 (365 to 733)	21·9 (16·7 to 28·4)	12·3 (8·9 to 16·6)	10·8 (7·6 to 15·3)
	Mali	6·8% (5·5 to 7·9)	5·4% (4·5 to 6·2)	5·0% (3·9 to 6·0)	5·3% (3·7 to 6·8)	2·6% (1·9 to 3·2)	2·3% (1·4 to 3·1)	1880 (1360 to 2590)	1920 (1400 to 2520)	2210 (1570 to 2960)	21·7 (15·7 to 29·8)	10·1 (7·4 to 13·3)	10·1 (7·2 to 13·5)
	Mauritania	20·3% (19·8 to 20·8)	12·7% (12·5 to 12·9)	11·7% (11·5 to 11·9)	15·0% (13·1 to 16·4)	4·4% (3·9 to 4·8)	3·4% (3·0 to 3·7)	362 (281 to 468)	336 (241 to 460)	335 (220 to 475)	17·5 (13·6 to 22·6)	9·0 (6·5 to 12·4)	8·3 (5·5 to 11·8)
	Niger	14·8% (14·2 to 15·5)	10·1% (9·8 to 10·5)	8·7% (8·4 to 9·0)	11·6% (10·1 to 13·0)	3·8% (3·3 to 4·3)	3·3% (2·8 to 3·7)	935 (658 to 1280)	1650 (1070 to 2440)	1690 (1060 to 2510)	11·7 (8·2 to 15·9)	8·3 (5·3 to 12·2)	7·2 (4·6 to 10·8)
	Nigeria	15·3% (13·3 to 17·6)	10·6% (9·2 to 12·2)	9·9% (8·6 to 11·3)	11·8% (9·5 to 14·3)	6·9% (5·7 to 8·3)	6·6% (5·4 to 8·0)	14 500 (9820 to 20 700)	19 700 (13 700 to 28 400)	20 200 (13 500 to 29 600)	16·0 (10·9 to 23·0)	10·2 (7·1 to 14·7)	9·4 (6·3 to 13·8)
	São Tomé and Príncipe	13·1% (10·6 to 15·4)	8·0% (6·6 to 9·3)	7·1% (5·8 to 8·4)	10·4% (7·8 to 13·3)	1·1% (0·8 to 1·4)	0·9% (0·7 to 1·2)	23·5 (16·2 to 33·1)	21·8 (15·4 to 29·7)	23·3 (16 to 32)	19·3 (13·3 to 27·2)	11·3 (8·0 to 15·5)	11·3 (7·8 to 15·6)
	Senegal	11·2% (9·1 to 13·3)	7·3% (5·8 to 8·8)	6·4% (5·3 to 7·6)	8·5% (6·4 to 11·1)	1·2% (0·9 to 1·6)	1·0% (0·7 to 1·2)	860 (627 to 1150)	1120 (797 to 1510)	1190 (800 to 1670)	11·3 (8·2 to 15·1)	8·0 (5·7 to 10·9)	7·9 (5·3 to 11·0)
	Sierra Leone	17·4% (12·8 to 20·4)	10·8% (8·1 to 12·7)	9·2% (7·1 to 10·7)	16·1% (9·9 to 20·1)	2·6% (1·7 to 3·3)	2·1% (1·4 to 2·7)	671 (474 to 934)	691 (498 to 937)	681 (481 to 932)	18·4 (13·0 to 25·6)	9·3 (6·7 to 12·7)	8·2 (5·8 to 11·2)
	Togo	7·9% (6·9 to 8·9)	5·8% (5·0 to 6·5)	5·0% (4·4 to 5·7)	6·1% (4·8 to 7·7)	1·1% (0·8 to 1·4)	0·8% (0·6 to 1·0)	441 (325 to 568)	721 (528 to 974)	713 (505 to 966)	12·0 (8·9 to 15·5)	10·0 (7·3 to 13·4)	9·0 (6·4 to 12·2)

Data in parentheses are 95% uncertainty intervals.

In 2019, the global HBsAg prevalence among infants and children younger than 5 years was 1·0% (95% UI 0·8 to 1·2; table). This was a reduction of 76·8% (76·2 to 77·5) from 1990 and 5·2% (13·6 to 16·7) from 2015 (appendix table S2). In the absence of HepB3, in 2019 the global HBsAg prevalence in children younger than 5 years would have been 3·9% (3·2 to 4·7). Similar to all-age chronic HBV prevalence, all regions experienced reductions in under-5 prevalence both since 1990 and since 2015. Notably, the largest regional percentage change between 1990 and 2019 was seen in the Western Pacific region (−93·6% [–94·0 to −93·3]), declining from the highest under-5 prevalence at the beginning of that period. The highest under-5 HBsAg prevalence in 2019 was in the African region (2·7% [2·2 to 3·2]), while the lowest was in the region of the Americas (0·08% [0·06 to 0·11]; table). Country-level variation in HBsAg prevalence in children younger than 5 years in 2019 is shown in figure 1B.

### HBV-related mortality

Globally, HBV-related diseases resulted in an estimated 555 000 deaths (95% UI 487 000 to 630 000) in 2019, accounting for 48·8% (44·6 to 52·7) of all hepatitis-related deaths (appendix figure S1). Hepatitis B was the leading aetiology responsible for deaths from liver cancer (39·5% [35·2 to 44·4]) and the third largest contributor to deaths from cirrhosis (22·5% [19·3 to 26·0]). HBV-related cirrhosis was responsible for 331 000 (279 000 to 392 000) global deaths, liver cancer was responsible for 192 000 (162 000 to 224 000) global deaths, and acute hepatitis was responsible for 32 500 (23 900 to 44 700) global deaths. The absolute number of HBV-related deaths in 2019 increased by 5·9% (−5·6 to 19·2) from 1990 and by 2·9% (−5·9 to 11·3) from 2015 (appendix table S3). The WHO South-East Asia and Western Pacific regions accounted for 67·5% (63·9 to 71·1) of HBV-related deaths worldwide. Notably, 70% of DALYs attributed to HBV were concentrated in ten countries: China, India, Indonesia, Nigeria, Pakistan, Egypt, Thailand, South Korea, Bangladesh, and Myanmar (appendix figure S2).

In 2019, HBV-related diseases resulted in 7·2 (95% UI 6·3 to 8·1) deaths per 100 000 people worldwide (table), a decline of 26·8% (17·6 to 34·7) since 1990 and 1·4% (−9·9 to 6·6) since 2015 (appendix table S3). Geographically, HBV-related death rates varied widely across regions, with the highest all-age death rates in the Western Pacific region (10·7 [9·0 to 12·4] per 100 000), and South-East Asia region (8·4 [7·1 to 10·0] per 100 000), and the lowest death rate in the Region of the Americas (1·8 [1·6 to 2·1] per 100 000; table). There was widespread heterogeneity across countries, with the highest all-age death rate in Mongolia (29·1 [20·3 to 39·9] per 100 000), and the lowest death rate in Colombia (0·7 [0·5 to 1·0] per 100 000; table, figure 1C). Although the highest death rate in 2019 was in the Western Pacific region, this region also had the largest percentage change in death rates between 1990 and 2019 (−37·5% [–48·8 to −22·8]; appendix table S3).

### Distribution of prevalence, death rates, and DALY rates by age

**Figure 2:**
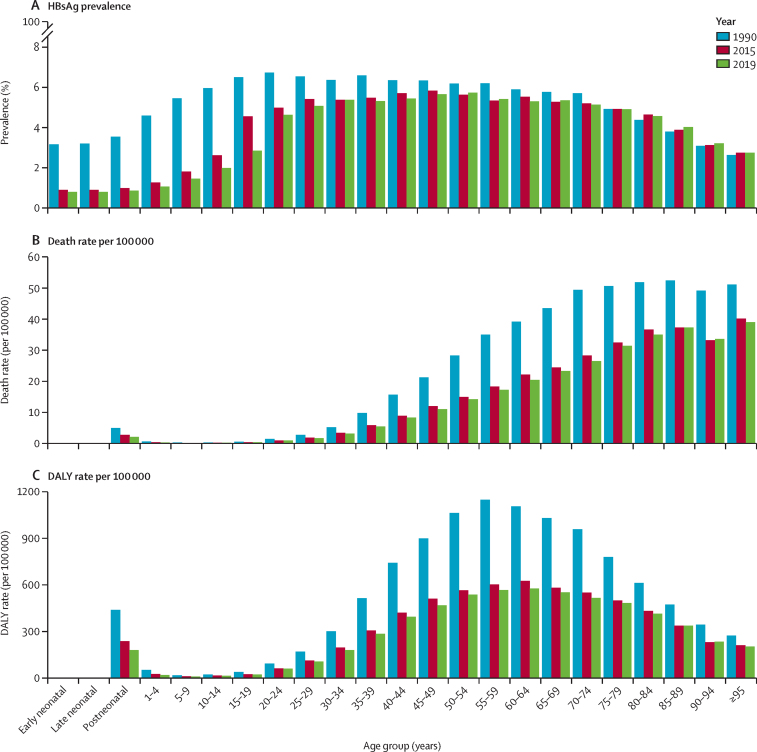
Global age-specific HBsAg prevalence (A), HBV-related death rate per 100 000 (B), and HBV-related DALY rate per 100 000 (C), in 1990, 2015, and 2019 HBV=hepatitis B virus. DALY=disability-adjusted life-year.

Age-specific HBsAg prevalence and HBV-related death rates and DALY rates generally declined over time (figure 2). Decreases were seen for many age groups from 2015 to 2019, but more substantial decreases were seen for most age groups from 1990 to 2015. The most notable reductions in HBsAg prevalence were seen in the youngest age groups, particularly people younger than 39 years. Improvements in age-specific death rates and DALY rates were most marked in the middle age groups (35–69 years).

### Burden by SDI

**Figure 3:**
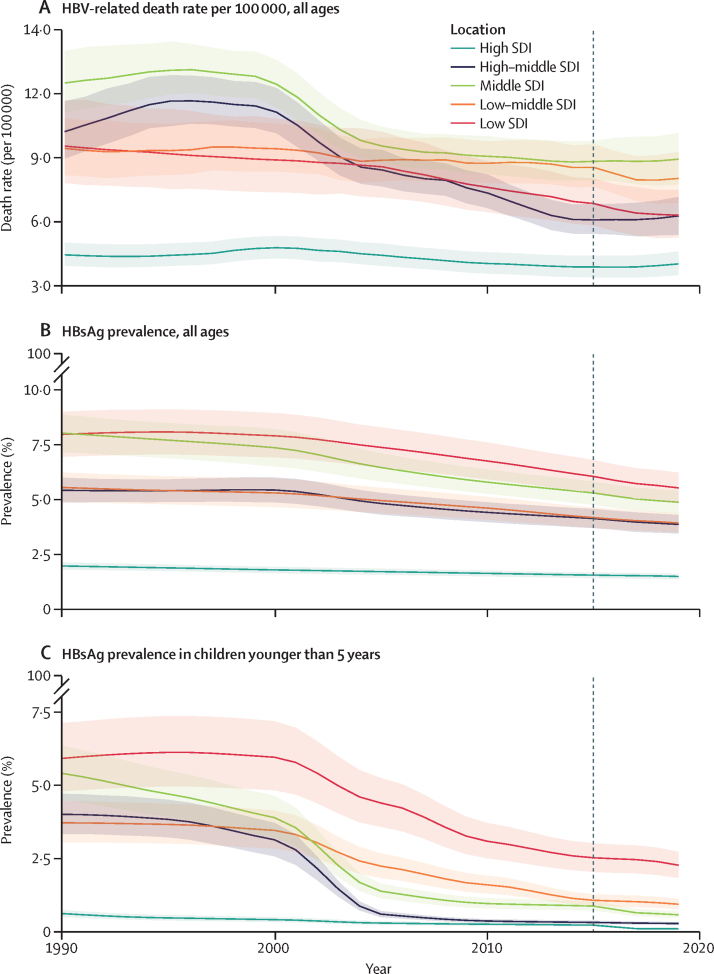
HBV-related death rates for all ages (A), HBsAg prevalence for all ages (B), and HBsAg prevalence in children younger than 5 years (C) by SDI quintile, 1990–2019 The vertical dashed line indicates the WHO-GHSS baseline year of 2015. The shading represents 95% uncertainty intervals. HBV=hepatitis B virus. SDI=Socio-demographic Index. WHO-GHSS=WHO Global Health Sector Strategy on Viral Hepatitis.

The all-age HBV-related death rate remained highest in the middle SDI quintile and lowest in the high SDI quintile between 1990 and 2019 (figure 3). In this period, the largest percentage decline in death rates was seen in the high-middle-income quintile, decreasing by 38·7% (95% UI 28·0–47·5). Although death rates decreased in all SDI quintiles since 1990, decreases were only seen in the low SDI and low-middle SDI quintiles between 2015 and 2019. All SDI quintiles showed a decrease in all-age and under-5 prevalence since 1990 and 2015. In 2019, under-5 prevalence was highest in the low SDI quintile. The high-middle SDI quintile saw the largest percentage change in under-5 prevalence since 1990, decreasing by 93·7% (93·3–94·0).

### Percentage change in HBV-related mortality by WHO region

**Figure 4:**
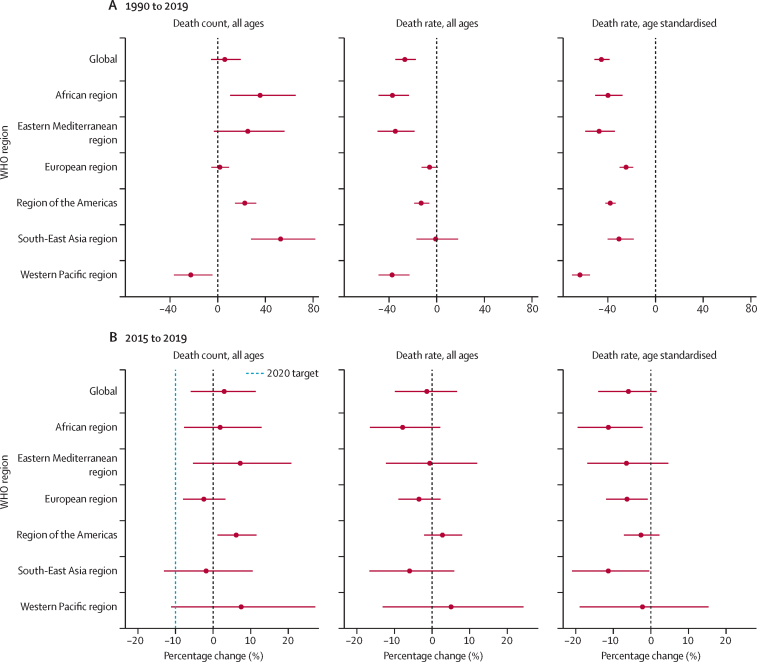
Changes in mortality by WHO region over time Percentage change in HBV-related death counts, all ages from 1990 to 2019 (A) and from 2015 to 2019 (B). The dashed line indicates the WHO-GHSS 2020 target of a 10% reduction in death counts. Error bars depict 95% uncertainty intervals. WHO-GHSS=WHO Global Health Sector Strategy on Viral Hepatitis.

We examined the percentage change by WHO region for death counts, all-age death rates, and age-standardised death rates due to HBV-related diseases from 1990 to 2019, to evaluate longer term changes, and from 2015 to 2019, to assess progress since the adoption of the WHO-GHSS goals in 2015 (figure 4). Since 1990, absolute death counts due to HBV increased in all regions except for the Western Pacific region. Since 2015, the European and South-East Asia regions were the only regions with decreased death counts, although neither achieved the WHO-GHSS goal of a 10% reduction over the time period. All-age death rates decreased in all regions since 1990 (ranging from a 0·9% decrease in the South-East Asia region to a 37·5% decrease in the Western Pacific region) and half of regions since 2015 (ranging from a 0·6% decrease in the Eastern Mediterranean region to a 7·8% decrease in the African region). Age-standardised HBV-related death rates, however, decreased across all regions in both time periods of interest.

### Monitoring progress towards goals

**Figure 5:**
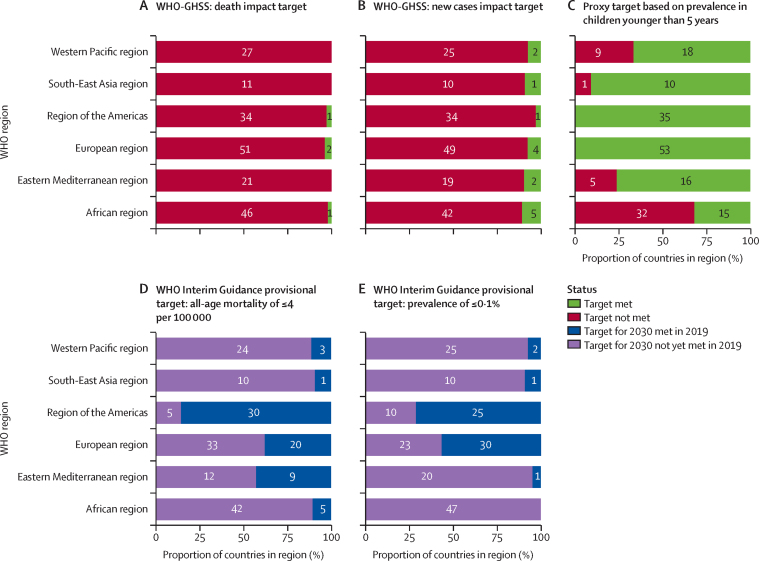
Proportion of countries by WHO region that met or exceeded WHO-GHSS and WHO Interim Guidance provisional targets (A) WHO-GHSS 2020 target of 10% reduction in deaths due to viral hepatitis from 2015 to 2020. (B) WHO-GHSS 2020 target of 30% reduction in new cases of viral hepatitis from 2015 to 2020. (C) Proxy target of less than 1% prevalence of viral hepatitis in infants and children younger than 5 years by 2020. (D) WHO Interim Guidance provisional mortality target of less than or equal to four deaths per 100 000. (E) WHO-GHSS proxy target of less than or equal to 0·1% prevalence in infants and children younger than 5 years by 2030. The numbers inside the bars represent the number of countries by WHO region that met or did not meet the specific targets. WHO-GHSS=WHO Global Health Sector Strategy on Viral Hepatitis.

Of the 194 WHO locations for which GBD generates estimates, only four countries reached or exceeded the WHO-GHSS 2020 interim impact target of a 10% reduction in deaths between 2015 and 2019 with high certainty (defined as a 95% probability of goal attainment; figure 5; appendix table S4). No countries in the Western Pacific, South-East Asia, or Eastern Mediterranean regions achieved the interim impact target of a 10% reduction in deaths. 15 countries met or surpassed the WHO-GHSS 2020 interim impact target of a 30% reduction in new cases between 2015 and 2019 (appendix table S4). 147 countries met or exceeded the interim proxy target of less than 1% prevalence in infants and children younger than 5 years by 2020 (appendix table S4). All countries in the region of the Americas and the European region achieved this proxy target with high certainty. The African region had the lowest percentage of countries meeting this proxy target (15 [32%] of 47).

Although many countries had not met the interim targets, some had already met targets for 2030 with high certainty. In 2019, 59 of 194 locations met or exceeded the proxy target of no more than 0·1% HBsAg seroprevalence in infants and children younger than 5 years, and 68 of 194 locations had already achieved an all-age mortality rate of less than or equal to four deaths per 100 000 people per year (figure 5; appendix table S5). No countries met the impact targets of a 65% reduction in deaths and a 95% reduction in new cases (data not shown). To achieve the originally described WHO-GHSS impact target of a 65% reduction in deaths by 2030,^9^ the annualised rate of change in mortality would need to be −10·7% (95% UI −11·4 to −9·9). To achieve the impact target of a 95% reduction in new cases by 2030, the annualised rate of change in seroprevalence would need to be −20·3% (−20·5 to −20·2). To achieve the absolute mortality rate target of less than four deaths per 100 000 globally by 2030, as put forth in the WHO Interim Guidance,^10^ the global annualised rate of change in mortality would have to be −5·3% (−6·5 to −4·1). In comparison, between 2015 and 2019, the annualised rate of change in mortality was −0·35% (−2·6 to 1·6) and the annualised rate of change in seroprevalence was −4·1% (−4·6 to −3·6).

## Discussion

Progress towards HBV elimination goals has been made, but hepatitis B remains a substantial public health problem. In 2019, the global prevalence of chronic HBV infection was 4·1%, representing 316 million people living with HBV. The all-age prevalence of chronic HBV infection has decreased across regions since the adoption of the SDGs in 2015 and the WHO-GHSS targets in 2016. Even more marked declines in prevalence, however, were seen between 1990 and 2019. 1990 is the first estimation year of GBD and just before the World Health Assembly recommended global infant vaccination against hepatitis B in 1992.^42^ This highlights the fact that progress can be made before formalisation of shared global goals, perhaps due to regional and local leadership, such as Taiwan (province of China) implementing the first universal hepatitis B vaccine programme in 1984,^43^ or the adoption of the first regional seroprevalence goal (for children) in the Western Pacific region in 2005.^44^

Unsurprisingly, declines in prevalence in children and infants were even more pronounced than in all ages during the same period. Prevalence in infants and children younger than 5 years declined by 77% between 1990 and 2019, to 1·0% globally. As of 2019, 147 countries had met or exceeded the WHO-GHSS 2020 interim proxy target of HBsAg prevalence less than or equal to 1% in children younger than 5 years, and 59 countries had already met or exceeded the 2030 proxy target of an HBsAg prevalence less than or equal to 0·1% in children younger than 5 years. The declines in HBV prevalence notably correspond with the scale-up of newborn and infant vaccination worldwide.^45^, ^46^ Globally, coverage of the three-dose series of HBV infant vaccination (HepB3) increased from 29% to 81% between 2000 and 2019.^29^ This scale-up is reflected in substantial declines in HBV prevalence in adolescents and young adults (aged 10–24 years) since 1990 as well. Nonetheless, disparities in HBsAg prevalence in this age group persisted, particularly in the African region, where 2·7% of infants and children younger than 5 years were HBsAg positive; additional progress is therefore still needed. Global coverage of the hepatitis B birth dose, in contrast to HepB3, is only 43%.^16^ There is still much regional and national variation in vaccine coverage.^29^ The Coalition for Global Hepatitis Elimination, US CDC, WHO, and other partners are assisting with the introduction and scale-up of hepatitis B vaccination of newborns in countries across Africa where chronic HBV prevalence is high and less than 10% of newborn babies receive timely hepatitis B vaccination.^47^

Hepatitis B vaccination remains a cost-effective, population-wide intervention to achieve elimination,^48^ prevent HBV transmission and occurrence of new cases of chronic HBV infection, and ultimately contribute to long-term reductions in mortality.^49^ It has previously been observed, however, that reliance on hepatitis B vaccination alone to decrease HBV-related mortality will require several decades, since progression from chronic hepatitis B infection to cirrhosis or liver cancer is slow.^50^, ^51^ This observation is consistent with our findings that reductions in the prevalence of chronic HBV infection outpace improvements in mortality. Since both 1990 and 2015, we found that absolute HBV-related deaths increased globally and across most regions. Indeed, only four countries met or surpassed the WHO-GHSS interim target of reducing HBV-related deaths by 10% by 2020. By contrast, declines were seen in all-age (crude) and age-standardised death rates. Comparing rates over time and location accounts for differences in population size, and comparing rates that have been age standardised with the GBD reference population removes confounding by population age structure. The decreases in mortality rates suggest that improvements in HBV-specific interventions and health systems overall have occurred, but continued rising death counts show that these improvements have been insufficient to overcome population growth and ageing. The decision to quantify hepatitis B elimination targets in terms of death counts, rather than death rates, means that a country's ability to meet elimination targets will depend heavily on its demographic trends. Furthermore, framing the target as a percentage reduction from a country-specific baseline might inappropriately equate small absolute improvements in low-burden countries with large absolute improvements in high-burden countries. Some of these potential limitations of the early WHO-GHSS 2016 targets were addressed when WHO issued its Interim Guidance for Country Validation of Viral Hepatitis Elimination,^10^ and provisionally expanded elimination targets to include achievement of an absolute all-age mortality rate of less than or equal to four deaths per 100 000 people per year. The fact that 68 countries already met this target as of 2019 re-emphasises the fact that the burden is uneven. This new target can help draw attention to countries most in need of support for elimination efforts, and flag which countries are likely to succeed if they undertake focused data collection for verifying elimination.

The findings of this study make it clear, however, that improvements must be accelerated to achieve either of the aforementioned HBV-related mortality targets: from relatively stable mortality between 2015 and 2019, to a downward annualised rate of change of 5–10% between 2020 and 2030. Countries can select the global elimination targets or individualised goals considered practical for their local epidemiological circumstances, factoring in health-systems capacity and population structures. Accelerating the pace of progress towards the selected goals for improvements in HBV-related mortality requires interventions that slow or prevent progression to serious sequelae for the approximately 316 million individuals who already have chronic HBV infection globally. Key interventions include improving access to existing diagnostics and suppressive antiviral medications,^52^ increased testing integrated into existing health systems, public health interventions to reduce alcohol exposure, access to medical and surgical treatment of cirrhosis and liver cancer, and development and deployment of functional cures for HBV infection.

For those already with chronic HBV infection, existing treatments are associated with a decreased risk of hepatocellular carcinoma, cirrhosis, and all-cause mortality,^12^, ^15^ but the proportion of people with chronic HBV infection being treated for HBV has yet to reach a scale to have an appreciable impact on trends in HBV-related mortality. Access to diagnostic and therapeutic services is scarce, particularly in resource-constrained settings. In 2016, only 10% of people with chronic HBV infection were diagnosed, and only 5% of those eligible for therapy had received antiviral treatment, well short of the WHO-GHSS 2030 target of 80% coverage of treatment for all those who are eligible.^9^, ^53^ In 2021, WHO estimated that only 30·4 million (24·3–38) people with chronic HBV infection were diagnosed and only 6·6 million (5·3–8·3) had received treatment.^54^ These services must be scaled up to achieve the full benefit of existing technology. Furthermore, although life-prolonging treatment does exist and is cost-effective in many settings,^55^, ^56^ there is no functional cure; patients must remain on suppressive therapies in the long term, which poses financial and sustainability barriers in some contexts.^57^ Coordinated partnerships, such as the International Coalition to Eliminate Hepatitis B Virus (ICE-HBV), facilitate global efforts towards the development of new HBV therapies and cures.^58^

Prevention and treatment initiatives make elimination feasible but require improved investment in the continuum of viral hepatitis services and broad health-systems strengthening.^11^ The geographical disparities in the burden of hepatitis B shown in this study highlight the importance of distributing and scaling up interventions in ways that are equitable and appropriate to the local context. Fortunately, many HBV-focused interventions can be integrated into existing services and, in turn, contribute to more widespread population health benefits. Integrated testing for HIV, syphilis, and HBV in antenatal care clinics, as recommended by the WHO Triple Elimination Initiative, can leverage existing infrastructure to eliminate mother-to-child transmission of multiple infectious diseases.^59^ The adoption of the hepatitis B birth dose vaccine can also be implemented within existing maternal and child health programmes. Uptake of the birth dose vaccination increases the likelihood that children will complete the infant vaccination series for protection against multiple diseases.^60^ Initiatives to strengthen blood transfusion and injection safety in health-care systems substantially reduce the risk of transmission of HBV and other bloodborne pathogens.^61^, ^62^

This study advances our understanding of the burden of hepatitis B in several important ways. In comparison with previous GBD publications,^3^, ^63^, ^64^, ^65^ we included more data on HBsAg prevalence, strengthened methods accounting for vaccination efforts, and improved garbage code redistribution methods for liver cancer, cirrhosis, and acute hepatitis. In comparison with estimates produced by the Center for Disease Analysis Foundation, WHO, and Schweitzer and colleagues,^24^ the estimates in GBD 2019 are embedded in a comprehensive framework encompassing 369 diseases and injuries, which allows policy makers to make informed decisions about how to prioritise HBV prevention, screening, and treatment within the context of the full burden of disease in a population. Schweitzer and colleagues^24^ and WHO^11^, ^54^ have large datasets informing estimation of HBsAg prevalence. The Center for Disease Analysis Foundation^23^ also uses a compartmental framework to generate estimates, including parameters on progression from HBsAg to downstream health states and horizontal and vertical transmission. Despite differences in data sources and methodologies, our HBsAg prevalence estimates are similar to those from other research groups,^4^ providing useful confirmation of estimates for stakeholders and policy makers (appendix table S6).

We estimated lower numbers of HBV-related deaths globally (555 000 [95% UI 487 000–630 000]) than the Centers for Disease Analysis Foundation (865 000) and WHO (820 000 [450 000–950 000]).^54^ Mortality estimates from WHO incorporate liver cancer mortality estimates from the Global Cancer Observatory (GLOBOCAN) and fractions of liver cancer attributable to HBV estimated by the International Agency for Research on Cancer (IARC). Thus, at least a proportion of the discrepancy between GBD and WHO estimates is due to our strategy of redistributing “malignant neoplasm of liver, not specified as primary or secondary” codes (ICD-10 C22.9) proportionately to both primary liver cancer and other primary cancers that metastasise to the liver, as C22.9 is mapped directly to liver cancer in GLOBOCAN data. An additional difference between WHO and GBD HBV-related mortality estimates is the proportion of the estimated liver cancer deaths assigned to HBV; WHO uses attributable fractions from IARC, whereas GBD estimates its own aetiological proportions. IARC's attributable fractions differ from GBD's aetiological proportions in both input data sources and approach. Whereas in GBD methodology, all liver cancer cases must be assigned to a single aetiology and aetiological proportions must sum to one, IARC attributable fractions recognise that multiple hepatic insults might interact, attributable fractions are based on the counterfactual removal of a single insult from a population, and the sum of attributable fractions for all aetiologies can exceed one.^66^, ^67^ This leads to unsurprising numerical differences in results. For example, in their published meta-analysis, for 2012, IARC estimated an attributable fraction for liver cancer due to HBV of 56% (95% CI 52–60) globally and 76% (95% CI 68–83) for China,^66^ whereas for the same year, GBD 2019 estimates for liver cancer were 40% globally and 65% for China. Similar differences in approach and numerical results are appreciated in IARC's preliminary results for estimation of the attributable fraction of cirrhosis.^68^ Our modelling strategy differs more substantially from that of the Center for Disease Analysis Foundation, so differences in results merit further investigation. Differences in point estimates of HBV-related deaths belie some degree of similarity, as uncertainty intervals for these estimates overlap. Discrepancies in some estimates, and the substantial uncertainty in all, should promote discourse about data inputs and methodological considerations, give rise to improved estimates, and guide future data collection to better understand disease burden.

We acknowledge several limitations of this study. First, there is data sparsity in several of the models, particularly the cirrhosis and liver cancer aetiological proportion models. For the years and locations for which data are not available, our estimates depend on predictive covariates, regional levels, and consistent temporal trends to generate estimates. We need to enhance data-seeking efforts, support data sharing with other modelling institutions, and promote primary data collection when possible. For example, a collaborative study between the European Centre for Disease Control and the European Association for the Study of the Liver piloted a WHO-developed protocol to support countries in collecting aetiological proportion data for cirrhosis and liver cancer at sentinel sites.^69^ Extending such efforts to additional sites can improve estimation of burden, particularly in low-income countries that are estimated to have a high burden with minimal or no data sources, such as Guinea, Guinea-Bissau, and Chad.

Second, there are additional covariates that could be used to refine estimates of HBsAg seroprevalence, aetiological proportions of cirrhosis and liver cancer, or mortality due to HBV-related diseases (acute hepatitis, cirrhosis, and liver cancer) where primary data for these quantities are sparse. Our suite of covariates is extensive but does not include coverage of suppressive antiviral therapy or prevalence of hepatitis delta virus (HDV) infection among those infected with HBV. These two factors are known to slow^52^ and accelerate^70^ progression from chronic infection to advanced liver disease and death. A systematic review and meta-analysis by Stockdale and colleagues^70^ reported the prevalence of HDV in general HBsAg seroprevalent populations ranging from less than 1% to almost 37%, and accounting for this variation in prevalence of co-infection might improve predictions of mortality for some locations. Furthermore, Stockdale and colleagues'^70^ provisional estimates of the population attributable fraction of cirrhosis (approximately 18%) and hepatocellular carcinoma (approximately 20%) in individuals with chronic HBV infection suggest that almost 98 000 HBV-related deaths in 2019 might have been attributable to HDV infection. A formal estimate of the HDV-attributable burden, alongside other risk factors for progression (such as alcohol or other viral co-infections) would provide further insight into opportunities for intervention.

Third, our modelling strategy relied on a post-hoc adjustment to incorporate vaccination trends more effectively because of limitations in DisMod-MR 2.1. DisMod fits steady-state compartmental models at 2-year to 5-year intervals, and results are later interpolated. HBsAg seroprevalence, however, has changed rapidly over time with the introduction of highly effective interventions. DisMod's serial, steady-state modelling approach does not faithfully follow data with such rapid temporal changes and strong cohort effects. The new approach of modelling a counterfactual seroprevalence and correcting for vaccination produces estimates that are more compatible with contemporary data, but this comes at the expense of not leveraging data from more recent sero-surveys. Additionally, the vaccination coverage used in the post-hoc adjustment is the hepatitis B three-dose primary series (with assumed 95% efficacy). This adjustment is applied only to the proportion of the population fully vaccinated in infancy, and therefore does not reflect any indirect benefit to unvaccinated individuals in the same cohort (via herd immunity), protective effects of partial vaccination, or vaccination of adults; these limitations might lead to over-estimation. Indeed, a previous study by Wiesen and colleagues^71^ estimated HBsAg seroprevalence in children by use of a natural history model that also did not account for these effects, and found their estimates exceeded measurements from serosurveys done in vaccinated cohorts in Kiribati and Papua New Guinea.^72^ The GBD adjustment also does not explicitly take into account birth-dose coverage, the most important intervention in preventing mother-to-child transmission.^73^, ^74^ Future rounds of GBD should use methods that account for these effects and explicitly incorporate data from vaccinated cohorts.

Fourth, the aetiological proportions for cirrhosis and liver cancer from case-series are used to divide both prevalence and mortality parent estimates. This implicitly assumes the rate of progression from compensated cirrhosis to decompensated cirrhosis to death is similar across all aetiologies of cirrhosis. The limited longitudinal studies available to date suggest this is not true, but it is unclear how large an impact differences in rates of progression across aetiologies would have on estimation.^75^, ^76^

In conclusion, this study identifies progress and barriers to achievement of the WHO-GHSS elimination goals and the SDG target for viral hepatitis. The prevalence of chronic HBV infection has declined in much of the world over the past several decades, as have mortality rates due to HBV-related diseases, but targeted reductions in death counts have yet to be achieved, due to population growth and ageing. There is an unequal distribution of the burden of HBV-related diseases due to differences in transmission modes and access to vaccination, screening, and treatment. Interventions along the hepatitis service continuum must be implemented to decrease hepatitis burden. With less than 10 years to go until the deadline for the elimination goals, efforts must be intensified to ensure the elimination goals for hepatitis B are achieved.

For the **GBD protocol** see http://www.healthdata.org/gbd/about/protocol

## Data sharing

To download the data used in these analyses, please visit the Global Health Data Exchange GBD 2019 website.

## Declaration of interests

S Afzal reports participation on a data safety monitoring board or advisory board on the Corona Expert Advisory Group and Infectious Diseases Expert Advisory Group; leadership or fiduciary roles in other board, society, committee or advocacy groups, paid or unpaid, as a Fellow of the Faculty of Public Health (UK), and as the Dean of Public Health and Preventive Medicine, and the Chairperson of Medicine at the King Edward Medical University (Lahore, Pakistan); all outside the submitted work. R Ancuceanu reports consulting fees from AbbVie; payment or honoraria for lectures, presentations, speakers' bureau, manuscript writing, or educational events from from AbbVie, Sandoz, and B Braun; all outside the submitted work. M Ausloos reports grants from Romanian National Authority for Scientific Research and Innovation (CNDS-UEFISCDI; project number PN-III-P4-ID-PCCF-2016-0084 Oct 2018–Sep 2022), outside the submitted work. X Dai reports support for the present manuscript from the University of Washington (Seattle, WA, USA), through their employment at IHME. T M Drake reports grant funding from Aligod Therapeutics for research into primary liver cancer, outside the submitted work. C Herteliu reports grants from CNDS-UEFISCDI (project numbers PN-III-P4-ID-PCCF-2016-0084 Oct 2018–Sept 2022 and PN-III-P2–2.1-SOL-2020-2-0351 June 2020–Oct 2020), from the Romanian Ministry of Research Innovation and Digitalization (project number ID-585-CTR-42-PFE-2021 Jan 2022–June 2023), and from the Ministry of Labour and Social Justice, Romania (project number 30/PSCD/2018 Sept 2018–June 2019), outside the submitted work. L Hiebert reports grants or contracts through the Task Force for Global Health, which receives funds for the general support of the Coalition for Global Hepatitis Elimination from Abbott, Gilead, AbbVie, Merck, Siemens, Cepheid, Roche, Pharco, Zydus-Cadila, and governmental agencies and philanthropic organisations; outside the submitted work. N E Ismail reports leadership or fiduciary roles in other board, society, committee or advocacy groups, unpaid, as a council member of the Malaysian Academy of Pharmacy, outside the submitted work. I M Karaye reports support for attending meetings or travel, or both, from Hofstra University for the Natural Hazards meeting and American College of Epidemiology Conference, outside the submitted work. J V Lazarus reports grants and consulting fees from AbbVie, Gilead Sciences, and MSD; payment or honoraria for lectures, presentations, speakers bureaus, manuscript writing or educational events from AbbVie, Gilead, Sciences, Intercept, Jannsen, and MSD; leadership or fiduciary roles in other board, society, committee or advocacy groups, unpaid, with the EASL international Liver Foundation; all outside the submitted work. J A Loureiro reports support for the present manuscript from Fundaçã o para a Ciência e Técnologia (FCT) under the Scientific Employment Stimulus (CEECINST/00049/2018). P C Matthews reports support for the present manuscript from the Welcome Trust through the Intermediate Clinical Fellowship (grant Ref 110110/Z/15/Z); grants or contracts from GlaxoSmithKline as a contribution to their PhD stipend; and royalties from Oxford University Press for the publication of a medical textbook; all outside the submitted work. O O Odukoya reports support for the present manuscript from the Fogarty International Center of the National Institutes of Health under the Award Number K43TW010704. M J Postma reports stock or stock options in HealthEcore and PAG, outside the submitted work. A Pana reports grants from CNDS-UEFISCDI (project numbers PN-III-P4-ID-PCCF-2016–0084 Oct 2018–Sep 2022 and PN-III-P2–2·1-SOL-2020–2-0351 June 2020–Oct 2020), outside the submitted work. J Sanabria reports grants for contracts from Marshall University School of Medicine and Joan Edwards Comprehensive Cancer Center (Huntington, WV, USA); patents planned, issued, or pending for pNaKtide for the treatment of hepatocellular carcinoma related to NASH and NASH; participation on a data safety monitoring board or advisory board with the Department of Surgery, Marshall University, as a quality assessment and assurance officer; leadership or fiduciary roles in other board, society, committee or advocacy groups, and unpaid roles with several national and international surgical societies; all outside the submitted work. J A Singh reports consulting fees from Crealta/Horizon, Medisys, Fidia, PK Med, Two labs, Adept Field Solutions, Clinical Care options, Clearview Healthcare Partners, Putnam Associates, Focus Forward, Navigant Consulting, Spherix, MedIQ, Jupiter Life, UBM, Trio Health, Medscape, WebMD, Practice Point Communications, the US National Institutes of Health, and the American College of Rheumatology; payment or honoraria for participating in the speakers bureau for Simply Speaking; support for attending meetings or travel, or both, from the steering committee of OMERACT, to attend their meeting every 2 years; participation on a data safety monitoring board or advisory board as an unpaid member of the US Food and Drug Administration (FDA) Arthritis Advisory Committee; leadership or fiduciary roles in other board, society, committee or advocacy groups, paid or unpaid, as a member of the steering committee of OMERACT, an international organisation that develops measures for clinical trials and receives arms' length funding from 12 pharmaceutical companies, with the Veterans Affairs Rheumatology Field Advisory Committee as Chair, and with the UAB Cochrane Musculoskeletal Group Satellite Center on Network Meta-analysis as a director and editor; stock or stock options in TPT Global Tech, Vaxart Pharmaceuticals, Atyu Biopharma, Adaptimmune Therapeutics, GeoVax Labs, Pieris Pharmaceuticals, Enzolytics, Series Therapeutics, Tonix Pharmaceuticals, and Charlotte's Web Holdings; and previously owned stock options in Amarin, Viking, and Moderna; all outside the submitted work. J W Ward reports grants or contracts through The Task Force for Global Health, which receives funds for the general support of the Coalition for Global Hepatitis Elimination from Abbott, Gilead, AbbVie, Merck, Siemens, Cepheid, Roche, Pharco, Zydus-Cadila, governmental agencies and philanthropic organisations; membership on an advisory board, unpaid, with the international Coalition to Eliminate HBV, and membership on an advisory committee, unpaid, for the Longevity Project for HCV at the University of Liverpool (UK); all outside the submitted work.
