# Supplementary material for: Global, regional, and national burden of hepatitis B, 1990–2019: a systematic analysis for the Global Burden of Disease Study 2019
Source: Lancet Gastroenterol Hepatol. 2022 Jun 21;7(9):796–829. doi: 10.1016/S2468-1253(22)00124-8 (PMC9349325; doi:10.1016/S2468-1253(22)00124-8)
Supplement: Supplementary appendix [file mmc1.pdf]

THE LANCET
Gastroenterology & Hepatology

Supplementary appendix

This appendix formed part of the original submission and has been peer reviewed. We post it as supplied by the authors.

Supplement to: GBD 2019 Hepatitis B Collaborators. Global, regional, and national burden of hepatitis B, 1990–2019: a systematic analysis for the Global Burden of Disease Study 2019. *Lancet Gastroenterol Hepatol* 2022; published online June 20. https://doi.org/10.1016/S2468-1253(22)00124-8.

1 Table of Contents

2 Statement of GATHER compliance 4
3 HBsAg estimation 5
3.1 Flowchart 5
3.2 HBsAg data 6
3.3 Exclude seroprevalence data exposed to vaccination 9
3.4 Cause-specific mortality rate inputs 9
3.5 Data adjustment 10
3.6 Modelling counterfactual HBsAg seroprevalence 11
3.7 Post-hoc adjustment 13
3.8 Disability weights 14
4 Cirrhosis and other chronic liver diseases non-fatal estimation 15
4.1 Flowchart 16
4.2 Aetologic proportion data 17
4.3 Clinical administrative data 19
4.4 Cause-specific mortality rate inputs 23
4.5 Excess mortality rate inputs 23
4.6 Aetiological proportion data adjustment 25
4.7 Clinical informatics data adjustment 26
4.8 Modelling total cirrhosis and other chronic liver diseases non-fatal 28
4.9 Modelling decompensated cirrhosis and other chronic liver diseases non-fatal 29
4.10 Modelling proportion of cirrhosis due to hepatitis B 30
4.11 Modelling compensated cirrhosis and other chronic liver diseases 31
4.12 Modelling aetiology-specific estimates 31
4.13 Disability weights 32
5 Cirrhosis and other chronic liver diseases fatal estimation 34
5.1 Flowchart 34
5.2 Data identification and processing 35
5.3 Modelling cirrhosis and other chronic liver diseases mortality 38
5.4 Modelling aetiology-specific estimates 39
6 Liver cancer fatal estimation 40
6.1 Flowchart 40

6.2 Data identification and processing 41
6.3 Modelling liver cancer mortality 47
6.4 Modelling aetiology-specific estimates 48
7 Liver cancer non-fatal estimation 49
7.1 Flowchart 49
7.2 Modelling liver cancer morbidity 50
7.3 Aetiological proportion data 51
7.4 Modelling proportion of liver cancer due to hepatitis B 54
7.5 Modelling aetiology-specific estimates 54
7.6 Disability weights 54
8 Acute hepatitis B non-fatal estimation 56
8.1 Flowchart 56
8.2 Modelling acute hepatitis B morbidity 56
8.3 Disability weights 58
9 Acute hepatitis B fatal estimation 59
9.1 Flowchart 59
9.2 Data identification and processing 60
9.3 Modelling acute hepatitis parent mortality 63
9.4 Modelling acute hepatitis B mortality 63
10 Hepatitis B 3-dose primary series vaccine coverage estimation 65
10.1 HepB3 vaccine data inputs 65
10.2 Modelling HepB3 vaccine coverage 65
11 Additional analyses 66
11.1 Target achievement 66
11.2 Annualised rate of change 66
12 Summary of changes from GBD 2017 67
12.1 HBsAg changes 67
12.2 Cirrhosis changes 67
12.3 Liver cancer changes 68
12.4 Acute hepatitis B changes 68
13 Geographical hierarchies 69
14 GATHER checklist 74
15 Supplementary tables and figures 76

Supplemental Table 1: HBsAg prevalence data sources a) for counterfactual model and b) for vetting of final estimates 77

Supplemental Table 2: Percentage change in HBsAg prevalence (%) and prevalence counts in all ages and under 5 years between 1990 and 2019 and 2015 and 2019, by location 133

Supplemental Table 3. Percentage change in HBV-related death rates and HBV-related death counts in all ages between 1990 and 2019 and 2015 and 2019, by location 144

Supplemental Table 4. Achievement in 2019 of WHO-GHSS 2020 death impact target, WHO-GHSS new cases impact target, and under-5 prevalence proxy target, with high probability, by location. High probability is defined by at least 950 out of 1000 draws meeting target. 154

Supplementary Table 5. Achievement in 2019 of WHO Interim Guidance's absolute mortality rate target and WHO-GHSS under-5 prevalence target for 2030, with high probability, by location. High probability is defined by at least 950 out of 1000 draws. 155

Supplementary Table 6. Prevalence of HBsAg, all ages, both sexes, as reported by GBD and multiple other estimation groups 156

Supplemental Figure 1. Hepatitis-related deaths by virus and cause, global, 2019 157

Supplemental Figure 2. Percentage of HBV-related disability-adjusted life-years (DALYs) in top burden countries (out of total HBV-related DALYs) 158

Supplemental Figure 3. Map of percentage change in all-age HBV-related death counts (A) and all-age HBV death rates (B) between 2015 and 2019 by country. Percentage change category was achieved with high probability (at least 950 out of 1000 draws of percentage change). 159

Supplemental Figure 4. Map of percentage change in under-5 HBsAg prevalence counts (A) and under-5 HBsAg seroprevalence (%) (B) between 2015 and 2019, by country. Percentage change category was achieved with high probability (at least 950 out of 1000 draws of percentage change). 160

Supplemental Figure 5. HBV-related death counts by WHO region by cause 161

Supplemental Figure 6. Age-sex-specific death counts of HBV-related diseases in 2019, globally and by WHO region 162

Supplemental Figure 7. Age-sex-specific death rates per 100,000 of HBV-related diseases in 2019, globally and by WHO region 163

16 Authorship Contributions 164

17 Supplementary References 169

2 Statement of GATHER compliance

This study was developed in accordance with the Guidelines for Accurate and Transparent Health Estimates Reporting (GATHER) guidelines.[1] Websites cited in this appendix were last accessed January 9, 2022, unless otherwise stated.

3 HBsAg estimation

3.1 Flowchart

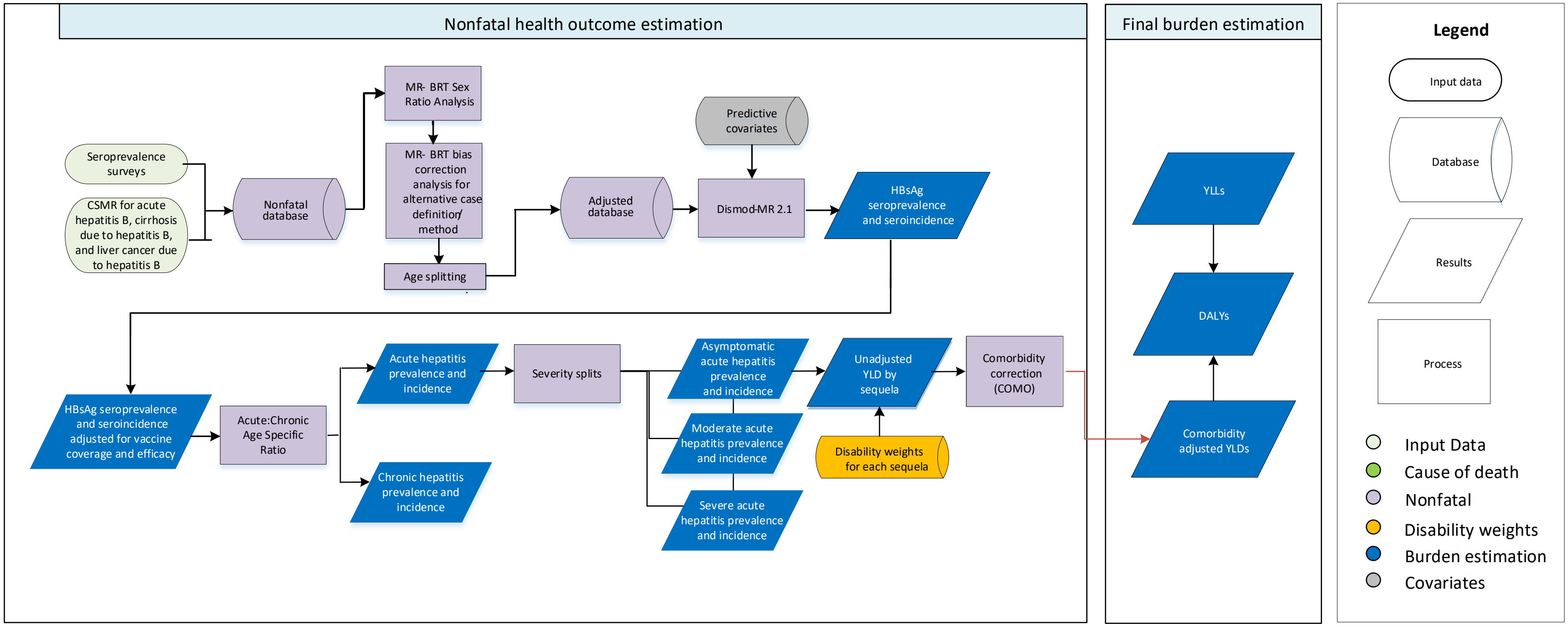

Figure 1: Flowchart GBD HBsAg and acute hepatitis B incidence, prevalence, and YLD estimation

3.2 HBsAg data

Systematic literature reviews were completed in GBD 2013[2] using the search string below.

PubMed search terms: ((HBsAg[Title/Abstract] AND seroprevalence[Title/Abstract]) AND ("2009"[Date – Publication]: "2013"[Date – Publication]))

Sources were included if they measured HBsAg prevalence; studies of hepatitis B core antigens or surface antibodies in the absence of surface antigens were not included. Studies were included if they sampled from the general population, or a subpopulations (blood donors and pregnant women) for which data could be adjusted for bias. Subpopulations that were excluded included sex workers andpopulations defined by the presence of another disease (for example, studies of HBsAg seroprevalence conducted exclusively in HIV-positive samples).

In lieu of updating the GBD 2013 systematic review, and in response to a hepatitis elimination global data collaborative meeting organized by the WHO – Global Hepatitis Programme and held in Seattle, United States, in May 2019, we worked to align GBD HBsAg data sources with those reported in the later systematic review by Schweitzer et al[3]. GBD researchers reviewed sources from Schweitzer et al according to the inclusion and exclusion criteria of our previous internal systematic review from GBD 2013. Given the length of the citation list, we prioritised data from certain time-periods and geographies, like data-sparse regions of sub-Saharan Africa, Australasia, Andean South America, Eastern Europe, and high-income North America. We identified and added 130 new sources prior to the GBD 2019 deadline for new data inclusion. Figure 2 shows the number of sources from Schweitzer et al. that were included and excluded for GBD 2019 HBsAg modelling as new data sources. We will continue review and harmonization of data sources for future rounds of GBD.

Data sources listed in Supplemental Table 1 can be viewed as extracted ("Unadjusted") and as adjusted per section 3.5 ("Adjusted"), with excluded sources marked in red, on the GBD Epi Visualization for the HBsAg Seroprevalence – counterfactual model

Figure 2: Flowsheet of sources cited by Schweitzer et al used in GBD modelling

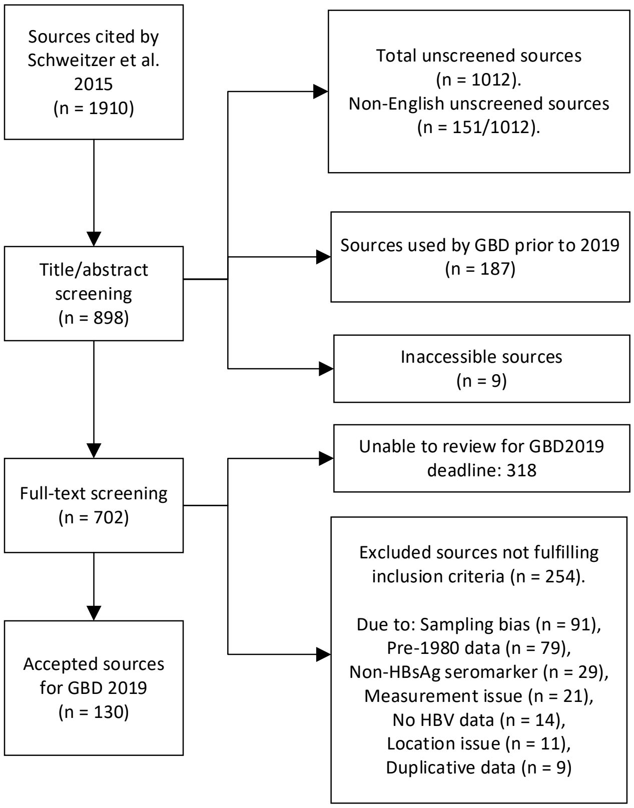

Figure 3: Data inputs for HBsAg prevalence models

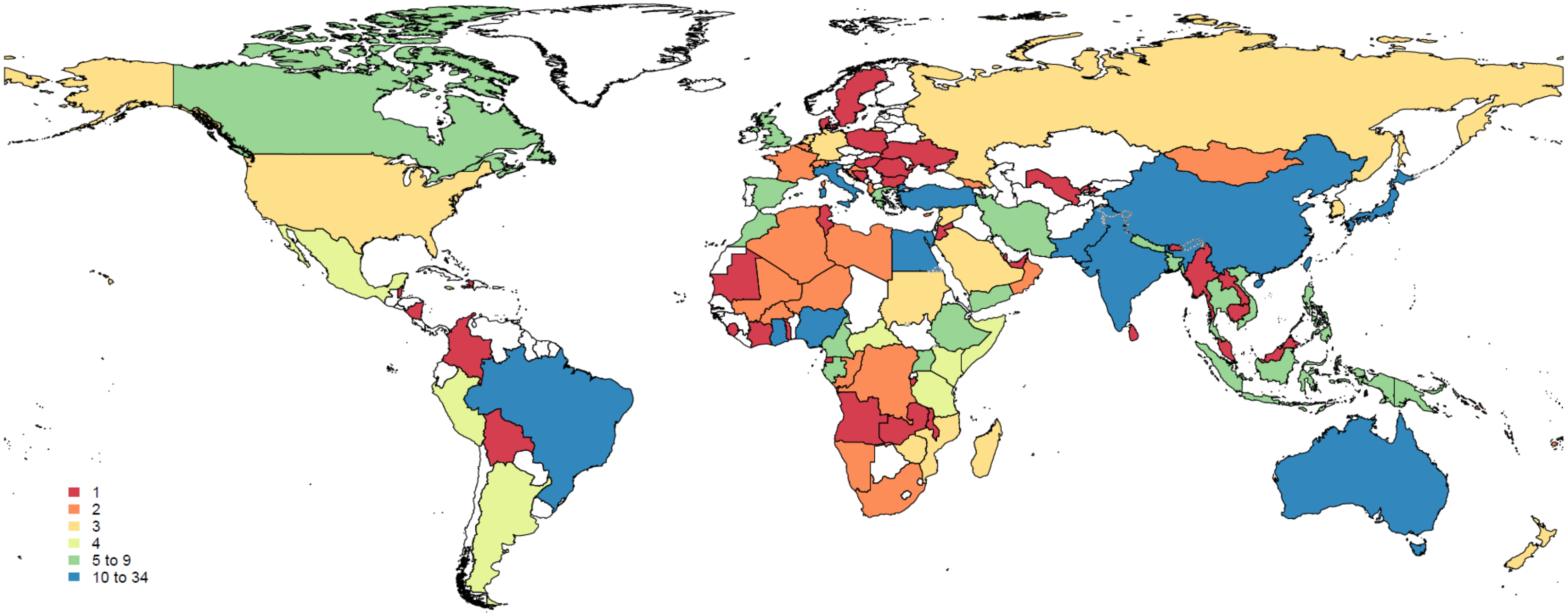

3.3 Exclude seroprevalence data exposed to vaccination

In previous iterations of the GBD[4–7], the DisMod-MR 2.1[8] model of HBsAg seroprevalence using all available data tended to follow the data from unvaccinated populations and poorly fit prevalence data from vaccinated populations at younger ages. DisMod-MR 2.1 separately fits steady-state compartmental models for specific years (1990, 1995, 2000, 2005, 2010, 2015, 2017, 2019), which are then interpolated to provide annual results. The data used to fit each of these models are those input data in the full input data-set that fall within a time-window specified by the modeler; in previous iterations of GBD, time-windows as narrow as 2 years were tried, but the model still did not capture the rapid, age-specific changes in HBsAg seroprevalence seen in the input data over the years in which HepB3 vaccine was introduced in infants. In GBD 2019, we changed the modelling strategy to a counterfactual model to estimate what seroprevalence would be in the absence of vaccination efforts. We excluded seroprevalence data from age groups and years that would have been affected by universal infant vaccination programs. We utilized only data from age group – year combinations that would not have been affected by these programs to model a counterfactual scenario in DisMod-MR 2.1 (as described below in 3.6). Studies excluded from the data-set for modeling the counterfactual were used to compare to final prevalence estimates produced by post hoc adjustment of the counterfactual model (as described in 3.7).

Data exclusion was determined by looking at the first and last year of the study and the oldest and youngest ages of study participants to calculate the earliest and latest possible years of birth of the sample participants. The year of birth was then compared to the year of introduction of the hepatitis B 3-dose vaccine for a given location.

- If all birth years in a study were before the year of vaccine introduction, the data were included.
- If all birth years in a study were after the year of vaccine introduction, the data were excluded.
- If the year of vaccine introduction fell between the earliest and latest year of birth values, then we assumed a normal distribution of year of births. If greater than 50% of the study population years of birth fell after the year of vaccine introduction, then we excluded data related to the source. If less than 50% of the birth years fell after the year of vaccine introduction, then the data source was included.

3.4 Cause-specific mortality rate inputs

We included inputs on cause-specific mortality rate (CSMR) to estimate prevalence in DisMod given compartmental structure of the modelling tool. Utilisation of non-prevalence data helps solve differential equations from the compartmental model, contributing to internal consistency of different parameters by providing information from fatal estimation processes into non-fatal estimation processes.[8,9] We summed the GBD-estimated CSMR of acute hepatitis B, cirrhosis due to hepatitis B, and liver cancer due to hepatitis B and used these aggregated estimates as an additional input to our counterfactual HBsAg DisMod model.

3.5 Data adjustment

Prior to fitting the counterfactual HBsAg DisMod model, we performed several data adjustments (to input data shown in Table 1a) to correct for non-"ideal" data, including "sex-splitting", "crosswalking", and "age-splitting".

First, we adjusted HBsAg seroprevalence data that reported prevalence in both sexes combined to produce sex-specific model inputs. To estimate the sex-specific adjustment factors, we reviewed the data-set described in 3.2, identified those studies that reported prevalence separately by sex, and calculated the log ratio of female to male prevalence for each study. The standard error of each log ratio was calculated from the standard errors of the individual male and female measurements using the delta method. We then modelled these log ratios using meta-regression—Bayesian, regularised, trimmed (MR-BRT), a tool developed at IHME for bias adjustment and previously described.[9,10] In brief, MR-BRT is a customized application of the open source package LimeTr, which fits Bayesian mixed effects models allowing nonlinear fixed effects, priors and constraints, while requiring random effects to enter the model in a linear way, and solves the marginal likelihood for these models using a least trimmed squares estimator. Estimated parameters include the coefficients for fixed effects (betas), their variances (lambdas), and the variance of the vector of random effects (gamma); when random effects are estimated for each study in the meta-regression, the latter serves as a measure of inter-study heterogeneity; uncertainty intervals for effects estimated for MR-BRT in our analyses include this gamma variance and thus reflect between-study heterogeneity. Priors for all effects estimated in MR-BRT models in this report were uninformative unless otherwise stated below. In the case of our log sex-ratio for HBsAg seroprevalence, we included a single intercept term with an uninformative prior, random effects for each serosurvey, and 10% trimming. Adjustment factors specific to super-regions or regions will be explored as additional matched data pairs become available in future iterations of GBD.

Table 1 shows the estimated sex ratio calculated using MR-BRT.

Table 1: MR-BRT sex ratios

Data input	Gamma	Beta coefficient, log<br><br>(95% CI)	Exponentiated beta coefficient (95% CI)*
HBsAg	0.018	–0.33 (–0.36 to –0.31)	0.71 (0.70 to 0.73)

*Ie, estimated ratio of female to male seroprevalence

We then used the modelled sex ratio to adjust "both"-sex data values to expected "male" and "female" values. We calculated the male values as $val_{male} = val_{both} * \frac{pop_{both}}{(pop_{male}+ratio*pop_{female})}$. We calculated female values $val_{female} = ratio * val_{male}$.

Next, we performed "crosswalks", or bias adjustments, to adjust seroprevalence measurements made in samples of blood donors and pregnant women to be more comparable to general population data. We identified pairs of studies of the same year, age, sex, and location that were measured in reference (general) vs alternative (pregnant or blood donor) populations. We calculated the logit difference for each matched pair as $logit(alternative) - logit(reference)$, calculated the standard error for each logit-transformed prevalence using the delta method, and calculated the standard error for the difference as $\sqrt{(variance\ of\ logit(alternative)) + (variance\ of\ logit(reference))}$. We used MR-BRT (as briefly described on page 10 and previously reported[9,10]) to estimate the weighted average of the logit differences; this model was conducted as a network analysis, with a single, pooled effect for each alternative study population type, uninformative priors, 10% trimming and random effects on unique pairs of matched studies. We then adjusted the mean of the non-reference data-points using the pooled estimate for the relevant effect and recalculated the uncertainty in the non-reference data-points (again using the delta method) to incorporate the uncertainty of the adjustment.

$$adjusted_{estimate} = inverse.logit((logit(alternative)) - (pooled\ logit\ difference))$$

Table 2 reports on estimated crosswalk coefficients.

Table 2: MR-BRT crosswalk factors for HBsAg seroprevalence non-representative populations

Data input	Reference or alternative case definition	Gamma	Beta coefficient, logit* (95% CI)
General population	Ref	0.72	---
Blood donors	Alt		–0.53 (–1.94 to 0.81)
Pregnant women	Alt		–0.86 (–2.44 to 0.65)

* A beta coefficient of zero in logit space can be interpreted as no meaningful difference between the reference and alternative definitions. A negative logit beta coefficient indicates that the alternative definition data are systematically lower than the reference definition data and will be adjusted up, whereas a positive value indicates the alternative data are systematically higher and will be adjusted down.

Lastly, we split data reported for groups with an age-range greater than 25 years to produce measurements for 5-year age bins. We assumed the age-distribution in the study sample was the same as the estimated population in GBD 2017.[7] We also assumed that the ratios of age-specific prevalence to full age-prevalence were the same as in the seroprevalence model from GBD 2017.

3.6 Modelling counterfactual HBsAg seroprevalence

We estimated HBsAg seroprevalence using DisMod-MR 2.1, a Bayesian meta-regression tool developed for GBD and previously described.[8,9] In brief, DisMod is designed as a geographical cascade where a first model is run on all the world's data, which produces an initial global fit and

estimates coefficients for predictor variables. The global fit adjusted by the values of random effects for each of seven GBD super-regions are passed down as Bayesian priors to models for each super-region, which are fit using the input data for that super-region only. The same steps are repeated going from 7 super-region fits to 21 region fits and then to 204 fits for individual countries and territories. Where applicable, country-level fits are used to generate priors for a round of models for subnational units. Below the global fit, all models are fit separately by sex and for eight years: 1990, 1995, 2000, 2005, 2010, 2015, 2017, and 2019. DisMod allows for the above-described cascade to be applied to a model of a single parameter (as for the models described in sections 4.10 and 7.4, below), but where data for multiple interrelated parameters are available, as is the case for HBsAg, the cascade is applied to multiple regressions in a compartmental model. Thus, for each model in the cascade, the system of differential equations connecting prevalence, incidence, remission, and mortality is solved using inputs for multiple epidemiologic quantities, assuming a steady state and producing estimates for all measures that are internally consistent. Solutions are obtained using a Markov chain Monte Carlo approach with a default of 5000 iterations, and the last 500 iterations are broken into four groups of 125 iterations, and summary statistics for these four groups are compared to assess convergence of the model for all quantities of interest.[8,9]

In the counterfactual HBsAg seroprevalence DisMod model, we used the processed inputs described in sections 3.2-3.5 to generate estimates for all year-age-sex-location combinations in the GBD framework. In addition to HBsAg seroprevalence and CSMR inputs, we included predictive covariates in the model to improve estimation in quantities of interest where HBsAg seroprevalence data are absent or scarce. We included remission priors between 0 and 0.02, excess mortality priors between 0 and 0.1, and incidence priors between 0 and 0.05 for all ages. The summary of covariates used in the counterfactual HBsAg seroprevalence DisMod-MR 2.1 model are listed in Table 3.

Table 3: Covariates used in DisMod-MR 2.1 model of counterfactual HBsAg seroprevalence

Covariate	Parameter	Exponentiated beta (95% UI)
Log-transformed age-standardised SEV scalar: Hep B	Prevalence	1.13 (1.00–1.43)
Socio-demographic Index (SDI)	Prevalence	0.14 (0.14–0.14)
Healthcare Access and Quality Index (HAQi)	Excess mortality rate	1.00 (1.00–1.00)

The data inputs and modeling approaches used to estimate summary exposure values (SEVs),[11] Socio-demographic Index (SDI)[12] and Healthcare Access and Quality Index (HAQi) [9,13] for GBD have been previously described. These processes result in estimates of these covariates for every year-age-sex-location combination in the GBD framework, and they are updated with each round of GBD.

3.7 Post-hoc adjustment

As described in section 3.3, above, previous attempts to fit all available HBsAg seroprevalence data in a steady-state DisMod multi-regression model poorly captured age-specific rapid temporal changes, which motivated fitting the counterfactual model of HBsAg seroprevalence in the absence of vaccination, as described in sections 3.4-3.6. After the completion of the counterfactual DisMod model described in section 3.6, a post-hoc adjustment was performed to modify the counterfactual (no vaccination) estimates of HBsAg seroprevalence toward the HBsAg seroprevalence expected based on HepB3 coverage and efficacy.

First, we multiplied counterfactual HBsAg seroprevalence estimates by vaccine coverage and efficacy to estimate the proportion of counterfactual cases averted due to vaccination efforts. We employed the GBD-produced location-year-specific hepatitis B three-dose primary vaccine series coverage (HepB3)[14]. A vaccine efficacy of 95% was assumed based on expert input from members of the GBD Collaborator Network and the participants in the May 2019 hepatitis elimination global data collaborative meeting (per above); this is within the range of previous reports of effectiveness in preventing chronic infection[15–18]. Then, the number of cases averted were subtracted from the number of counterfactual HBsAg seroprevalent cases to get the final estimates of HBsAg seroprevalent cases. The number of cases was divided by population to produce HBsAg seroprevalence estimates.

$$final\ HBsAg_{yasl} = counterfactual\ HBsAg_{yasl} - \left(counterfactual\ HBsAg_{yasl} * proportion\ of\ HepB3\ vaccine\ coverage_{yasl} * \ 0.95\right) ;$$

where yasl is the year-age-sex-location-specific value.

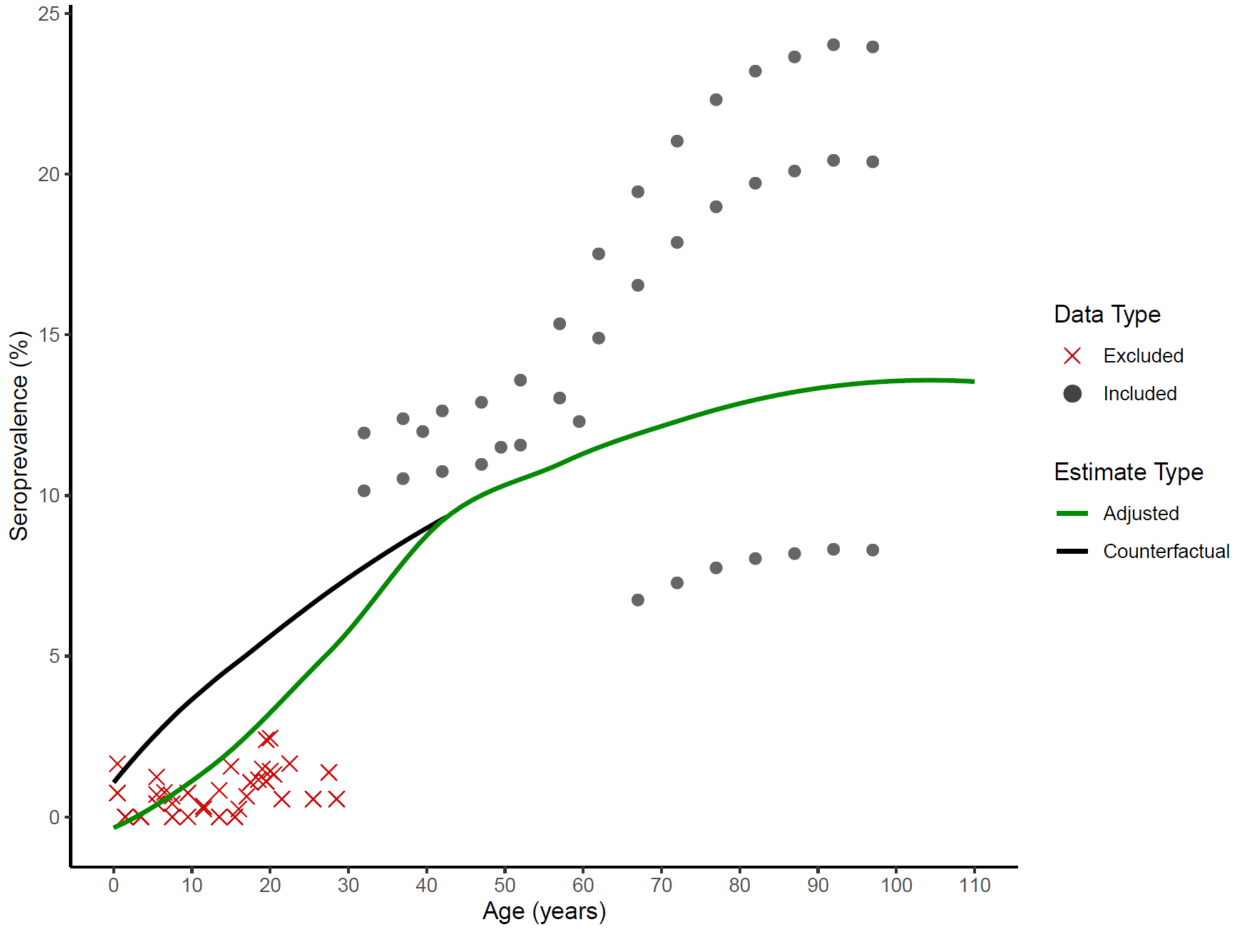

Figure 4: Counterfactual (black line) and HepB3 vaccine-adjusted (green line) HBsAg estimates in comparison to included (grey dots) and excluded (red crosses) datapoints

The final estimates produced by this post-hoc adjustment can be viewed in the IHME Viz Hub as the Seroprevalence of HBsAg – adjusted for hepatitis B 3 dose vaccine coverage model.

3.8 Disability weights

GBD does not assign disability to chronic HBV infection in the absence of cirrhosis or liver cancer.[19,20]

4 Cirrhosis and other chronic liver diseases non-fatal estimation

Cirrhosis is a chronic liver disease in which there is progressive destruction of functional hepatic cells and replacement with fibrosis (scarring) of the liver. It is often caused by alcohol use, chronic infection with hepatitis B or C, or non-alcoholic steatohepatitis, but there is also a residual category of multiple other causes. Early disease is typically asymptomatic as the liver's resilience compensates for cirrhotic damage. Decompensated cirrhosis occurs when the disease progresses beyond the capacity of the liver to compensate for the damage, and is marked by profound symptoms and health loss and typically progresses to death in a few years.

4.1 Flowchart

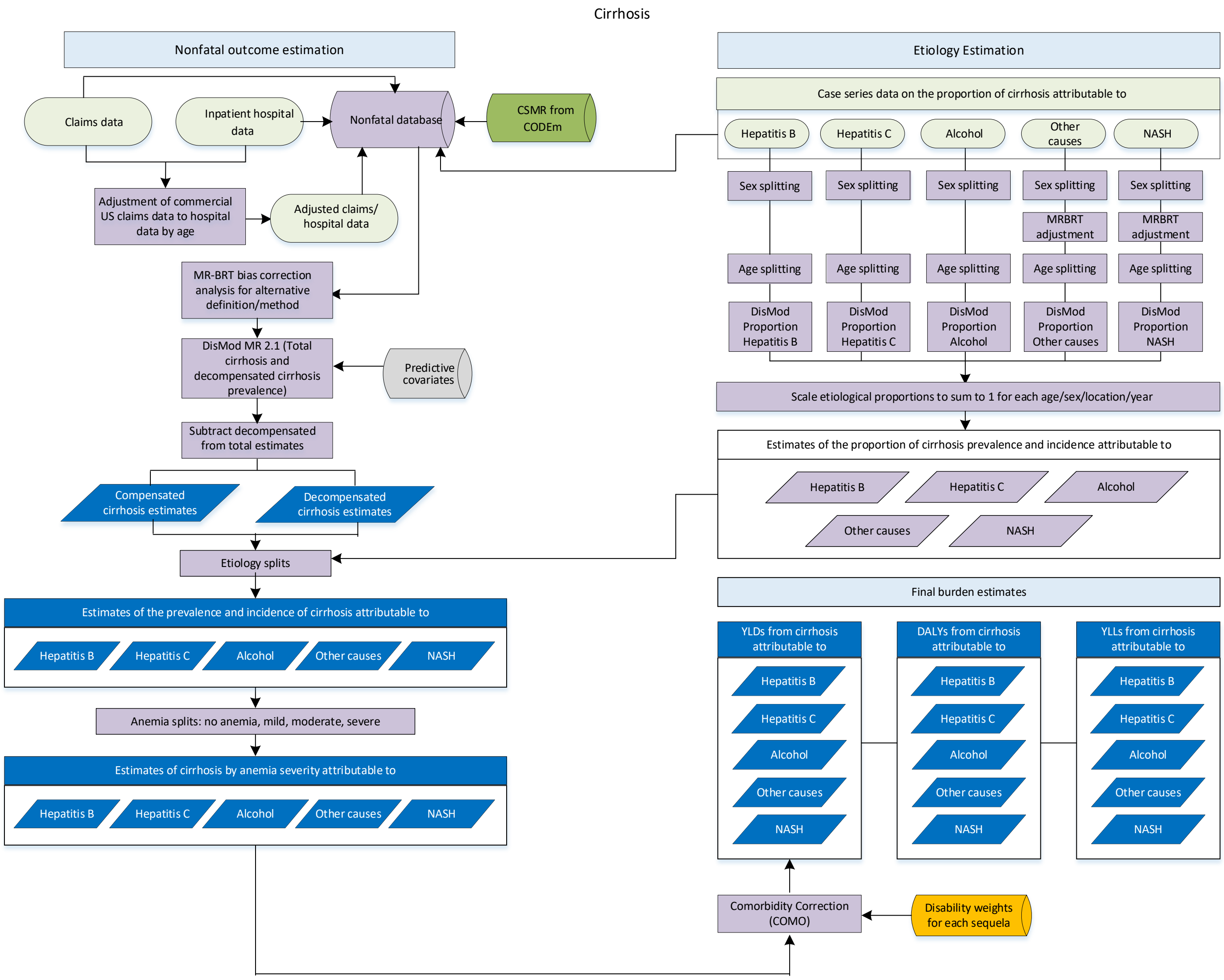

Figure 5: Flowchart of GBD cirrhosis incidence, prevalence, YLD estimation

4.2 Aetologic proportion data

In GBD 2016,[6] we performed a systematic review of published cirrhosis case-series that report the proportion of cases due to specific aetiologies: hepatitis B, hepatitis C, alcohol, NAFLD, and other causes. Examples of aetiologies included in "other causes" include cryptogenic, haemochromatosis, and primary biliary cholangitis.

Search terms: ("liver cirrhosis?[All Fields]) AND ((?hepatitis B?[All Fields] OR "Hepatitis B"[Mesh] OR "Hepatitis B virus"[Mesh] OR "Hepatitis B Antibodies"[Mesh] OR "Hepatitis B Antigens"[Mesh]) OR (?hepatitis C?[All Fields] OR "Hepatitis C"[Mesh] OR ?hepatitis C antibodies?[MESH] OR "Hepatitis C Antigens"[Mesh] OR ?Hepacivirus"[Mesh]) OR ("alcohol"[All Fields] OR "Alcohol Drinking"[Mesh] OR "Alcohol-Related Disorders"[Mesh] OR "Alcoholism"[Mesh] OR "Alcohol-Induced Disorders"[Mesh])) AND NOT (animals[MeSH] NOT humans[MeSH])

To be included, a study had to report the aetiology of cirrhosis for a representative sample of those with compensated or decompensated cirrhosis. Sufficient information on study method and sample characteristics was required to assess the quality of the study. Cases due to hepatitis B were confirmed based on presence of HBsAg.

Although methods of processing and modeling these proportion data were updated in GBD 2019, no new data were added. We plan to update the systematic review in future rounds of the GBD to expand our dataset.

Figure 6: Data inputs for the cirrhosis due to hepatitis B aetologic proportion model

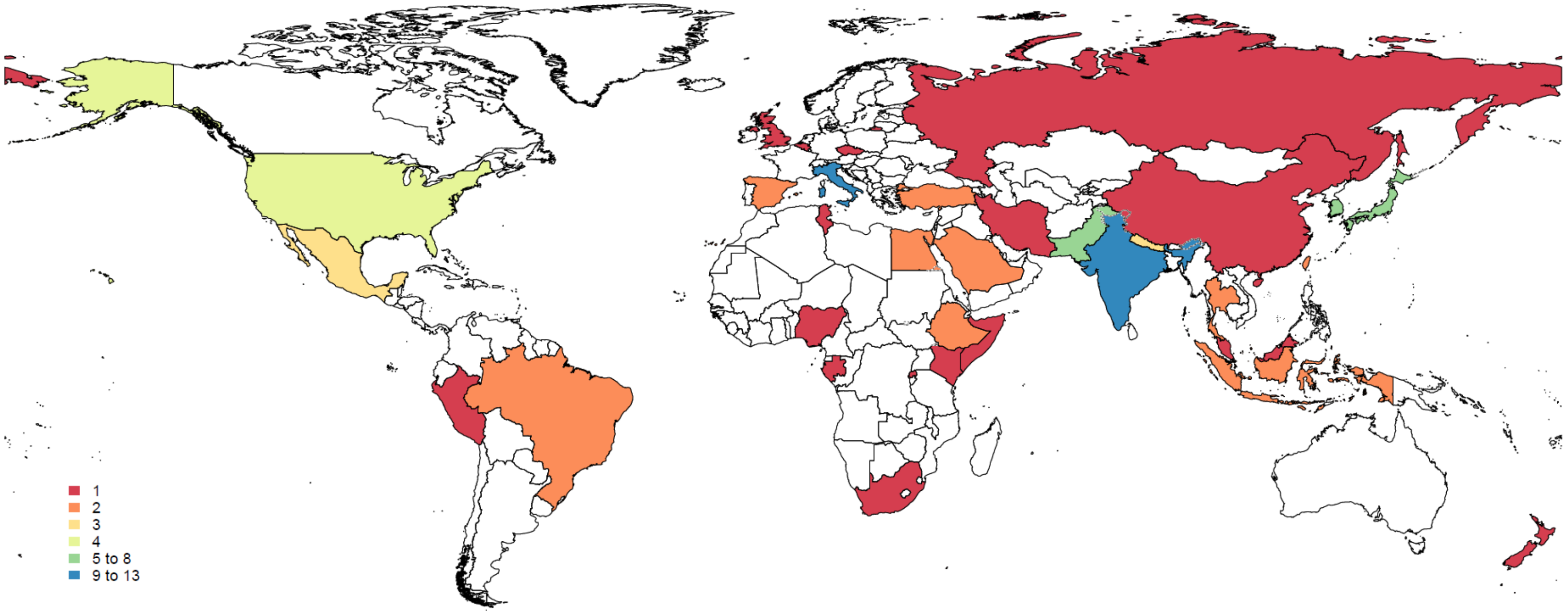

4.3 Clinical administrative data

We modelled total cirrhosis and decompensated cirrhosis prevalence based on hospital discharge data and claims data. Details of the extraction, utilization envelope and correction factor models used to process hospital discharge and claims data for GBD are found in the "Claims, inpatient hospital and outpatient data" section of the appendix to the GBD 2019 Diseases & Injuries report.[9]

In brief, and with regard to this report, individuals in claims databases were identified as prevalent cases of total cirrhosis if they had at least one inpatient encounter or two outpatient encounters with a relevant ICD code (as primary or other diagnosis) in a 12-month period. Individuals in claims databases were identified as prevalent cases of decompensated cirrhosis if they had at least one inpatient encounter with a relevant ICD code as primary diagnosis in a 12-month period. The population sample from which those cases arose was considered to comprise all individuals enrolled in that insurance plan that year.

Claims data, which link claims for multiple encounters to individuals, were also used to derive correction factors to estimate population prevalence from sources that only provide data on inpatient discharges. For each combination of year, age, sex, and location in claims data, we calculated a correction factor between inpatient discharges and prevalence for both total cirrhosis and decompensated cirrhosis. For total cirrhosis, we calculated the ratio of inpatient discharges with a cirrhosis code as primary diagnosis to all prevalent cirrhosis cases by inpatient or outpatient criteria. The logs of these ratios were calculated and entered into a non-linear mixed-effects model in MR-BRT[9,10] with covariates for age-groups and sex and 20% trimming. For decompensated cirrhosis, we calculated the ratio of inpatient discharges with a cirrhosis code as primary diagnosis to all prevalent cirrhosis cases by inpatient criteria, only. The log of these ratios was also calculated and modeled in MR-BRT with covariates for age and sex and 20% trimming.

Our hospital data sources provide information on inpatient discharges, rather than individuals. Inpatient discharges with ICDs for cirrhosis as primary diagnosis were identified from inpatient discharge data. For each source, we calculated the fraction of all hospital admissions primarily due to cirrhosis for a given year, age-group, sex, and this fraction was multiplied by that group's annual hospital utilization, estimated as previously described.[9] These annual rates of primary cirrhosis admission were then multiplied by the correction factors from claims data described above and a scalar based on HAQi to estimate year-age-sex-specific population prevalence for each source.

Table 4 reports ICD codes included in cirrhosis non-fatal estimation.

Table 4: List of International Classification of Diseases (ICD) codes mapped to the Global Burden of Disease cause list for cirrhosis prevalence data

ICD system	ICG name	ICD codes
10	Digest, liver, varices	I85, I85.0, I85.00, I85.01, I85.1, I85.10, I85.11, I85.9, I98.2
	Liver, chronic, etoh, non-cirrhosis	K70, K70.0, K70.1, K70.10
	Liver, chronic, etoh, cirrhosis	K70.11, K70.2, K70.3, K70.30, K70.31, K70.4, K70.40, K70.41, K70.9
	Liver, toxic liver disease	K71, K71.3, K71.4, K71.5, K71.50, K71.51, K71.6, K71.8
	Liver, chronic, non-etoh, non-cirrhosis	K71.7, K71.9, K73, K73.0, K73.1, K73.2, K73.8, K73.9, K74.0, K74.1, K74.2
	Liver, failure	K72, K72.1, K72.10, K72.11, K72.9, K72.90, K72.91
	Liver, chronic, non-etoh, cirrhosis	K74, K74.3, K74.4, K74.5, K74.6, K74.60, K74.69, K74.7, K74.8, K74.9, K75.8, K75.81, K75.89, R18, R18.0, R18.8, R18.9
	Liver, other	K75, K75.2, K75.4, K75.9, K76, K76.1, K76.2, K76.4, K76.5, K76.6, K76.7, K76.8, K76.81, K76.89, K76.9, K77, K77.0, K77.8
9	Digest, liver, varices	456.0, 456.1, 456.2, 456.20, 456.21
	Liver, failure	570, 570.0, 570.9
	Liver, chronic, etoh, unspecified	571
	Liver, chronic, non-etoh, non-cirrhosis	571.4, 571.40, 571.41, 571.42, 571.49, 571.8
	Liver, chronic, non-etoh, cirrhosis	571.5, 571.6, 572.2, 572.3, 572.4, 573, 573.5, 573.8, 573.9
	Liver, other	572, 572.5, 572.6, 572.8, 572.9, 573.0, 573.1, 573.2, 573.3, 573.4

Figure 7: Data inputs for total cirrhosis and other chronic liver diseases

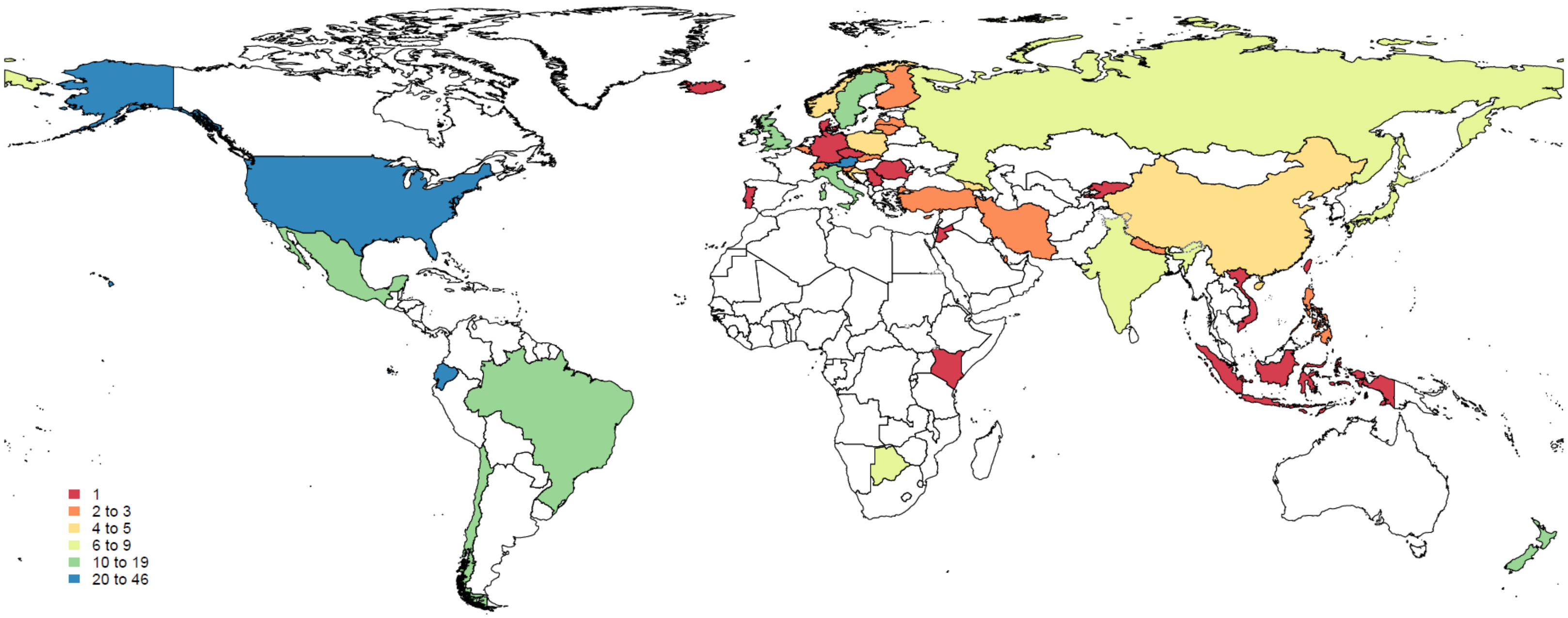

Figure 8: Data inputs for decompensated cirrhosis and other chronic liver diseases

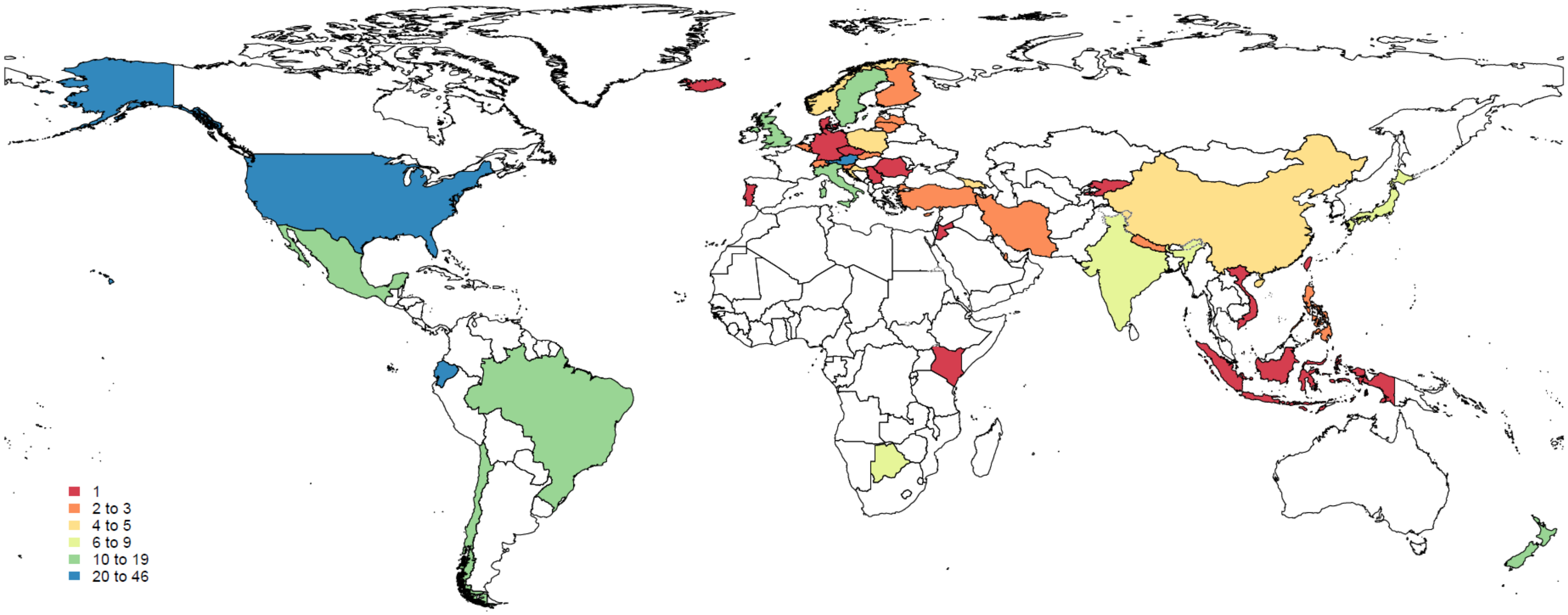

4.4 Cause-specific mortality rate inputs

We included CoDCorrected cirrhosis cause-specific mortality rate (CSMR) estimates as inputs in the total cirrhosis and decompensated cirrhosis DisMod models. We used non-prevalence data to help solve the differential equations of the compartmental framework and to promote internal consistency between fatal and non-fatal estimates of cirrhosis.[8,21]

4.5 Excess mortality rate inputs

We modeled excess mortality rates from CoDCorrected CSMR estimates (from section 4.4) and cirrhosis prevalence data (from section 4.3) to use as additional inputs into our total and decompensated cirrhosis DisMod models.

In previous rounds, across numerous GBD diseases and injuries, priors on excess mortality rate (EMR) were estimated in DisMod by matching prevalence data-points with their corresponding CSMR values within the year, same age, sex, location (by dividing CSMR by prevalence).[6,22] For many diseases and injuries, however, including cirrhosis, DisMod estimated a rather unrealistic pattern of EMR compared to an expected pattern of decreasing EMR with greater access to quality health care. GBD leadership surmised this may signal inconsistencies between CSMR estimates and measurements of prevalence and/or incidence, and in GBD 2019 developed a new method modeling EMR inputs for use in DisMod models across multiple diseases and injuries, which is described on pages 465-6 of Appendix 1of the GBD 2019 Diseases and Injuries Capstone.[9]

In brief, and with regard to this report, EMR inputs for the total cirrhosis DisMod model were generated by matching prevalence data-points (from 4.3) with their corresponding CSMR estimates (from 4.4) within the same year, age, sex, location (by dividing CSMR by prevalence). These were log-transformed and modeled with a nonlinear mixed effects model using the MR-BRT tool,[9,10] with fixed effects for age, sex, and Healthcare Access and Quality Index (HAQi), random effects on unique combinations of study and location, and 10% trimming. Age was modeled using a cubic spline with linear tails, and three evenly spaced knots. The prior on HAQi was to have a negative coefficient.

$$\log(EMR) = \beta_0 + \beta_1 sex + \beta_2 HAQ + f(age) + u_{loc-study} + \epsilon_i$$

EMR was then predicted for each year, sex, location and for ages 0, 10, 20 ….100, using the coefficients from the fitted MR-BRT model.

Figure 9. MR-BRT model results (line) overlaid on EMR calculated from prevalence data points and matched CSMR estimates (dots) for total cirrhosis EMR input estimation

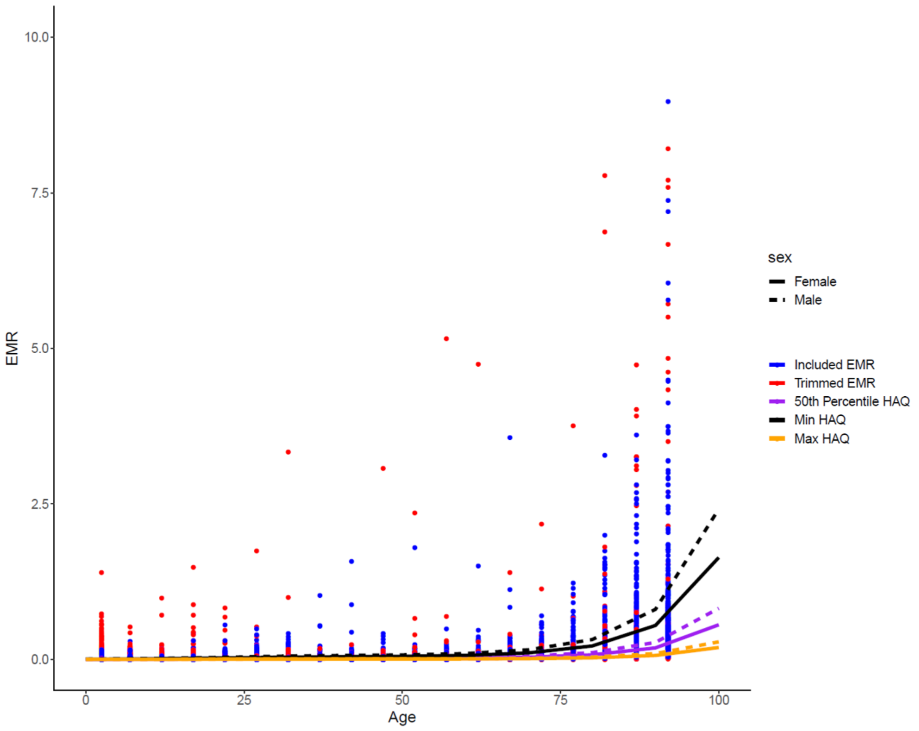

The same approach was taken to produce EMR inputs for the decompensated cirrhosis DisMod model, using decompensated cirrhosis prevalence data (from 4.3) divided by their corresponding CSMR estimates (from 4.4), and entering these quotients in an MR-BRT model of log(EMR), using fixed effects for age, sex, and Healthcare Access and Quality Index (HAQi), random effects on unique combinations of study and location, and 10% trimming. Age was modeled using a cubic spline with linear tails, and three evenly spaced knots. The prior on HAQi was to have a negative coefficient.

Figure 10: MR-BRT model results (line) overlaid on EMR calculated from prevalence data points and matched CSMR estimates (dots) for decompensated cirrhosis EMR input estimation

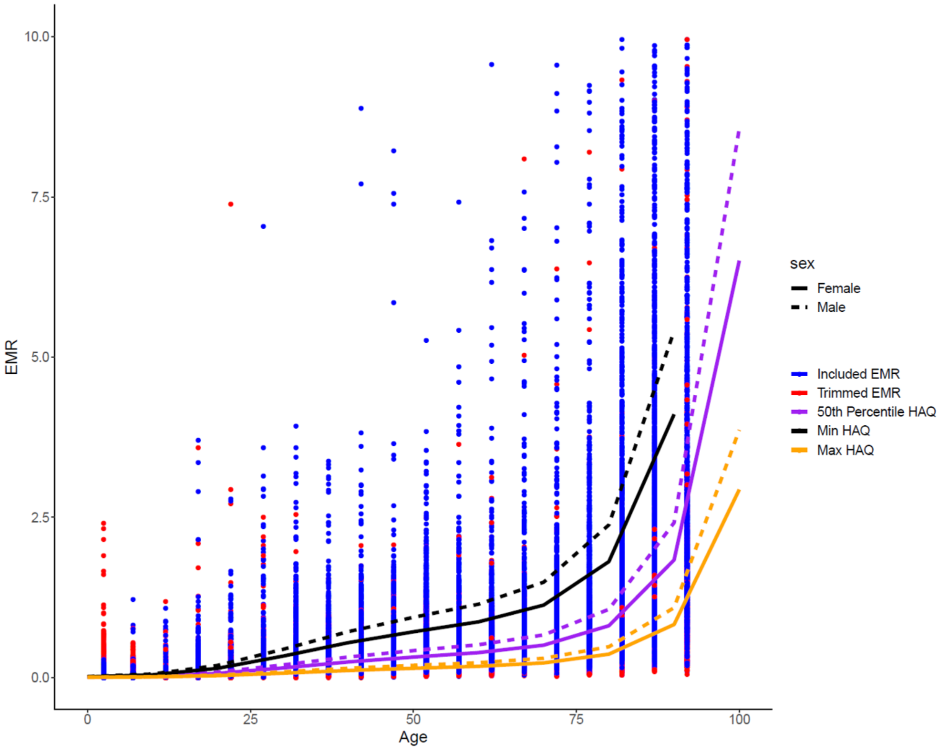

4.6 Aetiological proportion data adjustment

Prior to modelling, we performed several data adjustments to correct proportion data for non-"ideal" features, including "multi-aetiology case splitting", "sex-splitting", and "age-splitting".

Some studies reported cases in which multiple risk factors for cirrhosis were identified. There were, however, insufficient multiple-aetiology data to estimate combinations of aetiologies in distinct models. Instead, we reassigned these multi-aetiology cases to single aetiologies using the proportions observed in cases with only one aetiology in the same study.

Take as an example a study that reported 100 cases of cirrhosis total, of which 40 cases were due to hepatitis B, 20 due to alcohol, 30 due to NAFLD, and ten due to hepatitis B and alcohol. We must redistribute cases due to both hepatitis B and alcohol proportionate to cases of each aetiology separately, holding the cases due to NAFLD unchanged. We would redistribute the ten cases of hepatitis B and alcohol by a ratio of 40:20, resulting in 47 cases of hepatitis B and 23 cases of alcohol. Figure 11 illustrates for this example the proportion of cirrhosis cases assigned to each aetiology before and after adjustment of multi-aetiology cases.

Figure 11: Pre- and post-adjustment of multi-aetiology cases

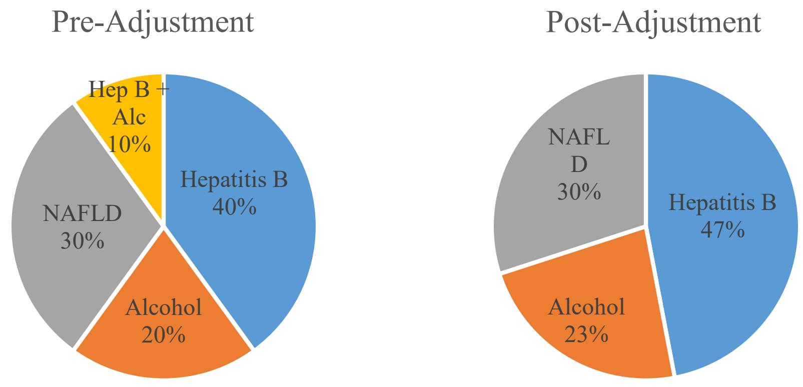

We used the "sex-splitting" and "age-splitting" method described in Section 3.5 to adjust both sex proportion data into male- and female-specific data-points and broad age data. The MR-BRT sex-ratio model used a single intercept term with an uninformative prior, random effects for each case-series, and 10% trimming. Table 5 below reports the estimated sex ratio. The age-pattern applied to split data from broad age-groups was the age-pattern in GBD 2017 estimates.

Table 5: MR-BRT sex ratios for proportion cirrhosis due to hepatitis B

Data input	Gamma	Beta coefficient, log (95% CI)	Exponentiated beta coefficient (95% CI)
Cirrhosis due to hepatitis B	0.155	–0.21 (–0.56 to 0.14)	0.81 (0.57 to 1.15)

4.7 Clinical informatics data adjustment

We performed "crosswalks", or bias adjustments, to adjust non-reference data sources toward reference data for both total cirrhosis and decompensated cirrhosis data using the "crosswalk" method, as described in Section 4.3. These adjustments affect only a subset of data for the United States. The United States MarketScan data may be biased because this data source only includes patients with commercial insurance, which may differ systematically in both underlying disease epidemiology and in case ascertainment (via healthcare utilisation) compared to a general population. On the other hand, MarketScan data are available for all U.S. states, whereas population-representative hospital data sources are only available for a subset of U.S. states. We identified pairs of MarketScan and hospital data for the same year, age, sex, and location, calculated the logit difference between the prevalence measurements from each source, and modeled the logit difference in MR-BRT with fixed effects for age, random effects on unique combinations of study, age, sex, year, and location, and 10% trimming. We conducted an

analysis in MR-BRT with a spline on age so that adjustments could be performed differentially by age. After the year 2000, there was a change in reporting in claims data. Because of this, the analysis was conducted between MarketScan data in 2000 compared to hospital data in 2000, and then all other years of MarketScan data compared to other years of hospital data.

Figure 12: MR-BRT adjustment factors for decompensated cirrhosis, (A) Marketscan 2000, (B) Marketscan other years

(A)

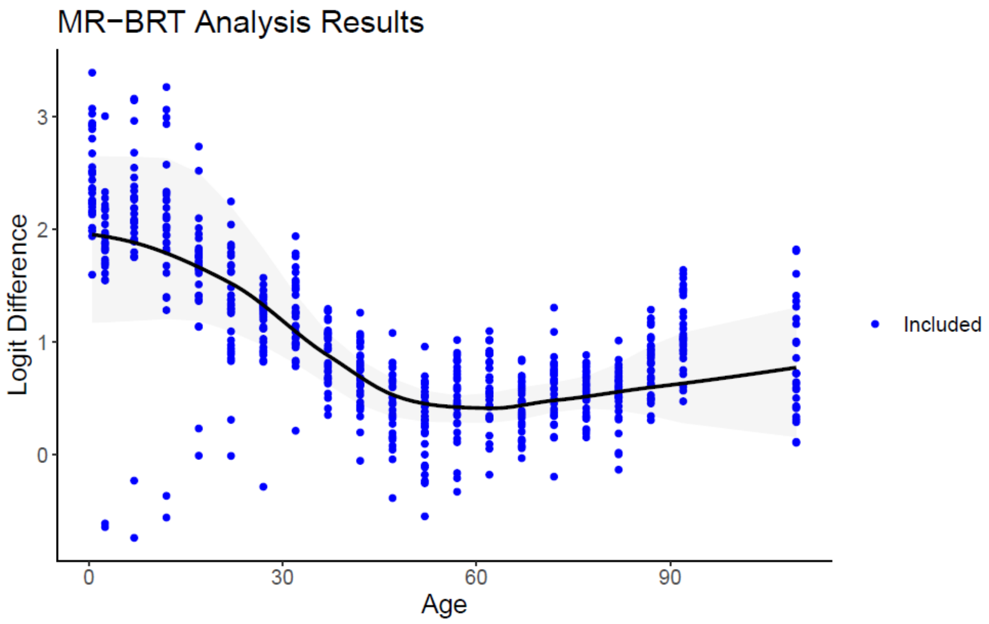

(B)

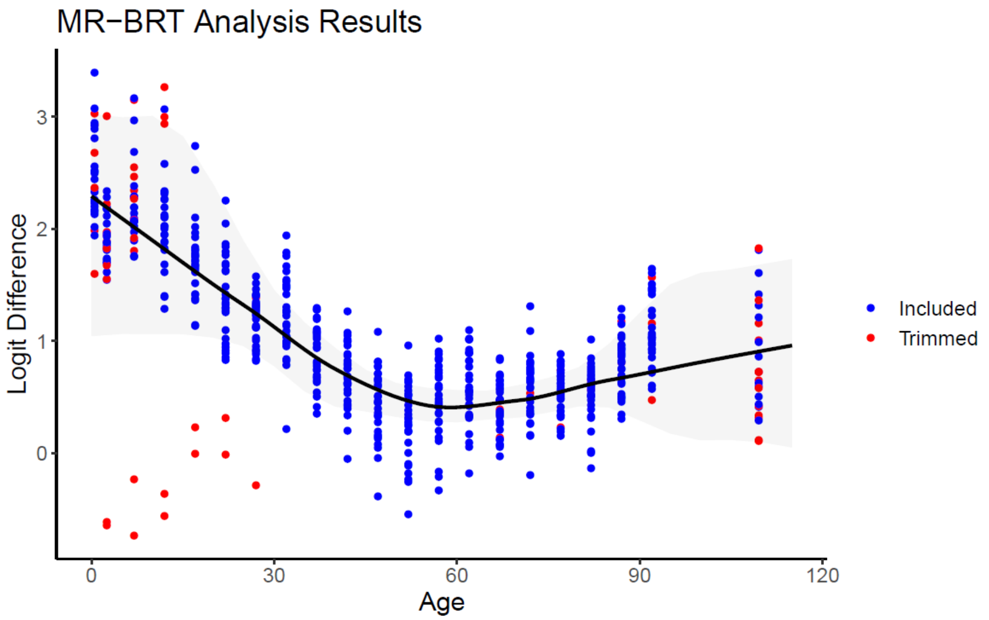

Figure 13: MR-BRT adjustment factors for total cirrhosis, (A) Marketscan 2000, (B) Marketscan other years

(A)

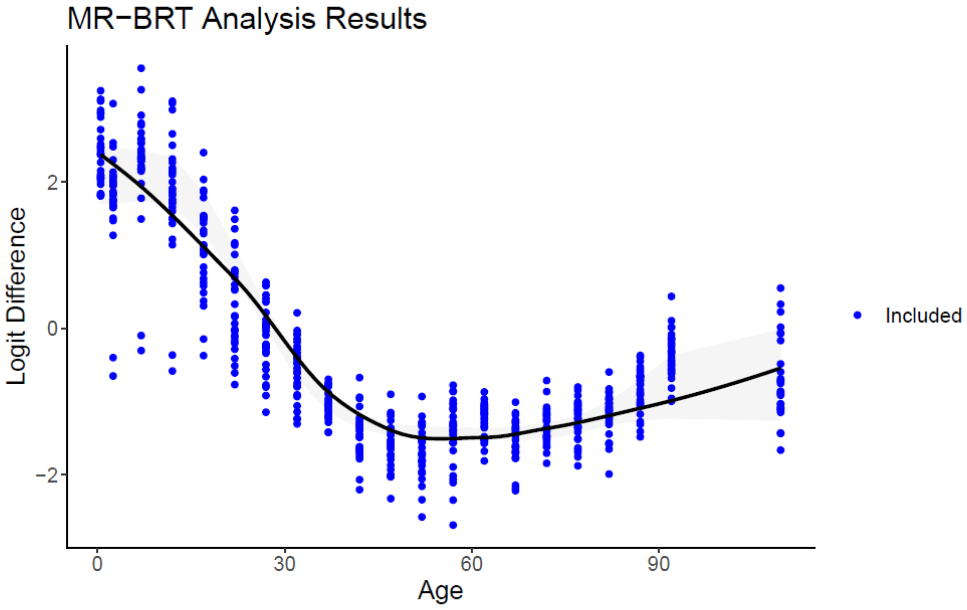

(B)

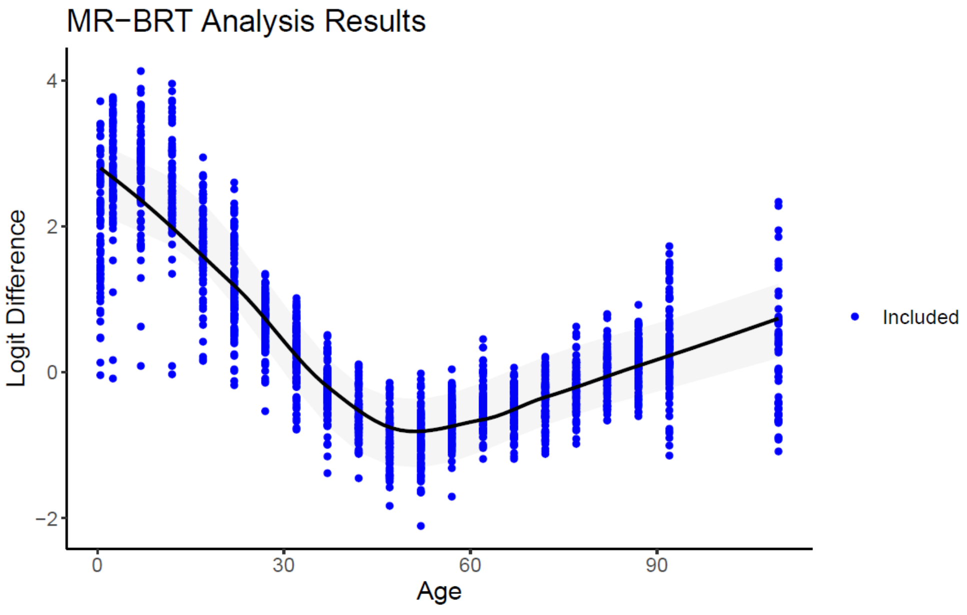

4.8 Modelling total cirrhosis and other chronic liver diseases non-fatal

The processed total cirrhosis hospital and claims data (sections 4.3 and 4.7), CSMR (4.4), and EMR (4.5) were entered into a Bayesian multi-regression model in DisMod-MR 2.1,[8,21] along with predictive covariates to improve estimation where these above inputs were absent or scarce. We set the prior on remission to 0 for all ages and the prior on EMR to 0 to 0.02 for ages 0 to 9 years. The minimum and maximum values for the prior for the fixed effect of HAQi on EMR

were based on the mean and standard deviation estimated in the MR-BRT model in section 4.5. A summary of covariates used in the total cirrhosis and other chronic liver diseases DisMod-MR 2.1 model are listed in Table 6.

Table 6: Covariates used in DisMod-MR 2.1 model of total cirrhosis

Covariate	Parameter	Prior (min, max)	Exponentiated beta (95% UI)
Hepatitis B seroprevalence (HBsAg) age-standardised	Prevalence	(0, 4)	39.30 (29.25–50.60)
Hepatitis C seroprevalence (anti-HCV) age-standardised	Prevalence	(0, 4)	1.34 (1.01–2.26)
Litres of alcohol consumed per capita	Prevalence	(0, 2)	1.00 (1.00–1.00)
Prevalence of obesity	Prevalence	(0, 2)	1.05 (1.00–1.16)
Healthcare Access and Quality Index (HAQi)	Excess mortality rate	(-0.025, -0.023)	0.98 (0.98–0.98)

The data inputs and modeling approaches used to estimate hepatitis C seroprevalence,[9] litres of alcohol consumed per capita,[11] prevalence of obesity,[11] and Healthcare Access and Quality Index (HAQi)[9,13] for GBD have been previously described. These processes result in estimates of these covariates for every year-age-sex-location combination in the GBD framework, and they are updated with each round of GBD. The prior for the HAQi covariate on EMR was based on the mean and standard deviation for this relationship estimated in the MR-BRT model described in section 4.5.

4.9 Modelling decompensated cirrhosis and other chronic liver diseases non-fatal

The processed decompensated cirrhosis hospital and claims data (sections 4.3 and 4.7), CSMR (4.4), and EMR (4.5) were entered into a Bayesian multi-regression model in DisMod-MR 2.1,[8,21] along with predictive covariates to improve estimation where these above inputs were absent or scarce. We set the prior on remission to 0 for all ages and the prior on EMR to 0 to 0.1 for ages 0 to 9y. A summary of covariates used in the decompensated cirrhosis and other chronic liver diseases DisMod-MR 2.1 model are listed in Table 7.

Table 7: Covariates used in DisMod-MR 2.1 model of decompensated cirrhosis

Covariate	Parameter	Prior (min, max)	Exponentiated beta (95% UI)
Hepatitis B seroprevalence (HBsAg) age-standardised	Prevalence	(0,4)	53.28 (51.37–54.54)
Hepatitis C seroprevalence (anti-HCV) age-standardised	Prevalence	(0,4)	13.04 (3.49–40.45)
Litres of alcohol consumed per capita	Prevalence	(0,2)	1.00 (1.00–1.00)
Prevalence of obesity	Prevalence	(0,2)	1.01 (1.00–1.02)
Healthcare Access and Quality Index	Excess mortality rate	(-0.019, -0.018)	0.98 (0.98–0.98)

The data inputs and modeling approaches used to estimate hepatitis C seroprevalence,[9] litres of alcohol consumed per capita,[11] prevalence of obesity,[11] and Healthcare Access and Quality Index (HAQi)[9,13] for GBD have been previously described. These processes result in estimates of these covariates for every year-age-sex-location combination in the GBD framework, and they are updated with each round of GBD. The prior for the HAQi covariate on EMR was based on the mean and standard deviation for this relationship estimated in the MR-BRT model described in section 4.5.

4.10 Modelling proportion of cirrhosis due to hepatitis B

We used the adjusted proportion of cirrhosis due to hepatitis B data (sections 4.2 and 4.6) in a single-parameter regression model in DisMod-MR 2.1. In contrast to the compartmental framework of many DisMod models,[8,21] which incorporates data for multiple epidemiologic measures and simultaneously solves multiple regression models for a system of differential equations, a single-parameter DisMod model uses only one data type to estimate a single quantity (in this case, proportions) while still making use of DisMod's Bayesian meta-regression and geographic cascade features.

We included predictive covariates in the model to improve estimation for areas where data were scant or absent. We also included covariates with specified negative associations for the proportions of cirrhosis in the other four estimated aetiologies. Table 8 summarises covariates used in the single-parameter proportion model.

Table 8: Covariates used in the proportion of cirrhosis due to hepatitis B DisMod-MR model

Covariate	Prior (min, max)	Exponentiated beta (95% UI)
Hepatitis B seroprevalence (HBsAg) age-standardised	(0,1)	2.37 (1.88–2.70)
Proportion of liver cancer due to hepatitis B (age-standardised)	(0,1)	1.59 (1.17–2.16)
Hepatitis B 3-dose coverage (proportion), lagged 10 years	(-1,0)	0.50 (0.45–0.55)
Proportion of cirrhosis due to alcohol	(-1,0)	0.88 (0.70–0.99)
Proportion of cirrhosis due to hepatitis C	(-1,0)	0.41 (0.37–0.50)
Proportion of cirrhosis due to other causes	(-1,0)	0.93 (0.82–1.00)
Proportion of cirrhosis due to NASH	(-1,0)	0.69 (0.45–0.98)

Separate single-parameter DisMod models were run to estimate the proportion of cirrhosis due to the four specified aetiologies other than hepatitis B and the results of the five proportion models were re-scaled at the draw level for each year, age, sex and location to produce final proportion estimates. Models for aetiologies other than hepatitis B have been previously described.[21]

4.11 Modelling compensated cirrhosis and other chronic liver diseases

Estimates of the prevalence of compensated cirrhosis were produced by subtracting estimates of decompensated cirrhosis prevalence (produced as described in 4.9) from estimates of total cirrhosis prevalence (produced as described in 4.8). This subtraction was carried out at the draw level to generate 1000 draws of compensated cirrhosis prevalence, which provides an estimated mean with 95% uncertainty interval.

$$compensated\ cirrhosis_{yasl} = total\ cirrhosis_{yasl} - decompensated\ cirrhosis_{yasl};$$

where yasl is the specific year-age-sex-location.

4.12 Modelling aetiology-specific estimates

We applied the scaled proportions of the five modelled aetiologies of cirrhosis (hepatitis B, hepatitis C, alcohol use, NAFLD, and other causes; see sections 4.2, 4.6 and 4.8 for additional details) to decompensated and compensated cirrhosis estimates to create estimates of cirrhosis by aetiology.

In GBD 2019, we estimated the proportion of those with decompensated cirrhosis that had complications due to anaemia as part of the anaemia causal attribution process described on pages 1368-76 of Appendix 1 of the GBD 2019 Diseases and Injuries Capstone.[9] We applied

scaled proportions of the modelled anaemia severities (no anaemia, mild anaemia, moderate anaemia, and severe anaemia) to decompensated cirrhosis estimates to create estimates of decompensated cirrhosis by both aetiology and anaemia severity.

4.13 Disability weights

GBD estimation of disability weights for estimation of years lived with disability (YLDs) in GBD has been previously described.[9,20,23,24] In brief, a series of household and web-based surveys were conducted between 2009 and 2013, ultimately in nine different countries. In these, respondents were presented a series descriptions of two hypothetical people, each with a particular health state, and asked which individual was healthier. The descriptions were written in lay language and described symptoms and function loss in generic terms. Probit regression analyses of the frequencies of answers to the pair-wise comparisons were used to infer distances between disability weight values for pairs of states, and then those distances were anchored to a 0-1 scale.

For cirrhosis, compensated cases were assigned an asymptomatic health state, with disability weight of zero. Cases of decompensated cirrhosis with varying levels of anaemia were assigned disability weights as shown in Table 9.[19,20]

Table 9. Disability weights of decompensated cirrhosis

Health state	Lay description	Disability weight (95% uncertainty interval)
Decompensated cirrhosis of the liver and no anaemia	Has a swollen belly and swollen legs. The person feels weakness, fatigue, and loss of appetite.	0.178 (0.113–0.243)
Decompensated cirrhosis of the liver and mild anaemia	Has a swollen belly and swollen legs. The person feels weakness, fatigue, and loss of appetite.<br><br>Feels slightly tired and weak at times, but this does not interfere with normal daily activities.	0.181<br><br>(0.116–0.246)
Decompensated cirrhosis of the liver and moderate anaemia	Has a swollen belly and swollen legs. The person feels weakness, fatigue, and loss of appetite.<br><br>Feels moderate fatigue, weakness, and shortness of breath after exercise, making daily activities more difficult.	0.220<br><br>(0.146 – 0.295)
Decompensated cirrhosis of the liver and severe anaemia	Has a swollen belly and swollen legs. The person feels weakness, fatigue, and loss of appetite.	0.300<br><br>(0.202–0.397)

	Feels very weak, tired, and short of breath, and has problems with activities that require physical effort or deep concentration.	

5 Cirrhosis and other chronic liver diseases fatal estimation

5.1 Flowchart

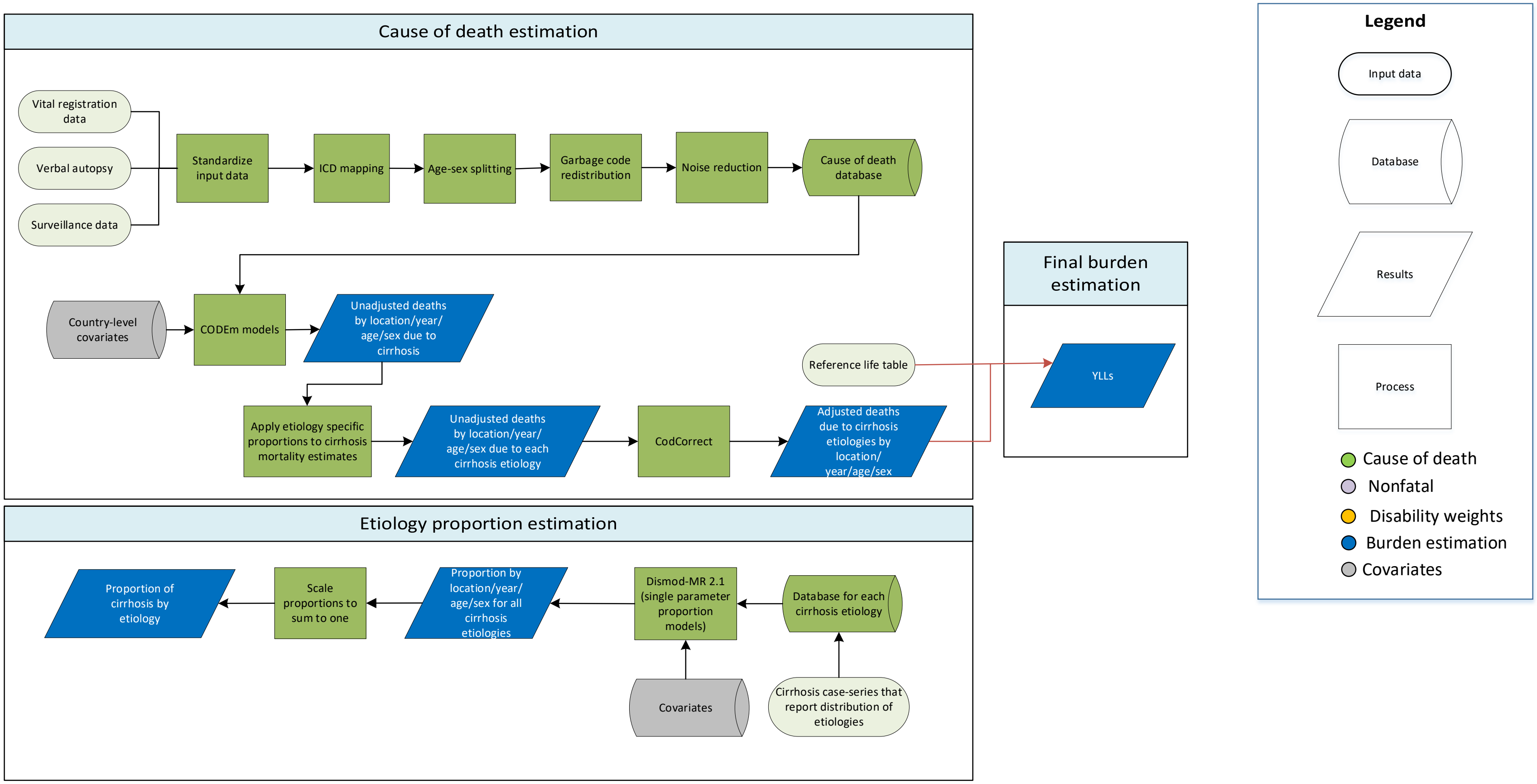

Figure 14: GBD cirrhosis mortality, YLL estimation

5.2 Data identification and processing

We used vital registration (VR), surveillance, and verbal autopsy (VA) data to model cirrhosis and other chronic liver diseases mortality. The details of processing cause of death (COD) data for GBD, including mapping, disaggregation, age-sex splitting, corrections for misclassification, redistribution of garbage codes, and noise reduction have been previously described.[9,25] The ICD codes that map directly to cirrhosis are included in Table 10.

Table 10: List of International Classification of Diseases (ICD) codes mapped to the Global Burden of Disease cause list for cirrhosis mortality data

ICD System	ICD Codes
10	B18, I85, K70, K70.0, K70.1, K70.10, K70.11, K70.2, K70.3, K70.30, K70.31, K71.7, K73, K74, K75, K75.2, K75.4, K75.8, K75.81, K75.89, K75.9, K76, K76.0, K76.1, K76.2, K76.4, K76.5, K76.6, K76.7, K76.8, K76.81, K76.89, K76.9, K77.8;
9	070.22, 070.33, 070.54, 456.0, 456.1, 456.2, 456.20, 456.21, 571, 571.0, 571.2, 571.3, 571.4, 571.5, 571.6, 571.8, 571.9, 572.2, 572.3, 572.4, 572.5, 572.6, 572.8, 572.9, 573, 573.0, 573.4, 573.5, 573.8, 573.9

Figure 15: Mortality data inputs for the fatal model of cirrhosis and other chronic liver diseases

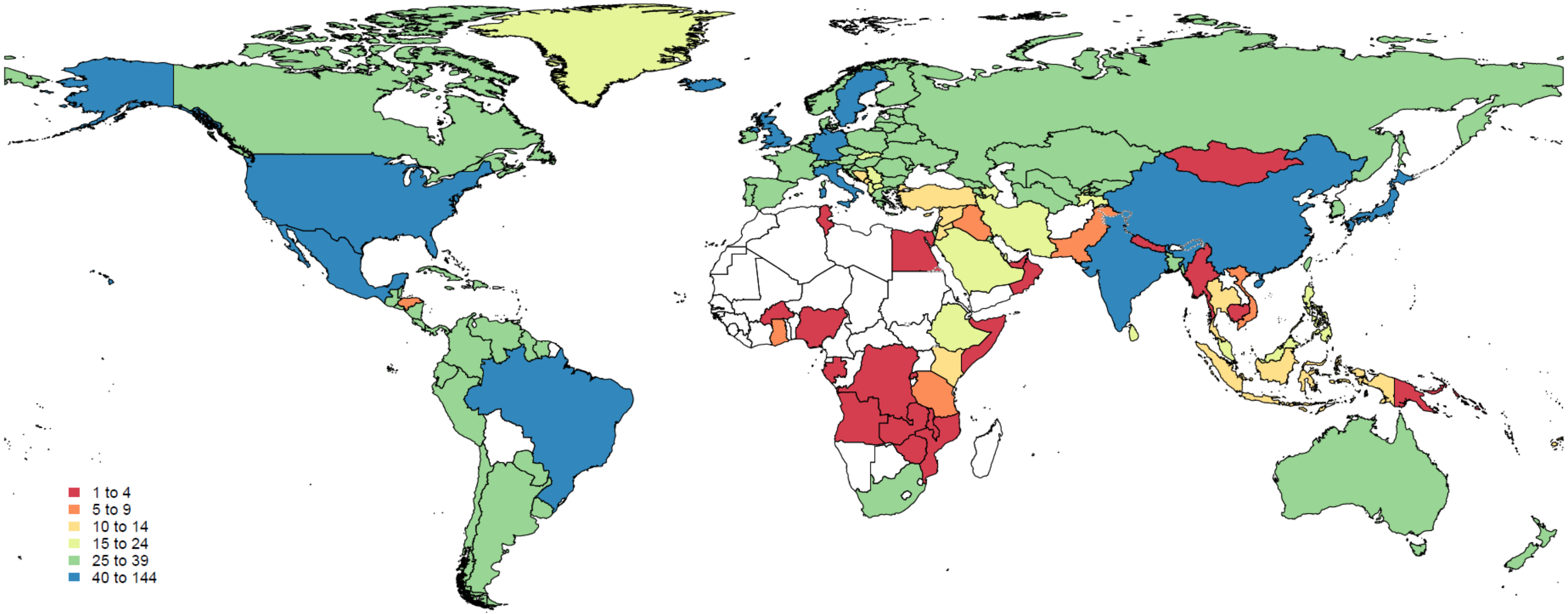

An important component of mortality estimation is redistribution of vague, impossible, intermediate, or immediate causes of death to valid underlying causes of death in the GBD cause list.[21] We account for every death from all known data sources, but some records are inaccurate or inconsistent and need to be processed prior to modelling. In GBD 2019, we reviewed vital registration data sources that record information on underlying cause of death, intermediate/immediate cause of death, and predisposing conditions, and noted that many "unspecified hepatitis" deaths included chronic liver diseases in the cause of death chain. Consequently, those deaths were proportionately redistributed to cirrhosis and other chronic liver diseases and acute hepatitis, with the majority assigned to the former, as shown in Figure 16.

Figure 16: Sankey diagram illustrating ICD-10 redistribution of unspecified hepatitis codes of death onto other causes of deaths

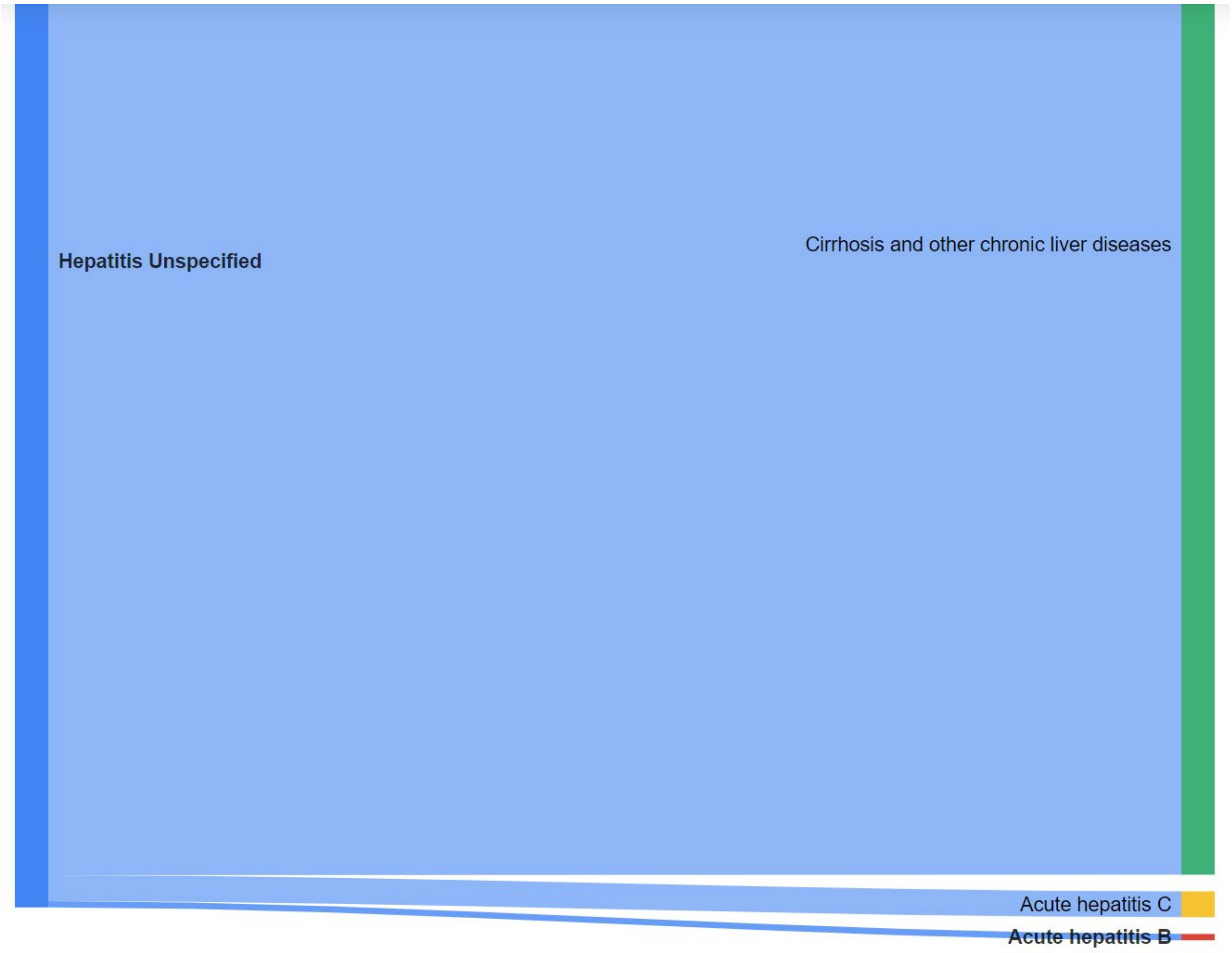

We marked data as outliers and excluded them in instances where garbage code redistribution and noise reduction, in combination with small sample sizes, resulted in unreasonable cause fractions or unreasonable time, age, or spatial trends.

5.3 Modelling cirrhosis and other chronic liver diseases mortality

We estimated cirrhosis mortality using the Cause of Death Ensemble (CODEm) model tool, a model generation and ensemble selection tool developed and periodically updated for GBD; the original CODEm tool and subsequent updates have been previously described.[21,26] In brief, CODEm estimates the mortality rate due to a given cause, for every combination of age-group, location and year from 1980 to 2019 by generating a diverse set of plausible statistical models ("sub-models") and evaluating differently weighted combinations of sub-models to select the ensemble model ("CODEm model") with the highest out-of-sample predictive validity. Four families of sub-models are generated for consideration, including models of logit cause-fractions (deaths due to cause of interest out of all deaths) and models of log cause-specific mortality rates (deaths due to cause of interest as population rate), and both linear mixed effect regression (LMER) models and spatiotemporal Gaussian process regression (ST-GPR) models.

Details of the fixed and random effects for LMER models, calculation of residual weights for spatiotemporal regression, determination of Gaussian process regression inputs (amplitude, scale, degree of differentiability, and data variance), and estimation of sub-model uncertainty are available in previous publications.[21,26] Potential covariates are selected based on externally established associations and entered into the CODEm algorithm in a ranked fashion, with the covariates with the strongest associations and evidence for causal and proximate relationships marked as "Level 1", covariates with strong associations but suspected distal or indirect relationships entered as "Level 2", and covariates with the weakest or most distal relationships marked "Level 3". All covariates are marked with their expected direction of association. Sub-models from all four families are generated with all possible Level 1 covariate combinations, and retained if the sign on all covariate coefficients is in the expected direction and significant at the $p < 0.05$ level. Level 2 covariates are then tested to see if adding them to the Level 1 sub-models retains the significance and direction of all the coefficients in the Level 1 sub-models and also meets the significance and direction criteria for the coefficient on the additional covariates. The sub-models retained after testing Level 2 covariates are then tested for the addition of Level 3 covariates. Sub-model generation is carried out using a training subset of 70% of the available data.

Retained sub-models are then ranked based on out-of-sample performance predicting a 15% data knock-out, and then combined into a set of ensemble models based on a monotonically declining weighting function and a pre-specified set of 21 weighting parameters. Each ensemble in the set is then evaluated based on performance predicting the remaining 15% of the data. Twenty-five train-test-test datasets are employed to stabilize ranking, and the test data-sets are selected to reflect observed patterns of missingness in the data.[26] Performance characteristics used to rank sub-models and select the best ensemble comprise: the root mean square error (RMSE) of the natural log of predicted death rates, and the percentage of consecutive-year prediction pairs for which the difference in log death rate between adjacent years has the same sign in both the test data and the predictions. The mean coverage across all 25 test sets is calculated for the final ensemble as the percent of the data in the test set included in the 95% prediction interval. . Table 11 below reports the covariates, level, and specified direction tested in the CODEm model for cirrhosis.

Table 21: Covariates, level, and specified direction tested in cirrhosis CODEm model

Level	Covariate	Direction
1	Litres of alcohol per capita	+
	Seroprevalence (HBsAg) age-standardised	+
	Seroprevalence (anti-HCV) age-standardised	+
	Hepatitis B vaccine coverage proportion, aged through time	-
2	Mean BMI	+
	Healthcare Access and Quality Index (HAQi)	-
	Diabetes prevalence age-standardised	+
	Schistosomiasis prevalence	+
	Intravenous drug use	+
3	Education (years per capita)	-
	Lag distributed income (LDI) (ln transformation)	-
	Socio-demographic Index (SDI)	-

The data inputs and modeling approaches used to estimate hepatitis C seroprevalence, diabetes prevalence, schistosomiasis prevalence,[9] litres of alcohol consumed per capita, mean body mass index, intravenous drug use,[11] Socio-demographic Index (SDI),[12] Healthcare Access and Quality Index (HAQi),[9,13] education,[27] and lag-distributed income[12] for GBD have been previously described. These processes result in estimates of these covariates for every year-age-sex-location combination in the GBD framework, and they are updated with each round of GBD.

5.4 Modelling aetiology-specific estimates

We multiplied scaled proportions of the five modelled aetiologies of cirrhosis (hepatitis B, hepatitis C, alcohol, NAFLD, and other causes; see sections 4.2, 4.6 and 4.8 for data inputs, processing and modeling details)) by the mortality estimates from CODEm to generate mortality estimates of cirrhosis by aetiology. These multiplications were carried out at the level of individual draws for each year, age-group, sex and most granular location.

6 Liver cancer fatal estimation

6.1 Flowchart

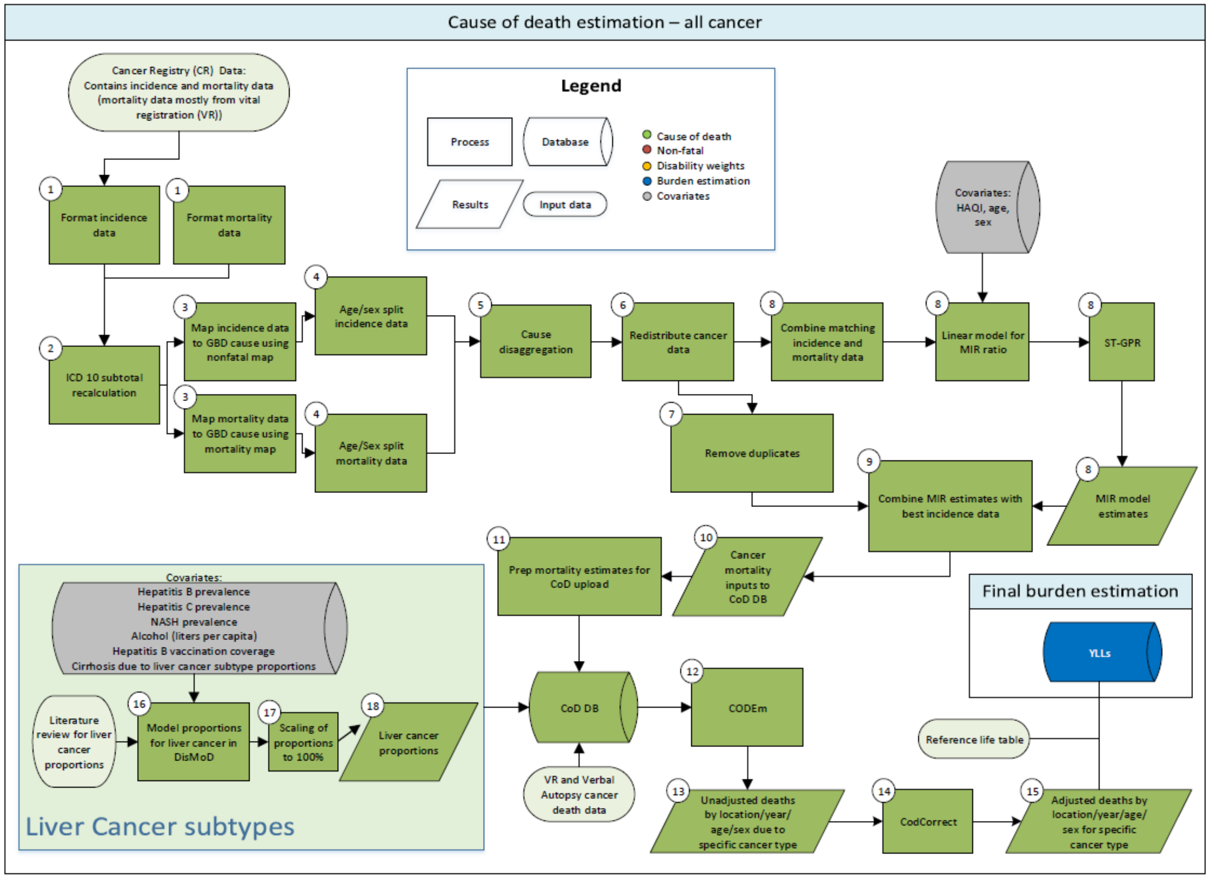

Abbreviations: ICD, International classification of diseases; DB, database; ST-GPR, spaciotemporal Gaussian process regression; COD, causes of death; MI, mortality to incidence ratio; CODEm, cause of death ensemble model; HAQI, healthcare access and quality index; NASH, nonalcoholic steatohepatitis

Figure 17: Flowchart GBD cancer mortality, YLL estimation

6.2 Data identification and processing

Data for estimating (primary) liver cancer mortality (without regard to etiology) included vital registration (VR), verbal autopsy (VA), and cancer registry data. VR and VA data were identified and processed using standard GBD approaches summarized in section 5.2 and detailed in Naghavi *et al*[25] and the methods supplement to the GBD 2019 Diseases & Injuries Capstone.[9] ICD codes used to map underlying cause of death from VR data to GBD causes of death are shown in Table 12.

Table 12: List of International Classification of Diseases (ICD) codes mapped to the Global Burden of Disease cause list for cancer mortality data

Cause	ICCC3	ICD10	ICD9
Liver cancer	VIIb, VIIc	C22, C22.0, C22.1, C22.3, C22.4, C22.5, C22.7, C22.8, D13.4	155, 155.0, 155.1, 155.3, 155.5, 155.9, 211.5

Cancer registry (CR) data sources and are processed using some additional unique steps, which have been previously described,[9,28,29] and are summarized here. CR data processed for fatal estimation also serve as the inputs for estimation of nonfatal cancer burden, as described in section 7.

Most cancer registries only report cancer incidence. If, however, a cancer registry also reported cancer mortality, mortality data were also extracted. CR sources with matching incidence and mortality data were used to estimate mortality-to-incidence ratios. Cancer incidence and mortality data were sought from individual cancer registries, such as the Surveillance, Epidemiology, and End Results (SEER) Program[30]; provided by collaborators; or downloaded from aggregated databases of cancer registry data such as "Cancer Incidence In Five Continents" (CI5),"[31–41] EUREG,[42] or NORDCAN.[43] Only population-based cancer registries were included, with inclusion criteria that they included all cancers (ie, were not specialty registries), reported data for all age groups (except for paediatric cancer registries), and reported data for both sexes. Pathology-based cancer registries were included if they had a defined population. Hospital-based cancer registries were excluded. Redundant cancer registry data were excluded from either the final incidence data input or the MI model input if a more detailed source (eg, providing more detailed age or diagnostic groups) was available for the same population. Preference was given to registries with national coverage over those with only local coverage, except those from countries where the GBD study provides subnational estimates. Data were excluded if the coverage population was unknown. We used all data from GBD 2017[7] and added registry data from Argentina, Australia, Austria, Bermuda, Canada, Chile, China, Colombia, Germany, the Netherlands, Switzerland, the United Kingdom, Uruguay, and Yemen.[9]

These CR data are first transformed into standardised files. Second, subtotals are recalculated for registries that report individual and aggregated totals to verify the totals and subtract the values of any individual codes from the aggregates. Third, cancer registry mortality data and cancer registry incidence data are mapped to GBD causes. A different map is used for mortality and incidence because of the assumption that there are no deaths for certain cancers. Maps of ICD codes to GBD causes for mortality and incidence data can be found in Table 12 and Table 13.

Table 13: List of International Classification of Diseases (ICD) codes mapped to the Global Burden of Disease cause list for cancer incidence data

Cause	ICCC3	ICD10	ICD9
Liver cancer	VIIb, VIIc	C22, C22.0, C22.1, C22.3, C22.4, C22.5, C22.7, C22.8	155, 155.0, 155.1, 155.3, 155.5, 155.9

Fourth, data are standardised to GBD age groups and specific sexes. Global age-specific incidence rates are generated using all datasets that include microdata, and datasets that report age groups up to 95+ years of age. Age-specific mortality rates are generated using age weights from CoD data, as specified in Section 2.5 of Appendix 1 of the GBD 2019 Diseases and Injuries Capstone.[9] For incidence or mortality datasets that require age-splitting, age-specific proportions are then generated by applying the age-specific rates to the registry population to produce the expected number of cases (or deaths for a mortality dataset) for that registry by age. The expected number of cases (or deaths) for each sex, age, and cancer were normalised to 1, creating final, age-specific proportions. These proportions were then applied to the total number of cases (or deaths) by sex and cancer to get the GBD age-group-specific number of cases (or deaths) related to that dataset. In the rare case that the cancer registry only contains data for both sexes combined, the age-specific cases/deaths are split and reassigned to separate sexes using the same weights that are used for the age-splitting process.

In the fifth step, data for cause entries that are aggregates of GBD causes were redistributed across those GBD causes. In the sixth step, unspecified ICD codes ("garbage codes") such as "ill-defined cancer site" are redistributed across relevant causes estimated within the GBD hierarchy. Redistribution of cancer registry incidence and mortality data mirrored the process of the redistribution used in the cause of death database and utilised the same redistribution maps as specified in Section 2.4 of Appendix 1 to the GBD 2019 Diseases and Injuries Capstone.[9]

In GBD 2019, we changed ICD code mapping of C22.9 ("Malignant neoplasm of liver not specified as primary or secondary") to no longer directly map to liver cancer. Instead, we redistributed these codes to liver cancer, as well as other cancers that metastasise to the liver. This mapping change greatly reduced the number of deaths distributed to primary liver cancer. The Sankey diagram below shows the target causes of the redistribution method (Figure 18). See Section 12 for a summary of estimate changes between GBD 2017 and GBD 2019.

Figure 18: Sankey diagram illustrating redistribution of ICD code 22.9 onto various cancers

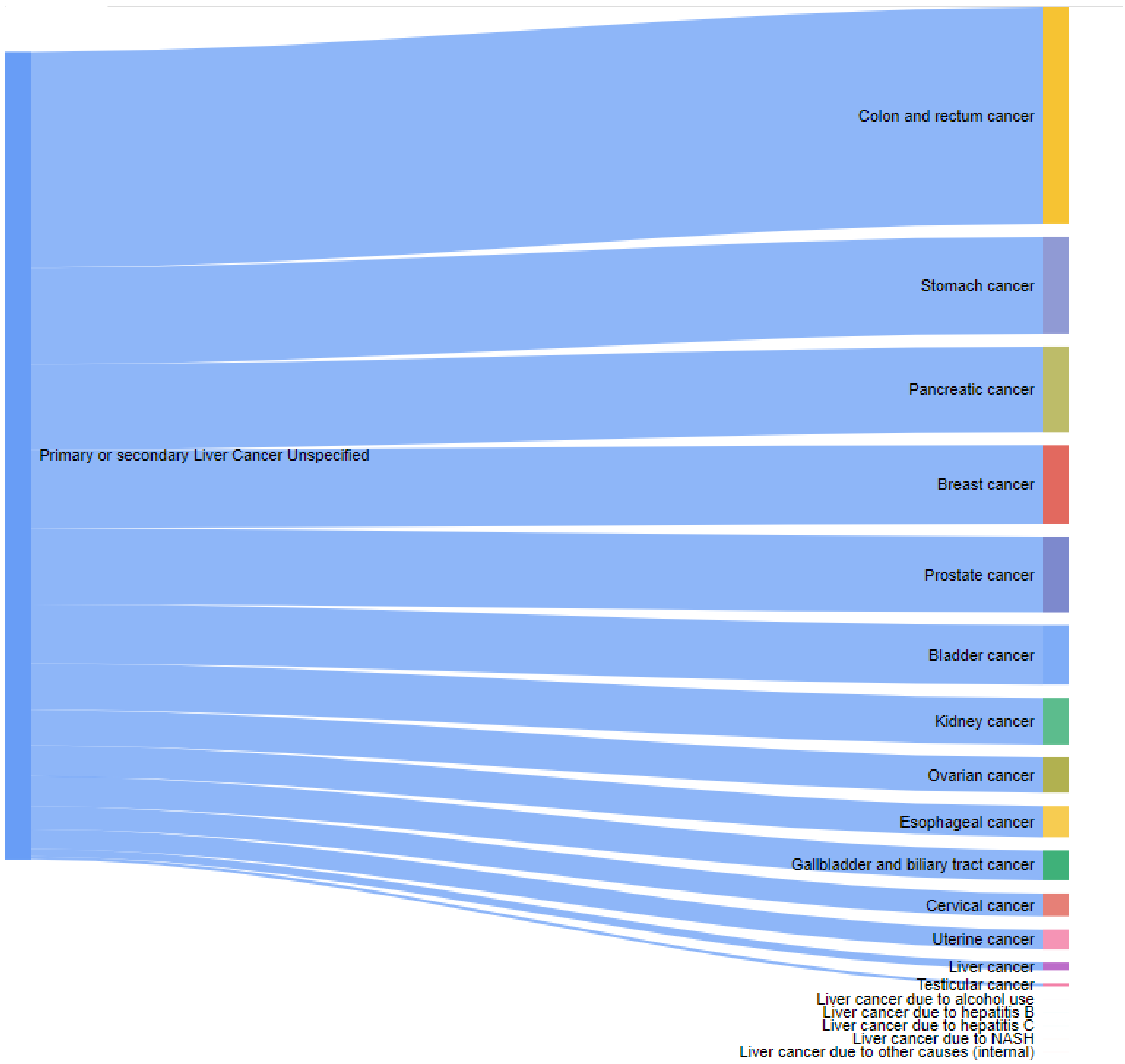

In the seventh step, duplicate or redundant data sources were removed from the processed cancer registry dataset. Duplicate sources were present if, for example, a cancer registry was part of the CI5 database but we also had data from that registry directly. Redundancies occurred and were removed where more detailed data were available, or when national registry data could replace regionally representative data. From here, two parallel selection processes were run: one to generate input data for the mortality-to-incidence ratio (MIR) models, and one to generate incidence for final mortality estimation. When creating the final incidence input, higher priority was given to registry data from the most standardised source, whereas for the MIR model input, only sources that reported both incidence and mortality were used.

In the eighth step, the processed incidence and mortality data from cancer registries were matched by cancer cause, age, sex, year, and location to generate MI ratios. MIRs from locations in HAQ quintiles 1-4 were dropped if they were below the median of MI ratios from locations in HAQ quintile 5. We also dropped MI ratios from locations in HAQ quintiles 1-4 if the MI ratios were above an outlier threshold calculated as the third quartile + 1.5 * IQR (inter-quartile range). We dropped all MIR data that were based on fewer than 15 incident cases to avoid excessive variation in the ratio due to small numbers.

The MI ratios were used as inputs for a three-step modelling approach using the general GBD spatiotemporal Gaussian process regression (ST-GPR)[11] approach, with the Healthcare Access and Quality Index (HAQi)[9,13] as a covariate in the linear step mixed effects model using a logit link function.[13]

$$\text{logit}\left(MI\ ratio_{c,a,s,t}\right) = \alpha + \beta_1 HAQI_{c,t} + \sum_{a}^{A} \beta_2 I_a + \beta_3 I_s + \epsilon_{c,a,s,t}$$

c: country, a: age group, t: time (years); s: sex, HAQI: Healthcare Access and Quality index, I: indicator variable, ϵc,a,s,t: error term

Predictions were made without the random effects.

The ST-GPR model has three main hyper-parameters that control for smoothing across time, age, and geography.[11] These hyper-parameters were adjusted for GBD 2019 in order to improve model performance in locations with sparse data. The time adjustment parameter lambda (λ) aims to borrow strength from neighbouring time points (ie, the value in this year is highly correlated with the value in the previous year but less so further back in time). For GBD 2019, lambda was lowered from 2 to 0.05, increasing the weight of more distant years. The age adjustment parameter omega (ω) borrows strength from data in neighbouring age groups and was lowered from 1.0 to 0.5, increasing the weight of more distant age groups. The space adjustment parameter zeta (ξ) aims to borrow strength across the hierarchy of geographical locations. Zeta was lowered from 0.95 to 0.01, reducing the weight of more distant geographical data at the region or super-region level. For the remaining parameters in the Gaussian process regression, we lowered the amplitude from 2 to 1 (reducing fluctuation from the mean function) and reduced the scale value from 15 to 10 (reducing the time distance over which points are correlated). Compared to GBD 2017 models, these model specification changes generally led to more smoothing of the MI ratio estimates across age and time, and less geographical smoothing at the region or super-region level. A description of the GBD ST-GPR modeling tool can be found in Appendix 1 of the GBD 2019 Risk Factor Capstone,[11] and more details on its application to cancer mortality modeling can be found in Appendix 1 of the GBD 2019 Diseases & Injuries Capstone[9] and Kocarnik et al.[29]

In the final CR data-processing step, we generated mortality estimates from the cleaned cancer registry incidence dataset and the MI ratios modeled as described above.

$$MIR_{estimates} * \ incidence_{registry} = mortality_{CR\ inputs}$$

These mortality estimates were then smoothed by a Bayesian noise-reduction algorithm (to deal with problems with zero counts, as also applied to the VR and VA data), as specified in Section

2.14 of Appendix 1 to the GBD 2019 Diseases and Injuries Capstone.[9] These data were uploaded into the COD database as CR data.

Figure 19 shows the geographic coverage of cancer registry, vital registration, and verbal autopsy data inputs for modelling liver cancer.

Figure 19: Mortality data inputs for the fatal model of liver cancer

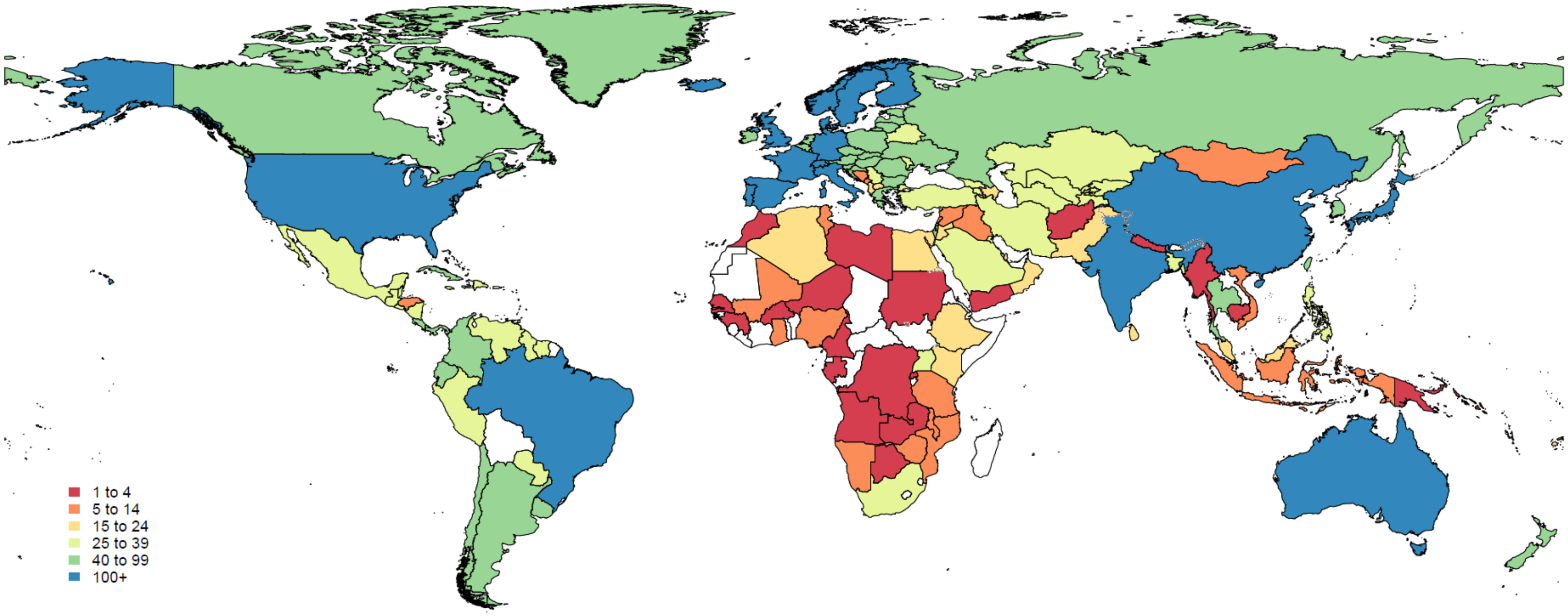

6.3 Modelling liver cancer mortality

Cancer-specific mortality modelling followed the general CODEm process using the totality of VA, VR, and CR data to generate estimates of location-age-year-sex liver cancer mortality. CODEm was described above in Section 5.3, and in greater detail in previous publications.[9,26] Table 14 reports the covariates, level, and specified direction tested in the liver cancer CODEm model.

Table 34: Covariates, level, and specified direction tested in liver cancer CODEm model

Level	Covariate	Direction
1	Litres of alcohol consumed per capita	+
	HIV age-standardised prevalence	+
	Vaccine-adjusted HBsAg seroprevalence age-standardised	+
	Hepatitis C seroprevalence (anti-HCV) age-standardised	+
	Log-transformed SEV scalar: liver cancer	+
2	Hepatitis B 3-dose vaccine coverage (proportion)	-
	Hepatitis B vaccine coverage proportion, aged through time	-
	Intravenous drug use (age-standarised proportion)	+
	Cumulative cigarettes (20 years)	+
	Mean BMI	+
	Tobacco (cigarettes per capita)	+
	Healthcare Access and Quality Index (HAQi)	-
	Diabetes fasting plasma glucose (mmol/L), age-standarised 25+	+
3	Education (years per capita)	-
	Age- and sex-specific SEV for high red meat	+
	LDI (I$ per capita)	-
	Socio-demographic Index (SDI)	-

The data inputs and modeling approaches used to estimate hepatitis C and HIV prevalence,[9] litres of alcohol consumed per capita, mean body mass index, intravenous drug use, summary exposure values (SEVs), cigarette and tobacco consumption, fasting plasma glucose,[11]

Healthcare Access and Quality Index (HAQi),[9,13] education,[27] lag-distributed income, and Socio-demographic index (SDI)[12] for GBD have been previously described. These processes result in estimates of these covariates for every year-age-sex-location combination in the GBD framework, and they are updated with each round of GBD.

6.4 Modelling aetiology-specific estimates

We applied scaled proportions of the five modelled aetiologies of liver cancer (hepatitis B, hepatitis C, alcohol, NAFLD, and other causes) to mortality estimates from CODEm to generate mortality estimates of liver cancer by aetiology. See sections 7.3 and 7.4 and previous publications[9,28] for descriptions of the data and models used to estimate aetiologic proportions for (primary) liver cancer.

7 Liver cancer non-fatal estimation

7.1 Flowchart

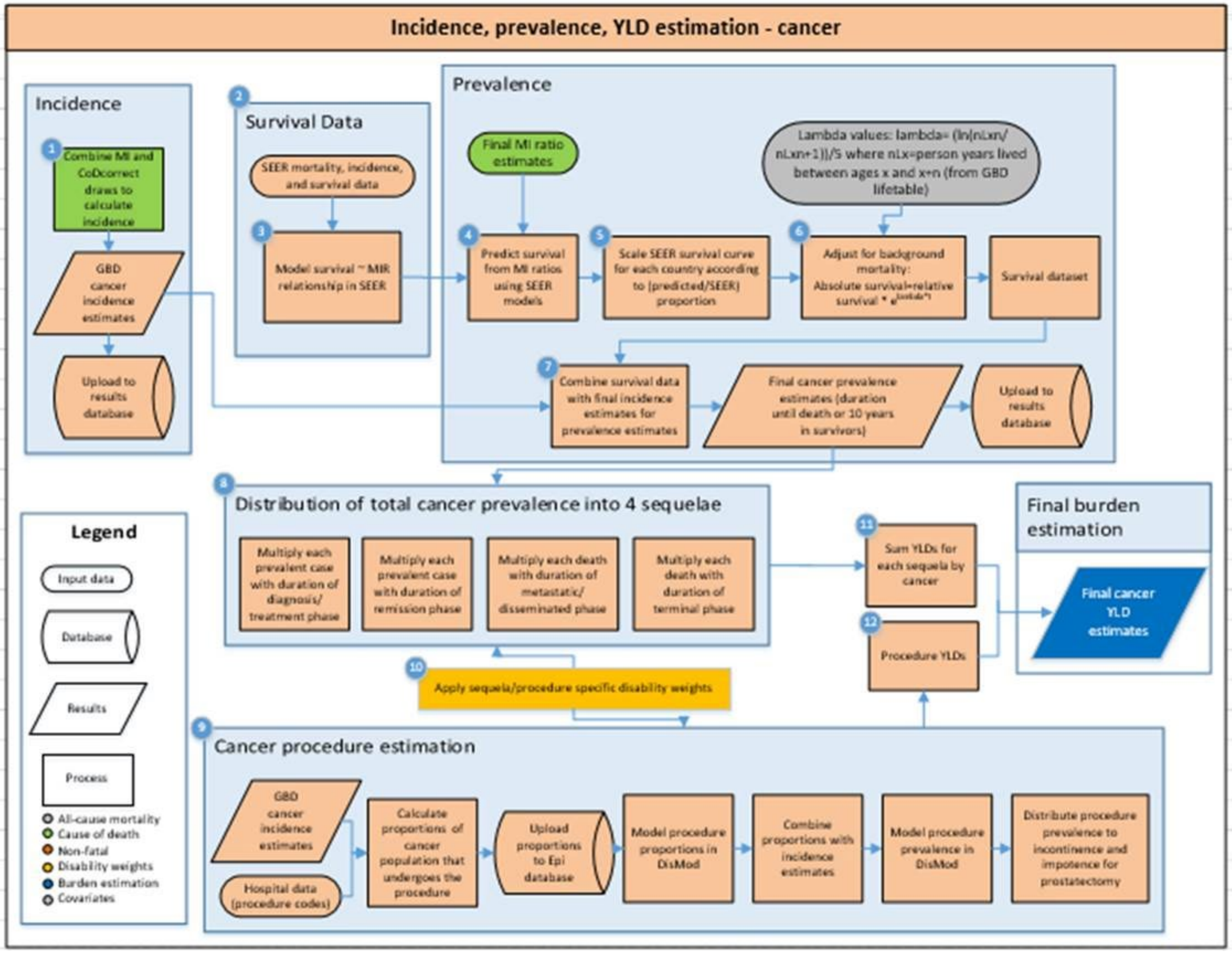

Figure 20: Flowchart GBD liver cancer incidence, prevalence, YLD estimation

7.2 Modelling liver cancer morbidity

Rather than employing data for one or more nonfatal epidemiologic parameters (such as incidence or prevalence) in a meta-regression model to estimate the incidence of a given cancer, estimates of cancer incidence in GBD are calculated from estimates of specific cancer mortality and MIRs,[9,28] as were described for (primary) liver cancer above in section 6. The final GBD liver cancer mortality estimates for every year-age-sex-location combination (after CoDCorrect adjustment) were divided by the modelled MIR estimates. As with other GBD calculations, this was done at the draw level. It was assumed that uncertainty in the MIRs is independent of uncertainty in the estimated mortality.

After transforming the final GBD cancer mortality estimates to incidence estimates (Figure 18), incidence was combined with annual relative survival estimates from 1 to 10 years after diagnosis. We estimated yearly cancer relative survival for each location as a function of HAQi and MIR. In GBD 2019, we updated these methods to estimate age-specific rather than all-ages survival curves.[9]

Previous reports suggest that the value of (1 – MIR) may serve as a proxy for 5-year relative survival, with the exact correlation varying slightly by cancer type.[44] Because this correlation varies, we trained cancer-specific prediction models to estimate 5-year survival from MIRs, using data from SEER.[30] We used SEER*Stat[45] to obtain mortality, incidence, and relative survival statistics from the nine SEER registries reporting from 1980 to 2014, by cancer type, sex, 5-year blocks (ie, 1980–84, 1985–89, etc.), and 5-year age groups (except combining 80+). For each cancer, we modelled SEER 5-year relative survival using MIRs calculated from SEER mortality and incidence. For GBD 2019, we updated this model from the Poisson regression used in GBD 2017[28] to using a generalised linear model with a quasi-binomial family and logit link, weighted by the number of index cases (step 3). These models were then applied to the GBD MIR estimates to predict an estimated 5-year survival for each age/sex/year/location. To prevent unrealistic values, predicted 5-year survival values were Winsorised to be between 0% and 100% survival.

To generate yearly survival estimates up to 10 years, in GBD 2019 we downloaded SEER sex- and age-specific annual 1- through 10-year relative survival data from persons diagnosed between 2001 and 2010.[46] A proportional scalar was calculated as the predicted GBD 5-year survival estimate divided by the SEER 5-year survival statistic, and was then used to generate yearly survival estimates by scaling the 1–10-year SEER curve to the GBD survival predictions under the proportional hazard assumption (step 5). This change from GBD 2017 (where we used SEER all-ages data from 2004 as the scalar and survival curve) impacts prevalence and YLD estimation, generally leading to survival estimates that are higher for younger ages and lower for older ages compared to estimates using the all-ages curve.

The estimated relative survival is next transformed into absolute survival estimates. To account for background mortality in the relative survival estimates, GBD 2019 life tables were used to calculate lambda values:

$$lambda = \frac{\ln\left(\frac{nLx_n}{nLx_{n+1}}\right)}{5}$$

nLx = person-years lived between ages x and x+n (from GBD life table).

Absolute survival was then calculated using an exponential survival function:

$$absolute\ survival = relative\ survival * e^{lambda*t}$$

t = time, in years

Absolute survival is combined with incidence to estimate the prevalence at each year 1 through 10 after diagnosis, which is then split into the four sequelae (step 8 in the flowchart). For the purposes of calculating disability due to cancer, survivors beyond 10 years were considered cured. For this group, the survivor population prevalence was divided into two sequelae: 1) diagnosis and primary therapy phase; and 2) controlled phase. For the population that did not survive beyond 10 years, the yearly prevalence was divided into the four sequelae by assigning the fixed durations for each of the diagnosis and primary therapy phase, metastatic phase, and terminal phase, and assigning the remaining prevalence to the controlled phase. Table 15 lists the duration of each phase.

Table 45: Duration of four prevalence phases[46]

Diagnosis/ treatment (months)	Remission (months)	Disseminated/ metastatic (months)	Terminal (months)
4	Calculated based on remainder of time after attributing other sequelae.	2.51	1

7.3 Aetiological proportion data

To find data to inform the proportions of (primary) liver cancer due to each of five aetiologies, a systematic literature search was performed in PubMed on 10/24/2016 using the following search string:

("liver neoplasms"[All Fields] OR "HCC"[All Fields] OR "liver cancer"[All Fields] OR "Carcinoma, Hepatocellular"[Mesh]) AND (("hepatitis B"[All Fields] OR "Hepatitis B"[Mesh] OR "Hepatitis B virus"[Mesh] OR "Hepatitis B Antibodies"[Mesh] OR "Hepatitis B Antigens"[Mesh]) OR ("hepatitis C"[All Fields] OR "Hepatitis C"[Mesh] OR "hepatitis C antibodies"[MESH] OR "Hepatitis C Antigens"[Mesh] OR "Hepacivirus"[Mesh]) OR ("alcohol"[All Fields] OR "Alcohol Drinking"[Mesh] OR "Alcohol-Related Disorders"[Mesh] OR "Alcoholism"[Mesh] OR "Alcohol-Induced Disorders"[Mesh])) NOT (animals[MeSH] NOT humans[MeSH])".

Studies were included if they reported on the aetiologies of a sample of liver cancer cases that were representative of the liver cancer population. Several studies not initially found through this

search but that were included in the meta-analysis by de Martel et al, were added.[47] We also included the study by Hong et al, after the authors provided us with additional data on the overlap in aetiologies.[48] Our systematic review of aetiologic proportions was not updated for GBD 2019. A comprehensive updated systematic review is planned for a future round of GBD.

For each study, the proportions of liver cancer due to the five specific aetiologies were calculated. Cases were considered to be due to NASH when the manuscript explicitly listed the aetiology to be NASH or non-alcoholic fatty liver disease (NAFLD). Cases where the aetiology was listed as "cryptogenic," "idiopathic," or "unknown" were included within the "other causes" category. In manuscripts where the aetiology for a case was not known but major categories could not be ruled out (for example, if the study tested for hepatitis B and C, but did not assess alcohol use), only the explicitly defined proportions were included (in this example, including proportions for hepatitis B and C, and excluding any remainder). Remaining aetiologies were included under a combined "other" group (for example, hemochromatosis, autoimmune hepatitis, Wilson's disease, etc.). If multiple aetiologies were reported for an individual patient, these were apportioned proportionally to the individual aetiologies (similar to the strategy reported for cirrhosis in section 4.6).

Figure 21: Data inputs for the liver cancer due to hepatitis B aetiological proportion model

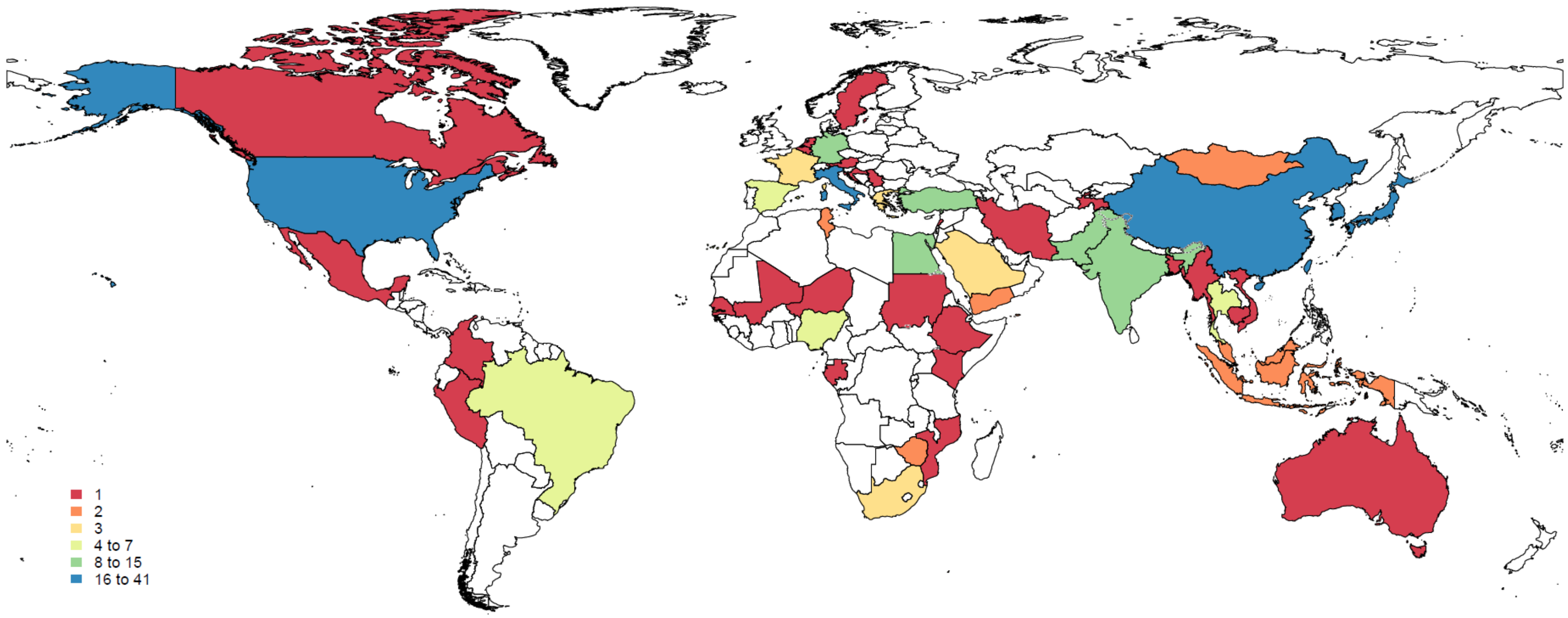

7.4 Modelling proportion of liver cancer due to hepatitis B

We used the proportion of liver cancer due to hepatitis B data to generate proportion estimates in a single-parameter model in DisMod-MR 2.1. As mentioned in section 4.10, and in contrast to the compartmental framework of many DisMod models, a single-parameter model uses only one data type to estimate one parameter, in this case proportion values. We included predictive covariates in the model to improve estimation for areas where data were scant or absent. Table 16 summarises covariates used in the single-parameter proportion model.

Table 56: Covariates used in the proportion of liver cancer due to hepatitis B DisMod-MR model

Covariate	Prior (min, max)	Exponentiated beta (95% UI)
Hepatitis B seroprevalence (HBsAg) age-standardised	(0,1)	1.90 (1.25–2.63)
Proportion of cirrhosis due to hepatitis B	(0,1)	1.54 (1.05–2.39)
Hepatitis B 3-dose coverage (proportion), lagged 10 years	(-1,0)	0.91 (0.82–0.99)

Separate single-parameter DisMod models were run to estimate the proportion of liver cancer due to the four specified aetiologies other than hepatitis B and the results of the five proportion models were re-scaled at the draw level for each year, age, sex and location to produce final proportion estimates. Models for aetiologies other than hepatitis B have been previously described.[9,28]

7.5 Modelling aetiology-specific estimates

We applied scaled proportions of the five modelled aetiologies of liver cancer (hepatitis B, hepatitis C, alcohol use, NAFLD/NASH, and other causes, as described in 7.3 and 7.4) to prevalence and incidence estimates to generate estimates of liver cancer by aetiology.

7.6 Disability weights

We generated estimates of years lived with disability by multiplying aetiology-specific prevalence estimates by their respective disability weights, as shown in Table 17. For a description of disability weight estimation, please see section 4.13 and previous GBD publications[11,19,20,24]

Table 17: Disability weights for liver cancer

Health state	Lay description	Disability weight (95% uncertainty interval)
Cancer, diagnosis and primary therapy	This person has pain, nausea, fatigue, weight loss, and high anxiety.	0.288 (0.193–0.399)
Cancer, controlled phase	This person has a chronic disease that requires medication every day and causes some worry but minimal interference with daily activities.	0.049 (0.031–0.072)
Cancer, metastatic	This person has severe pain, extreme fatigue, weight loss, and high anxiety.	0.451 (0.307–0.600)
Terminal phase, with medication	This person has lost a lot of weight and regularly uses strong medication to avoid constant pain. The person has no appetite, feels nauseous, and needs to spend most of the day in bed.	0.540 (0.377–0.687)

8 Acute hepatitis B non-fatal estimation

8.1 Flowchart

See Figure 1 (section 3.1) for flowchart on acute hepatitis B non-fatal estimation.

8.2 Modelling acute hepatitis B morbidity

We used the final estimates of HBsAg seroincidence (see Section 3) to generate estimates of total incidence of hepatitis B infection by dividing age-specific estimates of seroincidence by age-specific estimates of the probability of infection resulting in carriage based on Edmunds et al (Figure 22).[49] Edmunds et al only reports this probability for ages 0–25 years, so we assumed the probability for ages 25+ is the same as estimates for age 25.

$$P(carrier \mid age \leq 6\ months) = 0.885$$
$$P(carrier \mid 6\ months \leq age < 25\ years) = e^{-0.645 \times age^{0.455}}$$
$$P(carrier \mid age \geq 25\ years) = e^{-0.645 \times 25^{0.455}} = 0.061$$

Figure 22: Probability of infection resulting in chronic carriage from Edmunds et al[49]

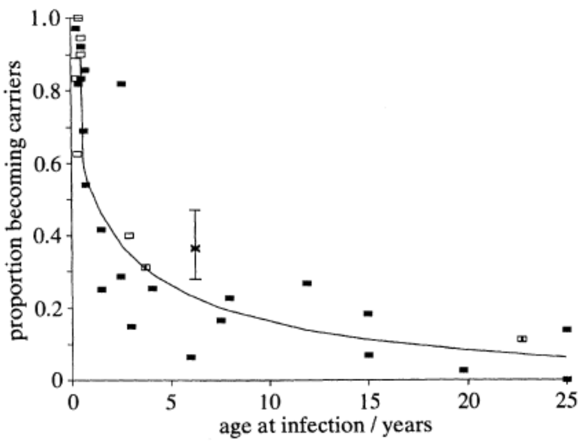

We then assigned cases of acute hepatitis B infection to one of three states: severe, moderate, or asymptomatic. To do so, we first estimated the incidence of symptomatic acute infections by multiplying the incident infection rate calculated above by the probability of acute symptomatic illness. We used estimates of this probability by age, for both sexes, from McMahon et al.[50] We assumed symptomatic perinatal cases are rare and used a probability of 1%, rather than the estimate from McMahon for age 0 (Figure 23).

Figure 23: Probability of symptomatic acute infection by age

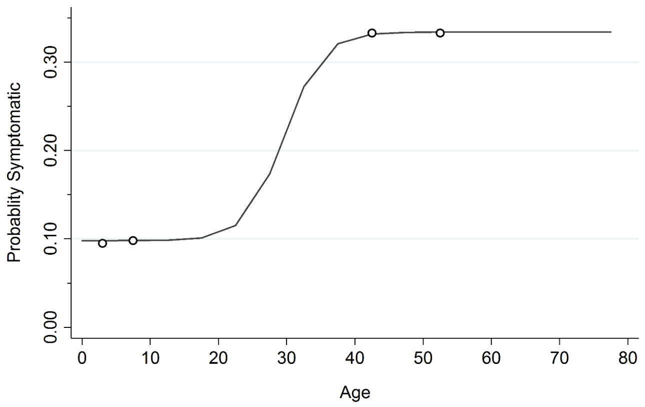

We used cases from McMahon et al[50] to calculate the probability an acute infection is severe, sampling from a beta distribution (Table 18). We assigned the remainder of acute symptomatic infections to the moderate state. We calculated asymptomatic infections as the difference between total acute infections and acute symptomatic infections. We assume duration of acute infections to be six weeks based on consultation with subject matter experts.

Table 18: Severity distribution of acute hepatitis B infection

0 percentile	25 percentile	50 percentile	75 percentile	100 percentile
0.032	0.2	0.26	0.32	0.62

8.3 Disability weights

Table 19 lists the disability weights associated with acute hepatitis B sequelae.[19,20] We do not assign disability to asymptomatic acute hepatitis B infections. For a description of disability weight estimation, please see section 4.13 and previous GBD publications[11,19,20,24]

Table 196: Disability weights for acute hepatitis B

Sequela	Description	Disability weight (95% uncertainty interval)
Moderate	Has a fever and aches, and feels weak, which causes some difficulty with daily activities.	0.051 (0.032–0.074)
Severe	Has a high fever and pain, and feels very weak, which causes great difficulty with daily activities.	0.133 (0.088–0.19)
Asymptomatic	Infection with no apparent illness	NA

9 Acute hepatitis B fatal estimation

9.1 Flowchart

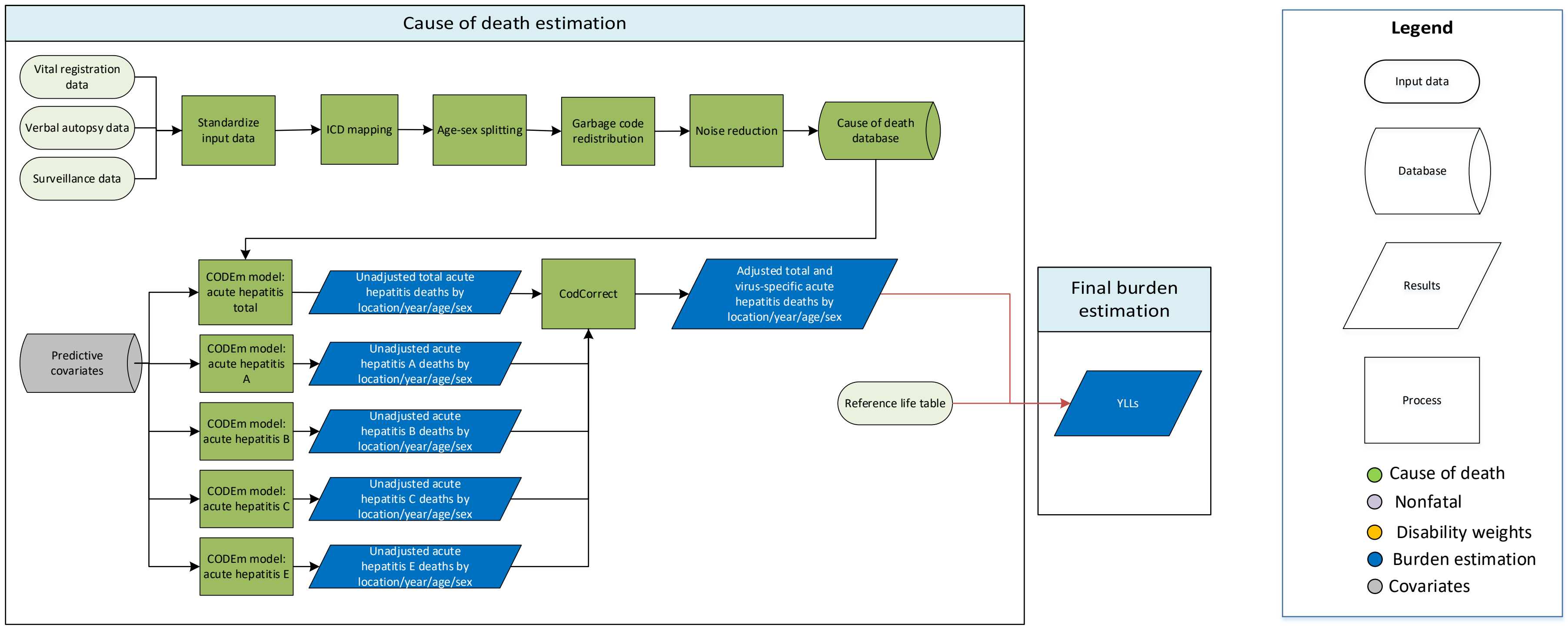

Figure 24: GBD acute hepatitis mortality, YLL estimation

9.2 Data identification and processing

We used vital registration (VR) and verbal autopsy (VA) data to model the acute (viral) hepatitis of any type (also called the "parent model") and high-quality VR data to model acute hepatitis B. The details of processing cause of death (COD) data for GBD, including mapping, disaggregation, age-sex splitting, corrections for misclassification, redistribution of garbage codes, noise reduction, and quality ratings have been previously described.[9,25] The ICD codes that map directly to total acute hepatitis and acute hepatitis B specifically are listed in Table 20.

Table 70: List of International Classification of Diseases (ICD) codes mapped to the Global Burden of Disease cause list for acute hepatitis parent and acute hepatitis B mortality data

Cause	ICD system	ICD codes
Acute hepatitis parent	10	B15, B16, B17.0, B17.2, B19.1, B19.10, B19.11, P35.3
	9	070.0, 070.1, 070.2, 070.20, 070.21, 070.42, 070.43, 070.52, 070.53
Acute hepatitis B	10	B16, B19.1, B19.1, B19.11, P35.3
	9	070.2, 070.20, 070.21, 070.3, 070.30, 070.31, 070.42, 070.52

An important component of mortality estimation is redistribution of vague, impossible, intermediate or immediate causes of death to valid underlying causes of death in the GBD cause list.[9,25] In GBD 2019, we investigated the subset of our data from vital registration systems that allow recording multiple diagnostic codes as causes of death (underlying, intermediate, etc) and found that where a code for hepatitis that did not specify acuity was assigned as the underlying cause of death, ICD codes for chronic liver disease often appeared in the cause of death chain. Based on this analysis, a proportion of hepatitis, unspecified deaths were redistributed to death due to cirrhosis and other chronic liver diseases, and a small proportion to acute hepatitis, as illustrated above in Figure 24. Prior to GBD 2019, all of these deaths had been mapped directly to acute hepatitis.

We marked data as outliers and excluded them in instances where garbage code redistribution and noise reduction, in combination with small sample sizes, resulted in unreasonable cause fractions or unreasonable time, age, or spatial trends.

Figure 25: Mortality data inputs for the parent acute hepatitis

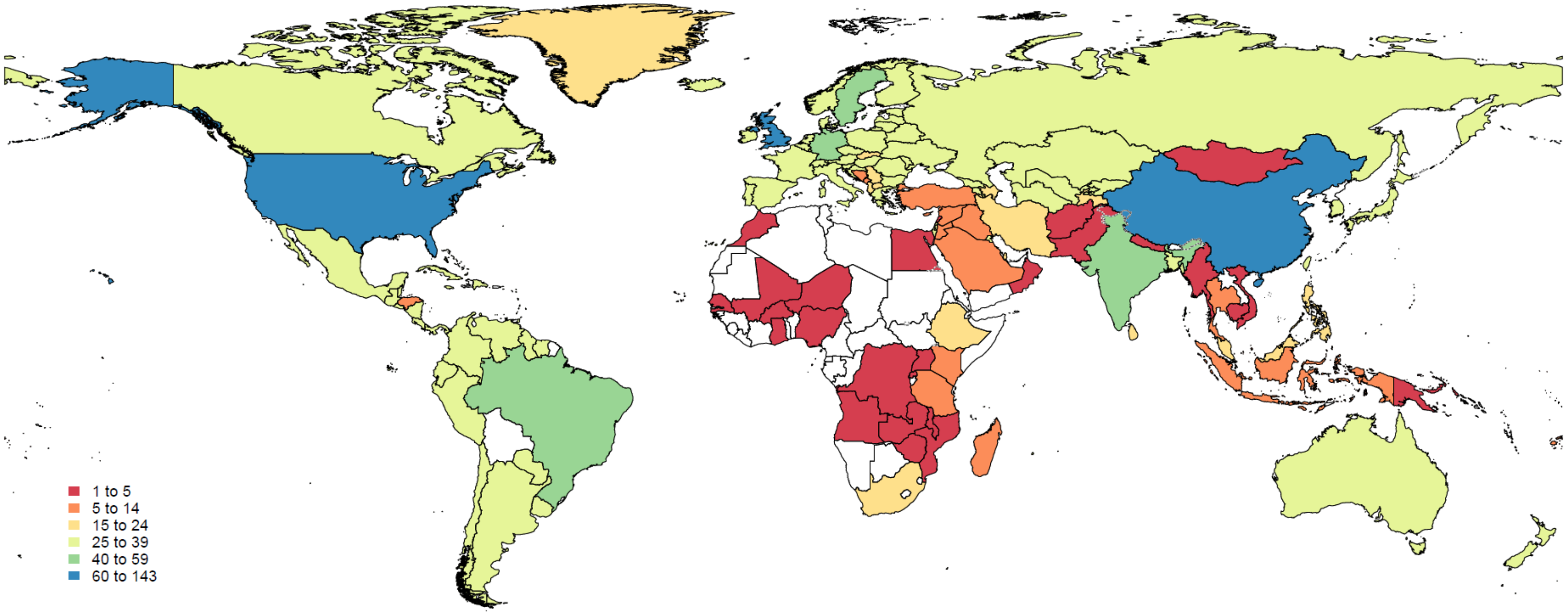

Figure 26: Mortality data inputs for the acute hepatitis B

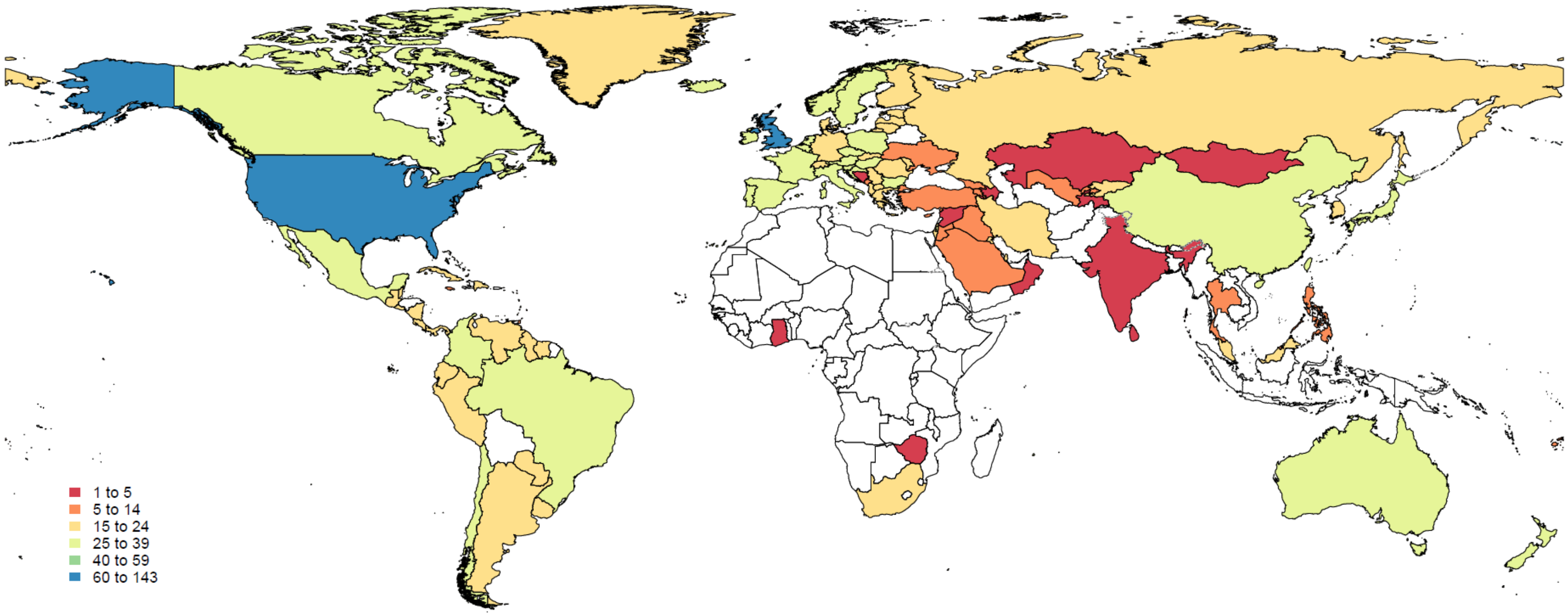

9.3 Modelling acute hepatitis parent mortality

Acute hepatitis parent mortality was modeled in CODEm using VR and VA data to generate estimates specific to year, age, sex and location. CODEm was described in more detail in Section 5.3 and previous publications.[9,26] Table 21 reports the covariates, level, and specified direction tested in the acute hepatitis parent CODEm model.

Table 81: Covariates, level, and specified direction tested in parent acute hepatitis CODEm model

Level	Covariate	Direction
1	SEV scalar age-standardised hepatitis	+
	Seroprevalence (HBsAg) age-standardised	+
	Seroprevalence (anti-HCV) age-standardised	+
	Seroprevalence (anti-HAV) age-standardised	+
	Seroprevalence (anti-HEV) age-standardised	+
2	Healthcare Access and Quality Index (HAQi)	-
	SEV unsafe sanitation	+
	SEV unsafe water	+
	Socio-demographic Index (SDI)	-
	Hep B vaccine coverage proportion, aged through time	-
	Injection drug use proportion by age	+
3	Education (years per capita)	-
	Lag distributed income (LDI) (ln transformation)	-

The data inputs and modeling approaches used to estimate seroprevalence of anti-HCV, anti-HAV and anti-HEV,[9] Healthcare Access and Quality Index (HAQi),[9,13] summary exposure values (SEVs) and injection drug use,[11] Socio-demographic index (SDI),[12] education,[27] and lag-distributed income[12] for GBD have been previously described. These processes result in estimates of these covariates for every year-age-sex-location combination in the GBD framework, and they are updated with each round of GBD.

9.4 Modelling acute hepatitis B mortality

Acute hepatitis B mortality was modeled in CODEm using high quality VR data to generate estimates specific to year, age, sex and location. CODEm was described in more detail in Section 5.3 and previous publications.[9,26] Table 22 reports the covariates, level, and specified direction tested in the acute hepatitis B CODEm model.

After modeling acute hepatitis deaths encompassing all hepatitis virus types (A, B, C, and E) in the parent CODEm model, and also modeling acute hepatitis A, B, C, and E in separate CODEm models, the virus-specific acute hepatitis deaths were rescaled to fit within the envelope defined by the parent through the CoDCorrect process.

Table 92: Covariates, level, and specified direction tested in acute hepatitis B CODEm model

Level	Covariate	Direction
1	SEV scalar (hepatitis)	+
	Seroprevalence (HBsAg) age-standardised	+
2	Healthcare Access and Quality Index (HAQi)	-
	Socio-demographic Index (SDI)	-
	Hep B vaccine coverage proportion, aged through time	-
	Injection drug use proportion by age	+
3	Education (years per capita)	-
	Lag distributed income (LDI) (ln transformation)	-

The data inputs and modeling approaches used to estimate summary exposure values (SEVs),[11] Healthcare Access and Quality Index (HAQi),[9,13] Socio-demographic index (SDI),[12] injection drug use,[11] education,[27] and lag-distributed income[12] for GBD have been previously described. These processes result in estimates of these covariates for every year-age-sex-location combination in the GBD framework, and they are updated with each round of GBD.

10 Hepatitis B 3-dose primary series vaccine coverage estimation

10.1 HepB3 vaccine data inputs

The hepatitis B 3 dose primary series (HepB3) vaccination coverage estimation process is described in detail in a separate report.[14] Input datasources were found by searching the Global Health Data Exchange (GHDx) with the keywords Hepatitis B vaccines OR Hepatitis vaccines. Sources include survey microdata (for example, DHS, MICS, and smaller country-specific surveys where available), survey report data in the absence of available microdata, and bias-adjusted official country-reported data obtained from the joint reporting form (JRF) collated by WHO and UNICEF.

10.2 Modelling HepB3 vaccine coverage

Estimates of HepB3 coverage capture the proportion of 0–11-month-old children who received the complete three-dose series by location and year. Given the relatively recent introduction of HepB3 compared to the diphtheria-tetanus-pertussis (DTP) series, we modelled HepB3 coverage as a ratio against DTP3 to better capture vaccine scale-up. This ratio was constrained so that HepB3 coverage never exceeded DTP3 coverage in any location-year. Vaccine introduction years were country-specific, aligning with country reports from the 2020 JRF. We ran spatiotemporal Gaussian progress regression (ST-GPR) models[11] to produce estimates of HepB3 coverage correlated over space and time. To inform coverage estimates in locations and years with sparse data, we included HAQi and the mortality rate due to events of war as covariates in the model, in addition to a custom covariate estimating the magnitude of disruption due to country-reported HepB3 delivery disruptions. After modelling the scale-up in ST-GPR, ratios were multiplied by final DTP3 estimates to calculate HepB3 coverage.

11 Additional analyses

11.1 Target achievement

All country estimates at the draw level were compared with the goals put forth in the World Health Organization-Global Health Sector Strategy (WHO-GHSS) 2020 Goals[51] and the World Health Organization's Interim Guidance for Country Validation of Viral Hepatitis Elimination (WHO Interim Guidance)[52] to assess the posterior probability of achieving targets. Countries were determined to have a high probability (95% or greater) of achieving a particular goal if 950 or more draws out of 1000 draws met or exceeded the proposed goal.

11.2 Annualised rate of change

Annualised rate of change (ARC) is the percentage change per year in a given time interval:

$$ARC = \frac{\ln\left(\frac{X\_y_2}{X\_y_1}\right)}{y_2 - y_1}$$

X_y_n = rate value at year $\_y_n$;y_1 = starting year (e.g. 2015); y_2 = ending year (e.g. 2019)

ARC was calculated between 2015 and 2019 to assess progress since the adoption of the WHO-GHSS. We assessed what the rate of change would need to be from 2019 to 2030 in order to meet WHO-GHSS 2030 targets. Final HBV-related mortality estimates from 2015 were multiplied by 0.35 (1 – 0.65, ie, the mortality target) to get what the HBV-related mortality estimates would need to be in 2030 to achieve the WHO-GHSS 2030 target. Final HBsAg prevalence under 5 estimates from 2015 were multiplied by 0.05 (1 – 0.95, ie, the new cases target) to get what the HBsAg estimates would need to be in 2030 to achieve the WHO-GHSS 2030 target. ARC was calculated between 2019 and 2030 estimates to determine what the rate of change needed to achieve goals would need to be. We also assessed what ARC would need be from 2019 to achieve the absolute mortality rate target of less than 4 per 100,000 in 2030, as put forth in recent WHO Interim Guidance. ARC was calculated by dividing 4 per 100,000 (i.e. the absolute mortality rate target) by the final HBV-related mortality estimates from 2019, and again by the year gap between 2019 and 2030.

12 Summary of changes from GBD 2017

Several important data and methods changes happened in GBD 2019 that led to differences from estimates produced in GBD 2017. Table 23 summarises the estimates between GBD studies.

Table 103: Summary of global death estimates for GBD 2017 and GBD 2019

**Cause**	**GBD 2017** (% of parent)	**GBD 2019** (% of parent)	**Percentage change between GBD cycles**
Total cirrhosis deaths	1,323,000	1,430,000	8.1%
Cirrhosis due to hepatitis B deaths	384,000 (29%)	321,000 (22%)	–16.4%
Total liver cancer deaths	819,000	461,000	–43.7%
Liver cancer due to hepatitis B	325,000 (40%)	184,000 (40%)	–43.4%
Total acute (viral) hepatitis deaths	126,000	82,500	–34.5%
Acute hepatitis B deaths	89,600 (71%)	32,700 (40%)	–63.5%
Total HBV-related deaths	799,000	538,000	–32.7%

12.1 HBsAg changes

The HBsAg prevalence estimates generally decreased between GBD 2017 and GBD 2019. In GBD 2017, the DisMod-MR 2.1 model estimating HBsAg seroprevalence used all available data; however, it tended to follow data from unvaccinated populations and poorly fit prevalence data from vaccinated populations at younger changes. DisMod-MR 2.1 assumes that diseases are steady-state; however, vaccine uptake and cohort effects have led to rapid changes in seroprevalence. In GBD 2019, we changed the modelling strategy to a counterfactual model to estimate what seroprevalence would be in the absence of vaccination efforts. We excluded seroprevalence data in age groups and years where vaccination had been introduced prior to modelling the counterfactual scenario in DisMod-MR 2.1. We then performed a post-hoc adjustment based on GBD-produced location-year-specific hepatitis B 3-dose vaccine coverage and an assumed vaccine efficacy. The proportion of coverage by location and year were multiplied by vaccine efficacy of 95% to get the proportion of the population that completed the hepatitis B 3-dose vaccine series in infancy. Then these results were subtracted from the HBsAg seroprevalence DisMod estimates to get final estimates of HBsAg prevalence and incidence. Smaller changes to HBsAg prevalence estimation resulted from the addition of more seroprevalence data, largely added from our comparison of sources with Schweitzer et al.[3]

12.2 Cirrhosis changes

There was a slight increase in overall cirrhosis mortality estimates between the estimation cycles. In GBD 2017 and earlier rounds, ICD codes for hepatitis that did not specify acuity were mapped to acute (viral) hepatitis. In GBD 2019, we reviewed vital registration data sources that provide

information on multiple causes of death (underlying, intermediate, and immediate, as well as predisposing conditions), and noted that many "unspecified hepatitis" deaths included chronic liver diseases in the cause of death chain. Based on these analyses, those deaths were proportionately redistributed to cirrhosis and other chronic liver diseases and acute hepatitis, with the majority assigned to the former. Details of the GBD multiple cause of death (MCOD) analysis have been previously published.[9] Smaller changes in cirrhosis mortality estimation were seen due to the addition of new VR and VA data sources.

Cirrhosis deaths due to hepatitis B, however, decreased from GBD 2017, due to additional case-series data sources to improve estimation of the proportion of cirrhosis deaths due to hepatitis B. Our model of the proportion of cirrhosis deaths attributable to hepatitis B was also influenced by decreases in our estimate of HBsAg seroprevalence, as this is employed as covariatein our aetiologic proportion model (as described above in section 4.10).

12.3 Liver cancer changes

There was a large decline in overall liver cancer deaths between GBD 2017 and GBD 2019. In GBD 2017, all deaths with ICD-10 code C22.9 ("Malignant neoplasms of liver not specific as primary or secondary") were mapped to liver cancer. This code, however, does not distinguish between deaths due to cancer from other primary locations that has metastasized to liver and deaths due to primary liver cancer. In GBD 2019, these deaths were instead mapped as garbage codes and redistributed to liver and 13 other cancers (colon and rectum, pancreas, prostate, breast, oesophagus, bladder, kidney, stomach, ovary, uterus, cervix, gallbladder, and testes), each according to the relative occurrence in the dataset of specific ICD codes for these primary cancers.

12.4 Acute hepatitis B changes

There was a large decline in acute hepatitis B deaths between GBD 2017 and GBD 2019. In GBD 2017 and earlier rounds, we estimated deaths due to acute hepatitis A, B, C, and E in natural history models that used incidence estimates from non-fatal hepatitis A, B, C, and E models and case-fatality ratios from hospital data.[2] The older approach relied on the assumption that case-fatality ratios in hospital data could be applied to all acute hepatitis cases in the community, and employed hospital data from fewer than twenty locations. We modified the modelling strategy for acute hepatitis A, B, C, and E from this natural history model to a CODEm approach to employ direct measures of cause of death, avoid assumptions about case fatality, and better leverage data from more locations. As mentioned in section 12.2, we also redistributed unspecified hepatitis deaths to cirrhosis and acute hepatitis causes in the GBD map through a proportional approach after reviewing codes in multiple causes of death chain in vital registration data.

13 Geographical hierarchies

Table 114. WHO member states by region

Region name	Locations
**African Region**	Algeria, Angola, Benin, Botswana, Burkina Faso, Burundi, Cape Verde, Cameroon, Central African Republic, Chad, Comoros, Congo (Brazzaville), Côte d'Ivoire, DR Congo, Equatorial Guinea, Eritrea, Eswatini, Ethiopia, Gabon, The Gambia, Ghana, Guinea, Guinea-Bissau, Kenya, Lesotho, Liberia, Madagascar, Malawi, Mali, Mauritania, Mauritius, Mozambique, Namibia, Niger, Nigeria, Rwanda, São Tomé and Príncipe, Senegal, Seychelles, Sierra Leone, South Africa, South Sudan, Togo, Uganda, Tanzania, Zambia, Zimbabwe
**Eastern Mediterranean Region**	Afghanistan, Bahrain, Djibouti, Egypt, Iran, Iraq, Jordan, Kuwait, Lebanon, Libya, Morocco, Oman, Pakistan, Qatar, Saudi Arabia, Somalia, Sudan, Syria, Tunisia, United Arab Emirates, Yemen
**European Region**	Albania, Andorra, Armenia, Austria, Azerbaijan, Belarus, Belgium, Bosnia and Herzegovina, Bulgaria, Croatia, Cyprus, Czech Republic, Denmark, Estonia, Finland, France, Georgia, Germany, Greece, Hungary, Iceland, Ireland, Israel, Italy, Kazakhstan, Kyrgyzstan, Latvia, Lithuania, Luxembourg, Malta, Monaco, Montenegro, Netherlands, North Macedonia, Norway, Poland, Portugal, Moldova, Romania, Russia, San Marino, Serbia, Slovakia, Slovenia, Spain, Sweden, Switzerland, Tajikistan, Turkey, Turkmenistan, Ukraine, UK, Uzbekistan
**Region of the Americas**	Antigua and Barbuda, Argentina, The Bahamas, Barbados, Belize, Bolivia, Brazil, Canada, Chile, Colombia, Costa Rica, Cuba, Dominica, Dominican Republic, Ecuador, El Salvador, Grenada, Guatemala, Guyana, Haiti, Honduras, Jamaica, Mexico, Nicaragua, Panama, Paraguay, Peru, Saint Kitts and Nevis, Saint Lucia, Saint Vincent and the Grenadines, Suriname, Trinidad and Tobago, USA, Uruguay, Venezuela
**South-East Asia Region**	Bangladesh, Bhutan, North Korea, India, Indonesia, Maldives, Myanmar, Nepal, Sri Lanka, Thailand, Timor-Leste
**Western Pacific Region**	Australia, Brunei, Cambodia, China, Cook Islands, Fiji, Japan, Kiribati, Laos, Malaysia, Marshall Islands, Federated States of Micronesia, Mongolia, Nauru, New Zealand, Niue, Palau, Papua New Guinea, Philippines, South Korea, Samoa, Singapore, Solomon Islands, Tonga, Tuvalu, Vanuatu, Vietnam

Table 125. Locations by Socio-demographic Index (SDI) quintiles

Region Name	Locations
**Low SDI**	Addis Ababa, Afar, Afghanistan, Amhara, Balochistan, Bauchi, Benin, Benishangul-Gumuz, Bihar, Rural, Borno, Burkina Faso, Burundi, Central African Republic, Chad, Comoros, Côte d'Ivoire, Democratic Republic of the Congo, Dire Dawa, Eritrea, Gambella, Gambia, Gilgit-Baltistan, Gombe, Guinea, Guinea-Bissau, Haiti, Harari,

	Jharkhand, Rural, Jigawa, Kano, Katsina, Kebbi, Khyber Pakhtunkhwa, Liberia, Madagascar, Madhya Pradesh, Rural, Malawi, Mali, Maranhao, Mozambique, Nepal, Niger, Niger, Oromia, Papua New Guinea, Rwanda, Senegal, Sierra Leone, Sindh, Sokoto, Solomon Islands, Somali, Somalia, South Sudan, Southern Nations, Nationalities, and Peoples, Tanzania, Taraba, Tigray, Togo, Uganda, Yemen, Yobe, Zamfara
**Low-middle SDI**	Acre, Adamawa, Alagoas, Amazonas, Andhra Pradesh, Rural, Angola, Arunachal Pradesh, Rural, Assam, Rural, Azad Jammu & Kashmir, Bahia, Bangladesh, Baringo, Belize, Benue, Bhutan, Bihar, Urban, Bolivia, Bomet, Bungoma, Busia, Cabo Verde, Cambodia, Cameroon, Ceara, Chhattisgarh, Rural, Congo, Cross River, North Korea, Djibouti, Dominican Republic, East Nusa Tenggara, Ebonyi, El Salvador, Elgeyo-Marakwet, Embu, Eswatini, Gansu, Garissa, Ghana, Gorontalo, Guatemala, Guizhou, Gujarat, Rural, Haryana, Rural, HomaBay, Honduras, Isiolo, Jammu and Kashmir, Rural, Kaduna, Kajiado, Kakamega, Karnataka, Rural, Kericho, Kiambu, Kilifi, Kiribati, Kirinyaga, Kisii, Kisumu, Kitui, Kogi, Kwale, Kwara, Kyrgyzstan, Laikipia, Lamu, Laos, Lesotho, Machakos, Maharashtra, Rural, Makueni, Maldives, Maluku, Mandera, Manipur, Rural, Marsabit, Marshall Islands, Mauritania, Meghalaya, Rural, Meru, Federated States of Micronesia, Migori, Mizoram, Rural, Mombasa, Mongolia, Morocco, Murang'a, Myanmar, Nagaland, Rural, Nairobi, Nakuru, Nandi, Narok, Nasarawa, Nicaragua, North Maluku, Nyamira, Nyandarua, Nyeri, Odisha, Rural, Ogun, Palestine, Para, Paraiba, Pernambuco, Piaui, Plateau, Punjab, Punjab, Rural, Rajasthan, Rural, Rio Grande do Norte, Rondonia, Samburu, São Tomé and Príncipe, Sergipe, Siaya, Sikkim, Rural, Sudan, TaitaTaveta, Tajikistan, Tamil Nadu, Rural, TanaRiver, Telangana, Rural, TharakaNithi, Tibet, Timor-Leste, Tocantins, TransNzoia, Tripura, Rural, Turkana, Tuvalu, UasinGishu, Union Territories other than Delhi, Rural, Uttar Pradesh, Rural, Uttarakhand, Rural, Vanuatu, Venezuela, Vihiga, Wajir, West Bengal, Rural, West Kalimantan, West Nusa Tenggara, West Sulawesi, WestPokot, Yunnan, Zambia, Zimbabwe
**Middle SDI**	Abia, Abra, Aceh, Aguascalientes, Agusan Del Norte, Agusan Del Sur, Aklan, Akwa Ibom, Albania, Albay, Alborz, Algeria, Amapa, Anambra, Andhra Pradesh, Urban, Anhui, Antique, Apayao, Ardebil, Armenia, Arunachal Pradesh, Urban, Assam, Urban, Aurora, Azerbaijan, Baja California, Baja California Sur, Bali, Bangka-Belitung Islands, Banten, Basilan, Bataan, Batanes, Batangas, Bayelsa, Bengkulu, Benguet, Biliran, Bohol, Botswana, Bukidnon, Bulacan, Bushehr, Cagayan, Camarines Norte, Camarines Sur, Camiguin, Campeche, Capiz, Catanduanes, Cavite, Cebu, Central Java, Central Kalimantan, Central Sulawesi, Chahar Mahaal and Bakhtiari, Chhattisgarh, Urban, Chiapas, Chihuahua, Chongqing, Coahuila, Colima, Colombia, Compostela Valley, Costa Rica, Cotabato (North Cotabato), Cuba, Davao Del Norte, Davao Del Sur, Davao Occidental, Davao Oriental, Delhi, Rural, Delta, Dinagat Islands, Durango, East Azarbayejan, East Java, Eastern Cape, Eastern Samar, Ecuador, Edo, Egypt, Ekiti, Enugu, Equatorial Guinea, Espirito Santo, Fars, FCT (Abuja), Fiji, Free State, Fujian, Gabon, Gauteng, Gilan, Goa, Rural, Goias, Golestan, Grenada, Guanajuato, Guangxi, Guerrero, Guimaras, Gujarat, Urban, Guyana, Hainan, Hamadan, Haryana, Urban, Hebei, Heilongjiang, Henan, Hidalgo, Himachal Pradesh, Rural, Hormozgan, Hubei, Hunan, Ifugao, Ilam, Ilocos Norte, Ilocos Sur, Iloilo, Imo, Iraq, Isabela, Isfahan, Islamabad Capital Territory, Jalisco, Jamaica, Jambi, Jammu and Kashmir, Urban,

	Jharkhand, Urban, Jiangxi, Kalinga, Karnataka, Urban, Kerala, Rural, Kerala, Urban, Kerman, Kermanshah, Khorasan-e-Razavi, Khuzestan, Kohgiluyeh and Boyer-Ahmad, Kurdistan, KwaZulu-Natal, La Union, Laguna, Lampung, Lanao Del Norte, Lanao Del Sur, Leyte, Limpopo, Lorestan, Madhya Pradesh, Urban, Maguindanao, Manipur, Urban, Marinduque, Markazi, Masbate, Mato Grosso, Mato Grosso do Sul, Mazandaran, Meghalaya, Urban, Mexico, Mexico City, Michoacan de Ocampo, Minas Gerais, Misamis Occidental, Misamis Oriental, Mizoram, Urban, Morelos, Mountain Province, Mpumalanga, Nagaland, Urban, Namibia, National Capital Region, Nauru, Nayarit, Negros Occidental, Negros Oriental, Ningxia, North Khorasan, North Sulawesi, North Sumatra, Northern Cape, Northern Samar, North-West, Nueva Ecija, Nueva Vizcaya, Nuevo Leon, Oaxaca, Occidental Mindoro, Odisha, Urban, Ondo, Oriental Mindoro, Osun, Oyo, Palawan, Pampanga, Panama, Pangasinan, Papua, Paraguay, Parana, Peru, Puebla, Punjab, Urban, Qazvin, Qinghai, Qom, Queretaro, Quezon, Quintana Roo, Quirino, Rajasthan, Urban, Rio Grande do Sul, Rivers, Rizal, Romblon, Roraima, Saint Lucia, Saint Vincent and the Grenadines, Samar (Western Samar), Samoa, San Luis Potosi, Sarangani, Semnan, Shanxi, Sichuan, Sinaloa, Siquijor, Sistan and Baluchistan, Sonora, Sorsogon, South Cotabato, South Kalimantan, South Khorasan, South Sulawesi, South Sumatra, Southeast Sulawesi, Southern Leyte, Sultan Kudarat, Sulu, Surigao Del Norte, Surigao Del Sur, Suriname, Syrian Arab Republic, Tabasco, Tamaulipas, Tamil Nadu, Urban, Tarlac, Tawi-Tawi, Tehran, Telangana, Urban, Thailand, Tlaxcala, Tokelau, Tonga, Tripura, Urban, Tunisia, Turkmenistan, Uttar Pradesh, Urban, Uzbekistan, Veracruz de Ignacio de la Llave, Vietnam, West Azarbayejan, West Bengal, Urban, West Java, West Papua, West Sumatra, Western Cape, Xinjiang, Yazd, Yogyakarta, Yucatan, Zacatecas, Zambales, Zamboanga Del Norte, Zamboanga Del Sur, Zamboanga Sibugay, Zanjan
**High-middle SDI**	Abruzzo, Altai kray, American Samoa, Amur oblast, Antigua and Barbuda, Argentina, Arkhangelsk oblast without Nenets autonomous district, Astrakhan oblast, Bahamas, Bahrain, Barbados, Basilicata, Belarus, Belgorod oblast, Bosnia and Herzegovina, Bryansk oblast, Bulgaria, Calabria, Campania, Chechen Republic, Chelyabinsk oblast, Chile, Chukchi autonomous area, Chuvash Republic, Cook Islands, Croatia, Delhi, Urban, Distrito Federal, Dolnoslaskie, Dominica, East Kalimantan, Emilia-Romagna, Friuli-Venezia Giulia, Georgia, Goa, Urban, Greece, Greenland, Guangdong, Himachal Pradesh, Urban, Hungary, Inner Mongolia, Irkutsk oblast, Israel, Ivanovo oblast, Jakarta, Jewish autonomous oblast, Jiangsu, Jilin, Jordan, Kabardian-Balkar Republic, Kaliningrad oblast, Kaluga oblast, Kamchatka kray, Karachaev-Chercassian Republic, Kazakhstan, Kemerovo oblast, Khabarovsk kray, Khanty-Mansi autonomous area, Kirov oblast, Komi Republic, Kostroma oblast, Krasnodar kray, Krasnoyarsk kray, Kujawsko-Pomorskie, Kurgan oblast, Kursk oblast, Lagos, Lazio, Lebanon, Leningrad oblast, Liaoning, Libya, Liguria, Lipetzk oblast, Lodzkie, Lombardia, Lubelskie, Lubuskie, Magadan oblast, Maharashtra, Urban, Malaysia, Malopolskie, Malta, Marche, Mauritius, Mazowieckie, Mississippi, Molise, Montenegro, Moscow City, Moscow oblast, Murmansk oblast, Nenets autonomous district, Niue, Nizhny Novgorod oblast, North Kalimantan, North Macedonia, Northern Mariana Islands, Novgorod oblast, Novosibirsk oblast, Oman, Omsk oblast, Opolskie, Orenburg oblast, Oryol oblast, Palau, Penza oblast, Perm kray, Piemonte, Podkarpackie, Podlaskie, Pomorskie, Portugal, Primorsky kray, Provincia autonoma di Bolzano, Provincia

	autonoma di Trento, Pskov oblast, Puglia, Republic of Adygeya, Republic of Altai, Republic of Bashkortostan, Republic of Buryatia, Republic of Crimea, Republic of Dagestan, Republic of Ingushetia, Republic of Kalmykia, Republic of Karelia, Republic of Khakasia, Republic of Mariy El, Republic of Moldova, Republic of Mordovia, Republic of North Ossetia-Alania, Republic of Sakha (Yakutia), Republic of Tatarstan, Republic of Tuva, Riau, Riau Islands, Rio de Janeiro, Romania, Rostov oblast, Ryazan oblast, Saint Kitts and Nevis, Sakhalin oblast, Samara oblast, Sankt-Petersburg, Santa Catarina, Sao Paulo, Saratov oblast, Sardegna, Serbia, Sevastopol, Seychelles, Shaanxi, Shandong, Shanghai, Sicilia, Sikkim, Urban, Slaskie, Smolensk oblast, Spain, Sri Lanka, Stavropol kray, Sverdlovsk oblast, Swietokrzyskie, Tambov oblast, Tianjin, Tomsk oblast, Toscana, Trinidad and Tobago, Tula oblast, Turkey, Tver oblast, Tyumen oblast without autonomous areas, Udmurt Republic, Ukraine (without Crimea & Sevastopol), Ulyanovsk oblast, Umbria, Union Territories other than Delhi, Urban, United States Virgin Islands, Uruguay, Uttarakhand, Urban, Valle d'Aosta, Veneto, Vladimir oblast, Volgograd oblast, Vologda oblast, Voronezh oblast, Warminsko-Mazurskie, Wielkopolskie, Yamalo-Nenets autonomous area, Yaroslavl oblast, Zabaikalsk kray, Zachodniopomorskie, Zhejiang
**High SDI**	Aichi, Akershus, Akita, Alabama, Alaska, Andorra, Aomori, Arizona, Arkansas, Aust-Agder, Australia, Austria, Barking and Dagenham, Barnet, Barnsley, Bath and North East Somerset, Bedford, Beijing, Belgium, Bermuda, Bexley, Birmingham, Blackburn with Darwen, Blackpool, Bolton, Bournemouth, Bracknell Forest, Bradford, Brent, Brighton and Hove, Bristol, City of, Bromley, Brunei Darussalam, Buckinghamshire, Bury, Buskerud, Calderdale, California, Cambridgeshire, Camden, Canada, Central Bedfordshire, Cheshire East, Cheshire West and Chester, Chiba, Colorado, Connecticut, Cornwall, County Durham, Coventry, Croydon, Cumbria, Cyprus, Czechia, Darlington, Delaware, Denmark, Derby, Derbyshire, Devon, District of Columbia, Doncaster, Dorset, Dudley, Ealing, East Riding of Yorkshire, East Sussex, Ehime, Enfield, Essex, Estonia, Finland, Finnmark, Florida, France, Fukui, Fukuoka, Fukushima, Gateshead, Georgia, Germany, Gifu, Gloucestershire, Greenwich, Guam, Gunma, Hackney, Halton, Hammersmith and Fulham, Hampshire, Haringey, Harrow, Hartlepool, Havering, Hawaii, Hedmark, Herefordshire, County of, Hertfordshire, Hillingdon, Hiroshima, Hokkaido, Hong Kong Special Administrative Region of China, Hordaland, Hounslow, Hyogo, Ibaraki, Iceland, Idaho, Illinois, Indiana, Iowa, Ireland, Ishikawa, Isle of Wight, Islington, Iwate, Kagawa, Kagoshima, Kanagawa, Kansas, Kensington and Chelsea, Kent, Kentucky, Kingston upon Hull, City of, Kingston upon Thames, Kirklees, Knowsley, Kochi, Kumamoto, Kuwait, Kyoto, Lambeth, Lancashire, Latvia, Leeds, Leicester, Leicestershire, Lewisham, Lincolnshire, Lithuania, Liverpool, Louisiana, Luton, Luxembourg, Macao Special Administrative Region of China, Maine, Manchester, Maryland, Massachusetts, Medway, Merton, Michigan, Middlesbrough, Mie, Milton Keynes, Minnesota, Missouri, Miyagi, Miyazaki, Monaco, Montana, More og Romsdal, Nagano, Nagasaki, Nara, Nebraska, Netherlands, Nevada, New Hampshire, New Jersey, New Mexico, New York, New Zealand Maori population, New Zealand non-Maori population, Newcastle upon Tyne, Newham, Niigata, Nordland, Norfolk, North Carolina, North Dakota, North East Lincolnshire, North Lincolnshire, North Somerset, North Tyneside, North Yorkshire, Northamptonshire, Northern Ireland, Northumberland, Nottingham, Nottinghamshire,

	Ohio, Oita, Okayama, Okinawa, Oklahoma, Oldham, Oppland, Oregon, Osaka, Oslo, Ostfold, Oxfordshire, Pennsylvania, Peterborough, Plymouth, Poole, Portsmouth, Puerto Rico, Qatar, Reading, Redbridge, Redcar and Cleveland, Republic of Korea, Rhode Island, Richmond upon Thames, Rochdale, Rogaland, Rotherham, Rutland, Saga, Saitama, Salford, San Marino, Sandwell, Saudi Arabia, Scotland, Sefton, Sheffield, Shiga, Shimane, Shizuoka, Shropshire, Singapore, Slough, Slovakia, Slovenia, Sogn og Fjordane, Solihull, Somerset, South Carolina, South Dakota, South Gloucestershire, South Tyneside, Southampton, Southend-on-Sea, Southwark, St Helens, Staffordshire, Stockholm, Stockport, Stockton-on-Tees, Stoke-on-Trent, Suffolk, Sunderland, Surrey, Sutton, Sweden except Stockholm, Swindon, Switzerland, Taiwan (Province of China), Tameside, Telemark, Telford and Wrekin, Tennessee, Texas, Thurrock, Tochigi, Tokushima, Tokyo, Torbay, Tottori, Tower Hamlets, Toyama, Trafford, Troms, Trondelag, United Arab Emirates, Utah, Vermont, Vest-Agder, Vestfold, Virginia, Wakayama, Wakefield, Wales, Walsall, Waltham Forest, Wandsworth, Warrington, Warwickshire, Washington, West Berkshire, West Sussex, West Virginia, Westminster, Wigan, Wiltshire, Windsor and Maidenhead, Wirral, Wisconsin, Wokingham, Wolverhampton, Worcestershire, Wyoming, Yamagata, Yamaguchi, Yamanashi, York

14 GATHER checklist

Table 136. GATHER checklist

Item #	Checklist item	Reported on page #
Objectives and funding		
1	Define the indicator(s), populations (including age, sex, and geographic entities), and time period(s) for which estimates were made.	Main text (Introduction, pg. 6-8)
2	List the funding sources for the work.	Main text (Methods; pg. 15)
Data Inputs		
For all data inputs from multiple sources that are synthesized as part of the study:		
3	Describe how the data were identified and how the data were accessed.	Main text (Methods; pg. 9-15) and Appendix (pg. 6-7, 9, 17, 19-20, 34, 36-37, 40-41, 51, 58)
4	Specify the inclusion and exclusion criteria. Identify all ad-hoc exclusions.	Appendix (pg. 6-7, 9, 13-14, 17, 19-20, 25-26, 36-37, 40, 42, 51, 58, 65)
5	Provide information on all included data sources and their main characteristics. For each data source used, report reference information or contact name/institution, population represented, data collection method, year(s) of data collection, sex and age range, diagnostic criteria or measurement method, and sample size, as relevant.	Online data citation tools
6	Identify and describe any categories of input data that have potentially important biases (e.g., based on characteristics listed in item 5).	Appendix (pg. 7, 9-11, 17, 19, 25-28, 36-37, 51)
For data inputs that contribute to the analysis but were not synthesized as part of the study:		
7	Describe and give sources for any other data inputs.	Online data citation tools http://ghdx.healthdata.org/gbd-2019
For all data inputs:		
8	Provide all data inputs in a file format from which data can be efficiently extracted (e.g., a spreadsheet rather than a PDF), including all relevant meta-data listed in item 5. For any data inputs that cannot be shared because of ethical or legal reasons, such as third-party ownership, provide a contact name or the name of the institution that retains the right to the data.	Online data visualization tools, data query tools, and the Global Health Data Exchange http://ghdx.healthdata.org
Data analysis		
9	Provide a conceptual overview of the data analysis method. A diagram may be helpful.	Main text (Methods; pg. 9) and Appendix (Figures 1, 4, 13, 16, 19, 23)

10	Provide a detailed description of all steps of the analysis, including mathematical formulae. This description should cover, as relevant, data cleaning, data pre-processing, data adjustments and weighting of data sources, and mathematical or statistical model(s).	Main text (Methods; pg. 9–15)
11	Describe how candidate models were evaluated and how the final model(s) were selected.	Appendix (pg. 37)
12	Provide the results of an evaluation of model performance, if done, as well as the results of any relevant sensitivity analysis.	Appendix (pg. 37)
13	Describe methods for calculating uncertainty of the estimates. State which sources of uncertainty were, and were not, accounted for in the uncertainty analysis.	Main text (Methods; pg. 9)
14	State how analytic or statistical source code used to generate estimates can be accessed.	Code is provided in an online repository, http://ghdx.healthdata.org/gbd-2019/code
Results and Discussion		
15	Provide published estimates in a file format from which data can be efficiently extracted.	Visualization tools, data query tools, and the Global Health Data Exchange, http://ghdx.healthdata.org/gbd-2019
16	Report a quantitative measure of the uncertainty of the estimates (e.g. uncertainty intervals).	Main text (Results, Table 1), Appendix (Supplemental Table 2 and 3, pg 114-135), and online data tools
17	Interpret results in light of existing evidence. If updating a previous set of estimates, describe the reasons for changes in estimates.	Main text (Discussion, pg 19-25) and Appendix (pg. 65-66)
18	Discuss limitations of the estimates. Include a discussion of any modelling assumptions or data limitations that affect interpretation of the estimates.	Main text (Discussion, pg 24-26)

15 Supplementary tables and figures

Supplemental Table 1. HBsAg prevalence data sources a) for counterfactual model and b) for vetting of final estimates

Supplemental Table 2. Percentage change in HBsAg prevalence (%) and prevalence counts in all-ages and under 5 years between 1990 and 2019 and 2015 and 2019, by location

Supplemental Table 3. Percentage change in HBV-related death rates and HBV-related death counts in all-ages between 1990 and 2019 and 2015 and 2019 by location

Supplemental Table 4. Achievement in 2019 of WHO-GHSS 2020 death impact target, WHO-GHSS new cases impact target, and under-5 prevalence proxy target, with high probability, by location. High probability is defined by at least 950 out of 1000 draws meeting target.

Supplementary Table 5. Achievement in 2019 of WHO Interim Guidance's absolute mortality rate target and WHO-GHSS under-5 prevalence target for 2030, with high probability, by location. High probability is defined by at least 950 out of 1000 draws.

Supplementary Table 6. Prevalence of HBsAg, all ages, both sexes, as reported by GBD and multiple other estimation groups

Supplemental Figure 1. Hepatitis-related deaths by virus and cause, global, 2019

Supplemental Figure 2. Percentage of HBV-related disability-adjusted life-years (DALYs) in top burden countries (out of total HBV-related DALYs)

Supplemental Figure 3. Map of percentage change in all-age HBV-related death counts (A) and all-age HBV death rates (B) between 2015 and 2019 by country. Percentage change category was achieved with high probability (at least 950 out of 1000 draws of percentage change).

Supplemental Figure 4. Map of percentage change in under-5 HBsAg prevalent counts (A) and under 5 HBsAg seroprevalence (%) (B) between 2015 and 2019 by country. Percentage change category was achieved with high probability (at least 950 out of 1000 draws of percentage change).

Supplemental Figure 5. HBV-related death counts by WHO regions by cause

Supplemental Figure 6. Age-sex-specific death counts of HBV-related diseases in 2019 by global and WHO regions

Supplemental Figure 7. Age-sex-specific death rates per 100,000 of HBV-related diseases in 2019 by global and WHO regions

Supplemental Table 1: HBsAg prevalence data sources a) for counterfactual model and b) for vetting of final estimates

a)

Citation	Location	Year start	Year end
Abdel Raheem SM, Abou-Lohum TS, el-Didy H, el-Eriani H, Mansour S, Hafez AS. Hepatitis B infection in Sanaa City, Republic of Yemen. Prevalence among pregnant women and materno-foetal transmission. J Egypt Public Health Assoc. 1991; 66(56): 491503.	Yemen	1988	1990
Abdelwahab S, Rewisha E, Hashem M, Sobhy M, Galal I, Allam WR, Mikhail N, Galal G, El-Tabbakh M, El-Kamary SS, Waked I, Strickland GT. Risk factors for hepatitis C virus infection among Egyptian healthcare workers in a national liver diseases referral centre. Trans R Soc Trop Med Hyg. 2012; 106(2): 98103.	Egypt	2008	2010
Abdool Karim SS, Coovadia HM, Windsor IM, Thejpal R, van den Ende J, Fouche A. The prevalence and transmission of hepatitis B virus infection in urban, rural and institutionalized black children of Natal/KwaZulu, South Africa. Int J Epidemiol. 1988; 17(1): 16873.	KwaZulu-Natal	1985	1985
Abdul Mujeeb S, Aamir K, Mehmood K. Seroprevalence of HBV, HCV and HIV infections among college going first time voluntary blood donors. J Pak Med Assoc. 2000; 50(8): 269-70.	Sindh	1998	1999
Abdul Mujeeb S, Nanan D, Sabir S, Altaf A, Kadir M. Hepatitis B and C infection in first-time blood donors in Karachi--a possible subgroup for sentinel surveillance. East Mediterr Health J. 2006; 12(6): 735-41.	Sindh	2000	2000
Abdulla EM, Abdulla FE. Seropositive HBsAg frequency in Karachi and interior Sindh, Pakistan. Pak J Med Sci. 2007; 23(2): 157-60.	Sindh	1998	2006
Abdulla EM, Abdulla FE. Seropositive HBsAg frequency in Karachi and interior Sindh, Pakistan. Pak J Med Sci. 2007; 23(2): 157-60.	Sindh	2005	2006
Abdullah FE, Memon AA, Mushtaq A, Adil SE-R, Kazmi SU. Frequency of HBsAg positivity: a laboratory data analysis. J Coll Physicians Surg Pak. 2012; 22(11): 7423.	Sindh	2009	2010
Abebe A, Nokes DJ, Dejene A, Enquselassie F, Messele T, Cutts FT. Seroepidemiology of hepatitis B virus in Addis Ababa, Ethiopia: transmission patterns and vaccine control. Epidemiol Infect. 2003; 131(1): 75770.	Addis Ababa	1994	1994
Abedi F, Madani H, Asadi A, Nejatizadeh A. Significance of blood-related high-risk behaviors and horizontal transmission of hepatitis B virus in Iran. Arch Virol. 2011; 156(4): 629-35.	Iran (Islamic Republic of)	2008	2009
Abou MAA, Eltahir YM, Ali AS. Seroprevalence of hepatitis B virus and hepatitis C virus among blood donors in Nyala, South Dar Fur, Sudan. Virol J. 2009; 6: 146.	Sudan	2007	2007
Abusheikha N, Akagbosu F, Marcus S, Lass A, Cousins C, Brinsden P. Viral screening and assisted conception treatment--the Bourn Hall experience. J Assist Reprod Genet. 1999; 16(6): 337-9.	England	1987	1998
Abuzwaida AR, Sidoni M, Yoshida CF, Schatzmayr HG. Seroepidemiology of hepatitis A and B in two urban communities of Rio de Janeiro, Brazil. Rev Inst Med Trop Sao Paulo. 1987; 29(4): 219-23.	Rio de Janeiro	1986	1986
Aceti A, Paparo BS, Celestino D, Pennica A, Caferro M, Grilli A, Sebastiani A, Mohamud OM, Abdirahman M, Bile K. Sero-epidemiology of hepatitis delta virus infection in Somalia. Trans R Soc Trop Med Hyg. 1989; 83(3): 399400.	Somalia	1986	1988
Achwan WA, Muttaqin Z, Zakaria E, Depamede SA, Mulyanto, Sumoharjo S, Tsuda F, Takahashi K, Abe N, Mishiro S.	Indonesia	2005	2005

Epidemiology of hepatitis B, C, and E viruses and human immunodeficiency virus infections in Tahuna, Sangihe-Talaud Archipelago, Indonesia. Intervirology. 2007; 50(6): 408-11.			
Acquaye JK, Mingle JA. Hepatitis B viral markers in Ghanaian pregnant women. West Afr J Med. 1994; 13(3): 134-7.	Ghana	1992	1992
Adabara NU, Ajala OO, Momohjimoh A, Hashimu Z, Agabi AYV. Prevalence of hepatitis B virus among women attending antenatal clinic in the general hospital, Minna, Niger State. Shiraz E Med J. 2012; 13(1): 28-32.	Niger	2010	2010
Adamo B, Stroffolini T, Sagliocca L, Simonetti A, Iadanza F, Fossi E, Tancredi F, Mele A. Ad hoc survey of hepatitis B vaccination campaign in newborns of HBsAg positive mothers and in 12-year-old subjects in southern Italy. Vaccine. 1998; 16(8): 775-7.	Campania	1994	1994
Addidle M. Impact of universal hepatitis B vaccination on antenatal hepatitis B prevalence in the Midlands region of the North Island, New Zealand. N Z Med J. 2011; 124(1332): 404.	New Zealand	1997	2009
Adebajo AO, Smith DJ, Hazleman BL, Wreghitt TG. Seroepidemiological associations between tuberculosis, malaria, hepatitis B, and AIDS in West Africa. J Med Virol. 1994; 42(4): 366-8.	Oyo	1990	1990
Aganga-Williams OM, Akanmu AS, Akinsete I, Njoku OS. Prevalence of hepatitis B surface antigen among women of childbearing age in Lagos, Nigeria. Afr J Reprod Health. 1999; 3(1): 45-50.	Lagos	1996	1998
Agbenu E, Banla A, Kolou M, DAlmeida A, Kpotsra A, Dorkenoo A, Redah D. Serologic markers used for hepatitis B surveillance in Togo: status report and action proposals. Med Trop (Mars). 2008; 68(6): 6214.	Togo	2007	2007
Agdamag DM, Kageyama S, Alesna ET, Solante RM, Leaño PS, Heredia AML, Abellanosa-Tac-An IP, Vibal ET, Jereza LD, Ichimura H. Rapid spread of hepatitis C virus among injecting-drug users in the Philippines: Implications for HIV epidemics. J Med Virol. 2005; 77(2): 221-6.	Cebu	2002	2002
Aguiar JI, Aguiar E, Paniago A, Cunha R, Galvão L, Daher R. Prevalence of antibodies to hepatitis B core antigen in blood donors in the middle West region of Brazil. Mem Inst Oswaldo Cruz. 2001; 96(2): 1857.	Mato Grosso do Sul	1997	1999
Aguilar CE, Soliman AS, McConnell DS, Zekri A-R, Banerjee M, Omar A, Sharawy M, Omar S, Raouf A, Sowers MR. Androgen profiles among Egyptian adults considering liver status. J Gastroenterol Hepatol. 2008; 23(7 Pt 2): e137-145.	Egypt	2005	2007
Ahmad A, Ahmad B, Ali A, et al. Seroprevalence of HBsAg and anti-HCV in general healthy population of Swat district with frequency of different HCV genotypes. Pak J Med Sci. 2009; 25(5): 744-748.	Khyber Pakhtunkhwa	2007	2008
Ahmed Elmukashfi T, Ali Ibrahim O, Elkhidir IM, Ali Bashir A, Ali Awad Elkarim M. Socio-demographic characteristics of health care workers and hepatitis B virus (HBV) infection in public teaching hospitals in Khartoum State, Sudan. Glob J Health Sci. 2012; 4(4): 3741.	Sudan	2004	2004
Aidaoui M, Bouzbid S, Laouar M. Seroprevalence of HIV infection in pregnant women in the Annaba region (Algeria). Rev Epidemiol Sante Publique. 2008; 56(4): 2616.	Algeria	2003	2004
Ajayi AO, Komolafe AO, Ajumobi K. Seroprevalence of hepatitis B surface antigenemia among health care workers in a Nigerian tertiary health institution. Niger J Clin Pract. 2007; 10(4): 2879.	Ekiti	2003	2003

Akbar N, Basuki B, Mulyanto, Garabrant DH, Sulaiman A, Noer HM. Ethnicity, socioeconomic status, transfusions and risk of hepatitis B and hepatitis C infection. J Gastroenterol Hepatol. 1997; 12(11): 752-7.	Indonesia	1994	1994
Akbar SM, Onji M, Kanaoka M, Horiike N, Michitaka K, Masumoto T, Nonaka T, Kanda K, Kajino K, Kumamoto I. The seroepidemiology of hepatitis A and B in a Japanese town. Asia Pac J Public Health. 1992; 6(2): 26-9.	Japan	1989	1990
Akbar SMF, Hossain M, Hossain Md F, et al. Seroepidemiolgy of hepatitis viruses of chronic liver diseases in bangladesh: High prevalence of hcv among blood donors and healthy person. Hepatol Res. 1997; 7(2): 113-120.	Bangladesh	1993	1993
Akcam FZ, Uskun E, Avsar K, Songur Y. Hepatitis B virus and hepatitis C virus seroprevalence in rural areas of the southwestern region of Turkey. Int J Infect Dis. 2009; 13(2): 274-84.	Turkey	2006	2007
Al Awaidy S, Abu-Elyazeed R, Al Hosani H, Al Mulla A, Al Busaiedy S, Al Amiry A, Farah Z, Al Marrie A, Bock HL, Al-Shaar I, Shah S. Sero-epidemiology of hepatitis B infection in pregnant women in Oman, Qatar and the United Arab Emirates. J Infect. 2006; 52(3): 2026.	Oman	2000	2000
Al Awaidy S, Abu-Elyazeed R, Al Hosani H, Al Mulla A, Al Busaiedy S, Al Amiry A, Farah Z, Al Marrie A, Bock HL, Al-Shaar I, Shah S. Sero-epidemiology of hepatitis B infection in pregnant women in Oman, Qatar and the United Arab Emirates. J Infect. 2006; 52(3): 2026.	Qatar	2000	2000
Al Awaidy S, Abu-Elyazeed R, Al Hosani H, Al Mulla A, Al Busaiedy S, Al Amiry A, Farah Z, Al Marrie A, Bock HL, Al-Shaar I, Shah S. Sero-epidemiology of hepatitis B infection in pregnant women in Oman, Qatar and the United Arab Emirates. J Infect. 2006; 52(3): 2026.	United Arab Emirates	2000	2000
Al Awaidy ST, Bawikar SP, Al Busaidy SS, Al Mahrouqi S, Al Baqlani S, Al Obaidani I, Alexander J, Patel MK. Progress toward elimination of hepatitis B virus transmission in Oman: impact of hepatitis B vaccination. Am J Trop Med Hyg. 2013; 89(4): 8115.	Oman	2005	2005
Alavian SM, Tabatabaei SV, Nourizad S, Mansouri F, Khademi N, Amini Kafi-abad S, Gharehbaghian A, Abolghasemi H. Seroepidemiology of HBV Infection in Kermanshah- West of Iran; a Population Based Study. Jundishapur J Microbiol. 2012; 5(4): 564-9.	Iran (Islamic Republic of)	2009	2010
al-Faleh FZ, Ramia S, Arif M, Ayoola EA, al-Rashed RS, al-Jeffry M, Hossain A, el-Hazmi M. Profile of hepatitis C virus and the possible modes of transmission of the virus in the Gizan area of Saudi Arabia: a community-based study. Ann Trop Med Parasitol. 1995; 89(4): 431-7.	Saudi Arabia	1993	1993
Alikor EA, Erhabor ON. Seroprevalence of hepatitis B surface antigenaemia in children in a tertiary health institution in the Niger Delta of Nigeria. Niger J Med. 2007; 16(3): 250-1.	Rivers	1999	2004
Alizadeh AHM, Ranjbar M, Ansari S, MirArab A, Alavian SM, Mohammad K, Adibi P, Sadri GH, Keramat F, Ardalan A, Arabi M, Gharekhani S, Ataei A, Amraei GR, Hosseinzadeh M, Hatami S, Zali M. Seroprevalence of hepatitis B in Nahavand, Islamic Republic of Iran. East Mediterr Health J. 2006; 12(5): 528-37.	Iran (Islamic Republic of)	2003	2003
Alkan ML, Maayan S, Belmaker I, Arbeli Y, Mani N, Ben-Yshai F. Serological markers for hepatitis B and treponemal infection among HIV carriers from Ethiopia. Isr J Med Sci. 1993; 29(6-7): 390-2.	Ethiopia	1991	1991

Al-Moslih MI, Al-Huraibi MA. Prevalence of hepatitis C virus among patients with liver disease in the Republic of Yemen. East Mediterr Health J. 2001; 7(4-5): 771-8.	Yemen	2001	2001
Al-Nassiri K, Raja'a Y. Hepatitis B infection in Yemenis in Sana'a: pattern and risk factors. East Mediterr Health J. 2000; 7(1-2): 147-52.	Yemen	1999	1999
Alvarado-Mora MV, Gutierrez Fernandez MF, Gomes-Gouvêa MS, de Azevedo Neto RS, Carrilho FJ, Pinho JRR. Hepatitis B (HBV), Hepatitis C (HCV) and Hepatitis Delta (HDV) Viruses in the Colombian Population-How Is the Epidemiological Situation?. PLoS One. 2011; 6(4): e18888.	Colombia	2008	2009
Amazigo UO, Chime AB. Hepatitis-B virus infection in rural and urban populations of eastern Nigeria: prevalence of serological markers. East Afr Med J. 1990; 67(8): 539-44.	Anambra	1988	1988
Amazigo UO, Chime AB. Hepatitis-B virus infection in rural and urban populations of eastern Nigeria: prevalence of serological markers. East Afr Med J. 1990; 67(8): 539-44.	Enugu	1988	1988
Amini S, Mahmoodi MF, Andalibi S, Solati AA. Seroepidemiology of hepatitis B, delta and human immunodeficiency virus infections in Hamadan province, Iran: a population based study. J Trop Med Hyg. 1993; 96(5): 277-87.	Iran (Islamic Republic of)	1989	1989
Amirudin R, Akil H, Akahane Y, Suzuki H. Hepatitis B and C virus infection in Ujung Pandang, Indonesia. Gastroenterol Jpn. 1991; 26(3): 184-8.	Indonesia	1990	1990
Anderson SR, Righarts A, Maguire H. Surveillance of antenatal infections--HIV, hepatitis B, syphilis and rubella susceptibility in London. Commun Dis Public Health. 2004; 7(4): 251-7.	England	2002	2002
Ansari-Moghaddam A, Ostovaneh MR, Sharifi Mood B, Sanei-Moghaddam E, Modabbernia A, Poustchi H. Seroprevalence of hepatitis B surface antigen and anti hepatitis C antibody in zahedan city, iran: a population-based study. Hepat Mon. 2012; 12(9): e6618.	Iran (Islamic Republic of)	2008	2009
Aquino JA, Pegado KA, Barros LP, Machado LFA. Soroprevalência de infecções por vírus da hepatite B e vírus da hepatite C em indivíduos do Estado do Pará. Rev Soc Bras Med Trop. 2008; 334-7.	Brazil	2002	2005
Arguillas MO, Domingo EO, Tsuda F, Mayumi M, Suzuki H. Seroepidemiology of hepatitis C virus infection in the Philippines: a preliminary study and comparison with hepatitis B virus infection among blood donors, medical personnel, and patient groups in Davao, Philippines. Gastroenterol Jpn. 1991; 170-5.	Davao Del Sur	1990	1990
Arivelo RZ, Hendrison RD, Elie RF, Tantely RM, Ramamonjisoa A, Barnia RF, Paquerette HS, Raft HF, Aimée RAO, Andry R. The seroprevalence of hepatitis B surface antigen among first time blood donors in Antananarivo (Madagascar) from 2003 to 2009. Blood Transfus. 2011; 9(4): 4757.	Madagascar	2003	2009
Armstrong SA, Gangam N, Chipman ML, Rootman DS. The prevalence of positive hepatitis B, hepatitis C, and HIV serology in cornea donors prescreened by medical and social history in Ontario, Canada. Cornea. 1997; 16(5): 512-6.	Canada	1993	1993
Armstrong SA, Gangam N, Chipman ML, Rootman DS. The prevalence of positive hepatitis B, hepatitis C, and HIV serology in cornea donors prescreened by medical and social history in Ontario, Canada. Cornea. 1997; 16(5): 512-6.	Canada	1993	1996
Armstrong SA, Gangam N, Chipman ML, Rootman DS. The prevalence of positive hepatitis B, hepatitis C, and HIV serology in cornea donors prescreened by medical and social history in Ontario, Canada. Cornea. 1997; 16(5): 512-6.	Canada	1994	1994

Armstrong SA, Gangam N, Chipman ML, Rootman DS. The prevalence of positive hepatitis B, hepatitis C, and HIV serology in cornea donors prescreened by medical and social history in Ontario, Canada. Cornea. 1997; 16(5): 512-6.	Canada	1995	1995
Armstrong SA, Gangam N, Chipman ML, Rootman DS. The prevalence of positive hepatitis B, hepatitis C, and HIV serology in cornea donors prescreened by medical and social history in Ontario, Canada. Cornea. 1997; 16(5): 512-6.	Canada	1996	1996
Asakura T, Tachibana K, Watanabe S, Teshima D, Ikeda M, Tokudome S. Concomitant carriage of hepatitis B virus and human T-lymphotropic virus type I among blood donors in Kitakyushu, Japan. J Infect. 1991; 23(1): 33-7.	Japan	1988	1988
Ashraf H, Alam NH, Rothermundt C, Brooks A, Bardhan P, Hossain L, Salam MA, Hassan MS, Beglinger C, Gyr N. Prevalence and risk factors of hepatitis B and C virus infections in an impoverished urban community in Dhaka, Bangladesh. BMC Infect Dis. 2010; 10(1): 208.	Bangladesh	2005	2006
Aspinall S, Joubert JJ, Evans AC, Joseph S, Steele AD, Lecatsas G. Prevalence of hepatitis B in !Kung (San) children from Bushmanland, Namibia. Ann Trop Paediatr. 1994; 14(2): 163-7.	Namibia	1992	1992
Assi C, Allah-Kouadio E, Ouattara A, et al. [Vaccination coverage against hepatitis B and the prevalence of HBsAg in a profession at risk: Cross-sectional study of 244 firefighters in Abidjan]. J Afr Hepato Gastroenterol. 2011; 5(2): 115-8.	Côte d'Ivoire	2009	2009
Atabek ME, Kart H, Erkul I. Prevalence of hepatitis A, B, C and E virus in adolescents with type-1 diabetes mellitus. Int J Adolesc Med Health. 2003; 15(2): 133-7.	Turkey	2001	2001
Atanasova MV, Haydouchka IA, Zlatev SP, Stoilova YD, Iliev YT, Mateva NG. Prevalence of antibodies against hepatitis C virus and hepatitis B coinfection in healthy population in Bulgaria. A seroepidemiological study. Minerva Gastroenterol Dietol. 2004; 50(1): 89-96.	Bulgaria	1999	2000
Aubry P, Niel L, Niyongabo T, Kerguelen S, Larouze B. Seroprevalence of hepatitis E virus in an adult urban population from Burundi. Am J Trop Med Hyg. 1997; 57(3): 272-3.	Burundi	1992	1993
Ayed Z, Houinato D, Hocine M, Ranger-Rogez S, Denis F. Prevalence of serum markers of hepatitis B and C in blood donors and pregnant women in Algeria. Bull Soc Pathol Exot. 1995; 88(5): 2258.	Algeria	1992	1993
Baha W, Foullous A, Dersi N, They-they TP, El alaoui K, Nourichafi N, Oukkache B, Lazar F, Benjelloun S, Ennaji MM, Elmalki A, Mifdal H, Bennani A. Prevalence and risk factors of hepatitis B and C virus infections among the general population and blood donors in Morocco. BMC Public Health. 2013; 50.	Morocco	2005	2011
Baha W, Foullous A, Dersi N, They-they TP, El alaoui K, Nourichafi N, Oukkache B, Lazar F, Benjelloun S, Ennaji MM, Elmalki A, Mifdal H, Bennani A. Prevalence and risk factors of hepatitis B and C virus infections among the general population and blood donors in Morocco. BMC Public Health. 2013; 50.	Morocco	2008	2008
Baha W, Foullous A, Dersi N, They-they TP, El alaoui K, Nourichafi N, Oukkache B, Lazar F, Benjelloun S, Ennaji MM, Elmalki A, Mifdal H, Bennani A. Prevalence and risk factors of hepatitis B and C virus infections among the general population and blood donors in Morocco. BMC Public Health. 2013; 50.	Morocco	2009	2009
Baha W, Foullous A, Dersi N, They-they TP, El alaoui K, Nourichafi N, Oukkache B, Lazar F, Benjelloun S, Ennaji MM, Elmalki A, Mifdal H, Bennani A. Prevalence and risk factors of hepatitis B and C	Morocco	2010	2010

virus infections among the general population and blood donors in Morocco. BMC Public Health. 2013; 50.			
Bakir TM, Kurbaan KM, al Fawaz I, Ramia S. Infection with hepatitis viruses (B and C) and human retroviruses (HTLV-1 and HIV) in Saudi children receiving cycled cancer chemotherapy. J Trop Pediatr. 1995; 41(4): 206-9.	Saudi Arabia	1993	1993
Baldo V, Floreani A, Menegon T, Grella P, Paternoster DM, Trivello R. Hepatitis C virus, hepatitis B virus and human immunodeficiency virus infection in pregnant women in North-East Italy: a seroepidemiological study. Eur J Epidemiol. 2000; 16(1): 87-91.	Veneto	1996	1996
Balogun WO, Adeleye JO, Akinlade KS, Kuti M, Otegbayo JA. Low prevalence of hepatitis-C viral seropositivity among patients with type-2 diabetes mellitus in a tertiary hospital. J Natl Med Assoc. 2006; 98(11): 1805-8.	Oyo	2004	2004
Banerjee A, Chakravarty R, Mondal PN, Chakraborty MS. Hepatitis B virus genotype D infection among antenatal patients attending a maternity hospital in Calcutta, India: assessment of infectivity status. Southeast Asian J Trop Med Public Health. 2005; 36(1): 203-6.	India	1998	1998
Bart PA, Jacquier P, Zuber PL, Lavanchy D, Frei PC. Seroprevalence of HBV (anti-HBc, HBsAg and anti-HBs) and HDV infections among 9006 women at delivery. Liver. 1996; 16(2): 110-6.	Switzerland	1990	1991
Basaras M, Santamaría A, Sarsa M, Gutiérrez E, de Olano Y, Cisterna R. Seroprevalence of hepatitis B and C, and human immunodeficiency type 1 viruses in a rural population from the Republic of Equatorial Guinea. Trans R Soc Trop Med Hyg. 1999; 93(3): 2502.	Equatorial Guinea	1996	1998
Batina A, Kabemba S, Malengela R. Infectious markers among blood donors in Democratic Republic of Congo (DRC). Rev Med Brux. 2007; 28(3): 1459.	Democratic Republic of the Congo	2003	2004
Beckers K, Schaad UB, Heininger U. Compliance with antenatal screening for hepatitis B surface antigen carrier status in pregnant women and consecutive procedures in exposed newborns. Eur J Pediatr. 2004; 163(11): 654-7.	Switzerland	2001	2001
Belo AC. Prevalence of hepatitis B virus markers in surgeons in Lagos, Nigeria. East Afr Med J. 2000; 77(5): 283-5.	Lagos	1998	1998
Ben-Alaya-Bouafif N, Bahri O, Chlif S, Bettaieb J, Toumi A, Bel Haj HN, Zâatour A, Gharbi A, Dellagi K, Triki H, Ben Salah A. Heterogeneity of hepatitis B transmission in Tunisia: risk factors for infection and chronic carriage before the introduction of a universal vaccine program. Vaccine. 2010; 28(19): 33017.	Tunisia	1996	1996
Bernabe-Ortiz A, Carcamo CP, Scott JD, Hughes JP, Garcia PJ, Holmes KK. HBV infection in relation to consistent condom use: a population-based study in Peru. PLoS One. 2011; 6(9): e24721.	Peru	2005	2007
Bertherat E, Georges-Courbot MC, Nabias R, Georges AJ, Renaut A. Seroprevalence of four sexually transmitted diseases in a semi-urban population of Gabon. Int J STD AIDS. 1998; 9(1): 31-6.	Gabon	1996	1996
Bertolini DA, Pinho JRR, Saraceni CP, Moreira RC, Granato CFH, Carrilho FJ. Prevalence of serological markers of hepatitis B virus in pregnant women from Paraná State, Brazil. Braz J Med Biol Res. 2006; 39(8): 1083-90.	Brazil	1998	2002
Beutels M, Van Damme P, Aelvoet W, Desmyter J, Dondeyne F, Goilav C, Mak R, Muylle L, Pierard D, Stroobant A, Van Loock F, Waumans P, Vranckx R. Prevalence of hepatitis A, B and C in the Flemish population. Eur J Epidemiol. 1997; 13(3): 275-80.	Belgium	1993	1994

Bhalla P, Garg S, Kakkar M, Sharma VK. Community-based study of hepatitis B markers in women of reproductive age. Indian J Gastroenterol. 2003; 22(1): 33-4.	India	1996	2000
Bhatta CP, Thapa B, Rana BB. Seroprevalence of hepatitis ""B"" in Kathmandu Medical College Teaching Hospital (KMCTH). Kathmandu Univ Med J (KUMJ). 2003; 1(2): 113-6.	Nepal	2001	2001
Bhattacharya P, Chandra P-K, Datta S, Banerjee A, Chakraborty S, Rajendran K, Basu S-K, Bhattacharya S-K, Chakravarty R. Significant increase in HBV, HCV, HIV and syphilis infections among blood donors in West Bengal, Eastern India 2004-2005: exploratory screening reveals high frequency of occult HBV infection. World J Gastroenterol. 2007; 13(27): 3730-3.	India	2004	2004
Bhattacharya P, Chandra P-K, Datta S, Banerjee A, Chakraborty S, Rajendran K, Basu S-K, Bhattacharya S-K, Chakravarty R. Significant increase in HBV, HCV, HIV and syphilis infections among blood donors in West Bengal, Eastern India 2004-2005: exploratory screening reveals high frequency of occult HBV infection. World J Gastroenterol. 2007; 13(27): 3730-3.	India	2005	2005
Bialek SR, Helgenberger L, Fischer GE, Bower WA, Konelios M, Chaine J-P, Armstrong G, Williams IT, Bell BP. Impact of routine hepatitis B immunization on the prevalence of chronic hepatitis B virus infection in the marshall islands and the federated States of micronesia. Pediatr Infect Dis J. 2010; 29(1): 1822.	Micronesia (Federated States of)	2000	2000
Bialek SR, Helgenberger L, Fischer GE, Bower WA, Konelios M, Chaine J-P, Armstrong G, Williams IT, Bell BP. Impact of routine hepatitis B immunization on the prevalence of chronic hepatitis B virus infection in the marshall islands and the federated States of micronesia. Pediatr Infect Dis J. 2010; 29(1): 1822.	Micronesia (Federated States of)	2005	2005
Bialek SR, Helgenberger L, Fischer GE, Bower WA, Konelios M, Chaine J-P, Armstrong G, Williams IT, Bell BP. Impact of routine hepatitis B immunization on the prevalence of chronic hepatitis B virus infection in the marshall islands and the federated States of micronesia. Pediatr Infect Dis J. 2010; 29(1): 1822.	Marshall Islands	2007	2007
Bile K, Abdirahman M, Mohamud O, Aden C, Isse A, Nilsson L, Norder H, Magnius L. Late seroconversion to hepatitis B in a Somali village indicates the important role of venereal transmission. J Trop Med Hyg. 1991; 94(6): 36773.	Somalia	1985	1985
Bile K, Abdirahman M, Mohamud O, Aden C, Isse A, Nilsson L, Norder H, Magnius L. Late seroconversion to hepatitis B in a Somali village indicates the important role of venereal transmission. J Trop Med Hyg. 1991; 94(6): 36773.	Somalia	1989	1989
Bogaerts J, Ahmed J, Akhter N, Begum N, Rahman M, Nahar S, Van Ranst M, Verhaegen J. Sexually transmitted infections among married women in Dhaka, Bangladesh: unexpected high prevalence of herpes simplex type 2 infection. Sex Transm Infect. 2001; 77(2): 114-9.	Bangladesh	1996	1998
Bolzan HE, Chiera A. Homes for the aged. Are they a high risk population for hepatitis B?. Acta Gastroenterol Latinoam. 1993; 23(3): 14950.	Argentina	1990	1992
Bongiorno MR, Pistone G, Aricò G. Hepatitis B and hepatitis C virus infections in dermatological patients in west Sicily: a seroepidemiological study. J Eur Acad Dermatol Venereol. 2002; 16(1): 43-6.	Sicilia	2000	2000
Bonura F, Sorgi M, Perna AM, Puccio G, Tramuto F, Cajozzo C, Romano N, Vitale F. Pregnant women as a sentinel population to	Sicilia	2001	2003

target and implement hepatitis B virus (HBV) vaccine coverage: a three-year survey in Palermo, Sicily. Vaccine. 2005; 23(25): 3243-6.			
Bossali F, Taty-Taty R, Houssissa P, et al. Seroprevalence of co-infections: HBV-HCV-HIV in women delivering at the Adolphe Sirce maternity in 2010. Rev Med Brux. 2012; 6(4): 315-319.	Congo	2010	2010
Boulos R, Ruff AJ, Nahmias A, Holt E, Harrison L, Magder L, Wiktor SZ, Quinn TC, Margolis H, Halsey NA. Herpes simplex virus type 2 infection, syphilis, and hepatitis B virus infection in Haitian women with human immunodeficiency virus type 1 and human T lymphotropic virus type I infections. J Infect Dis. 1992; 166(2): 418-20.	Haiti	1989	1991
Bowden FJ, OKeefe EJ, Primrose R, Currie MJ. Sexually transmitted infections, blood-borne viruses and risk behaviour in an Australian senior high school population-the SHLiRP study. Sex Health. 2005; 2(4): 22936.	Australia	2002	2003
Brabin L, Brabin BJ, Dimitrakakis M, Gust I. Factors affecting the prevalence of infection with hepatitis B virus among non-pregnant women in the Alexishafen area of Papua New Guinea. Ann Trop Med Parasitol. 1989; 83(4): 365-74.	Papua New Guinea	1985	1987
Brady-West DC, Buchner LM. Retrospective audit of blood donation at a hospital-based blood centre. Implications for blood product supply and safety. West Indian Med J. 2000; 49(3): 226-8.	Jamaica	1995	1998
Braga WSM, Castilho M da C, Borges FG, Martinho AC de S, Rodrigues IS, Azevedo EP de, Scazufca M, Menezes PR. Prevalence of hepatitis B virus infection and carriage after nineteen years of vaccination program in the Western Brazilian Amazon. Rev Soc Bras Med Trop. 2012; 45(1): 13-7.	Brazil	2005	2006
Braga WSM, Castilho M da C, Borges FG, Martinho AC de S, Rodrigues IS, Azevedo EP de, Scazufca M, Menezes PR. Prevalence of hepatitis B virus infection and carriage after nineteen years of vaccination program in the Western Brazilian Amazon. Rev Soc Bras Med Trop. 2012; 45(1): 13-7.	Brazil	2007	2007
Braka F, Nanyunja M, Makumbi I, Mbabazi W, Kasasa S, Lewis RF. Hepatitis B infection among health workers in Uganda: evidence of the need for health worker protection. Vaccine. 2006; 24(4748): 69307.	Uganda	2003	2003
Brevi A, Naldi L, Cainelli T, Parazzini F. Prevalence and awareness of hepatitis B virus carrier status in Italy. Genitourin Med. 1993; 69(3): 241.	Lombardia	1989	1990
Burgess MA, McIntosh ED, Allars HM, Kenrick KG. Hepatitis B in urban Australian schoolchildren. No evidence of horizontal transmission between high-risk and low-risk groups. Med J Aust. 1993; 159(5): 3159.	Australia	1990	1991
Butsashvili M, Kamkamidze G, Kajaia M, Morse DL, Triner W, Dehovitz J, McNutt L-A. Occupational exposure to body fluids among health care workers in Georgia. Occup Med (Lond). 2012; 62(8): 6206.	Georgia	2006	2007
Bwogi J, Braka F, Makumbi I, Mishra V, Bakamutumaho B, Nanyunja M, Opio A, Downing R, Biryahwaho B, Lewis RF. Hepatitis B infection is highly endemic in Uganda: findings from a national serosurvey. Afr Health Sci. 2009; 9(2): 98108.	Uganda	2005	2005
Cacoub P, Ohayon V, Sekkat S, et al. Epidemiologic and virologic study of hepatitis C virus infection in Morocco. Gastroenterol Clin Biol. 2002; 24(2): 169-73.	Morocco	1995	1996

Cai W, Poethko-Müller C, Hamouda O, Radun D. Hepatitis B virus infections among children and adolescents in Germany: migration background as a risk factor in a low seroprevalence population. Pediatr Infect Dis J. 2011; 30(1): 1924.	Germany	2003	2006
Candotti D, Danso K, Allain J-P. Maternofetal transmission of hepatitis B virus genotype E in Ghana, west Africa. J Gen Virol. 2007; 88(Pt 10): 268695.	Ghana	2004	2006
Candotti D, Mundy C, Kadewele G, Nkhoma W, Bates I, Allain JP. Serological and molecular screening for viruses in blood donors from Ntcheu, Malawi: high prevalence of HIV-1 subtype C and of markers of hepatitis B and C viruses. J Med Virol. 2001; 65(1): 1-5.	Malawi	1999	1999
Cetinkaya F, Gürses N, Öztürk F. Hepatitis B seroprevalence among children in a Turkish hospital. J Hosp Infect. 1995; 29(3): 217-9.	Turkey	1993	1994
Chacaltana A, Espinoza J. Seroprevalence of the infection and risk factors of hepatitis B and C in healthy military personnel. Rev Gastroenterol Peru. 2008; 28(3): 21725.	Peru	2007	2007
Chang H-C, Yen C-J, Lee Y-C, Chiu T-Y, Jan C-F. Seroprevalence of hepatitis B viral markers among freshmen-20 years after mass hepatitis B vaccination program in Taiwan. J Formos Med Assoc. 2007; 106(7): 5139.	Taiwan (Province of China)	2003	2004
Chang H-C, Yen C-J, Lee Y-C, Chiu T-Y, Jan C-F. Seroprevalence of hepatitis B viral markers among freshmen--20 years after mass hepatitis B vaccination program in Taiwan. J Formos Med Assoc. 2007; 106(7): 513-9.	Taiwan (Province of China)	2003	2004
Chapman BA, Burt MJ, Frampton CM, Collett JA, Yeo KH, Wilkinson ID, Cook HB, Barclay MJ, Ross AG, George PM. The prevalence of viral hepatitis (HAV, HBV and HCV) in the Christchurch community. N Z Med J. 2000; 113(1118): 394-6.	New Zealand	1998	1998
Chaudhuri V, Nanu A, Panda SK, Chand P. Evaluation of serologic screening of blood donors in India reveals a lack of correlation between anti-HBc titer and PCR-amplified HBV DNA. Transfusion. 2003; 43(10): 1442-8.	India	2001	2001
Chen C-L, Yang J-Y, Lin S-F, Sun C-A, Bai C-H, You S-L, Chen C-J, Kao J-H, Chen P-J, Chen D-S. Slow decline of hepatitis B burden in general population: Results from a population-based survey and longitudinal follow-up study in Taiwan. J Hepatol. 2015; 63(2): 35463.	Taiwan (Province of China)	2002	2002
Chen C-Y, Hsu H-Y, Liu C-C, Chang M-H, Ni Y-H. Stable seroepidemiology of hepatitis B after universal immunization in Taiwan: A 3-year study of national surveillance of primary school students. Vaccine. 2010; 28(34): 56058.	Taiwan (Province of China)	2005	2005
Chen C-Y, Hsu H-Y, Liu C-C, Chang M-H, Ni Y-H. Stable seroepidemiology of hepatitis B after universal immunization in Taiwan: A 3-year study of national surveillance of primary school students. Vaccine. 2010; 28(34): 56058.	Taiwan (Province of China)	2006	2006
Chen C-Y, Hsu H-Y, Liu C-C, Chang M-H, Ni Y-H. Stable seroepidemiology of hepatitis B after universal immunization in Taiwan: A 3-year study of national surveillance of primary school students. Vaccine. 2010; 28(34): 56058.	Taiwan (Province of China)	2007	2007
Chen G, Lin W, Shen F, Iloeje UH, London WT, Evans AA. Chronic hepatitis B virus infection and mortality from non-liver causes: results from the Haimen City cohort study. Int J Epidemiol. 2005; 34(1): 132-7.	Jiangsu	1992	1993

Chen H-F, Li C-Y, Chen P, See T-T, Lee H-Y. Seroprevalence of hepatitis B and C in type 2 diabetic patients. J Chin Med Assoc. 2006; 69(4): 146-52.	Jiangsu	2003	2003
Chen HL, Chang MH, Ni YH, Hsu HY, Lee PI, Lee CY, Chen DS. Seroepidemiology of hepatitis B virus infection in children: Ten years of mass vaccination in Taiwan. JAMA. 1996; 276(11): 9068.	Taiwan (Province of China)	1984	1984
Chen HL, Chang MH, Ni YH, Hsu HY, Lee PI, Lee CY, Chen DS. Seroepidemiology of hepatitis B virus infection in children: Ten years of mass vaccination in Taiwan. JAMA. 1996; 276(11): 9068.	Taiwan (Province of China)	1989	1989
Chen HL, Chang MH, Ni YH, Hsu HY, Lee PI, Lee CY, Chen DS. Seroepidemiology of hepatitis B virus infection in children: Ten years of mass vaccination in Taiwan. JAMA. 1996; 276(11): 9068.	Taiwan (Province of China)	1994	1994
Chen S-M, Kung C-M, Yang W-J, Wang H-L. Efficacy of the nationwide hepatitis B infant vaccination program in Taiwan. J Clin Virol. 2011; 52(1): 116.	Taiwan (Province of China)	1995	2001
Chen S-M, Kung C-M, Yang W-J, Wang H-L. Efficacy of the nationwide hepatitis B infant vaccination program in Taiwan. J Clin Virol. 2011; 52(1): 116.	Taiwan (Province of China)	2002	2003
Chen S-M, Kung C-M, Yang W-J, Wang H-L. Efficacy of the nationwide hepatitis B infant vaccination program in Taiwan. J Clin Virol. 2011; 52(1): 116.	Taiwan (Province of China)	2004	2009
Chiaramonte M, Floreani A, Naccarato R. Hepatitis B virus infection in homes for the aged. J Med Virol. 1982; 9(4): 247-55.	Veneto	1980	1980
Chiaramonte M, Floreani A, Silvan C, Zampieri L, Trivello R, Renzulli G, Moschen M, Naccarato R. Hepatitis A and hepatitis B virus infection in children and adolescents in north-east Italy. J Med Virol. 1983; 12(3): 179-86.	Veneto	1979	1980
Chikwem JO, Mohammed I, Okara GC, Ukwandu NC, Ola TO. Prevalence of transmissible blood infections among blood donors at the University of Maiducuri Teaching Hospital, Maiduguri, Nigeria. East Afr Med J. 1997; 74(4): 213-6.	Borno	1992	1992
Chiquete E, Sánchez LV, Becerra G, Quintero A, Maldonado M, Panduro A. Performance of the serologic and molecular screening of blood donations for the hepatitis B and C viruses in a Mexican Transfusion Center. Ann Hepatol. 2005; 4(4): 275-8.	Jalisco	1999	1999
Chiquete E, Sánchez LV, Becerra G, Quintero A, Maldonado M, Panduro A. Performance of the serologic and molecular screening of blood donations for the hepatitis B and C viruses in a Mexican Transfusion Center. Ann Hepatol. 2005; 4(4): 275-8.	Jalisco	1999	2005
Chiquete E, Sánchez LV, Becerra G, Quintero A, Maldonado M, Panduro A. Performance of the serologic and molecular screening of blood donations for the hepatitis B and C viruses in a Mexican Transfusion Center. Ann Hepatol. 2005; 4(4): 275-8.	Jalisco	2000	2000
Chiquete E, Sánchez LV, Becerra G, Quintero A, Maldonado M, Panduro A. Performance of the serologic and molecular screening of blood donations for the hepatitis B and C viruses in a Mexican Transfusion Center. Ann Hepatol. 2005; 4(4): 275-8.	Jalisco	2001	2001
Chiquete E, Sánchez LV, Becerra G, Quintero A, Maldonado M, Panduro A. Performance of the serologic and molecular screening of blood donations for the hepatitis B and C viruses in a Mexican Transfusion Center. Ann Hepatol. 2005; 4(4): 275-8.	Jalisco	2002	2002
Chiquete E, Sánchez LV, Becerra G, Quintero A, Maldonado M, Panduro A. Performance of the serologic and molecular screening of blood donations for the hepatitis B and C viruses in a Mexican Transfusion Center. Ann Hepatol. 2005; 4(4): 275-8.	Jalisco	2003	2003

Chiquete E, Sánchez LV, Becerra G, Quintero A, Maldonado M, Panduro A. Performance of the serologic and molecular screening of blood donations for the hepatitis B and C viruses in a Mexican Transfusion Center. Ann Hepatol. 2005; 4(4): 275-8.	Jalisco	2004	2004
Chlabicz S, Bonifatiuk I, Radziwon P. Prevalence of hepatitis C virus antibodies among blood donors in north-eastern Poland. Hepatol Res. 2005; 33(3): 206-10.	Podlaskie	1998	1998
Chlabicz S, Bonifatiuk I, Radziwon P. Prevalence of hepatitis C virus antibodies among blood donors in north-eastern Poland. Hepatol Res. 2005; 33(3): 206-10.	Podlaskie	1999	1999
Chlabicz S, Bonifatiuk I, Radziwon P. Prevalence of hepatitis C virus antibodies among blood donors in north-eastern Poland. Hepatol Res. 2005; 33(3): 206-10.	Podlaskie	2000	2000
Chlabicz S, Bonifatiuk I, Radziwon P. Prevalence of hepatitis C virus antibodies among blood donors in north-eastern Poland. Hepatol Res. 2005; 33(3): 206-10.	Podlaskie	2001	2001
Chlabicz S, Bonifatiuk I, Radziwon P. Prevalence of hepatitis C virus antibodies among blood donors in north-eastern Poland. Hepatol Res. 2005; 33(3): 206-10.	Podlaskie	2002	2002
Chlabicz S, Bonifatiuk I, Radziwon P. Prevalence of hepatitis C virus antibodies among blood donors in north-eastern Poland. Hepatol Res. 2005; 33(3): 206-10.	Podlaskie	2003	2003
Cho Y, Bonsu G, Akoto-Ampaw A, Nkrumah-Mills G, Nimo JJA, Park JK, Ki M. The prevalence and risk factors for hepatitis B surface ag positivity in pregnant women in eastern region of ghana. Gut Liver. 2012; 6(2): 23540.	Ghana	2008	2009
Chongsrisawat V, Thawornsuk N, Theamboonlers A, Louisirirotchanakul S, Poovorawan Y. Hepatitis B virus DNA in unusual serological profiles of hepatitis B surface antigen-positive sera. Viral Immunol. 2006; 19(4): 623-9.	Thailand	2004	2004
Choudhury N, Ramesh V, Saraswat S, Naik S. Effectiveness of mandatory transmissible diseases screening in Indian blood donors. Indian J Med Res. 1995; 229-32.	India	1993	1993
Choudhury N, Ramesh V, Saraswat S, Naik S. Effectiveness of mandatory transmissible diseases screening in Indian blood donors. Indian J Med Res. 1995; 229-32.	India	1997	1997
Chowdhury A, Santra A, Chakravorty R, Banerji A, Pal S, Dhali GK, Datta S, Banerji S, Manna B, Chowdhury SR, Bhattacharya SK, Mazumder DG. Community-based epidemiology of hepatitis B virus infection in West Bengal, India: prevalence of hepatitis B e antigen-negative infection and associated viral variants. J Gastroenterol Hepatol. 2005; 20(11): 1712-20.	India	2001	2002
Chrystie I, Sumner D, Palmer S, Kenney A, Banatvala J. Screening of pregnant women for evidence of current hepatitis B infection: selective or universal?. Health Trends. 1992; 24(1): 13-5.	England	1988	1989
Chrystie I, Sumner D, Palmer S, Kenney A, Banatvala J. Screening of pregnant women for evidence of current hepatitis B infection: selective or universal?. Health Trends. 1992; 24(1): 13-5.	England	1990	1990
Chu F-Y, Su F-H, Cheng S-H, Lin Y-S, Li C-Y, Chien C-C, Lin Y-C, Chiang S-Y. Hepatitis B surface antigen confirmatory testing for diagnosis of hepatitis B virus infection in Taiwan. J Med Virol. 2011; 83(9): 151421.	Taiwan (Province of China)	2008	2008
Chunsuttiwat S, Biggs BA, Maynard J, Thamapalo S, Laoboripat S, Bovornsin S, Charanasri U, Pinyowiwat W, Kunasol P. Integration of hepatitis B vaccination into the expanded programme on	Thailand	1988	1988

immunization in Chonburi and Chiangmai provinces, Thailand. Vaccine. 1997; 15(6-7): 769-74.			
Cisneros-Castolo M, Hernández-Ruiz L, Ibarra-Robles IE, Fernández-Gárate RH, Escobedo-De La Peña J. Prevalence of hepatitis B virus infection and related risk factors in a rural community of Mexico. Am J Trop Med Hyg. 2001; 65(6): 759-63.	Chihuahua	1997	1997
Coimbra Júnior CE, Santos RV, Yoshida CF, Baptista ML, Flowers NM, do Valle AC. Hepatitis B epidemiology and cultural practices in Amerindian populations of Amazonia: the Tupí-Mondé and the Xavánte from Brazil. Soc Sci Med. 1996; 42(12): 1735-43.	Brazil	1990	1990
Corrao G, Zambon A, Bagnardi V, Aricò S, Loguercio C, D'Amicis A, Collaborative SIDECIR Group. Nutrient intakes, nutritional patterns and the risk of liver cirrhosis: an explorative case-control study. Eur J Epidemiol. 2004; 19(9): 861-9.	Italy	1994	1998
Cunha L, Plouzeau C, Ingrand P, Gudo JPS, Ingrand I, Mondlane J, Beauchant M, Agius G. Use of replacement blood donors to study the epidemiology of major blood-borne viruses in the general population of Maputo, Mozambique. J Med Virol. 2007; 79(12): 1832-40.	Mozambique	2004	2004
Cunningham R, Northwood JL, Kelly CD, Boxall EH, Andrews NJ. Routine antenatal screening for hepatitis B using pooled sera: validation and review of 10 years experience. J Clin Pathol. 1998; 51(5): 392-5.	England	1986	1995
Cunningham R, Northwood JL, Kelly CD, Boxall EH, Andrews NJ. Routine antenatal screening for hepatitis B using pooled sera: validation and review of 10 years experience. J Clin Pathol. 1998; 51(5): 392-5.	England	1996	1996
Da Villa G, Andjaparidze A, Cauletti M, Franco E, Roggendorf M, Sepe A, Zaratti L. Viral hepatitis in the Bhutanese population: preliminary results of a seroepidemiological investigation. Res Virol. 1997; 148(2): 115-7.	Bhutan	1995	1996
Dai C-Y, Lin C-I, Yeh M-L, Hsieh M-H, Huang C-F, Hou N-J, Hsieh M-Y, Huang J-F, Lin Z-Y, Chen S-C, Wang L-Y, Chang W-Y, Chen J-S, Yu M-L, Chuang W-L. Association between gallbladder stones and chronic hepatitis C: ultrasonographic survey in a hepatitis C and B hyperendemic township in Taiwan. Kaohsiung J Med Sci. 2013; 29(8): 4305.	Taiwan (Province of China)	2010	2012
Dalekos GN, Zervou E, Merkouropoulos MH, Tsianos EV. Prevalence of hepatitis B and C viruses infection in chronic alcoholics with or without liver disease in Ioannina, Greece: low incidence of HCV infection. Eur J Epidemiol. 1996; 12(1): 21-5.	Greece	1994	1994
Dal-Ré R, Aguilar L, Coronel P. Current prevalence of hepatitis B, A and C in a healthy Spanish population. A seroepidemiological study. Infection. 1991; 19(6): 409-13.	Spain	1989	1989
Damale NKR, Lassey AT, Bekoe V. Hepatitis B virus seroprevalence among parturients in Accra, Ghana. Int J Gynaecol Obstet. 2005; 90(3): 240-1.	Ghana	2003	2003
Daramola OOM, George AO, Ogunbiyi AO, Otegbayo JA. Hepatitis B virus in Nigerians with lichen planus. West Afr J Med. 2004; 23(2): 104-6.	Oyo	2002	2002
D'Argenio P, Esposito D, Mele A, Ortolani G, Adamo B, Rapicetta M, Forte P, Pisani A, Soldo L, Sarrecchia B. Decline in the exposure to hepatitis A and B infections in children in Naples, Italy. Public Health. 1989; 103(5): 385-9.	Campania	1980	1980
D'Argenio P, Esposito D, Mele A, Ortolani G, Adamo B, Rapicetta M, Forte P, Pisani A, Soldo L, Sarrecchia B. Decline in the exposure	Campania	1988	1988

to hepatitis A and B infections in children in Naples, Italy. Public Health. 1989; 103(5): 385-9.			
Das PK, Harris VK, Shoma B, Bose YN, Annie S. Trend of hepatitis B virus infection in southern Indian blood donors. Indian J Gastroenterol. 1999; 18(4): 182.	India	1986	1990
Das PK, Harris VK, Shoma B, Bose YN, Annie S. Trend of hepatitis B virus infection in southern Indian blood donors. Indian J Gastroenterol. 1999; 18(4): 182.	India	1991	1994
Das PK, Harris VK, Shoma B, Bose YN, Annie S. Trend of hepatitis B virus infection in southern Indian blood donors. Indian J Gastroenterol. 1999; 18(4): 182.	India	1995	1999
Dawar M, Patrick DM, Bigham M, Cook D, Krajden M, Ng H. Impact of universal preadolescent vaccination against hepatitis B on antenatal seroprevalence of hepatitis B markers in British Columbia women. CMAJ. 2003; 168(6): 703-4.	Canada	1999	1999
De Souza NCN, Botelho CAO, Honer MR. Retrospective study of a pioneer antenatal screening program with 8,477 pregnant women in Brazil. Clin Exp Obstet Gynecol. 2004; 31(3): 217-20.	Brazil	2002	2003
Degertekin H, Tuzcu A, Yalçin K. Horizontal transmission of HBV infection among students in Turkey. Public Health. 2000; 114(5): 411-2.	Turkey	1997	1999
Del Pino N, Martínez Peralta L, Pampuro S, Pimentel E, Libonatti O. HTLV-I/II seroprevalence and coinfection with other pathogens in blood donors in Buenos Aires. J Acquir Immune Defic Syndr. 1994; 7(2): 206-7.	Argentina	1991	1992
Demirel Y, Duran B, Toktamis A, Erden O, Cetin M. Seroprevalence of syphilis, hepatitis B and C, and human immunodeficiency virus infections among women. Saudi Med J. 2004; 25(12): 2037-8.	Turkey	2002	2002
Demirtürk N, Demirdal T, Toprak D, Altindiş M, Aktepe OC. Hepatitis B and C virus in West-Central Turkey: seroprevalence in healthy individuals admitted to a university hospital for routine health checks. Turk J Gastroenterol. 2006; 17(4): 267-72.	Turkey	2002	2004
Denis F, Ranger-Rogez S, Alain S, Mounier M, Debrock C, Wagner A, Delpeyroux C, Tabaste JL, Aubard Y, Preux P-M. Screening of pregnant women for hepatitis B markers in a French Provincial University Hospital (Limoges) during 15 years. Eur J Epidemiol. 2004; 19(10): 973-8.	France	1984	1998
Denis F, Ranger-Rogez S, Alain S, Mounier M, Debrock C, Wagner A, Delpeyroux C, Tabaste JL, Aubard Y, Preux P-M. Screening of pregnant women for hepatitis B markers in a French Provincial University Hospital (Limoges) during 15 years. Eur J Epidemiol. 2004; 19(10): 973-8.	France	1985	1985
Denis F, Ranger-Rogez S, Alain S, Mounier M, Debrock C, Wagner A, Delpeyroux C, Tabaste JL, Aubard Y, Preux P-M. Screening of pregnant women for hepatitis B markers in a French Provincial University Hospital (Limoges) during 15 years. Eur J Epidemiol. 2004; 19(10): 973-8.	France	1986	1986
Denis F, Ranger-Rogez S, Alain S, Mounier M, Debrock C, Wagner A, Delpeyroux C, Tabaste JL, Aubard Y, Preux P-M. Screening of pregnant women for hepatitis B markers in a French Provincial University Hospital (Limoges) during 15 years. Eur J Epidemiol. 2004; 19(10): 973-8.	France	1987	1987
Denis F, Ranger-Rogez S, Alain S, Mounier M, Debrock C, Wagner A, Delpeyroux C, Tabaste JL, Aubard Y, Preux P-M. Screening of pregnant women for hepatitis B markers in a French Provincial	France	1988	1988

University Hospital (Limoges) during 15 years. Eur J Epidemiol. 2004; 19(10): 973-8.			
Denis F, Ranger-Rogez S, Alain S, Mounier M, Debrock C, Wagner A, Delpeyroux C, Tabaste JL, Aubard Y, Preux P-M. Screening of pregnant women for hepatitis B markers in a French Provincial University Hospital (Limoges) during 15 years. Eur J Epidemiol. 2004; 19(10): 973-8.	France	1989	1989
Denis F, Ranger-Rogez S, Alain S, Mounier M, Debrock C, Wagner A, Delpeyroux C, Tabaste JL, Aubard Y, Preux P-M. Screening of pregnant women for hepatitis B markers in a French Provincial University Hospital (Limoges) during 15 years. Eur J Epidemiol. 2004; 19(10): 973-8.	France	1990	1990
Denis F, Ranger-Rogez S, Alain S, Mounier M, Debrock C, Wagner A, Delpeyroux C, Tabaste JL, Aubard Y, Preux P-M. Screening of pregnant women for hepatitis B markers in a French Provincial University Hospital (Limoges) during 15 years. Eur J Epidemiol. 2004; 19(10): 973-8.	France	1991	1991
Denis F, Ranger-Rogez S, Alain S, Mounier M, Debrock C, Wagner A, Delpeyroux C, Tabaste JL, Aubard Y, Preux P-M. Screening of pregnant women for hepatitis B markers in a French Provincial University Hospital (Limoges) during 15 years. Eur J Epidemiol. 2004; 19(10): 973-8.	France	1992	1992
Denis F, Ranger-Rogez S, Alain S, Mounier M, Debrock C, Wagner A, Delpeyroux C, Tabaste JL, Aubard Y, Preux P-M. Screening of pregnant women for hepatitis B markers in a French Provincial University Hospital (Limoges) during 15 years. Eur J Epidemiol. 2004; 19(10): 973-8.	France	1993	1993
Denis F, Ranger-Rogez S, Alain S, Mounier M, Debrock C, Wagner A, Delpeyroux C, Tabaste JL, Aubard Y, Preux P-M. Screening of pregnant women for hepatitis B markers in a French Provincial University Hospital (Limoges) during 15 years. Eur J Epidemiol. 2004; 19(10): 973-8.	France	1994	1994
Denis F, Ranger-Rogez S, Alain S, Mounier M, Debrock C, Wagner A, Delpeyroux C, Tabaste JL, Aubard Y, Preux P-M. Screening of pregnant women for hepatitis B markers in a French Provincial University Hospital (Limoges) during 15 years. Eur J Epidemiol. 2004; 19(10): 973-8.	France	1995	1995
Denis F, Ranger-Rogez S, Alain S, Mounier M, Debrock C, Wagner A, Delpeyroux C, Tabaste JL, Aubard Y, Preux P-M. Screening of pregnant women for hepatitis B markers in a French Provincial University Hospital (Limoges) during 15 years. Eur J Epidemiol. 2004; 19(10): 973-8.	France	1996	1996
Denis F, Ranger-Rogez S, Alain S, Mounier M, Debrock C, Wagner A, Delpeyroux C, Tabaste JL, Aubard Y, Preux P-M. Screening of pregnant women for hepatitis B markers in a French Provincial University Hospital (Limoges) during 15 years. Eur J Epidemiol. 2004; 19(10): 973-8.	France	1997	1997
Denis F, Ranger-Rogez S, Alain S, Mounier M, Debrock C, Wagner A, Delpeyroux C, Tabaste JL, Aubard Y, Preux P-M. Screening of pregnant women for hepatitis B markers in a French Provincial University Hospital (Limoges) during 15 years. Eur J Epidemiol. 2004; 19(10): 973-8.	France	1998	1998
Dilek, Demir C, Bay A, Akdeniz H, Öner AF. Seropositivity rates of HBsAg, anti-HCV, anti-HIV and VDRL in blood donors in Eastern Turkey. Turk J Haematol. 2007; 24(1): 4-7.	Turkey	1995	2003

Division of Reproductive Health, Centers for Disease Control and Prevention (CDC), Ministry of Health (Uganda). Uganda AIDS Indicator Survey 2004-2005.	Uganda	2004	2005
Domínguez A, Bruguera M, Vidal J, Plans P, Salleras L. Changes in the seroepidemiology of hepatitis B infection in Catalonia 1989-1996. Vaccine. 2000; 18(22): 2345-50.	Spain	1996	1996
Dongdem JT, Kampo S, Soyiri IN, Asebga PN, Ziem JB, Sagoe K. Prevalence of hepatitis B virus infection among blood donors at the Tamale Teaching Hospital, Ghana (2009). BMC Res Notes. 2012; 5: 115.	Ghana	2009	2009
Dos Santos JI, Lopes MA, Deliège-Vasconcelos E, Couto-Fernandez JC, Patel BN, Barreto ML, Ferreira Júnior OC, Galvão-Castro B. Seroprevalence of HIV, HTLV-I/II and other perinatally-transmitted pathogens in Salvador, Bahia. Rev Inst Med Trop Sao Paulo. 1995; 37(4): 343-8.	Bahia	1990	1991
Dounias G, Kypraiou E, Rachiotis G, Tsovili E, Kostopoulos S. Prevalence of hepatitis B virus markers in municipal solid waste workers in Keratsini (Greece). Occup Med (Lond). 2005; 55(1): 60-3.	Greece	1999	2001
Dunford L, Carr MJ, Dean J, Nguyen LT, Ta Thi TH, Nguyen BT, Connell J, Coughlan S, Nguyen HT, Hall WW, Thi LAN. A multicentre molecular analysis of hepatitis B and blood-borne virus coinfections in Viet Nam. PLoS One. 2012; 7(6): e39027.	Viet Nam	2008	2009
Duong TH, Nguyen PH, Henley K, Peters M. Risk factors for hepatitis B infection in rural Vietnam. Asian Pac J Cancer Prev. 2009; 10(1): 97-102.	Viet Nam	2006	2006
Dupinay T, Restorp K, Leutscher P, Rousset D, Chemin I, Migliani R, Magnius L, Norder H. High prevalence of hepatitis B virus genotype E in Northern Madagascar indicates a West-African lineage. J Med Virol. 2010; 82(9): 151526.	Madagascar	2007	2009
Dupont A, Delaporte E, Jego JM, Schrijvers D, Merlin M, Josse R. Prevalence of hepatitis B antigen among randomized representative urban and rural populations in Gabon. Ann Soc Belg Med Trop. 1988; 68(2): 157-8.	Gabon	1986	1987
Elefsiniotis IS, Glynou I, Brokalaki H, Magaziotou I, Pantazis KD, Fotiou A, Liosis G, Kada H, Saroglou G. Serological and virological profile of chronic HBV infected women at reproductive age in Greece. A two-year single center study. Eur J Obstet Gynecol Reprod Biol. 2007; 132(2): 200-3.	Greece	2003	2005
Elefsiniotis IS, Glynou I, Pantazis KD, Fotos NV, Magaziotou I, Kada H. Prevalence of chronic HBV infection among 13,581 women at reproductive age in Greece. A prospective single center study. J Clin Virol. 2005; 32(2): 179-80.	Greece	2003	2004
Eleftheriou A, Teloni F, Ioannou P. HDV infection in Cyprus. Prog Clin Biol Res. 1992; 277-85.	Cyprus	1986	1992
El-Magrahe H, Furarah AR, El-Figih K, El-Urshfany S, Ghenghesh KS. Maternal and neonatal seroprevalence of Hepatitis B surface antigen (HBsAg) in Tripoli, Libya. J Infect Dev Ctries. 2010; 4(3): 16870.	Libya	2001	2002
Elzouki AN, Smeo MN, Sammud M, Elahmer O, Daw M, Furarah A, Abudher A, Mohamed MK. Prevalence of hepatitis B and C virus infections and their related risk factors in Libya: a national seroepidemiological survey. East Mediterr Health J. 2013; 19(7): 589-99.	Libya	2005	2005

Erden S, Büyüköztürk S, Calangu S, Yilmaz G, Palanduz S, Badur S. A study of serological markers of hepatitis B and C viruses in Istanbul, Turkey. Med Princ Pract. 2003; 12(3): 184-8.	Turkey	1998	2001
Ertekin V, Selimoğlu MA, Altinkaynak S. Sero-epidemiology of hepatitis B infection in an urban paediatric population in Turkey. Public Health. 2003; 117(1): 49-53.	Turkey	2001	2001
Fomulu NJ, Morfaw FLI, Torimiro JN, Nana P, Koh MV, William T. Prevalence, correlates and pattern of Hepatitis B among antenatal clinic attenders in Yaounde-Cameroon: is perinatal transmission of HBV neglected in Cameroon?. BMC Pregnancy Childbirth. 2013; 13: 158.	Cameroon	2011	2012
Fritzsche C, Becker F, Hemmer CJ, Riebold D, Klammt S, Hufert F, Akam W, Kinge TN, Reisinger EC. Hepatitis B and C: neglected diseases among health care workers in Cameroon. Trans R Soc Trop Med Hyg. 2013; 107(3): 15864.	Cameroon	2011	2011
Fukuizumi K, Sata M, Suzuki H, Nakano H, Tanikawa K. Hepatitis C virus seroconversion rate in a hyperendemic area of HCV in Japan: a prospective study. Scand J Infect Dis. 1997; 29(4): 345-7.	Japan	1990	1997
Furusyo N, Hayashi J, Ariyama I, Sawayama Y, Etoh Y, Kashiwagi S. Lower hepatitis G virus infection prevalence compared to hepatitis B and C virus infection prevalences. Dig Dis Sci. 2000; 45(1): 188-95.	Japan	1996	1996
Furusyo N, Hayashi J, Kakuda K, Sawayama Y, Ariyama I, Eddie R, Kashiwagi S. Markedly high seroprevalence of hepatitis B virus infection in comparison to hepatitis C virus and human T lymphotropic virus type-1 infections in selected Solomon Islands populations. Am J Trop Med Hyg. 1999; 61(1): 85-91.	Solomon Islands	1994	1994
Gacche RN, Kaid AMS. Epidemiology of viral hepatitis B and C infections in ibb city, yemen. Hepat Mon. 2012; 12(7): 4602.	Yemen	2010	2010
Gangaidzo IT, Moyo VM, Khumalo H, Saungweme T, Gomo Z, Rouault T, Gordeuk VR. Hepatitis C virus in Zimbabwe. Cent Afr J Med. 1997; 43(5): 122-5.	Zimbabwe	1994	1994
Ganju SA, Goel A. Sero-surveillance of HIV, HBV and HCV infections in antenatal and STD clinic attendees. J Commun Dis. 2004; 36(1): 60-2.	India	1998	1998
Ganju SA, Goel A. Sero-surveillance of HIV, HBV and HCV infections in antenatal and STD clinic attendees. J Commun Dis. 2004; 36(1): 60-2.	India	1999	1999
Garcia-Fulgueiras A, Tormo MJ, Rodriguez T, Perez-Flores D, Chirlaque D, Navarro C. Prevalence of hepatitis B and C markers in the south-east of Spain: an unlinked community-based serosurvey of 2,203 adults. Scand J Infect Dis. 1996; 28(1): 17-20.	Spain	1992	1993
Garg S, Mathur DR, Garg DK. Comparison of seropositivity of HIV, HBV, HCV and syphilis in replacement and voluntary blood donors in western India. Indian J Pathol Microbiol. 2001; 44(4): 409-12.	India	1994	1999
Gessoni G, Manoni F. Prevalence of anti-hepatitis C virus antibodies among teenagers in the Venetian area: a seroepidemiological study. Eur J Med. 1993; 2(2): 79-82.	Veneto	1991	1991
Gholamreza R, Shahryar S, Abbasali K, Hamidreza J, Abdolvahab M, Khodaberdi K, Danyal R, Nafiseh A. Seroprevalence of hepatitis B virus and its co-infection with hepatitis D virus and hepatitis C virus in Iranian adult population. Indian J Med Sci. 2007; 61(5): 263-8.	Iran (Islamic Republic of)	2004	2005
Gidding HF, Warlow M, MacIntyre CR, Backhouse J, Gilbert GL, Quinn HE, McIntyre PB. The impact of a new universal infant and	Australia	2002	2002

school-based adolescent hepatitis B vaccination program in Australia. Vaccine. 2007; 25(51): 8637-41.			
Glasgow KW, Schabas R, Williams DC, Wallace E, Nalezyty LA. A population-based hepatitis B seroprevalence and risk factor study in a northern Ontario town. Can J Public Health. 1997; 88(2): 87-90.	Canada	1993	1993
Gogos CA, Fouka KP, Nikiforidis G, Avgeridis K, Sakellaropoulos G, Bassaris H, Maniatis A, Skoutelis A. Prevalence of hepatitis B and C virus infection in the general population and selected groups in South-Western Greece. Eur J Epidemiol. 2003; 18(6): 551-7.	Greece	1997	1998
Goh KT, Chan YW, Wong LY, Kong KH, Oon CJ, Guan R. The prevalence of hepatitis B virus markers in dental personnel in Singapore. Trans R Soc Trop Med Hyg. 1988; 82(6): 908-10.	Singapore	1986	1986
Goh KT, Kong KH, Heng BH, Oon CJ. Seroepidemiology of hepatitis A and hepatitis B virus infection in a Gurkha community in Singapore. J Med Virol. 1993; 41(2): 146-9.	Singapore	1991	1991
Goh KT. Prevention and control of hepatitis B virus infection in Singapore. Ann Acad Med Singapore. 1997; 26(5): 671-81.	Singapore	1978	1992
Goh KT. Prevention and control of hepatitis B virus infection in Singapore. Ann Acad Med Singapore. 1997; 26(5): 671-81.	Singapore	1983	1985
Goh KT. Prevention and control of hepatitis B virus infection in Singapore. Ann Acad Med Singapore. 1997; 26(5): 671-81.	Singapore	1987	1987
González L, Roses A, Alornar P, Del Valle JM, Garau A, Ferrer P, Mairnó M, Llinares R, Blanco I, Lardinois R. The maternal-infant center in the control of hepatitis B. Acta Obstet Gynecol Scand. 1988; 67(5): 421-7.	Spain	1984	1986
Grosheide PM, Wladimiroff JW, Heijtink RA, Mazel JA, Christiaens GC, Nuijten AS, Schalm SW. Proposal for routine antenatal screening at 14 weeks for hepatitis B surface antigen. Dutch Study Group on Prevention of Neonatal Hepatitis. BMJ. 1995; 311(7014): 1197-9.	Netherlands	1984	1985
Grosheide PM, Wladimiroff JW, Heijtink RA, Mazel JA, Christiaens GC, Nuijten AS, Schalm SW. Proposal for routine antenatal screening at 14 weeks for hepatitis B surface antigen. Dutch Study Group on Prevention of Neonatal Hepatitis. BMJ. 1995; 311(7014): 1197-9.	Netherlands	1985	1986
Grosheide PM, Wladimiroff JW, Heijtink RA, Mazel JA, Christiaens GC, Nuijten AS, Schalm SW. Proposal for routine antenatal screening at 14 weeks for hepatitis B surface antigen. Dutch Study Group on Prevention of Neonatal Hepatitis. BMJ. 1995; 311(7014): 1197-9.	Netherlands	1986	1987
Grosheide PM, Wladimiroff JW, Heijtink RA, Mazel JA, Christiaens GC, Nuijten AS, Schalm SW. Proposal for routine antenatal screening at 14 weeks for hepatitis B surface antigen. Dutch Study Group on Prevention of Neonatal Hepatitis. BMJ. 1995; 311(7014): 1197-9.	Netherlands	1987	1988
Grosheide PM, Wladimiroff JW, Heijtink RA, Mazel JA, Christiaens GC, Nuijten AS, Schalm SW. Proposal for routine antenatal screening at 14 weeks for hepatitis B surface antigen. Dutch Study Group on Prevention of Neonatal Hepatitis. BMJ. 1995; 311(7014): 1197-9.	Netherlands	1988	1989
Grossman DW, Hans LM, Glazier R. Geographic origin and risk for congenital infection in a Canadian inner city: findings and implications for policy. Can J Public Health. 1999; 90(6): 385-8.	Canada	1996	1997
Guariguata L, de Beer I, Hough R, Bindels E, Weimers-Maasdorp D, Feeley FG 3rd, de Wit TFR. Diabetes, HIV and other health determinants associated with absenteeism among formal sector workers in Namibia. BMC Public Health. 2012; 44.	Namibia	2009	2010
Gupta N, Kumar V, Kaur A. Seroprevalence of HIV, HBV, HCV and syphilis in voluntary blood donors. Indian J Med Sci. 2004; 58(6): 255-7.	India	2001	2003

Gurol E, Saban C, Oral O, Cigdem A, Armagan A. Trends in Hepatitis B and Hepatitis C Virus among Blood Donors over 16 Years in Turkey. Eur J Epidemiol. 2006; 21(4): 299-305.	Turkey	1989	2004
Habil FE, Mahdi WKM, Abdelwahab SF, Abdel-Hamid M. Hepatitis B virus genotype D predominates HBsAg-positive egyptian blood donors and is mainly associated with a negative HBeAg serostatus. Intervirology. 2013; 56(5): 27883.	Egypt	2006	2007
Hakim S, Kazmi S, Bagasra O. Seroprevalence of Hepatitis B and C Genotypes Among Young Apparently Healthy Females of Karachi-Pakistan. Libyan J Med. 2008; 3(2): 66-70.	Sindh	2002	2006
Halim NK, Madukwe U, Saheeb BD, Airauhi LU. Hepatitis B surface antigen and antibody to hepatitis C virus among accident and emergency patients. East Afr Med J. 2001; 78(9): 480-3.	Edo	1995	1995
Hansson BG, Hansson HB, Ohlin AK, Nordenfelt E. Screening for anti-HIV and HBsAg in pooled sera from a clinical chemistry section as a tool for epidemiological survey. Scand J Infect Dis. 1993; 25(3): 297-303.	Sweden	1988	1991
Hepburn I, Schade R, Babenko N. High prevalence of chronic hepatitis B among pregnant women in Ukraine: An opportunity lost. Hepatology. 2011; 54(S1): 590A-1A.	Ukraine (without Crimea & Sevastopol)	2008	2008
Hepburn I, Schade R, Babenko N. High prevalence of chronic hepatitis B among pregnant women in Ukraine: An opportunity lost. Hepatology. 2011; 54(S1): 590A-1A.	Ukraine (without Crimea & Sevastopol)	2009	2009
Hepburn I, Schade R, Babenko N. High prevalence of chronic hepatitis B among pregnant women in Ukraine: An opportunity lost. Hepatology. 2011; 54(S1): 590A-1A.	Ukraine (without Crimea & Sevastopol)	2010	2010
Hodges M, Sanders E, Aitken C. Seroprevalence of hepatitis markers; HAV, HBV, HCV and HEV amongst primary school children in Freetown, Sierra Leone. West Afr J Med. 1998; 17(1): 36-7.	Sierra Leone	1996	1996
Hussein E, Teruya J. Evaluation of blood supply operation and infectious disease markers in blood donors during the Egyptian revolution. Transfusion. 2012; 52(11): 23218.	Egypt	2010	2010
Hyams KC, al-Arabi MA, al-Tagani AA, Messiter JF, al-Gaali AA, George JF. Epidemiology of hepatitis B in the Gezira region of Sudan. Am J Trop Med Hyg. 1989; 40(2): 200-6.	Sudan	1986	1986
icddr,b. Impact of Hepatitis B vaccination programme in Bangladesh. Health Sci Bull. 2013; 2(1).	Bangladesh	2011	2012
Ikeme AC, Ezegwui HU, Ogbonna C. Sero prevalence of hepatitis B surface antigen (HBsAg) in pregnant women in Southeast Nigeria. Trop Doct. 2006; 36(2): 128.	Enugu	2000	2004
Ishida T, Takao S, Settheetham-Ishida W, Tiwawech D. Prevalence of hepatitis B and C virus infection in rural ethnic populations of Northern Thailand. J Clin Virol. 2002; 24(1-2): 31-5.	Thailand	1996	1998
Ismail SO, Ahmed HJ, Grillner L, Hederstedt B, Issa A, Bygdeman SM. Sexually transmitted diseases in men in Mogadishu, Somalia. Int J STD AIDS. 1990; 1(2): 102-6.	Somalia	1986	1986
Ito S, Yao DF, Nii C, Hibino S, Kamamura M, Nisikado T, Honda H, Shimizu I, Meng XY. Epidemiological characteristics of the incidence of hepatitis C virus (C100-3) antibodies in patients with liver diseases in the inshore area of the Yangtze River. J Gastroenterol Hepatol. 1993; 8(3): 232-7.	Jiangsu	1991	1992
Ivić I, Banović I, Bradarić N. Hepatitis B virus infection among pregnant women in Split region. Eur J Epidemiol. 1999; 15(6): 589-90.	Croatia	1995	1995

Jäger H, Nseka K, Goussard B, Kabeya C-M, Rauhaus G, Peyerl G, Salaun J-J, Korte R. Voluntary blood donor recruitment: a strategy to reduce transmission of HIV-1, hepatitis-B and syphilis in Kinshasa, Zaïre. Infusionstherapie. 1990; 17(4): 224-6.	Congo	1989	1989
Jain DC, Jain RK, Ichhpujani RL, Sharma RS. Prevalence of hepatitis B virus in pregnant women. J Commun Dis. 1994; 26(4): 233-4.	India	1992	1992
Jain RC, Soni SB. Detection of HBsAg and HIV carriage among blood donors or rural population of Loni areas. J Assoc Physicians India. 1995; 43(5): 378.	India	1993	1994
James L, Fong CW, Foong BH, Wee MK, Chow A, Shum E, Chew SK. Hepatitis B Seroprevalence Study 1999. Singapore Med J. 2001; 42(9): 420-4.	Singapore	1999	1999
Jang MK, Lee JY, Lee JH, Kim YB, Kim HY, Lee MS, Park CK, Yoo JY. Seroepidemiology of HBV infection in South Korea, 1995 through 1999. Korean J Intern Med. 2001; 16(3): 153-9.	Republic of Korea	1995	1995
Jang MK, Lee JY, Lee JH, Kim YB, Kim HY, Lee MS, Park CK, Yoo JY. Seroepidemiology of HBV infection in South Korea, 1995 through 1999. Korean J Intern Med. 2001; 16(3): 153-9.	Republic of Korea	1996	1996
Jang MK, Lee JY, Lee JH, Kim YB, Kim HY, Lee MS, Park CK, Yoo JY. Seroepidemiology of HBV infection in South Korea, 1995 through 1999. Korean J Intern Med. 2001; 16(3): 153-9.	Republic of Korea	1997	1997
Jang MK, Lee JY, Lee JH, Kim YB, Kim HY, Lee MS, Park CK, Yoo JY. Seroepidemiology of HBV infection in South Korea, 1995 through 1999. Korean J Intern Med. 2001; 16(3): 153-9.	Republic of Korea	1998	1998
Jang MK, Lee JY, Lee JH, Kim YB, Kim HY, Lee MS, Park CK, Yoo JY. Seroepidemiology of HBV infection in South Korea, 1995 through 1999. Korean J Intern Med. 2001; 16(3): 153-9.	Republic of Korea	1999	1999
Jelic O, Jelic D, Balen I, Jelic A, Jelic N, Mihaljevic I. Prevalence of markers of hepatitis B virus infection among the general population of the municipality of Slavonski Brod. Acta Med Croatica. 1993; 48(3): 111-6.	Croatia	1990	1991
Jensen L, Heilmann C, Smith E, Wantzin P, Peitersen B, Weber T, Krogsgaard K. Efficacy of selective antenatal screening for hepatitis B among pregnant women in Denmark: is selective screening still an acceptable strategy in a low-endemicity country?. Scand J Infect Dis. 2003; 35(6-7): 378-82.	Denmark	2000	2001
Jilg W, Hottenträger B, Weinberger K, Schlottmann K, Frick E, Holstege A, Schölmerich J, Palitzsch KD. Prevalence of markers of hepatitis B in the adult German population. J Med Virol. 2001; 63(2): 96-102.	Germany	1999	1999
Juárez-Figueroa LA, Uribe-Salas FJ, Conde-González CJ, Sánchez-Alemán MÁ. Marcadores serológicos de hepatitis B y C, y VIH en La Calera y Cuambio, Guerrero, México. Salud Publica Mex. 2011; S32-S36.	Guerrero	2004	2004
Jutavijittum P, Yousukh A, Samountry B, Samountry K, Ounavong A, Thammavong T, Keokhamphue J, Toriyama K. Seroprevalence of hepatitis B and C virus infections among Lao blood donors. Southeast Asian J Trop Med Public Health. 2007; 38(4): 674-9.	Lao People's Democratic Republic	2003	2003
Jutavijittum P, Yousukh A, Samountry B, Samountry K, Ounavong A, Thammavong T, Keokhamphue J, Toriyama K. Seroprevalence of hepatitis B and C virus infections among Lao blood donors. Southeast Asian J Trop Med Public Health. 2007; 38(4): 674-9.	Lao People's Democratic Republic	2004	2004
Jutavijittum P, Yousukh A, Samountry B, Samountry K, Ounavong A, Thammavong T, Keokhamphue J, Toriyama K. Seroprevalence of	Lao People's Democratic Republic	2005	2005

hepatitis B and C virus infections among Lao blood donors. Southeast Asian J Trop Med Public Health. 2007; 38(4): 674-9.			
Kakkar N, Kaur R, Dhanoa J. Voluntary donors-need for a second look. Indian J Pathol Microbiol. 2004; 47(3): 381-3.	India	2001	2003
Kangin M, Turhanoglu M, Gulsun S, Cakabay B. Seroprevalence of Hepatitis B and C among Children in Endemic Areas of Turkey. Hepat Mon. 2010; 10(1): 36-41.	Turkey	2005	2008
Kanra G, Tezcan S, Badur S, Turkish National Study Team. Hepatitis B and measles seroprevalence among Turkish children. Turk J Pediatr. 2005; 47(2): 105-10.	Turkey	1998	1998
Kapur S, Mittal A. Incidence of HIV infection & its predictors in blood donors in Delhi. Indian J Med Res. 1998; 45-50.	India	1989	1997
Karabay O, Serin E, Tamer A, Gökdoğan F, Alpteker H, Ozcan A, Gündüz H. Hepatitis B carriage and Brucella seroprevalence in urban and rural areas of Bolu province of Turkey: a prospective epidemiologic study. Turk J Gastroenterol. 2004; 15(1): 11-3.	Turkey	2003	2003
Karatekin G, Kilinç M, Gulcan Öksuz B, Iğde M. Hepatitis B seroprevalence in children and women and the impact of the hepatitis B vaccination program in the Black Sea Region of Turkey. J Infect Dev Ctries. 2013; 7(12): 960-5.	Turkey	2007	2009
Karim R. Seroprevalence of Hepatitis B - surface antigen among non-professional blood donors in selected hospital in Dhaka city. Retrovirology. 2010; 7(S1): 52-53.	Bangladesh	2007	2009
Karimi M, Ghavanini AA. Seroprevalence of HBsAg, anti-HCV, and anti-HIV among haemophiliac patients in Shiraz, Iran. Haematologia (Budap). 2001; 31(3): 251-5.	Iran (Islamic Republic of)	1999	2000
Kashiwagi S, Hayashi J, Nomura H, Kajiyama W, Ikematsu H, Noguchi A. Changing pattern of intrafamilial transmission of hepatitis B virus in Okinawa, Japan. Am J Epidemiol. 1988; 127(4): 783-7.	Japan	1985	1985
Kefene H, Rapicetta M, Rossi GB, Bisanti L, Bekura D, Morace G, Palladino P, Di Rienzo A, Conti S, Bassani F, Bertolaso G, Pasquini P. Ethiopian national hepatitis B study. J Med Virol. 1988; 24(1): 75-84.	Ethiopia	1985	1986
Kfutwah AK, Tejiokem MC, Njouom R. A low proportion of HBeAg among HBsAg-positive pregnant women with known HIV status could suggest low perinatal transmission of HBV in Cameroon. Virol J. 2012; 9: 62.	Cameroon	2000	2000
Khan NR, Sadiq F. Prenatal screening for hepatitis B virus. Int J Gynaecol Obstet. 1996; 55(1): 79-80.	Punjab	1994	1994
Kim O, Kim S-S, Park M-S, Suh S-D, Lee M-W, Kim K-S, Yoon J-D, Lee J-S. Seroprevalence of sexually transmitted viruses in Korean populations including HIV-seropositive individuals. Int J STD AIDS. 2003; 14(1): 46-9.	Republic of Korea	2000	2000
King SD, Dodd RY, Haynes G, Wynter HH, Sullivan MT, Serjeant GR, Choo-Kang E, Michael E. Prevalence of antibodies to hepatitis C virus and other markers in Jamaica. West Indian Med J. 1995; 44(2): 55-7.	Jamaica	1991	1991
Kitano M, Sakaguchi K, Miyashita M, Mouri H, Senoh T, Nishimura M, Ohta T, Fujio K, Shimomura H, Tsuji T. Prevalence of hepatitis G virus (HGV) infection in an endemic area of hepatitis C virus (HCV) infection. Hepatogastroenterology. 2000; 47(35): 1340-2.	Japan	1996	1996
Kiyosawa K, Oofusa H, Saitoh H, Sodeyama T, Tanaka E, Furuta S, Itoh S, Ogata H, Kobuchi H, Kameko M. Seroepidemiology of hepatitis A, B, and D viruses and human T-lymphocyte tropic viruses in Japanese drug abusers. J Med Virol. 1989; 29(3): 160-3.	Japan	1988	1988

Komas NP, Baï-Sepou S, Manirakiza A, Léal J, Béré A, Le Faou A. The prevalence of hepatitis B virus markers in a cohort of students in Bangui, Central African Republic. BMC Infect Dis. 2010; 10: 226.	Central African Republic	2007	2007
Komas NP, Vickos U, Hübschen JM, Béré A, Manirakiza A, Muller CP, Le Faou A. Cross-sectional study of hepatitis B virus infection in rural communities, Central African Republic. BMC Infect Dis. 2013; 13: 286.	Central African Republic	2007	2008
Koné MC, Sidibé ET, Mallé KK, Beye SA, Lurton G, Dao S, Diarra MT, Dao S. Seroprevalence of human immunodeficiency virus, hepatitis B virus and hepatitis C virus among blood donors in Segou, Mali. Med Sante Trop. 2012; 22(1): 978.	Mali	2007	2010
Kothari A, Ramachandran VG, Gupta P, Singh B, Talwar V. Seroprevalence of cytomegalovirus among voluntary blood donors in Delhi, India. J Health Popul Nutr. 2002; 20(4): 348-51.	India	2000	2000
Kumar D, Arora A, Singh NP, Kohli R, Kar P, Das BC. Hepatitis G virus infection in hemodialysis patients from urban Delhi. Ren Fail. 2005; 27(1): 87-93.	India	2003	2003
Kumar H, Gupta PK, Jaiprakash M. The Role of anti-HBc IgM in Screening of Blood Donors. Med J Armed Forces India. 2007; 63(4): 350-2.	India	2005	2005
Kuperan P, Choon AT, Ding SH, Lee G. Prevalence of antibodies to hepatitis C virus in relation to surrogate markers in a blood donor population of Singapore. Southeast Asian J Trop Med Public Health. 1993; 127-9.	Singapore	1991	1991
Kurçer M, Pehlivan E. Hepatitis B seroprevalance and risk factors in urban areas of Malatya. Turk J Gastroenterol. 2002; 13(1): 1-5.	Turkey	1997	1997
Kurien T, Thyagarajan SP, Jeyaseelan L, Peedicayil A, Rajendran P, Sivaram S, Hansdak SG, Renu G, Krishnamurthy P, Sudhakar K, Varghese JC, STD Study Group. Community prevalence of hepatitis B infection and modes of transmission in Tamil Nadu, India. Indian J Med Res. 2005; 121(5): 670-5.	India	1998	1998
Kuyucu N, Dökmen A, Yöney A, Teziç T. Seroprevalence of hepatitis B infection in Turkish children. Infection. 1998; 26(5): 317-8.	Turkey	1996	1996
Kyriakis KP, Foudoulaki LE, Papoulia EI, Sofroniadou KE. Seroprevalence of hepatitis B surface antigen (HBsAg) among first-time and sporadic blood donors in Greece: 1991-1996. Transfus Med. 2000; 10(3): 175-80.	Greece	1991	1996
Larke RP, Froese GJ, Devine RD, Petruk MW. Extension of the epidemiology of hepatitis B in circumpolar regions through a comprehensive serologic study in the Northwest Territories of Canada. J Med Virol. 1987; 22(3): 269-76.	Canada	1983	1985
Lee J-J, Lin M-Y, Yang Y-H, Lu S-N, Chen H-C, Hwang S-J. Association of hepatitis C and B virus infection with CKD in an endemic area in Taiwan: a cross-sectional study. Am J Kidney Dis. 2010; 56(1): 2331.	Taiwan (Province of China)	2004	2004
Li W-C, Lee Y-Y, Chen I-C, Sun C, Chiu F-H, Chuang C-H. Association between the hepatitis B and C viruses and metabolic diseases in patients stratified by age. Liver Int. 2013; 33(8): 1194202.	Taiwan (Province of China)	2008	2010
Liang X, Bi S, Yang W, Wang L, Cui G, Cui F, Zhang Y, Liu J, Gong X, Chen Y, Wang F, Zheng H, Wang F, Guo J, Jia Z, Ma J, Wang H, Luo H, Li L, Jin S, Hadler SC, Wang Y. Reprint of: Epidemiological serosurvey of Hepatitis B in China--declining HBV prevalence due to Hepatitis B vaccination. Vaccine. 2013; J21-28.	China	2005	2007
Liang X1, Bi S, Yang W, Wang L, Cui G, Cui F, Zhang Y, Liu J, Gong X, Chen Y, Wang F, Zheng H, Wang F, Guo J, Jia Z, Ma J,	Anhui	2006	2006

Wang H, Luo H, Li L, Jin S, Hadler SC, Wang Y. Epidemiological serosurvey of hepatitis B in China--declining HBV prevalence due to hepatitis B vaccination. Vaccine. 2009; 5(27): 6550-7.			
Liang X1, Bi S, Yang W, Wang L, Cui G, Cui F, Zhang Y, Liu J, Gong X, Chen Y, Wang F, Zheng H, Wang F, Guo J, Jia Z, Ma J, Wang H, Luo H, Li L, Jin S, Hadler SC, Wang Y. Epidemiological serosurvey of hepatitis B in China--declining HBV prevalence due to hepatitis B vaccination. Vaccine. 2009; 5(27): 6550-7.	Beijing	2006	2006
Liang X1, Bi S, Yang W, Wang L, Cui G, Cui F, Zhang Y, Liu J, Gong X, Chen Y, Wang F, Zheng H, Wang F, Guo J, Jia Z, Ma J, Wang H, Luo H, Li L, Jin S, Hadler SC, Wang Y. Epidemiological serosurvey of hepatitis B in China--declining HBV prevalence due to hepatitis B vaccination. Vaccine. 2009; 5(27): 6550-7.	Chongqing	2006	2006
Liang X1, Bi S, Yang W, Wang L, Cui G, Cui F, Zhang Y, Liu J, Gong X, Chen Y, Wang F, Zheng H, Wang F, Guo J, Jia Z, Ma J, Wang H, Luo H, Li L, Jin S, Hadler SC, Wang Y. Epidemiological serosurvey of hepatitis B in China--declining HBV prevalence due to hepatitis B vaccination. Vaccine. 2009; 5(27): 6550-7.	Fujian	2006	2006
Liang X1, Bi S, Yang W, Wang L, Cui G, Cui F, Zhang Y, Liu J, Gong X, Chen Y, Wang F, Zheng H, Wang F, Guo J, Jia Z, Ma J, Wang H, Luo H, Li L, Jin S, Hadler SC, Wang Y. Epidemiological serosurvey of hepatitis B in China--declining HBV prevalence due to hepatitis B vaccination. Vaccine. 2009; 5(27): 6550-7.	Gansu	2006	2006
Liang X1, Bi S, Yang W, Wang L, Cui G, Cui F, Zhang Y, Liu J, Gong X, Chen Y, Wang F, Zheng H, Wang F, Guo J, Jia Z, Ma J, Wang H, Luo H, Li L, Jin S, Hadler SC, Wang Y. Epidemiological serosurvey of hepatitis B in China--declining HBV prevalence due to hepatitis B vaccination. Vaccine. 2009; 5(27): 6550-7.	Guangdong	2006	2006
Liang X1, Bi S, Yang W, Wang L, Cui G, Cui F, Zhang Y, Liu J, Gong X, Chen Y, Wang F, Zheng H, Wang F, Guo J, Jia Z, Ma J, Wang H, Luo H, Li L, Jin S, Hadler SC, Wang Y. Epidemiological serosurvey of hepatitis B in China--declining HBV prevalence due to hepatitis B vaccination. Vaccine. 2009; 5(27): 6550-7.	Guangxi	2006	2006
Liang X1, Bi S, Yang W, Wang L, Cui G, Cui F, Zhang Y, Liu J, Gong X, Chen Y, Wang F, Zheng H, Wang F, Guo J, Jia Z, Ma J, Wang H, Luo H, Li L, Jin S, Hadler SC, Wang Y. Epidemiological serosurvey of hepatitis B in China--declining HBV prevalence due to hepatitis B vaccination. Vaccine. 2009; 5(27): 6550-7.	Guizhou	2006	2006
Liang X1, Bi S, Yang W, Wang L, Cui G, Cui F, Zhang Y, Liu J, Gong X, Chen Y, Wang F, Zheng H, Wang F, Guo J, Jia Z, Ma J, Wang H, Luo H, Li L, Jin S, Hadler SC, Wang Y. Epidemiological serosurvey of hepatitis B in China--declining HBV prevalence due to hepatitis B vaccination. Vaccine. 2009; 5(27): 6550-7.	Hainan	2006	2006
Liang X1, Bi S, Yang W, Wang L, Cui G, Cui F, Zhang Y, Liu J, Gong X, Chen Y, Wang F, Zheng H, Wang F, Guo J, Jia Z, Ma J, Wang H, Luo H, Li L, Jin S, Hadler SC, Wang Y. Epidemiological serosurvey of hepatitis B in China--declining HBV prevalence due to hepatitis B vaccination. Vaccine. 2009; 5(27): 6550-7.	Hebei	2006	2006
Liang X1, Bi S, Yang W, Wang L, Cui G, Cui F, Zhang Y, Liu J, Gong X, Chen Y, Wang F, Zheng H, Wang F, Guo J, Jia Z, Ma J, Wang H, Luo H, Li L, Jin S, Hadler SC, Wang Y. Epidemiological serosurvey of hepatitis B in China--declining HBV prevalence due to hepatitis B vaccination. Vaccine. 2009; 5(27): 6550-7.	Heilongjiang	2006	2006
Liang X1, Bi S, Yang W, Wang L, Cui G, Cui F, Zhang Y, Liu J, Gong X, Chen Y, Wang F, Zheng H, Wang F, Guo J, Jia Z, Ma J,	Henan	2006	2006

Wang H, Luo H, Li L, Jin S, Hadler SC, Wang Y. Epidemiological serosurvey of hepatitis B in China--declining HBV prevalence due to hepatitis B vaccination. Vaccine. 2009; 5(27): 6550-7.			
Liang X1, Bi S, Yang W, Wang L, Cui G, Cui F, Zhang Y, Liu J, Gong X, Chen Y, Wang F, Zheng H, Wang F, Guo J, Jia Z, Ma J, Wang H, Luo H, Li L, Jin S, Hadler SC, Wang Y. Epidemiological serosurvey of hepatitis B in China--declining HBV prevalence due to hepatitis B vaccination. Vaccine. 2009; 5(27): 6550-7.	Hubei	2006	2006
Liang X1, Bi S, Yang W, Wang L, Cui G, Cui F, Zhang Y, Liu J, Gong X, Chen Y, Wang F, Zheng H, Wang F, Guo J, Jia Z, Ma J, Wang H, Luo H, Li L, Jin S, Hadler SC, Wang Y. Epidemiological serosurvey of hepatitis B in China--declining HBV prevalence due to hepatitis B vaccination. Vaccine. 2009; 5(27): 6550-7.	Hunan	2006	2006
Liang X1, Bi S, Yang W, Wang L, Cui G, Cui F, Zhang Y, Liu J, Gong X, Chen Y, Wang F, Zheng H, Wang F, Guo J, Jia Z, Ma J, Wang H, Luo H, Li L, Jin S, Hadler SC, Wang Y. Epidemiological serosurvey of hepatitis B in China--declining HBV prevalence due to hepatitis B vaccination. Vaccine. 2009; 5(27): 6550-7.	Inner Mongolia	2006	2006
Liang X1, Bi S, Yang W, Wang L, Cui G, Cui F, Zhang Y, Liu J, Gong X, Chen Y, Wang F, Zheng H, Wang F, Guo J, Jia Z, Ma J, Wang H, Luo H, Li L, Jin S, Hadler SC, Wang Y. Epidemiological serosurvey of hepatitis B in China--declining HBV prevalence due to hepatitis B vaccination. Vaccine. 2009; 5(27): 6550-7.	Jiangsu	2006	2006
Liang X1, Bi S, Yang W, Wang L, Cui G, Cui F, Zhang Y, Liu J, Gong X, Chen Y, Wang F, Zheng H, Wang F, Guo J, Jia Z, Ma J, Wang H, Luo H, Li L, Jin S, Hadler SC, Wang Y. Epidemiological serosurvey of hepatitis B in China--declining HBV prevalence due to hepatitis B vaccination. Vaccine. 2009; 5(27): 6550-7.	Jiangxi	2006	2006
Liang X1, Bi S, Yang W, Wang L, Cui G, Cui F, Zhang Y, Liu J, Gong X, Chen Y, Wang F, Zheng H, Wang F, Guo J, Jia Z, Ma J, Wang H, Luo H, Li L, Jin S, Hadler SC, Wang Y. Epidemiological serosurvey of hepatitis B in China--declining HBV prevalence due to hepatitis B vaccination. Vaccine. 2009; 5(27): 6550-7.	Jilin	2006	2006
Liang X1, Bi S, Yang W, Wang L, Cui G, Cui F, Zhang Y, Liu J, Gong X, Chen Y, Wang F, Zheng H, Wang F, Guo J, Jia Z, Ma J, Wang H, Luo H, Li L, Jin S, Hadler SC, Wang Y. Epidemiological serosurvey of hepatitis B in China--declining HBV prevalence due to hepatitis B vaccination. Vaccine. 2009; 5(27): 6550-7.	Liaoning	2006	2006
Liang X1, Bi S, Yang W, Wang L, Cui G, Cui F, Zhang Y, Liu J, Gong X, Chen Y, Wang F, Zheng H, Wang F, Guo J, Jia Z, Ma J, Wang H, Luo H, Li L, Jin S, Hadler SC, Wang Y. Epidemiological serosurvey of hepatitis B in China--declining HBV prevalence due to hepatitis B vaccination. Vaccine. 2009; 5(27): 6550-7.	Ningxia	2006	2006
Liang X1, Bi S, Yang W, Wang L, Cui G, Cui F, Zhang Y, Liu J, Gong X, Chen Y, Wang F, Zheng H, Wang F, Guo J, Jia Z, Ma J, Wang H, Luo H, Li L, Jin S, Hadler SC, Wang Y. Epidemiological serosurvey of hepatitis B in China--declining HBV prevalence due to hepatitis B vaccination. Vaccine. 2009; 5(27): 6550-7.	Qinghai	2006	2006
Liang X1, Bi S, Yang W, Wang L, Cui G, Cui F, Zhang Y, Liu J, Gong X, Chen Y, Wang F, Zheng H, Wang F, Guo J, Jia Z, Ma J, Wang H, Luo H, Li L, Jin S, Hadler SC, Wang Y. Epidemiological serosurvey of hepatitis B in China--declining HBV prevalence due to hepatitis B vaccination. Vaccine. 2009; 5(27): 6550-7.	Shaanxi	2006	2006
Liang X1, Bi S, Yang W, Wang L, Cui G, Cui F, Zhang Y, Liu J, Gong X, Chen Y, Wang F, Zheng H, Wang F, Guo J, Jia Z, Ma J,	Shandong	2006	2006

Wang H, Luo H, Li L, Jin S, Hadler SC, Wang Y. Epidemiological serosurvey of hepatitis B in China--declining HBV prevalence due to hepatitis B vaccination. Vaccine. 2009; 5(27): 6550-7.			
Liang X1, Bi S, Yang W, Wang L, Cui G, Cui F, Zhang Y, Liu J, Gong X, Chen Y, Wang F, Zheng H, Wang F, Guo J, Jia Z, Ma J, Wang H, Luo H, Li L, Jin S, Hadler SC, Wang Y. Epidemiological serosurvey of hepatitis B in China--declining HBV prevalence due to hepatitis B vaccination. Vaccine. 2009; 5(27): 6550-7.	Shanghai	2006	2006
Liang X1, Bi S, Yang W, Wang L, Cui G, Cui F, Zhang Y, Liu J, Gong X, Chen Y, Wang F, Zheng H, Wang F, Guo J, Jia Z, Ma J, Wang H, Luo H, Li L, Jin S, Hadler SC, Wang Y. Epidemiological serosurvey of hepatitis B in China--declining HBV prevalence due to hepatitis B vaccination. Vaccine. 2009; 5(27): 6550-7.	Shanxi	2006	2006
Liang X1, Bi S, Yang W, Wang L, Cui G, Cui F, Zhang Y, Liu J, Gong X, Chen Y, Wang F, Zheng H, Wang F, Guo J, Jia Z, Ma J, Wang H, Luo H, Li L, Jin S, Hadler SC, Wang Y. Epidemiological serosurvey of hepatitis B in China--declining HBV prevalence due to hepatitis B vaccination. Vaccine. 2009; 5(27): 6550-7.	Sichuan	2006	2006
Liang X1, Bi S, Yang W, Wang L, Cui G, Cui F, Zhang Y, Liu J, Gong X, Chen Y, Wang F, Zheng H, Wang F, Guo J, Jia Z, Ma J, Wang H, Luo H, Li L, Jin S, Hadler SC, Wang Y. Epidemiological serosurvey of hepatitis B in China--declining HBV prevalence due to hepatitis B vaccination. Vaccine. 2009; 5(27): 6550-7.	Tianjin	2006	2006
Liang X1, Bi S, Yang W, Wang L, Cui G, Cui F, Zhang Y, Liu J, Gong X, Chen Y, Wang F, Zheng H, Wang F, Guo J, Jia Z, Ma J, Wang H, Luo H, Li L, Jin S, Hadler SC, Wang Y. Epidemiological serosurvey of hepatitis B in China--declining HBV prevalence due to hepatitis B vaccination. Vaccine. 2009; 5(27): 6550-7.	Tibet	2006	2006
Liang X1, Bi S, Yang W, Wang L, Cui G, Cui F, Zhang Y, Liu J, Gong X, Chen Y, Wang F, Zheng H, Wang F, Guo J, Jia Z, Ma J, Wang H, Luo H, Li L, Jin S, Hadler SC, Wang Y. Epidemiological serosurvey of hepatitis B in China--declining HBV prevalence due to hepatitis B vaccination. Vaccine. 2009; 5(27): 6550-7.	Xinjiang	2006	2006
Liang X1, Bi S, Yang W, Wang L, Cui G, Cui F, Zhang Y, Liu J, Gong X, Chen Y, Wang F, Zheng H, Wang F, Guo J, Jia Z, Ma J, Wang H, Luo H, Li L, Jin S, Hadler SC, Wang Y. Epidemiological serosurvey of hepatitis B in China--declining HBV prevalence due to hepatitis B vaccination. Vaccine. 2009; 5(27): 6550-7.	Yunnan	2006	2006
Liang X1, Bi S, Yang W, Wang L, Cui G, Cui F, Zhang Y, Liu J, Gong X, Chen Y, Wang F, Zheng H, Wang F, Guo J, Jia Z, Ma J, Wang H, Luo H, Li L, Jin S, Hadler SC, Wang Y. Epidemiological serosurvey of hepatitis B in China--declining HBV prevalence due to hepatitis B vaccination. Vaccine. 2009; 5(27): 6550-7.	Zhejiang	2006	2006
Lin C-C, Yang C-Y, Shih C-T, Chen B-H, Huang Y-L. Waning immunity and booster responses in nursing and medical technology students who had received plasma-derived or recombinant hepatitis B vaccine during infancy. Am J Infect Control. 2011; 39(5): 40814.	Taiwan (Province of China)	2007	2008
Lionis C, Frangoulis E, Koulentakis M, Biziagos E, Kouroumalis E. Current prevalence of hepatitis A, B, and C markers in school children of a rural area of Crete, Greece. J Viral Hepat. 1997; 4(1): 55-61.	Greece	1993	1993
Lionis C, Frangoulis E, Koulentakis M, Biziagos E, Kouroumalis E. Current prevalence of hepatitis A, B, and C markers in school children of a rural area of Crete, Greece. J Viral Hepat. 1997; 4(1): 55-61.	Greece	1993	1994

Lionis C, Frangoulis E, Koulentakis M, Biziagos E, Kouroumalis E. Current prevalence of hepatitis A, B, and C markers in school children of a rural area of Crete, Greece. J Viral Hepat. 1997; 4(1): 55-61.	Greece	1994	1995
Liu J-L, Chen J-Y, Chen C-T, Wang J-H, Lin C-Y, Chen P-F, Hung C-H, Kee K-M, Lee C-M, Tsai L-S, Chen S-C, Lin S-C, Lu S-N. Community-based cross-sectional study: the association of lipids with hepatitis C seropositivity and diabetes mellitus. J Gastroenterol Hepatol. 2012; 27(11): 168894.	Taiwan (Province of China)	2004	2004
López-Izquierdo R, Antonia Udaondo Ma, Zarzosa P, García-Ramón E, Garcinuño S, Ángel Bratos M, Orduña A, Rodríguez-Torres A, Almaraz A. Seroprevalencia de las hepatitis virales en población general representativa de una zona básica de salud urbana en Castilla y León. Enferm Infecc Microbiol Clin. 2007; 25(5): 317-23.	Spain	2003	2003
Louie M, Low DE, Feinman SV, McLaughlin B, Simor AE. Prevalence of bloodborne infective agents among people admitted to a Canadian hospital. CMAJ. 1992; 146(8): 1331-4.	Canada	1990	1990
Louisirirotchanakul S, Myint KSA, Srimee B, Kanoksinsombat C, Khamboonruang C, Kunstadter P, Wasi C. The prevalence of viral hepatitis among the Hmong people of northern Thailand. Southeast Asian J Trop Med Public Health. 2002; 33(4): 837-44.	Thailand	2000	2000
Mabayoje VO, Oparinde DP, Akanni EO, Taiwo SS, Muhibi MA, Adebayo TO. Seroprevalence of hepatitis B and C and of human immunodeficiency virus among blood donors in south-west Nigeria. Br J Biomed Sci. 2007; 64(4): 177-9.	Oyo	2004	2005
MacLean AB, Cameron S, Follett EAC. Prevalence of hepatitis B and C viruses and human immunodeficiency virus infections in women of reproductive age. Br J Obstet Gynaecol. 1993; 100(7): 702-3.	England	1991	1991
Maio G, d' Argenio P, Stroffolini T, Bozza A, Sacco L, Tosti ME, Intorcia M, Fossi E, d' Alessio G, Kondili LA, Rapicetta M, Mele A. Hepatitis C virus infection and alanine transaminase levels in the general population: a survey in a southern Italian town. J Hepatol. 2000; 33(1): 116-20.	Campania	1997	1997
Maio G, d' Argenio P, Stroffolini T, Bozza A, Sacco L, Tosti ME, Intorcia M, Fossi E, d' Alessio G, Kondili LA, Rapicetta M, Mele A. Hepatitis C virus infection and alanine transaminase levels in the general population: a survey in a southern Italian town. J Hepatol. 2000; 33(1): 116-20.	Sicilia	1999	2000
Makroo RN, Hassain G, Koul A, Shah GN. Prevalence of hepatitis B surface antigen in Kashmiri blood donors. Indian J Med Res. 1989; 310-3.	India	1985	1986
Makroo RN, Hassain G, Koul A, Shah GN. Prevalence of hepatitis B surface antigen in Kashmiri blood donors. Indian J Med Res. 1989; 310-3.	India	1985	1989
Makuwa M, Souqui□re S, Telfer P, Apetrei C, Vray M, Bedjabaga I, Mouinga-Ondeme A, Onanga R, Marx PA, Kazanji M, Roques P, Simon F. Identification of hepatitis B virus subgenotype A3 in rural Gabon. J Med Virol. 2006; 78(9): 1175-84.	Gabon	2001	2001
Mamadou S, Ide M, Maazou ARA, Aoula B, Labo S, Bozari M. HIV infection and hepatitis B seroprevalence among antenatal clinic attendees in Niger, West Africa. HIV AIDS (Auckl). 2012; 4: 14.	Niger	2008	2008
Mansour W, Malick F-ZF, Sidiya A, Ishagh E, Chekaraou MA, Veillon P, Ducancelle A, Brichler S, Le Gal F, Lo B, Gordien E, Lunel-Fabiani F. Prevalence, risk factors, and molecular epidemiology of hepatitis B and hepatitis delta virus in pregnant	Mauritania	2008	2009

women and in patients in Mauritania. J Med Virol. 2012; 84(8): 118698.			
Marion SA, Tomm Pastore M, Pi DW, Mathias RG. Long-term follow-up of hepatitis B vaccine in infants of carrier mothers. Am J Epidemiol. 1994; 140(8): 734-46.	Canada	1984	1989
Marranconi F, Fabris P, Stecca C, Zampieri L, Bettini MC, Di Fabrizio N, de Lalla F. Prevalence of anti-HCV and risk factors for hepatitis C virus infection in healthy pregnant women. Infection. 1994; 22(5): 333-7.	Veneto	1992	1992
Martinson FE, Weigle KA, Mushahwar IK, Weber DJ, Royce R, Lemon SM. Seroepidemiological survey of hepatitis B and C virus infections in Ghanaian children. J Med Virol. 1996; 48(3): 278-83.	Ghana	1993	1995
Masuet-Aumatell C, Ramon-Torrell JM, Casanova-Rituerto A, Banqué-Navarro M, Dávalos-Gamboa MDR, Rodríguez SLM. Seroprevalence of hepatitis B in two period birth cohorts of Bolivian children: effect of universal vaccination. Trans R Soc Trop Med Hyg. 2013; 107(9): 57883.	Bolivia (Plurinational State of)	2010	2010
Mazzei C, Imberciadori G, Saccone F, Durante C, Mattiauda M, Lavagna G, Barberis G, Cavagnaro G. Infectious disease markers in autologous blood. Transfusion. 1989; 29(9): 829-30.	Liguria	1987	1987
Mehmet D, Meliksah E, Serif Y, Gunay S, Tuncer O, Zeynep S. Prevalence of hepatitis B infection in the southeastern region of Turkey: comparison of risk factors for HBV infection in rural and urban areas. Jpn J Infect Dis. 2005; 58(1): 15-9.	Turkey	2003	2003
Miédougé M, Chatelut M, Mansuy J-M, Rostaing L, Malecaze F, Sandres-Sauné K, Boudet F, Puel J, Abbal M, Izopet J. Screening of blood from potential organ and cornea donors for viruses. J Med Virol. 2002; 66(4): 571-5.	France	1999	2001
Miller WC, Shao JF, Weaver DJ, Shimokura GH, Paul DA, Lallinger GJ. Seroprevalence of viral hepatitis in Tanzanian adults. Trop Med Int Health. 1998; 3(9): 757-63.	United Republic of Tanzania	1992	1992
Molijn MH, van der Linden JM, Ko LK, Gorgels J, Hop W, van Rhenen DJ. Risk factors and anti-HBc reactivity among first time blood donors. Vox Sang. 1997; 72(4): 207-10.	Netherlands	1992	1995
Mollah AH, Nahar N, Siddique MA, Anwar KS, Hassan T, Azam MG. Common transfusion-transmitted infectious agents among thalassaemic children in Bangladesh. J Health Popul Nutr. 2003; 21(1): 67-71.	Bangladesh	2000	2001
Moore DJ, Bucens MR, Holman CD, Ott AK, Wells JI. Prenatal screening for markers of hepatitis B in aboriginal mothers resident in non-metropolitan Western Australia. Med J Aust. 1987; 147(11-12): 557-8.	Australia	1983	1985
Morris BA, Sabetti L. Prenatal screening for hepatitis B surface antigen. Is universal screening necessary?. Can Fam Physician. 1993; 61-4.	Canada	1990	1990
Mosendane T, Kew MC, Osih R, Mahomed A. Nurses at risk for occupationally acquired blood-borne virus infection at a South African academic hospital. S Afr Med J. 2012; 102(3 Pt 1): 1536.	Gauteng	2008	2008
Msuya SE, Mbizvo EM, Hussain A, Sam NE, Stray-Pedersen B. Seroprevalence of hepatitis B and C viruses among women of childbearing age in Moshi Urban, Tanzania. East Afr Med J. 2006; 83(2): 91-4.	United Republic of Tanzania	1999	1999
Muro FJ, Fiorillo SP, Sakasaka P, Odhiambo C, Reddy EA, Cunningham CK, Buchanan AM. Seroprevalence of Hepatitis B and	United Republic of Tanzania	2006	2008

C Viruses Among Children in Kilimanjaro Region, Tanzania. J Pediatr Infect Dis Soc. 2013.			
Muselmani W, Habbal W, Monem F. Significance of screening antibodies to hepatitis B virus core antigen among Syrian blood donors. Transfus Med. 2013; 23(4): 2658.	Syrian Arab Republic	2011	2011
Mwangi JW. Viral markers in a blood donor population. East Afr Med J. 1999; 76(1): 35-7.	Kenya	1995	1995
Mwangi JW. Viral markers in a blood donor population. East Afr Med J. 1999; 76(1): 35-7.	Kenya	1996	1996
Mwangi JW. Viral markers in a blood donor population. East Afr Med J. 1999; 76(1): 35-7.	Kenya	1997	1997
Mwangi JW. Viral markers in a blood donor population. East Afr Med J. 1999; 76(1): 35-7.	Kenya	1998	1998
Myo-Khin null, San-San-Oo null, Oo KM, Shimono K, Koide N, Okada S. Prevalence and factors associated with hepatitis C virus infection among Myanmar blood donors. Acta Med Okayama. 2010; 64(5): 31721.	Myanmar	2005	2007
Nagalo BM, Bisseye C, Sanou M, Kienou K, Nebie YK, Kiba A, Dahourou H, Ouattara S, Nikiema JB, Moret R, Zongo JD, Simpore, J. Seroprevalence and incidence of transfusion-transmitted infectious diseases among blood donors from regional blood transfusion centres in Burkina Faso, West Africa. Trop Med Int Health. 2012; 17(2): 247-53.	Burkina Faso	2009	2009
Nakata S, Song P, Duc DD, Nguyen XQ, Murata K, Tsuda F, Okamoto H. Hepatitis C and B virus infections in populations at low or high risk in Ho Chi Minh and Hanoi, Vietnam. J Gastroenterol Hepatol. 1994; 9(4): 416-9.	Viet Nam	1993	1993
Nandi J, Bhawalkar V, Mody H, Elavia A, Desai PK, Banerjee K. Detection of HIV-1, HBV and HCV antibodies in blood donors from Surat, western India. Vox Sang. 1994; 67(4): 406-7.	India	1992	1992
Nas T, Taner MZ, Yildiz A. Seroprevalence of syphilis, human immunodeficiency virus type-1, and hepatitis B virus infections among pregnant women in Turkey. Int J Gynaecol Obstet. 1999; 66(2): 171-2.	Turkey	1997	1997
Nasidi A, Harry TO, Vyazov SO, Munube GM, Azzan BB, Ananiev VA. Prevalence of hepatitis B infection markers in representative areas of Nigeria. Int J Epidemiol. 1986; 15(2): 274-6.	Lagos	1984	1984
Nasidi A, Harry TO, Vyazov SO, Munube GM, Azzan BB, Ananiev VA. Prevalence of hepatitis B infection markers in representative areas of Nigeria. Int J Epidemiol. 1986; 15(2): 274-6.	Bauchi	1984	1984
National Center for Health Statistics (NCHS), Centers for Disease Control and Prevention (CDC). United States National Health and Nutrition Examination Survey 2005-2006. Hyattsville, United States: National Center for Health Statistics (NCHS), Centers for Disease Control and Prevention (CDC), 2007.	United States of America	2005	2006
National Center for Health Statistics (NCHS), Centers for Disease Control and Prevention (CDC). United States National Health and Nutrition Examination Survey 2005-2006. Hyattsville, United States: National Center for Health Statistics (NCHS), Centers for Disease Control and Prevention (CDC), 2007.	United States of America	2007	2008
National Center for Health Statistics (NCHS), Centers for Disease Control and Prevention (CDC). United States National Health and Nutrition Examination Survey 2005-2006. Hyattsville, United States: National Center for Health Statistics (NCHS), Centers for Disease Control and Prevention (CDC), 2007.	United States of America	2009	2010

Ndumbe PM, Skalsky J, Joller-Jemelka HI. Seroprevalence of hepatitis and HIV infection among rural pregnant women in Cameroon. APMIS. 1994; 102(9): 662-6.	Cameroon	1991	1992
Nelson KE, Suriyanon V, Taylor E, Wongchak T, Kingkeow C, Srirak N, Lertsrimongkol C, Cheewawat W, Celentano D. The incidence of HIV-1 infections in village populations of northern Thailand. AIDS. 1994; 8(7): 951-5.	Thailand	1990	1990
Ng KP, Ngeow YF, K R, M R. Hepatitis B seroprevalence among University of Malaya Students in the Post-universal Infant Vaccination Era. Med J Malaysia. 2013; 68(2): 144-7.	Southeast Asia	2005	2011
Ng KP, Saw TL, Baki A, Rozainah K, Pang KW, Ramanathan M. Impact of the Expanded Program of Immunization against hepatitis B infection in school children in Malaysia. Med Microbiol Immunol. 2005; 194(3): 163-8.	Malaysia	1997	1997
Ng KP, Saw TL, Baki A, Rozainah K, Pang KW, Ramanathan M. Impact of the Expanded Program of Immunization against hepatitis B infection in school children in Malaysia. Med Microbiol Immunol. 2005; 194(3): 163-8.	Malaysia	1998	1998
Ni Y-H, Chang M-H, Wu J-F, Hsu H-Y, Chen H-L, Chen D-S. Minimization of hepatitis B infection by a 25-year universal vaccination program. J Hepatol. 2012; 57(4): 7305.	Taiwan (Province of China)	2009	2009
Nkrumah B, Owusu M, Frempong HO, Averu P. Hepatitis B and C viral infections among blood donors from rural Ghana. Ghana Med J. 2011; 45(3): 97100.	Ghana	2006	2006
Nkrumah B, Owusu M, Frempong HO, Averu P. Hepatitis B and C viral infections among blood donors from rural Ghana. Ghana Med J. 2011; 45(3): 97100.	Ghana	2007	2007
Nkrumah B, Owusu M, Frempong HO, Averu P. Hepatitis B and C viral infections among blood donors from rural Ghana. Ghana Med J. 2011; 45(3): 97100.	Ghana	2008	2008
Noguchi A, Hayashi J, Nakashima K, Ikematsu H, Hirata M, Kashiwagi S. Decrease of hepatitis A and B virus infections in the population of Okinawa, Japan. J Infect. 1991; 23(3): 255–62.	Japan	1988	1988
Noubiap JJ, Joko WY, Nansseu JR, Tene UG, Siaka C. Sero-epidemiology of human immunodeficiency virus, hepatitis B and C viruses, and syphilis infections among first-time blood donors in Edea, Cameroon. Int J Infect Dis. 2013; 17(10): e832-7.	Cameroon	2011	2012
Nzaji MK, Ilunga BK. [A study of the prevalence of infectious markers in blood donors in rural areas. The case of Kamina hospital]. Sante Publique. 2013; 25(2): 213-7.	Democratic Republic of the Congo	2008	2008
Obi CL, Anyiwo CE, Nnatu SN, Agbonlahor DE, Esumeh FI, Karpas A. A comparison of human immunodeficiency virus (HIV) seropositivity and hepatitis B surface antigenemia (HBs Ag) among the same group of apparently healthy pregnant women in Lagos, Nigeria: a preliminary report. Viral Immunol. 1993; 6(1): 43-7.	Lagos	1991	1991
Ochi S, Onji M, Shiraishi K, Ohtu K, Akao T, Yano Y, Takei N, Matsui H, Ohta Y, Umeda M. Prevalence of hepatitis C virus antibody in an area endemic for hepatitis B virus and human T cell leukaemia virus. J Gastroenterol Hepatol. 1991; 6(6): 599-602.	Ehime	1990	1990
Ochola E, Ocama P, Orach CG, Nankinga ZK, Kalyango JN, McFarland W, Karamagi C. High burden of hepatitis B infection in Northern Uganda: results of a population-based survey. BMC Public Health. 2013; 13: 727.	Uganda	2010	2010
Ojo OS, Akonai AK, Thursz M, Ndububa DA, Durosinmi MA, Adeodu OO, Fatusi OA, Goldin RD. Hepatitis D virus antigen in	Osun	1995	1997

HBsAg positive chronic liver disease in Nigeria. East Afr Med J. 1998; 75(6): 329-31.			
Okoth F, Mbuthia J, Gatheru Z, Murila F, Kanyingi F, Mugo F, Esamai F, Alavi Z, Otieno J, Kiambati H, Wanjuki N. Seroprevalence of hepatitis B markers in pregnant women in Kenya. East Afr Med J. 2006; 83(9): 485-93.	Kenya	2001	2002
Okoth FA, Kobayashi M, Kaptich DC, Kaiguri PM, Tukei PM, Takayanagi T, Yamanaka T. Seroepidemiological study for HBV markers and anti-delta in Kenya. East Afr Med J. 1991; 68(7): 515-25.	Kenya	1986	1986
Okoth FA, Kobayashi M, Kaptich DC, Kaiguri PM, Tukei PM, Takayanagi T, Yamanaka T. Seroepidemiological study for HBV markers and anti-delta in Kenya. East Afr Med J. 1991; 68(7): 515-25.	Kenya	1986	1987
Okoth FA, Kobayashi M, Kaptich DC, Kaiguri PM, Tukei PM, Takayanagi T, Yamanaka T. Seroepidemiological study for HBV markers and anti-delta in Kenya. East Afr Med J. 1991; 68(7): 515-25.	Kenya	1987	1987
Oshitani H, Kasolo F, Tembo C, Mpabalwani M, Mizuta K, Luo N, Suzuki H, Numazaki Y. Hepatitis B virus infection among pregnant women in Zambia. East Afr Med J. 1995; 72(12): 813-5.	Zambia	1992	1993
O'Sullivan BG, Gidding HF, Law M, Kaldor JM, Gilbert GL, Dore GJ. Estimates of chronic hepatitis B virus infection in Australia, 2000. Aust N Z J Public Health. 2004; 28(3): 212-6.	Australia	1996	1999
Othman BM, Monem FS. Prevalence of hepatitis C virus antibodies among health care workers in Damascus, Syria. Saudi Med J. 2001; 22(7): 603-5.	Syrian Arab Republic	1999	1999
Othman BM, Monem FS. Prevalence of hepatitis C virus antibodies among intravenous drug abusers and prostitutes in Damascus, Syria. Saudi Med J. 2002; 23(4): 393-5.	Syrian Arab Republic	2000	2000
Ouwe-Missi-Oukem-Boyer O, Ndouo FST, Ollomo B, Mezui-Me-Ndong J, Noulin F, Lachard I, Ndong-Atome G-R, Makuwa M, Roques P, Branger M, Preux P-M, Mazier D, Bisser S. Hepatitis C virus infection may lead to slower emergence of P. falciparum in blood. PLoS One. 2011; 6(1): e16034.	Gabon	2003	2004
Ozsoy MF, Oncul O, Cavuslu S, Erdemoglu A, Emekdas G, Pahsa A. Seroprevalences of hepatitis B and C among health care workers in Turkey. J Viral Hepat. 2003; 10(2): 150-6.	Turkey	1998	2000
Padmasiri E, Rajapaksa L, Jayakuru WS, Withana N. The prevalence of hepatitis B surface antigen in the Gampaha district. Ceylon Med J. 1995; 40(1): 10-3.	Sri Lanka	1993	1993
Pahuja S, Sharma M, Baitha B, Jain M. Prevalence and trends of markers of hepatitis C virus, hepatitis B virus and human immunodeficiency virus in Delhi blood donors: a hospital based study. Jpn J Infect Dis. 2007; 60(6): 389-91.	India	2002	2002
Pahuja S, Sharma M, Baitha B, Jain M. Prevalence and trends of markers of hepatitis C virus, hepatitis B virus and human immunodeficiency virus in Delhi blood donors: a hospital based study. Jpn J Infect Dis. 2007; 60(6): 389-91.	India	2003	2003
Pahuja S, Sharma M, Baitha B, Jain M. Prevalence and trends of markers of hepatitis C virus, hepatitis B virus and human immunodeficiency virus in Delhi blood donors: a hospital based study. Jpn J Infect Dis. 2007; 60(6): 389-91.	India	2004	2004
Pahuja S, Sharma M, Baitha B, Jain M. Prevalence and trends of markers of hepatitis C virus, hepatitis B virus and human	India	2005	2005

immunodeficiency virus in Delhi blood donors: a hospital based study. Jpn J Infect Dis. 2007; 60(6): 389-91.			
Papaevangelou G, Róumeliotou A, Chatziminas M, Kotsianopoulou M, Ioannou P, Trichopoulou E, Nestoridou A. Epidemiological characteristics of hepatitis B virus infection in cyprus. Eur J Epidemiol. 1988; 4(2): 150-3.	Cyprus	1987	1987
Patterson F, Bumak J, Batey R. Changing prevalence of hepatitis B virus in urbanized Australian Aborigines. J Gastroenterol Hepatol. 1993; 8(5): 410-3.	Australia	1987	1988
Pawlotsky JM, B□lec L, Gr□senguet G, Deforges L, Bouvier M, Duval J, Dhumeaux D. High prevalence of hepatitis B, C, and E markers in young sexually active adults from the Central African Republic. J Med Virol. 1995; 46(3): 269-72.	Central African Republic	1993	1993
Pellissier G, Yazdanpanah Y, Adehossi E, Tosini W, Madougou B, Ibrahima K, Lolom I, Legac S, Rouveix E, Champenois K, Rabaud C, Bouvet E. Is universal HBV vaccination of healthcare workers a relevant strategy in developing endemic countries? The case of a university hospital in Niger. PLoS One. 2012; 7(9): e44442.	Niger	2009	2009
Pereira A, Sanz C, Tàssies D, Ramírez B. Do patient-related blood donors represent a threat to the safety of the blood supply?. Haematologica. 2002; 87(4): 427-33.	Spain	1996	2001
Perez OM, Morales W, Paniagua M, Strannegard O. Prevalence of antibodies to hepatitis A, B, C, and E viruses in a healthy population in Leon, Nicaragua. Am J Trop Med Hyg. 1996; 55(1): 17-21.	Nicaragua	1990	1992
Petrovic J, Salkic NN, Ahmetagic S, Stojic V, Mott-Divkovic S. Prevalence of chronic hepatitis B and hepatitis C among first time blood donors in Northeast Bosnia and Herzegovina: an estimate of prevalence in general population. Hepat Mon. 2011; 11(8): 62933.	Bosnia and Herzegovina	2009	2009
Poethko-Müller C, Zimmermann R, Hamouda O, Faber M, Stark K, Ross RS, Thamm M. [Epidemiology of hepatitis A, B, and C among adults in Germany: results of the German Health Interview and Examination Survey for Adults (DEGS1)]. Bundesgesundheitsblatt. 2013; 56(5-6): 707-15.	Germany	2008	2011
Puro V, Girardi E, Ippolito G, Lo Presti E, Benedetto A, Zaniratti S, Giannini V, Gioia C, Natili S, Tossini G. Prevalence of hepatitis B and C viruses and human immunodeficiency virus infections in women of reproductive age. Br J Obstet Gynaecol. 1992; 99(7): 598-600.	Lazio	1989	1990
Qamer S, Shahab T, Alam S, Malik A, Afzal K. Age-specific prevalence of hepatitis B surface antigen in pediatric population of Aligarh, North India. Indian J Pediatr. 2004; 71(11): 965-7.	India	2002	2003
Quinti I, Hassan NF, El Salman D, Shalaby H, El Zimatty D, Monier MK, Arthur RR. Hepatitis C virus-specific B cell activation: IgG and IgM detection in acute and chronic hepatitis C. J Hepatol. 1995; 23(6): 640-7.	Egypt	1992	1994
Quoilin S, Hutse V, Vandenberghe H, Claeys F, Verhaegen E, De Cock L, Van Loock F, Top G, Van Damme P, Vranckx R, Van Oyen H. A population-based prevalence study of hepatitis A, B and C virus using oral fluid in Flanders, Belgium. Eur J Epidemiol. 2007; 22(3): 195-202.	Belgium	2003	2003
Qureshi H, Bile KM, Jooma R, Alam SE, Afridi HUR. Prevalence of hepatitis B and C viral infections in Pakistan: findings of a national survey appealing for effective prevention and control measures. East Mediterr Health J. 2010; S15-23.	Balochistan	2007	2008

Qureshi H, Bile KM, Jooma R, Alam SE, Afridi HUR. Prevalence of hepatitis B and C viral infections in Pakistan: findings of a national survey appealing for effective prevention and control measures. East Mediterr Health J. 2010; S15-23.	Khyber Pakhtunkhwa	2007	2008
Qureshi H, Bile KM, Jooma R, Alam SE, Afridi HUR. Prevalence of hepatitis B and C viral infections in Pakistan: findings of a national survey appealing for effective prevention and control measures. East Mediterr Health J. 2010; S15-23.	Punjab	2007	2008
Qureshi H, Bile KM, Jooma R, Alam SE, Afridi HUR. Prevalence of hepatitis B and C viral infections in Pakistan: findings of a national survey appealing for effective prevention and control measures. East Mediterr Health J. 2010; S15-23.	Sindh	2007	2008
Raffaele A, Valenti M, Iovenitti M, Matani A, Bruno ML, Altobelli E, D'Alessandro A, Barnabei R, Leonardis B, Taglieri G. High prevalence of HCV infection among the general population in a rural area of central Italy. Eur J Epidemiol. 2001; 17(1): 41-6.	Abruzzo	1997	1997
Rai SK, Shibata H, Satoh M, Murakoso K, Sumi K, Kubo T, Matsuoka A. Seroprevalence of hepatitis B and C viruses in eastern Nepal. J Jpn Assoc Infec Dis. 1994; 68(12): 1492-7.	Nepal	1992	1992
Ramírez-Soto MC, Huichi-Atamari M, Aguilar-Ancori EG, Pezo-Ochoa JD. Seroprevalence of viral hepatitis B in university students in Abancay, Peru. Rev Peru Med Exp Salud Publica. 2011; 28(3): 5137.	Peru	2010	2010
Ramos JM, Belda S, Reyes F, Rodríguez JC, Royo G, Gutiérrez F. Prevalence of HIV, HBV, HCV, HTLV and Treponema pallidum among patients attending a rural hospital in Southern Ethiopia. J Clin Virol. 2012; 53(3): 268-9.	Oromia	2008	2008
Ramos JM, Belda S, Reyes F, Rodríguez JC, Royo G, Gutiérrez F. Prevalence of HIV, HBV, HCV, HTLV and Treponema pallidum among patients attending a rural hospital in Southern Ethiopia. J Clin Virol. 2012; 53(3): 268-9.	Oromia	2010	2010
Ratanasuwan W, Sonji A, Tiengrim S, Techasathit W, Suwanagool S. Serological survey of viral hepatitis A, B, and C at Thai Central Region and Bangkok: a population base study. Southeast Asian J Trop Med Public Health. 2004; 35(2): 416-20.	Thailand	2000	2002
Richard-Lenoble D, Traore O, Kombila M, Roingeard P, Dubois F, Goudeau A. Hepatitis B, C, D, and E markers in rural equatorial African villages (Gabon). Am J Trop Med Hyg. 1995; 53(4): 338-41.	Gabon	1991	1992
Richards AL, Perrault JG, Caringal LT, Manaloto CR, Sie A, Graham R, Ramos RM, Leonardo JB, Hyams KC. A non-invasive assessment of hepatitis B virus carrier status using saliva samples. Southeast Asian J Trop Med Public Health. 1996; 27(1): 80-4.	National Capital Region	1994	1994
Robinson T, Bullen C, Humphries W, Hornell J, Moyes C. The New Zealand Hepatitis B Screening Programme: screening coverage and prevalence of chronic hepatitis B infection. N Z Med J. 2005; 118(1211): U1345.	New Zealand	1999	2002
Ruzibakiev R, Kato H, Ueda R, Yuldasheva N, Hegay T, Avazova D, Kurbanov F, Zalalieva M, Tuichiev L, Achundjanov B, Mizokami M. Risk factors and seroprevalence of hepatitis B virus, hepatitis C virus, and human immunodeficiency virus infection in Uzbekistan. Intervirology. 2001; 44(6): 327-32.	Uzbekistan	1999	2000
Salawu L, Murainah HA. Pre-donation screening of intending blood donors for antibodies to infectious agents in a Nigerian tertiary health institution: a pilot study. Afr J Med Med Sci. 2006; 35(4): 453-6.	Osun	2003	2004

Sallam TA, Cuevas LE, Tong CYW. Increase in susceptibility of young adults to hepatitis B infection in the Republic of Yemen. Trans R Soc Trop Med Hyg. 2003; 97(3): 302-4.	Yemen	1999	2000
Sallam TA, Tong CYW, Cuevas LE, Raja'a YA, Othman AM, Al-Kharsa KR. Prevalence of blood-borne viral hepatitis in different communities in Yemen. Epidemiol Infect. 2003; 131(1): 771-5.	Yemen	2001	2001
Salleras L, Domínguez A, Bruguera M, Plans P, Costa J, Cardeñosa N, Batalla J, Plasència A. Declining prevalence of hepatitis B virus infection in Catalonia (Spain) 12 years after the introduction of universal vaccination. Vaccine. 2007; 25(52): 8726-31.	Spain	2002	2002
Sanaei-Zadeh H, Amoei M, Taghaddosinejad F. Seroprevalence of HIV, HBV and HCV in forensic autopsies, of presumed low risk, in Tehran, the capital of Iran. J Clin Forensic Med. 2002; 9(4): 179-81.	Iran (Islamic Republic of)	2000	2001
Sandesh K, Varghese T, Harikumar R, Beena P, Sasidharan VP, Bindu CS, Tony J, Harish K, Sunilkumar K, Ramachandran TM. Prevalence of Hepatitis B and C in the normal population and high risk groups in north Kerala. Trop Gastroenterol. 2006; 27(2): 80-3.	India	2002	2004
Santana Rodríguez OE, Malé Gil ML, HernándezSantana JF, Limiñana Cañal JM, Martín Sánchez AM. Prevalence of serologic markers of HBV, HDV, HCV and HIV in non-injection drug users compared to injection drug users in Gran Canaria, Spain. Eur J Epidemiol. 1998; 14(6): 555-61.	Spain	1993	1994
Sata M, Nakano H, Suzuki H, Noguchi S, Yamakawa Y, Tanaka E, Fukuizumi K, Tanaka K, Yoshida H, Tanikawa K. Sero-epidemiologic study of hepatitis C virus infection in Fukuoka, Japan. J Gastroenterol. 1998; 33(2): 218-22.	Japan	1990	1990
Satoskar A, Ray V. Prevalence of hepatitis B surface antigen (HBsAg) in blood donors from Bombay. Trop Geogr Med. 1992; 44(1-2): 119-21.	India	1990	1990
Sawaithul VK, Ukey PM, Bobhate SK. Prevalence of HIV infection among persons attending voluntary counseling and testing center, Nagpur. Biomed Res. 2006; 201-4.	India	2004	2004
Sawanpanyalert P, Boonmar S, Maeda T, Matsuura Y, Miyamura T. Risk factors for hepatitis C virus infection among blood donors in an HIV-epidemic area in Thailand. J Epidemiol Community Health. 1996; 50(2): 174-7.	Thailand	1994	1994
Sawayama Y, Hayashi J, Ariyama I, Furusyo N, Kawasaki T, Kawasaki M, Itoh K, Acharya GP, Kashiwagi S. A ten year serological survey of hepatitis A, B and C viruses infections in Nepal. J Epidemiol. 1999; 9(5): 350-4.	Nepal	1996	1996
Sbai A, Baha W, Ougabrai H, Allalia T, Dersi N, Lazaar F, Ennaji MM, Benjouad A, El Malki A, Hassar M, Benani A. Hepatitis B prevalence and risk factors in Morocco. Pathol Biol. 2012; 60(5): e65-69.	Morocco	2006	2009
Scott DA, Burans JP, al-Ouzeib HD, Arunkumar BK, al-Fadeel M, Nigad YR, al-Hadad A, Elyazeed RR, Hyams KC, Woody JN. A seroepidemiological survey of viral hepatitis in the Yemen Arab Republic. Trans R Soc Trop Med Hyg. 1990; 84(2): 288-91.	Yemen	1988	1988
Scott DA, Constantine NT, Callahan J, Burans JP, Olson JG, al-Fadeel M, al-Ozieb H, Arunkumer H, Hyams KC. The epidemiology of hepatitis C virus antibody in Yemen. Am J Trop Med Hyg. 1992; 46(1): 63-8.	Yemen	1988	1988
Seiji K, Inoue O, Liu SJ, Xu XP, Jin C, Cai SX, Nakatsuka H, Watanabe T, Uchida Y, Ikeda M. Prevalence of hepatitis B virus	Beijing	1988	1989

infection markers among factory workers in Beijing, China. Asia Pac J Public Health. 1991; 5(4): 345-9.			
Shalaby S, Kabbash IA, El Saleet G, Mansour N, Omar A, El Nawawy A. Hepatitis B and C viral infection: prevalence, knowledge, attitude and practice among barbers and clients in Gharbia governorate, Egypt. East Mediterr Health J. 2010; 16(1): 107.	Egypt	2007	2007
Sharma RR, Cheema R, Vajpayee M, Rao U, Kumar S, Marwaha N, Agnihotri SK. Prevalence of markers of transfusion transmissible diseases in voluntary and replacement blood donors. Natl Med J India. 2004; 17(1): 19-21.	India	1996	2002
Shimakawa Y, Lemoine M, Njai HF, et al. Community-based screening for hepatitis B virus infection in the Gambia, West Africa: Prevalence of infection and factors affecting the screening attendance. J Hepatol. 2013; 58(S1): S21-S21.	Gambia	2011	2012
Shimbo S, Zhang ZW, Qu JB, Wang JJ, Zhang CL, Song LH, Watanabe T, Higashikawa K, Ikeda M. Urban-rural comparison of HBV and HCV infection prevalence among adult women in Shandong Province, China. Southeast Asian J Trop Med Public Health. 1997; 28(3): 500-6.	Shandong	1996	1996
Shin HR, Kim JY, Kim JI, Lee DH, Yoo KY, Lee DS, Franceschi S. Hepatitis B and C virus prevalence in a rural area of South Korea: the role of acupuncture. Br J Cancer. 2002; 87(3): 314-8.	Republic of Korea	1991	1991
Shrestha SM, Subedi NB, Shrestha S, Maharjan KG, Tsuda F, Okamoto H. Epidemiology of hepatitis C virus infection in Nepal. Trop Gastroenterol. 1998; 19(3): 102-4.	Nepal	1994	1994
Shrestha SM, Subedi NB, Shrestha S, Maharjan KG, Tsuda F, Okamoto H. Epidemiology of hepatitis C virus infection in Nepal. Trop Gastroenterol. 1998; 19(3): 102-4.	Nepal	1994	1996
Shrestha SM, Subedi NB, Shrestha S, Maharjan KG, Tsuda F, Okamoto H. Epidemiology of hepatitis C virus infection in Nepal. Trop Gastroenterol. 1998; 19(3): 102-4.	Nepal	1996	1996
Shrestha SM. Seroepidemiology of hepatitis B in Nepal. J Commun Dis. 1990; 22(1): 27-32.	Nepal	1988	1988
Singh H, Aggarwal R, Singh RL, Naik SR, Naik S. Frequency of infection by hepatitis B virus and its surface mutants in a northern Indian population. Indian J Gastroenterol. 2003; 22(4): 132-7.	India	2002	2002
Singhvi A, Pulimood RB, John TJ, Babu PG, Samuel BU, Padankatti T, Carman RH. The prevalence of markers for hepatitis B and human immunodeficiency viruses, malarial parasites and microfilaria in blood donors in a large hospital in south India. J Trop Med Hyg. 1990; 93(3): 178-82.	India	1986	1988
Sirisena ND, Njoku MO, Idoko JA, Isamade E, Barau C, Jelpe D, Zamani A, Otowo S. Carriage rate of hepatitis-B surface antigen (HBsAg) in an urban community in Jos, Plateau State, Nigeria. Niger Postgrad Med J. 2002; 9(1): 7-10.	Plateau	1999	1999
Song P, Duc DD, Hien B, Nakata S, Chosa T, Watanabe J, Tsuda F, Murata K, Okamoto H. Markers of hepatitis C and B virus infections among blood donors in Ho Chi Minh City and Hanoi, Vietnam. Clin Diagn Lab Immunol. 1994; 1(4): 413-8.	Viet Nam	1992	1992
Songsivilai S, Jinathongthai S, Wongsena W, Tiangpitayakorn C, Dharakul T. High prevalence of hepatitis C infection among blood donors in northeastern Thailand. Am J Trop Med Hyg. 1997; 57(1): 66-9.	Thailand	1995	1995

Sonwane BR, Birare SD, Kulkarni PV. Prevalence of seroreactivity among blood donors in rural population. Indian J Med Sci. 2003; 57(9): 405-7.	India	1996	2001
Stabinski L, Reynolds SJ, Ocama P, Laeyendecker O, Serwadda D, Gray RH, Wawer M, Thomas DL, Quinn TC, Kirk GD. Hepatitis B virus and sexual behavior in Rakai, Uganda. J Med Virol. 2011; 83(5): 796-800.	Uganda	1998	1998
Stoetter L, Kasang C, Kalluvya S, et al. Prevalence of Hepatitis B and C among health care professionals in a tertiary hospital in Tanzania. Trop Med Int Health. 2013; 18(S1): 93-93.	United Republic of Tanzania	2010	2012
Stroffolini T, Guadagnino V, Chionne P, Procopio B, Mazzuca EG, Quintieri F, Scerbo P, Giancotti A, Nistic˜ S, Focˆ A, Tosti ME, Rapicetta M. A population based survey of hepatitis B virus infection in a southern Italian town. Ital J Gastroenterol Hepatol. 1997; 29(5): 415-8.	Calabria	1996	1996
Stroffolini T, Menchinelli M, Taliani G, Dambruoso V, Poliandri G, Bozza A, Lecce R, Clementi C, Ippolito FM, Compagnoni A. High prevalence of hepatitis C virus infection in a small central Italian town: lack of evidence of parenteral exposure. Ital J Gastroenterol. 1995; 27(5): 235-8.	Lazio	1994	1994
Stroffolini T, Rigo G, Collinassi P, Biffoni F. Prevalence of hepatitis B markers among teen-agers in Friuli. Boll Ist Sieroter Milan. 1990; 69(2): 455-7.	Friuli-Venezia Giulia	1989	1989
Sultan F, Mehmood T, Mahmood MT. Infectious pathogens in volunteer and replacement blood donors in Pakistan: a ten-year experience. Int J Infect Dis. 2007; 11(5): 407-12.	Punjab	1996	1996
Sultan F, Mehmood T, Mahmood MT. Infectious pathogens in volunteer and replacement blood donors in Pakistan: a ten-year experience. Int J Infect Dis. 2007; 11(5): 407-12.	Punjab	1997	1997
Sultan F, Mehmood T, Mahmood MT. Infectious pathogens in volunteer and replacement blood donors in Pakistan: a ten-year experience. Int J Infect Dis. 2007; 11(5): 407-12.	Punjab	1998	1998
Sultan F, Mehmood T, Mahmood MT. Infectious pathogens in volunteer and replacement blood donors in Pakistan: a ten-year experience. Int J Infect Dis. 2007; 11(5): 407-12.	Punjab	1999	1999
Sultan F, Mehmood T, Mahmood MT. Infectious pathogens in volunteer and replacement blood donors in Pakistan: a ten-year experience. Int J Infect Dis. 2007; 11(5): 407-12.	Punjab	2000	2000
Sultan F, Mehmood T, Mahmood MT. Infectious pathogens in volunteer and replacement blood donors in Pakistan: a ten-year experience. Int J Infect Dis. 2007; 11(5): 407-12.	Punjab	2001	2001
Sultan F, Mehmood T, Mahmood MT. Infectious pathogens in volunteer and replacement blood donors in Pakistan: a ten-year experience. Int J Infect Dis. 2007; 11(5): 407-12.	Punjab	2002	2002
Sultan F, Mehmood T, Mahmood MT. Infectious pathogens in volunteer and replacement blood donors in Pakistan: a ten-year experience. Int J Infect Dis. 2007; 11(5): 407-12.	Punjab	2003	2003
Sultan F, Mehmood T, Mahmood MT. Infectious pathogens in volunteer and replacement blood donors in Pakistan: a ten-year experience. Int J Infect Dis. 2007; 11(5): 407-12.	Punjab	2004	2004
Sultan F, Mehmood T, Mahmood MT. Infectious pathogens in volunteer and replacement blood donors in Pakistan: a ten-year experience. Int J Infect Dis. 2007; 11(5): 407-12.	Punjab	2005	2005
Takahashi M, Nishizawa T, Gotanda Y, Tsuda F, Komatsu F, Kawabata T, Hasegawa K, Altankhuu M, Chimedregzen U,	Mongolia	2002	2002

Narantuya L, Hoshino H, Hino K, Kagawa Y, Okamoto H. High prevalence of antibodies to hepatitis A and E viruses and viremia of hepatitis B, C, and D viruses among apparently healthy populations in Mongolia. Clin Diagn Lab Immunol. 2004; 11(2): 392-8.			
Tanaka E, Kiyosawa K, Sodeyama T, Hayata T, Ohike Y, Nakano Y, Yoshizawa K, Furuta S, Watanabe Y, Watanabe J, Nishioka K. Prevalence of antibody to hepatitis C virus in Japanese schoolchildren: comparison with adult blood donors. Am J Trop Med Hyg. 1992; 46(4): 460-4.	Japan	1986	1990
Tao QM, Wang Y, Wang H, Chen WR, Sun Y, Meng Q, Watanabe J, Nishioka K. Seroepidemiology of HCV and HBV infection in northern China. Gastroenterol Jpn. 1991; 156-8.	Beijing	1989	1989
Tareen S, Eslick GD, Kam EPY, Byles JE, Durrani AB, Maree SM. High prevalence of hepatitis B virus (HBV) among male blood donors in a developing country: urgent need for systematic screening. Scand J Infect Dis. 2002; 34(9): 712-3.	Balochistan	1996	1996
Tawk HM, Vickery K, Bisset L, Selby W, Cossart YE, Infection in Endoscopy Study Group. The impact of hepatitis B vaccination in a Western country: recall of vaccination and serological status in Australian adults. Vaccine. 2006; 24(8): 1095-106.	Australia	1999	2001
Tessema B, Yismaw G, Kassu A, Amsalu A, Mulu A, Emmrich F, Sack U. Seroprevalence of HIV, HBV, HCV and syphilis infections among blood donors at Gondar University Teaching Hospital, Northwest Ethiopia: declining trends over a period of five years. BMC Infect Dis. 2010; 10: 111.	Amhara	2003	2007
Thakur TS, Goyal A, Sharma V, Gupta ML, Singh S. Incidence of australia antigen (HBs Ag) in Himachal Pradesh. J Commun Dis. 1990; 22(3): 173-7.	India	1987	1989
Thakur TS, Sharma V, Goyal A, Gupta ML. Seroprevalence of HIV antibodies, Australia antigen and VDRL reactivity in Himachal Pradesh. Indian J Med Sci. 1991; 45(12): 332-5.	India	1987	1990
Tong D-Y, Wang X-H, Xu C-F, Yang Y-Z, Xiong S-D. Hepatitis B virus infection and coronary atherosclerosis: results from a population with relatively high prevalence of hepatitis B virus. World J Gastroenterol. 2005; 11(9): 1292-6.	Shanghai	2002	2004
Topuridze M, Butsashvili M, Kamkamidze G, Kajaia M, Morse D, McNutt LA. Barriers to hepatitis B vaccine coverage among healthcare workers in the Republic of Georgia: An international perspective. Infect Control Hosp Epidemiol. 2010; 31(2): 15864.	Georgia	2007	2007
Toukan AU, Sharaiha ZK, Abu-el -Rub OA, Hmoud MK, Dahbour SS, Abu-Hassan H, Yacoub SM, Hadler SC, Margolis HS, Coleman PJ. The epidemiology of hepatitis B virus among family members in the Middle East. Am J Epidemiol. 1990; 132(2): 220-32.	Jordan	1985	1985
Tresó B, Barcsay E, Tarján A, Horváth G, Dencs A, Hettmann A, Csépai MM, Gyori Z, Rusvai E, Takács M. Prevalence and correlates of HCV, HVB, and HIV infection among prison inmates and staff, Hungary. J Urban Health. 2012; 89(1): 10816.	Hungary	2007	2009
Tsega E, Mengesha B, Nordenfelt E, Hansson BG, Lindberg J. Prevalence of hepatitis B virus markers among Ethiopian blood donors: is HBsAg screening necessary?. Trop Geogr Med. 1987; 39(4): 336-40.	Ethiopia	1984	1985
Tsen YJ, Chang MH, Hsu HY, Lee CY, Sung JL, Chen DS. Seroprevalence of hepatitis B virus infection in children in Taipei, 1989: five years after a mass hepatitis B vaccination program. J Med Virol. 1991; 34(2): 96-9.	Taiwan (Province of China)	1989	1989

Tuinakelo LR, Tayler-Smith K, Khogali M, Marks GB. Prevalence of anaemia, syphilis and hepatitis B in pregnant women in Nausori, Fiji. Public Health Action. 2013; 3(1): 725.	Fiji	2011	2011
Turnour CE, Cretikos MA, Conaty SJ. Prevalence of chronic hepatitis B in South Western Sydney: evaluation of the country of birth method using maternal seroprevalence data. Aust N Z J Public Health. 2011; 35(1): 226.	Australia	2007	2009
Umolu PI, Okoror LE, Orhue P. Human immunodeficiency virus (HIV) seropositivity and hepatitis B surface antigenemia (HBSAG) among blood donors in Benin city, Edo state, Nigeria. Afr Health Sci. 2005; 5(1): 55-8.	Edo	2003	2003
Uneke CJ, Ogbu O, Inyama PU, Anyanwu GI, Njoku MO, Idoko JH. Prevalence of hepatitis-B surface antigen among blood donors and human immunodeficiency virus-infected patients in Jos, Nigeria. Mem Inst Oswaldo Cruz. 2005; 100(1): 13-6.	Plateau	1999	2002
Valdespino JL, Conde-González CJ, Olaíz-Fernández G, Palma O, Sepúlveda J. Prevalence in Mexico of infection and the carrier state of hepatitis B in adults. Salud Publica Mex 2007; 49(Supl 3): S404-S411. and Juárez-Figueroa LA, Uribe-Salas FJ, Conde-Glez CJ. Heterogeneous distribution of Hepatitis B serological markers in rural areas of Mexico. Salud Publica Mex 2011; 53(Supl 1): S26-S31. Mexico Hepatitis B Prevalence 1999-2000. [Unpublished].	Mexico	1999	2000
Valdespino JL, Conde-González CJ, Olaíz-Fernández G, Palma O, Sepúlveda J. Prevalence in Mexico of infection and the carrier state of hepatitis B in adults. Salud Publica Mex 2007; 49(Supl 3): S404-S411. and Juárez-Figueroa LA, Uribe-Salas FJ, Conde-Glez CJ. Heterogeneous distribution of Hepatitis B serological markers in rural areas of Mexico. Salud Publica Mex 2011; 53(Supl 1): S26-S31. Mexico Hepatitis B Prevalence 1999-2000. [Unpublished].	Aguascalientes	1999	2000
Valdespino JL, Conde-González CJ, Olaíz-Fernández G, Palma O, Sepúlveda J. Prevalence in Mexico of infection and the carrier state of hepatitis B in adults. Salud Publica Mex 2007; 49(Supl 3): S404-S411. and Juárez-Figueroa LA, Uribe-Salas FJ, Conde-Glez CJ. Heterogeneous distribution of Hepatitis B serological markers in rural areas of Mexico. Salud Publica Mex 2011; 53(Supl 1): S26-S31. Mexico Hepatitis B Prevalence 1999-2000. [Unpublished].	Baja California	1999	2000
Valdespino JL, Conde-González CJ, Olaíz-Fernández G, Palma O, Sepúlveda J. Prevalence in Mexico of infection and the carrier state of hepatitis B in adults. Salud Publica Mex 2007; 49(Supl 3): S404-S411. and Juárez-Figueroa LA, Uribe-Salas FJ, Conde-Glez CJ. Heterogeneous distribution of Hepatitis B serological markers in rural areas of Mexico. Salud Publica Mex 2011; 53(Supl 1): S26-S31. Mexico Hepatitis B Prevalence 1999-2000. [Unpublished].	Baja California Sur	1999	2000
Valdespino JL, Conde-González CJ, Olaíz-Fernández G, Palma O, Sepúlveda J. Prevalence in Mexico of infection and the carrier state of hepatitis B in adults. Salud Publica Mex 2007; 49(Supl 3): S404-S411. and Juárez-Figueroa LA, Uribe-Salas FJ, Conde-Glez CJ. Heterogeneous distribution of Hepatitis B serological markers in rural areas of Mexico. Salud Publica Mex 2011; 53(Supl 1): S26-S31. Mexico Hepatitis B Prevalence 1999-2000. [Unpublished].	Campeche	1999	2000
Valdespino JL, Conde-González CJ, Olaíz-Fernández G, Palma O, Sepúlveda J. Prevalence in Mexico of infection and the carrier state of hepatitis B in adults. Salud Publica Mex 2007; 49(Supl 3): S404-S411. and Juárez-Figueroa LA, Uribe-Salas FJ, Conde-Glez CJ. Heterogeneous distribution of Hepatitis B serological markers in rural	Coahuila	1999	2000

areas of Mexico. Salud Publica Mex 2011; 53(Supl 1): S26-S31. Mexico Hepatitis B Prevalence 1999-2000. [Unpublished].			
Valdespino JL, Conde-González CJ, Olaíz-Fernández G, Palma O, Sepúlveda J. Prevalence in Mexico of infection and the carrier state of hepatitis B in adults. Salud Publica Mex 2007; 49(Supl 3): S404-S411. and Juárez-Figueroa LA, Uribe-Salas FJ, Conde-Glez CJ. Heterogeneous distribution of Hepatitis B serological markers in rural areas of Mexico. Salud Publica Mex 2011; 53(Supl 1): S26-S31. Mexico Hepatitis B Prevalence 1999-2000. [Unpublished].	Colima	1999	2000
Valdespino JL, Conde-González CJ, Olaíz-Fernández G, Palma O, Sepúlveda J. Prevalence in Mexico of infection and the carrier state of hepatitis B in adults. Salud Publica Mex 2007; 49(Supl 3): S404-S411. and Juárez-Figueroa LA, Uribe-Salas FJ, Conde-Glez CJ. Heterogeneous distribution of Hepatitis B serological markers in rural areas of Mexico. Salud Publica Mex 2011; 53(Supl 1): S26-S31. Mexico Hepatitis B Prevalence 1999-2000. [Unpublished].	Chiapas	1999	2000
Valdespino JL, Conde-González CJ, Olaíz-Fernández G, Palma O, Sepúlveda J. Prevalence in Mexico of infection and the carrier state of hepatitis B in adults. Salud Publica Mex 2007; 49(Supl 3): S404-S411. and Juárez-Figueroa LA, Uribe-Salas FJ, Conde-Glez CJ. Heterogeneous distribution of Hepatitis B serological markers in rural areas of Mexico. Salud Publica Mex 2011; 53(Supl 1): S26-S31. Mexico Hepatitis B Prevalence 1999-2000. [Unpublished].	Chihuahua	1999	2000
Valdespino JL, Conde-González CJ, Olaíz-Fernández G, Palma O, Sepúlveda J. Prevalence in Mexico of infection and the carrier state of hepatitis B in adults. Salud Publica Mex 2007; 49(Supl 3): S404-S411. and Juárez-Figueroa LA, Uribe-Salas FJ, Conde-Glez CJ. Heterogeneous distribution of Hepatitis B serological markers in rural areas of Mexico. Salud Publica Mex 2011; 53(Supl 1): S26-S31. Mexico Hepatitis B Prevalence 1999-2000. [Unpublished].	Mexico City	1999	2000
Valdespino JL, Conde-González CJ, Olaíz-Fernández G, Palma O, Sepúlveda J. Prevalence in Mexico of infection and the carrier state of hepatitis B in adults. Salud Publica Mex 2007; 49(Supl 3): S404-S411. and Juárez-Figueroa LA, Uribe-Salas FJ, Conde-Glez CJ. Heterogeneous distribution of Hepatitis B serological markers in rural areas of Mexico. Salud Publica Mex 2011; 53(Supl 1): S26-S31. Mexico Hepatitis B Prevalence 1999-2000. [Unpublished].	Durango	1999	2000
Valdespino JL, Conde-González CJ, Olaíz-Fernández G, Palma O, Sepúlveda J. Prevalence in Mexico of infection and the carrier state of hepatitis B in adults. Salud Publica Mex 2007; 49(Supl 3): S404-S411. and Juárez-Figueroa LA, Uribe-Salas FJ, Conde-Glez CJ. Heterogeneous distribution of Hepatitis B serological markers in rural areas of Mexico. Salud Publica Mex 2011; 53(Supl 1): S26-S31. Mexico Hepatitis B Prevalence 1999-2000. [Unpublished].	Guanajuato	1999	2000
Valdespino JL, Conde-González CJ, Olaíz-Fernández G, Palma O, Sepúlveda J. Prevalence in Mexico of infection and the carrier state of hepatitis B in adults. Salud Publica Mex 2007; 49(Supl 3): S404-S411. and Juárez-Figueroa LA, Uribe-Salas FJ, Conde-Glez CJ. Heterogeneous distribution of Hepatitis B serological markers in rural areas of Mexico. Salud Publica Mex 2011; 53(Supl 1): S26-S31. Mexico Hepatitis B Prevalence 1999-2000. [Unpublished].	Guerrero	1999	2000
Valdespino JL, Conde-González CJ, Olaíz-Fernández G, Palma O, Sepúlveda J. Prevalence in Mexico of infection and the carrier state of hepatitis B in adults. Salud Publica Mex 2007; 49(Supl 3): S404-S411. and Juárez-Figueroa LA, Uribe-Salas FJ, Conde-Glez CJ.	Hidalgo	1999	2000

Heterogeneous distribution of Hepatitis B serological markers in rural areas of Mexico. Salud Publica Mex 2011; 53(Supl 1): S26-S31. Mexico Hepatitis B Prevalence 1999-2000. [Unpublished].			
Valdespino JL, Conde-González CJ, Olaíz-Fernández G, Palma O, Sepúlveda J. Prevalence in Mexico of infection and the carrier state of hepatitis B in adults. Salud Publica Mex 2007; 49(Supl 3): S404-S411. and Juárez-Figueroa LA, Uribe-Salas FJ, Conde-Glez CJ. Heterogeneous distribution of Hepatitis B serological markers in rural areas of Mexico. Salud Publica Mex 2011; 53(Supl 1): S26-S31. Mexico Hepatitis B Prevalence 1999-2000. [Unpublished].	Jalisco	1999	2000
Valdespino JL, Conde-González CJ, Olaíz-Fernández G, Palma O, Sepúlveda J. Prevalence in Mexico of infection and the carrier state of hepatitis B in adults. Salud Publica Mex 2007; 49(Supl 3): S404-S411. and Juárez-Figueroa LA, Uribe-Salas FJ, Conde-Glez CJ. Heterogeneous distribution of Hepatitis B serological markers in rural areas of Mexico. Salud Publica Mex 2011; 53(Supl 1): S26-S31. Mexico Hepatitis B Prevalence 1999-2000. [Unpublished].	México	1999	2000
Valdespino JL, Conde-González CJ, Olaíz-Fernández G, Palma O, Sepúlveda J. Prevalence in Mexico of infection and the carrier state of hepatitis B in adults. Salud Publica Mex 2007; 49(Supl 3): S404-S411. and Juárez-Figueroa LA, Uribe-Salas FJ, Conde-Glez CJ. Heterogeneous distribution of Hepatitis B serological markers in rural areas of Mexico. Salud Publica Mex 2011; 53(Supl 1): S26-S31. Mexico Hepatitis B Prevalence 1999-2000. [Unpublished].	Michoacán de Ocampo	1999	2000
Valdespino JL, Conde-González CJ, Olaíz-Fernández G, Palma O, Sepúlveda J. Prevalence in Mexico of infection and the carrier state of hepatitis B in adults. Salud Publica Mex 2007; 49(Supl 3): S404-S411. and Juárez-Figueroa LA, Uribe-Salas FJ, Conde-Glez CJ. Heterogeneous distribution of Hepatitis B serological markers in rural areas of Mexico. Salud Publica Mex 2011; 53(Supl 1): S26-S31. Mexico Hepatitis B Prevalence 1999-2000. [Unpublished].	Morelos	1999	2000
Valdespino JL, Conde-González CJ, Olaíz-Fernández G, Palma O, Sepúlveda J. Prevalence in Mexico of infection and the carrier state of hepatitis B in adults. Salud Publica Mex 2007; 49(Supl 3): S404-S411. and Juárez-Figueroa LA, Uribe-Salas FJ, Conde-Glez CJ. Heterogeneous distribution of Hepatitis B serological markers in rural areas of Mexico. Salud Publica Mex 2011; 53(Supl 1): S26-S31. Mexico Hepatitis B Prevalence 1999-2000. [Unpublished].	Nayarit	1999	2000
Valdespino JL, Conde-González CJ, Olaíz-Fernández G, Palma O, Sepúlveda J. Prevalence in Mexico of infection and the carrier state of hepatitis B in adults. Salud Publica Mex 2007; 49(Supl 3): S404-S411. and Juárez-Figueroa LA, Uribe-Salas FJ, Conde-Glez CJ. Heterogeneous distribution of Hepatitis B serological markers in rural areas of Mexico. Salud Publica Mex 2011; 53(Supl 1): S26-S31. Mexico Hepatitis B Prevalence 1999-2000. [Unpublished].	Nuevo León	1999	2000
Valdespino JL, Conde-González CJ, Olaíz-Fernández G, Palma O, Sepúlveda J. Prevalence in Mexico of infection and the carrier state of hepatitis B in adults. Salud Publica Mex 2007; 49(Supl 3): S404-S411. and Juárez-Figueroa LA, Uribe-Salas FJ, Conde-Glez CJ. Heterogeneous distribution of Hepatitis B serological markers in rural areas of Mexico. Salud Publica Mex 2011; 53(Supl 1): S26-S31. Mexico Hepatitis B Prevalence 1999-2000. [Unpublished].	Oaxaca	1999	2000
Valdespino JL, Conde-González CJ, Olaíz-Fernández G, Palma O, Sepúlveda J. Prevalence in Mexico of infection and the carrier state of hepatitis B in adults. Salud Publica Mex 2007; 49(Supl 3): S404-	Puebla	1999	2000

S411. and Juárez-Figueroa LA, Uribe-Salas FJ, Conde-Glez CJ. Heterogeneous distribution of Hepatitis B serological markers in rural areas of Mexico. Salud Publica Mex 2011; 53(Supl 1): S26-S31. Mexico Hepatitis B Prevalence 1999-2000. [Unpublished].			
Valdespino JL, Conde-González CJ, Olaíz-Fernández G, Palma O, Sepúlveda J. Prevalence in Mexico of infection and the carrier state of hepatitis B in adults. Salud Publica Mex 2007; 49(Supl 3): S404-S411. and Juárez-Figueroa LA, Uribe-Salas FJ, Conde-Glez CJ. Heterogeneous distribution of Hepatitis B serological markers in rural areas of Mexico. Salud Publica Mex 2011; 53(Supl 1): S26-S31. Mexico Hepatitis B Prevalence 1999-2000. [Unpublished].	Querétaro	1999	2000
Valdespino JL, Conde-González CJ, Olaíz-Fernández G, Palma O, Sepúlveda J. Prevalence in Mexico of infection and the carrier state of hepatitis B in adults. Salud Publica Mex 2007; 49(Supl 3): S404-S411. and Juárez-Figueroa LA, Uribe-Salas FJ, Conde-Glez CJ. Heterogeneous distribution of Hepatitis B serological markers in rural areas of Mexico. Salud Publica Mex 2011; 53(Supl 1): S26-S31. Mexico Hepatitis B Prevalence 1999-2000. [Unpublished].	Quintana Roo	1999	2000
Valdespino JL, Conde-González CJ, Olaíz-Fernández G, Palma O, Sepúlveda J. Prevalence in Mexico of infection and the carrier state of hepatitis B in adults. Salud Publica Mex 2007; 49(Supl 3): S404-S411. and Juárez-Figueroa LA, Uribe-Salas FJ, Conde-Glez CJ. Heterogeneous distribution of Hepatitis B serological markers in rural areas of Mexico. Salud Publica Mex 2011; 53(Supl 1): S26-S31. Mexico Hepatitis B Prevalence 1999-2000. [Unpublished].	San Luis Potosí	1999	2000
Valdespino JL, Conde-González CJ, Olaíz-Fernández G, Palma O, Sepúlveda J. Prevalence in Mexico of infection and the carrier state of hepatitis B in adults. Salud Publica Mex 2007; 49(Supl 3): S404-S411. and Juárez-Figueroa LA, Uribe-Salas FJ, Conde-Glez CJ. Heterogeneous distribution of Hepatitis B serological markers in rural areas of Mexico. Salud Publica Mex 2011; 53(Supl 1): S26-S31. Mexico Hepatitis B Prevalence 1999-2000. [Unpublished].	Sinaloa	1999	2000
Valdespino JL, Conde-González CJ, Olaíz-Fernández G, Palma O, Sepúlveda J. Prevalence in Mexico of infection and the carrier state of hepatitis B in adults. Salud Publica Mex 2007; 49(Supl 3): S404-S411. and Juárez-Figueroa LA, Uribe-Salas FJ, Conde-Glez CJ. Heterogeneous distribution of Hepatitis B serological markers in rural areas of Mexico. Salud Publica Mex 2011; 53(Supl 1): S26-S31. Mexico Hepatitis B Prevalence 1999-2000. [Unpublished].	Sonora	1999	2000
Valdespino JL, Conde-González CJ, Olaíz-Fernández G, Palma O, Sepúlveda J. Prevalence in Mexico of infection and the carrier state of hepatitis B in adults. Salud Publica Mex 2007; 49(Supl 3): S404-S411. and Juárez-Figueroa LA, Uribe-Salas FJ, Conde-Glez CJ. Heterogeneous distribution of Hepatitis B serological markers in rural areas of Mexico. Salud Publica Mex 2011; 53(Supl 1): S26-S31. Mexico Hepatitis B Prevalence 1999-2000. [Unpublished].	Tabasco	1999	2000
Valdespino JL, Conde-González CJ, Olaíz-Fernández G, Palma O, Sepúlveda J. Prevalence in Mexico of infection and the carrier state of hepatitis B in adults. Salud Publica Mex 2007; 49(Supl 3): S404-S411. and Juárez-Figueroa LA, Uribe-Salas FJ, Conde-Glez CJ. Heterogeneous distribution of Hepatitis B serological markers in rural areas of Mexico. Salud Publica Mex 2011; 53(Supl 1): S26-S31. Mexico Hepatitis B Prevalence 1999-2000. [Unpublished].	Tamaulipas	1999	2000
Valdespino JL, Conde-González CJ, Olaíz-Fernández G, Palma O, Sepúlveda J. Prevalence in Mexico of infection and the carrier state of	Tlaxcala	1999	2000

hepatitis B in adults. Salud Publica Mex 2007; 49(Supl 3): S404-S411. and Juárez-Figueroa LA, Uribe-Salas FJ, Conde-Glez CJ. Heterogeneous distribution of Hepatitis B serological markers in rural areas of Mexico. Salud Publica Mex 2011; 53(Supl 1): S26-S31. Mexico Hepatitis B Prevalence 1999-2000. [Unpublished].			
Valdespino JL, Conde-González CJ, Olaíz-Fernández G, Palma O, Sepúlveda J. Prevalence in Mexico of infection and the carrier state of hepatitis B in adults. Salud Publica Mex 2007; 49(Supl 3): S404-S411. and Juárez-Figueroa LA, Uribe-Salas FJ, Conde-Glez CJ. Heterogeneous distribution of Hepatitis B serological markers in rural areas of Mexico. Salud Publica Mex 2011; 53(Supl 1): S26-S31. Mexico Hepatitis B Prevalence 1999-2000. [Unpublished].	Veracruz de Ignacio de la Llave	1999	2000
Valdespino JL, Conde-González CJ, Olaíz-Fernández G, Palma O, Sepúlveda J. Prevalence in Mexico of infection and the carrier state of hepatitis B in adults. Salud Publica Mex 2007; 49(Supl 3): S404-S411. and Juárez-Figueroa LA, Uribe-Salas FJ, Conde-Glez CJ. Heterogeneous distribution of Hepatitis B serological markers in rural areas of Mexico. Salud Publica Mex 2011; 53(Supl 1): S26-S31. Mexico Hepatitis B Prevalence 1999-2000. [Unpublished].	Yucatán	1999	2000
Valdespino JL, Conde-González CJ, Olaíz-Fernández G, Palma O, Sepúlveda J. Prevalence in Mexico of infection and the carrier state of hepatitis B in adults. Salud Publica Mex 2007; 49(Supl 3): S404-S411. and Juárez-Figueroa LA, Uribe-Salas FJ, Conde-Glez CJ. Heterogeneous distribution of Hepatitis B serological markers in rural areas of Mexico. Salud Publica Mex 2011; 53(Supl 1): S26-S31. Mexico Hepatitis B Prevalence 1999-2000. [Unpublished].	Zacatecas	1999	2000
Valente F, Lago BV do, Castro CAV de, Almeida AJ de, Gomes SA, Soares CC. Epidemiology and molecular characterization of hepatitis B virus in Luanda, Angola. Mem Inst Oswaldo Cruz. 2010; 105(8): 9707.	Angola	2007	2009
Van Hattum J, Boland GJ, Jansen KGJJ, Kleinpenning AS, van Bommel T, van Loon AM, Abdurachman SA, Yusuf H, Rulos-van den Berg A, van den Berg J. Transmission profile of hepatitis B virus infection in the Batam region, Indonesia. Evidence for a predominantly horizontal transmission profile. Adv Exp Med Biol. 2003; 177-83.	Indonesia	1997	1997
Van Steenbergen JE, Leentvaar-Kuijpers A, Baayen D, Dukers HT, van Doornum GJ, van den Hoek JA, Coutinho RA. Evaluation of the hepatitis B antenatal screening and neonatal immunization program in Amsterdam, 1993-1998. Vaccine. 2001; 20(1-2): 7-11.	Netherlands	1993	1998
Vickers IE, Brathwaite AR, Levy M, Figueroa JP. Seroprevalence of sexually transmitted infections among accepted and deferred blood donors in Jamaica. West Indian Med J. 2006; 55(2): 89-94.	Jamaica	1998	1999
Wang CS, Wang ST, Chou P. Using the prevalence of an elevated serum alanine aminotransferase level for identifying communities with a high prevalence of hepatitis C virus infection. Arch Intern Med. 2001; 161(3): 392-4.	Taiwan (Province of China)	1999	1999
Wang L-Y, Hu C-T, Ho T-Y, Lin HH. Geographic and ethnic variations of long-term efficacy and immunogenicity of hepatitis B vaccination in Hualien, a HBV hyperendemic area. Vaccine. 2006; 24(20): 4427-32.	Taiwan (Province of China)	2002	2004
Wang Y, Tao QM, Zhao HY, Tsuda F, Nagayama R, Yamamoto K, Tanaka T, Tokita H, Okamoto H, Miyakawa Y. Hepatitis C virus RNA and antibodies among blood donors in Beijing. J Hepatol. 1994; 21(4): 634-40.	Beijing	1992	1992

Wasfi O a S, Sadek NA. Prevalence of hepatitis B surface antigen and hepatitis C virus antibodies among blood donors in Alexandria, Egypt. East Mediterr Health J. 2011; 17(3): 23842.	Egypt	2007	2008
Werner GT, Frosner GG, Sareen DK. Prevalence of hepatitis A, B and HIV markers in Punjab. J Indian Med Assoc. 1990; 88(10): 293-4.	India	1986	1988
Wilson N, Ruff TA, Rana BJ, Leydon J, Locarnini S. The effectiveness of the infant hepatitis B immunisation program in Fiji, Kiribati, Tonga and Vanuatu. Vaccine. 2000; 18(26): 3059-66.	Fiji	1998	1998
Wong SN, Ong JP, Labio MED, Cabahug OT, Daez MLO, Valdellon EV, Sollano JD Jr, Arguillas MO. Hepatitis B infection among adults in the philippines: A national seroprevalence study. World J Hepatol. 2013; 5(4): 214-9.	Philippines	2003	2003
Wu T-W, Lin HH, Wang L-Y. Chronic hepatitis B infection in adolescents who received primary infantile vaccination. Hepatology. 2013; 57(1): 3745.	Taiwan (Province of China)	2003	2008
Xia GL, Liu CB, Caoa HL, BP SL, Zhan MY, Sub CA, Nan JH, Qi XQ. Prevalence of hepatitis B and C virus infections in the general Chinese population: Results from a nationwide cross-sectional seroepidemiologic study of hepatitis A, B, C, D, and E virus infections in China, 1992. Int Hepatol Commun. 1996; 5: 62-73.	Anhui	1992	1992
Xia GL, Liu CB, Caoa HL, BP SL, Zhan MY, Sub CA, Nan JH, Qi XQ. Prevalence of hepatitis B and C virus infections in the general Chinese population: Results from a nationwide cross-sectional seroepidemiologic study of hepatitis A, B, C, D, and E virus infections in China, 1992. Int Hepatol Commun. 1996; 5: 62-73.	Beijing	1992	1992
Xia GL, Liu CB, Caoa HL, BP SL, Zhan MY, Sub CA, Nan JH, Qi XQ. Prevalence of hepatitis B and C virus infections in the general Chinese population: Results from a nationwide cross-sectional seroepidemiologic study of hepatitis A, B, C, D, and E virus infections in China, 1992. Int Hepatol Commun. 1996; 5: 62-73.	Chongqing	1992	1992
Xia GL, Liu CB, Caoa HL, BP SL, Zhan MY, Sub CA, Nan JH, Qi XQ. Prevalence of hepatitis B and C virus infections in the general Chinese population: Results from a nationwide cross-sectional seroepidemiologic study of hepatitis A, B, C, D, and E virus infections in China, 1992. Int Hepatol Commun. 1996; 5: 62-73.	Fujian	1992	1992
Xia GL, Liu CB, Caoa HL, BP SL, Zhan MY, Sub CA, Nan JH, Qi XQ. Prevalence of hepatitis B and C virus infections in the general Chinese population: Results from a nationwide cross-sectional seroepidemiologic study of hepatitis A, B, C, D, and E virus infections in China, 1992. Int Hepatol Commun. 1996; 5: 62-73.	Gansu	1992	1992
Xia GL, Liu CB, Caoa HL, BP SL, Zhan MY, Sub CA, Nan JH, Qi XQ. Prevalence of hepatitis B and C virus infections in the general Chinese population: Results from a nationwide cross-sectional seroepidemiologic study of hepatitis A, B, C, D, and E virus infections in China, 1992. Int Hepatol Commun. 1996; 5: 62-73.	Guangdong	1992	1992
Xia GL, Liu CB, Caoa HL, BP SL, Zhan MY, Sub CA, Nan JH, Qi XQ. Prevalence of hepatitis B and C virus infections in the general Chinese population: Results from a nationwide cross-sectional seroepidemiologic study of hepatitis A, B, C, D, and E virus infections in China, 1992. Int Hepatol Commun. 1996; 5: 62-73.	Guangxi	1992	1992
Xia GL, Liu CB, Caoa HL, BP SL, Zhan MY, Sub CA, Nan JH, Qi XQ. Prevalence of hepatitis B and C virus infections in the general Chinese population: Results from a nationwide cross-sectional	Guizhou	1992	1992

seroepidemiologic study of hepatitis A, B, C, D, and E virus infections in China, 1992. Int Hepatol Commun. 1996; 5: 62-73.			
Xia GL, Liu CB, Caoa HL, BP SL, Zhan MY, Sub CA, Nan JH, Qi XQ. Prevalence of hepatitis B and C virus infections in the general Chinese population: Results from a nationwide cross-sectional seroepidemiologic study of hepatitis A, B, C, D, and E virus infections in China, 1992. Int Hepatol Commun. 1996; 5: 62-73.	Hainan	1992	1992
Xia GL, Liu CB, Caoa HL, BP SL, Zhan MY, Sub CA, Nan JH, Qi XQ. Prevalence of hepatitis B and C virus infections in the general Chinese population: Results from a nationwide cross-sectional seroepidemiologic study of hepatitis A, B, C, D, and E virus infections in China, 1992. Int Hepatol Commun. 1996; 5: 62-73.	Hebei	1992	1992
Xia GL, Liu CB, Caoa HL, BP SL, Zhan MY, Sub CA, Nan JH, Qi XQ. Prevalence of hepatitis B and C virus infections in the general Chinese population: Results from a nationwide cross-sectional seroepidemiologic study of hepatitis A, B, C, D, and E virus infections in China, 1992. Int Hepatol Commun. 1996; 5: 62-73.	Heilongjiang	1992	1992
Xia GL, Liu CB, Caoa HL, BP SL, Zhan MY, Sub CA, Nan JH, Qi XQ. Prevalence of hepatitis B and C virus infections in the general Chinese population: Results from a nationwide cross-sectional seroepidemiologic study of hepatitis A, B, C, D, and E virus infections in China, 1992. Int Hepatol Commun. 1996; 5: 62-73.	Henan	1992	1992
Xia GL, Liu CB, Caoa HL, BP SL, Zhan MY, Sub CA, Nan JH, Qi XQ. Prevalence of hepatitis B and C virus infections in the general Chinese population: Results from a nationwide cross-sectional seroepidemiologic study of hepatitis A, B, C, D, and E virus infections in China, 1992. Int Hepatol Commun. 1996; 5: 62-73.	Hubei	1992	1992
Xia GL, Liu CB, Caoa HL, BP SL, Zhan MY, Sub CA, Nan JH, Qi XQ. Prevalence of hepatitis B and C virus infections in the general Chinese population: Results from a nationwide cross-sectional seroepidemiologic study of hepatitis A, B, C, D, and E virus infections in China, 1992. Int Hepatol Commun. 1996; 5: 62-73.	Hunan	1992	1992
Xia GL, Liu CB, Caoa HL, BP SL, Zhan MY, Sub CA, Nan JH, Qi XQ. Prevalence of hepatitis B and C virus infections in the general Chinese population: Results from a nationwide cross-sectional seroepidemiologic study of hepatitis A, B, C, D, and E virus infections in China, 1992. Int Hepatol Commun. 1996; 5: 62-73.	Inner Mongolia	1992	1992
Xia GL, Liu CB, Caoa HL, BP SL, Zhan MY, Sub CA, Nan JH, Qi XQ. Prevalence of hepatitis B and C virus infections in the general Chinese population: Results from a nationwide cross-sectional seroepidemiologic study of hepatitis A, B, C, D, and E virus infections in China, 1992. Int Hepatol Commun. 1996; 5: 62-73.	Jiangsu	1992	1992
Xia GL, Liu CB, Caoa HL, BP SL, Zhan MY, Sub CA, Nan JH, Qi XQ. Prevalence of hepatitis B and C virus infections in the general Chinese population: Results from a nationwide cross-sectional seroepidemiologic study of hepatitis A, B, C, D, and E virus infections in China, 1992. Int Hepatol Commun. 1996; 5: 62-73.	Jiangxi	1992	1992
Xia GL, Liu CB, Caoa HL, BP SL, Zhan MY, Sub CA, Nan JH, Qi XQ. Prevalence of hepatitis B and C virus infections in the general Chinese population: Results from a nationwide cross-sectional seroepidemiologic study of hepatitis A, B, C, D, and E virus infections in China, 1992. Int Hepatol Commun. 1996; 5: 62-73.	Jilin	1992	1992
Xia GL, Liu CB, Caoa HL, BP SL, Zhan MY, Sub CA, Nan JH, Qi XQ. Prevalence of hepatitis B and C virus infections in the general Chinese population: Results from a nationwide cross-sectional	Liaoning	1992	1992

seroepidemiologic study of hepatitis A, B, C, D, and E virus infections in China, 1992. Int Hepatol Commun. 1996; 5: 62-73.			
Xia GL, Liu CB, Caoa HL, BP SL, Zhan MY, Sub CA, Nan JH, Qi XQ. Prevalence of hepatitis B and C virus infections in the general Chinese population: Results from a nationwide cross-sectional seroepidemiologic study of hepatitis A, B, C, D, and E virus infections in China, 1992. Int Hepatol Commun. 1996; 5: 62-73.	Ningxia	1992	1992
Xia GL, Liu CB, Caoa HL, BP SL, Zhan MY, Sub CA, Nan JH, Qi XQ. Prevalence of hepatitis B and C virus infections in the general Chinese population: Results from a nationwide cross-sectional seroepidemiologic study of hepatitis A, B, C, D, and E virus infections in China, 1992. Int Hepatol Commun. 1996; 5: 62-73.	Qinghai	1992	1992
Xia GL, Liu CB, Caoa HL, BP SL, Zhan MY, Sub CA, Nan JH, Qi XQ. Prevalence of hepatitis B and C virus infections in the general Chinese population: Results from a nationwide cross-sectional seroepidemiologic study of hepatitis A, B, C, D, and E virus infections in China, 1992. Int Hepatol Commun. 1996; 5: 62-73.	Shaanxi	1992	1992
Xia GL, Liu CB, Caoa HL, BP SL, Zhan MY, Sub CA, Nan JH, Qi XQ. Prevalence of hepatitis B and C virus infections in the general Chinese population: Results from a nationwide cross-sectional seroepidemiologic study of hepatitis A, B, C, D, and E virus infections in China, 1992. Int Hepatol Commun. 1996; 5: 62-73.	Shandong	1992	1992
Xia GL, Liu CB, Caoa HL, BP SL, Zhan MY, Sub CA, Nan JH, Qi XQ. Prevalence of hepatitis B and C virus infections in the general Chinese population: Results from a nationwide cross-sectional seroepidemiologic study of hepatitis A, B, C, D, and E virus infections in China, 1992. Int Hepatol Commun. 1996; 5: 62-73.	Shanghai	1992	1992
Xia GL, Liu CB, Caoa HL, BP SL, Zhan MY, Sub CA, Nan JH, Qi XQ. Prevalence of hepatitis B and C virus infections in the general Chinese population: Results from a nationwide cross-sectional seroepidemiologic study of hepatitis A, B, C, D, and E virus infections in China, 1992. Int Hepatol Commun. 1996; 5: 62-73.	Shanxi	1992	1992
Xia GL, Liu CB, Caoa HL, BP SL, Zhan MY, Sub CA, Nan JH, Qi XQ. Prevalence of hepatitis B and C virus infections in the general Chinese population: Results from a nationwide cross-sectional seroepidemiologic study of hepatitis A, B, C, D, and E virus infections in China, 1992. Int Hepatol Commun. 1996; 5: 62-73.	Sichuan	1992	1992
Xia GL, Liu CB, Caoa HL, BP SL, Zhan MY, Sub CA, Nan JH, Qi XQ. Prevalence of hepatitis B and C virus infections in the general Chinese population: Results from a nationwide cross-sectional seroepidemiologic study of hepatitis A, B, C, D, and E virus infections in China, 1992. Int Hepatol Commun. 1996; 5: 62-73.	Tianjin	1992	1992
Xia GL, Liu CB, Caoa HL, BP SL, Zhan MY, Sub CA, Nan JH, Qi XQ. Prevalence of hepatitis B and C virus infections in the general Chinese population: Results from a nationwide cross-sectional seroepidemiologic study of hepatitis A, B, C, D, and E virus infections in China, 1992. Int Hepatol Commun. 1996; 5: 62-73.	Tibet	1992	1992
Xia GL, Liu CB, Caoa HL, BP SL, Zhan MY, Sub CA, Nan JH, Qi XQ. Prevalence of hepatitis B and C virus infections in the general Chinese population: Results from a nationwide cross-sectional seroepidemiologic study of hepatitis A, B, C, D, and E virus infections in China, 1992. Int Hepatol Commun. 1996; 5: 62-73.	Xinjiang	1992	1992
Xia GL, Liu CB, Caoa HL, BP SL, Zhan MY, Sub CA, Nan JH, Qi XQ. Prevalence of hepatitis B and C virus infections in the general Chinese population: Results from a nationwide cross-sectional	Yunnan	1992	1992

seroepidemiologic study of hepatitis A, B, C, D, and E virus infections in China, 1992. Int Hepatol Commun. 1996; 5: 62-73.			
Xia GL, Liu CB, Caoa HL, BP SL, Zhan MY, Sub CA, Nan JH, Qi XQ. Prevalence of hepatitis B and C virus infections in the general Chinese population: Results from a nationwide cross-sectional seroepidemiologic study of hepatitis A, B, C, D, and E virus infections in China, 1992. Int Hepatol Commun. 1996; 5: 62-73.	Zhejiang	1992	1992
Yamaguchi K, Inaoka T, Ohtsuka R, Akimichi T, Hongo T, Kawabe T, Nakazawa M, Futatsuka M, Takatsuki K. HTLV-I, HIV-I, and hepatitis B and C viruses in Western Province, Papua New Guinea: a serological survey. Jpn J Cancer Res. 1993; 84(7): 715-9.	Papua New Guinea	1989	1989
Yami A, Alemseged F, Hassen A. Hepatitis B and C Viruses Infections and Their Association with Human Immunodeficiency Virus: A Cross-Sectional Study among Blood Donors in Ethiopia. Ethiop J Health Sci. 2011; 21(1): 6775.	Oromia	1988	2010
Yanase Y, Ohida T, Kaneita Y, Agdamag DMD, Leaño PSA, Gill CJ. The prevalence of HIV, HBV and HCV among Filipino blood donors and overseas work visa applicants. Bull World Health Organ. 2007; 85(2): 131-7.	Philippines	2002	2004
Yang J-F, Lin C-I, Huang J-F, Dai C-Y, Lin W-Y, Ho C-K, Hsieh M-Y, Lee L-P, Ho N-J, Lin Z-Y, Chen S-C, Hsieh M-Y, Wang L-Y, Yu M-L, Chuang W-L, Chang W-Y. Viral hepatitis infections in southern Taiwan: a multicenter community-based study. Kaohsiung J Med Sci. 2010; 26(9): 461-9.	Taiwan (Province of China)	1999	2005
Yildirim B, Barut S, Bulut Y, Yenişehirli G, Ozdemir M, Cetin I, Etikan I, Akbaş A, Atiş O, Ozyurt H, Sahin S. Seroprevalence of hepatitis B and C viruses in the province of Tokat in the Black Sea region of Turkey: A population-based study. Turk J Gastroenterol. 2009; 20(1): 27-30.	Turkey	2006	2007
Zacharakis G, Kotsiou S, Papoutselis M, Vafiadis N, Tzara F, Pouliou E, Maltezos E, Koskinas J, Papoutselis K. Changes in the epidemiology of hepatitis B virus infection following the implementation of immunisation programmes in northeastern Greece. Euro Surveill. 2009; 14(32).	Greece	1992	1994
Zacharakis G, Kotsiou S, Papoutselis M, Vafiadis N, Tzara F, Pouliou E, Maltezos E, Koskinas J, Papoutselis K. Changes in the epidemiology of hepatitis B virus infection following the implementation of immunisation programmes in northeastern Greece. Euro Surveill. 2009; 14(32).	Greece	1998	2006
Zahran KM, Badary MS, Agban MN, Abdel Aziz NHR. Pattern of hepatitis virus infection among pregnant women and their newborns at the Womens Health Center of Assiut University, Upper Egypt. Int J Gynaecol Obstet. 2010; 111(2): 1714.	Egypt	2008	2009
Zaki H, Darmstadt GL, Baten A, Ahsan CR, Saha SK. Seroepidemiology of hepatitis B and delta virus infections in Bangladesh. J Trop Pediatr. 2003; 49(6): 371-4.	Bangladesh	1997	1998
Zali MR, Mohammad K, Noorbala AA, Noorimayer B, Shahraz S, Sahraz S. Rate of hepatitis B seropositivity following mass vaccination in the Islamic Republic of Iran. East Mediterr Health J. 2005; 11(1-2): 62-7.	Iran (Islamic Republic of)	1991	1991
Zali MR, Mohammad K, Noorbala AA, Noorimayer B, Shahraz S, Sahraz S. Rate of hepatitis B seropositivity following mass vaccination in the Islamic Republic of Iran. East Mediterr Health J. 2005; 11(1-2): 62-7.	Iran (Islamic Republic of)	1999	1999

Zekri A-RN, Awlia AA, El Mahalawi H, Ismail EF, Mabrouk GM. Evaluation of blood units with isolated anti HBC for the presence of HBV DNA. Dis Markers. 2002; 18(3): 107-10.	Saudi Arabia	1999	2000
Zhang ZW, Shimbo S, Qu JB, Liu ZM, Cai XC, Wang LQ, Watanabe T, Nakatsuka H, Matsuda-Inoguchi N, Higashikawa K, Ikeda M. Hepatitis B and C virus infection among adult women in Jilin Province, China: an urban-rural comparison in prevalence of infection markers. Southeast Asian J Trop Med Public Health. 2000; 31(3): 530-6.	Jilin	1999	1999
Zhuo J, Tao G, Ebrahim SH, Wang S, Luo Z, Wang H. The relationship of hepatitis B virus infection between adults and their children in Guangxi Province, China. J Hepatol. 2000; 33(4): 628-31.	Guangxi	1992	1992
Ziraba AK, Bwogi J, Namale A, Wainaina CW, Mayanja-Kizza H. Sero-prevalence and risk factors for hepatitis B virus infection among health care workers in a tertiary hospital in Uganda. BMC Infect Dis. 2010; 10: 191.	Uganda	2003	2003
Zohoun A, Hadef R, Zahid H, Benkirane M. Seroprevalence of HBV and HCV in blood donors at the Blood Transfusion Center of Mohammed V Military Teaching Hospital in Rabat Morocco. Med Trop (Mars). 2011; 71(5): 5134.	Morocco	2008	2009

b)

Citation	Location	Year start	Year end
National Center for Health Statistics (NCHS), Centers for Disease Control and Prevention (CDC). United States National Health and Nutrition Examination Survey 2005-2006. Hyattsville, United States: National Center for Health Statistics (NCHS), Centers for Disease Control and Prevention (CDC), 2007.	United States of America	2005	2006
National Center for Health Statistics (NCHS), Centers for Disease Control and Prevention (CDC). United States National Health and Nutrition Examination Survey 2005-2006. Hyattsville, United States: National Center for Health Statistics (NCHS), Centers for Disease Control and Prevention (CDC), 2007.	United States of America	2007	2008
National Center for Health Statistics (NCHS), Centers for Disease Control and Prevention (CDC). United States National Health and Nutrition Examination Survey 2005-2006. Hyattsville, United States: National Center for Health Statistics (NCHS), Centers for Disease Control and Prevention (CDC), 2007.	United States of America	2009	2010
Abbas Z, Jeswani NL, Kakepoto GN, Islam M, Mehdi K, Jafri W. Prevalence and mode of spread of hepatitis B and C in rural Sindh, Pakistan. Trop Gastroenterol. 2008; 29(4): 210-216.	Sindh	2005	2007
Abdullah FE, Memon AA, Mushtaq A, Adil SE-R, Kazmi SU. Frequency of HBsAg positivity: a laboratory data analysis. J Coll Physicians Surg Pak. 2012; 22(11): 7423.	Sindh	2009	2010
Acquaye JK, Mingle JA. Hepatitis B viral markers in Ghanaian pregnant women. West Afr J Med. 1994; 13(3): 134-7.	Ghana	1992	1992
Agbede OO, Iseniyi JO, Kolawole MO, Ojuawo A. Risk factors and seroprevalence of hepatitis B surface antigenemia in mothers and their preschool age children in Ilorin, Nigeria. Ther. 2007; 4(1): 67-72.	Kwara	2004	2006
Al Awaidy ST, Bawikar SP, Al Busaidy SS, Al Mahrouqi S, Al Baqlani S, Al Obaidani I, Alexander J, Patel MK. Progress toward elimination of hepatitis B virus transmission in Oman: impact of hepatitis B vaccination. Am J Trop Med Hyg. 2013; 89(4): 8115.	Oman	2005	2005

Alvarado-Mora MV, Gutierrez Fernandez MF, Gomes-Gouvêa MS, de Azevedo Neto RS, Carrilho FJ, Pinho JRR. Hepatitis B (HBV), Hepatitis C (HCV) and Hepatitis Delta (HDV) Viruses in the Colombian Population-How Is the Epidemiological Situation?. PLoS One. 2011; 6(4): e18888.	Colombia	2008	2009
Ang LW, Tey SH, Cutter J, James L, Goh KT. Seroprevalence of hepatitis B virus infection among children and adolescents in Singapore, 2008-2010. J Med Virol. 2013; 85(4): 583-8.	Singapore	2008	2010
Ansari-Moghaddam A, Ostovaneh MR, Sharifi Mood B, Sanei-Moghaddam E, Modabbernia A, Poustchi H. Seroprevalence of hepatitis B surface antigen and anti hepatitis C antibody in zahedan city, iran: a population-based study. Hepat Mon. 2012; 12(9): e6618.	Iran (Islamic Republic of)	2008	2009
Ashraf H, Alam NH, Rothermundt C, Brooks A, Bardhan P, Hossain L, Salam MA, Hassan MS, Beglinger C, Gyr N. Prevalence and risk factors of hepatitis B and C virus infections in an impoverished urban community in Dhaka, Bangladesh. BMC Infect Dis. 2010; 10(1): 208.	Bangladesh	2005	2006
Ashwell MJ, Cossart YE. An autopsy survey of hepatitis B in Sydney. Pathology. 1995; 27(1): 43-7.	Australia	1984	1984
Barton EN, King SD, Douglas LL. The seroprevalence of hepatitis and retroviral infection in Jamaican haemodialysis patients. West Indian Med J. 1998; 47(3): 105-7.	Jamaica	1996	1996
Bialek SR, Helgenberger L, Fischer GE, Bower WA, Konelios M, Chaine J-P, Armstrong G, Williams IT, Bell BP. Impact of routine hepatitis B immunization on the prevalence of chronic hepatitis B virus infection in the marshall islands and the federated States of micronesia. Pediatr Infect Dis J. 2010; 29(1): 1822.	Micronesia (Federated States of)	2000	2000
Bialek SR, Helgenberger L, Fischer GE, Bower WA, Konelios M, Chaine J-P, Armstrong G, Williams IT, Bell BP. Impact of routine hepatitis B immunization on the prevalence of chronic hepatitis B virus infection in the marshall islands and the federated States of micronesia. Pediatr Infect Dis J. 2010; 29(1): 1822.	Micronesia (Federated States of)	2005	2005
Bialek SR, Helgenberger L, Fischer GE, Bower WA, Konelios M, Chaine J-P, Armstrong G, Williams IT, Bell BP. Impact of routine hepatitis B immunization on the prevalence of chronic hepatitis B virus infection in the marshall islands and the federated States of micronesia. Pediatr Infect Dis J. 2010; 29(1): 1822.	Marshall Islands	2007	2007
Boisier P, Rabarijaona L, Piollet M, Roux JF, Zeller HG. Hepatitis B virus infection in general population in Madagascar: evidence for different epidemiological patterns in urban and in rural areas. Epidemiol Infect. 1996; 117(1): 133-7.	Madagascar	1993	1993
Bonura F, Sorgi M, Perna AM, Puccio G, Tramuto F, Cajozzo C, Romano N, Vitale F. Pregnant women as a sentinel population to target and implement hepatitis B virus (HBV) vaccine coverage: a three-year survey in Palermo, Sicily. Vaccine. 2005; 23(25): 3243-6.	Sicilia	2001	2003
Bowry TR, Pade J, Omari M, Chemtai A. A pilot study of hepatitis B virus seroepidemiology suggests widespread immunosuppression in the nomadic inhabitants of Turkana District of Kenya. East Afr Med J. 1985; 62(7): 501-6.	Kenya	1983	1983
Braga WSM, Castilho M da C, Borges FG, Martinho AC de S, Rodrigues IS, Azevedo EP de, Scazufca M, Menezes PR. Prevalence of hepatitis B virus infection and carriage after nineteen years of vaccination program in the Western Brazilian Amazon. Rev Soc Bras Med Trop. 2012; 45(1): 13-7.	Brazil	2007	2007

Brown P, Breguet G, Smallwood L, Ney R, Moerdowo RM, Gerety RJ. Serologic markers of hepatitis A and B in the population of Bali, Indonesia. Am J Trop Med Hyg. 1985; 34(3): 616-9.	Indonesia	1978	1981
Chamberlin J, Bryan JP, Jones DL, Reyes L, Hakre S. Seroprevalence of hepatitis B virus among school-age children in the Stann Creek District of Belize, Central America. Am J Trop Med Hyg. 1996; 55(4): 452-5.	Belize	1995	1995
Chen C-H, Yang P-M, Huang G-T, Lee H-S, Sung J-L, Sheu J-C. Estimation of seroprevalence of hepatitis B virus and hepatitis C virus in Taiwan from a large-scale survey of free hepatitis screening participants. J Formos Med Assoc. 2007; 106(2): 148-55.	Taiwan (Province of China)	1996	2005
Chen C-Y, Hsu H-Y, Liu C-C, Chang M-H, Ni Y-H. Stable seroepidemiology of hepatitis B after universal immunization in Taiwan: A 3-year study of national surveillance of primary school students. Vaccine. 2010; 28(34): 56058.	Taiwan (Province of China)	2005	2005
Chen C-Y, Hsu H-Y, Liu C-C, Chang M-H, Ni Y-H. Stable seroepidemiology of hepatitis B after universal immunization in Taiwan: A 3-year study of national surveillance of primary school students. Vaccine. 2010; 28(34): 56058.	Taiwan (Province of China)	2006	2006
Chen C-Y, Hsu H-Y, Liu C-C, Chang M-H, Ni Y-H. Stable seroepidemiology of hepatitis B after universal immunization in Taiwan: A 3-year study of national surveillance of primary school students. Vaccine. 2010; 28(34): 56058.	Taiwan (Province of China)	2007	2007
Chen HL, Chang MH, Ni YH, Hsu HY, Lee PI, Lee CY, Chen DS. Seroepidemiology of hepatitis B virus infection in children: Ten years of mass vaccination in Taiwan. JAMA. 1996; 276(11): 9068.	Taiwan (Province of China)	1989	1989
Chen HL, Chang MH, Ni YH, Hsu HY, Lee PI, Lee CY, Chen DS. Seroepidemiology of hepatitis B virus infection in children: Ten years of mass vaccination in Taiwan. JAMA. 1996; 276(11): 9068.	Taiwan (Province of China)	1994	1994
Chen S-M, Kung C-M, Yang W-J, Wang H-L. Efficacy of the nationwide hepatitis B infant vaccination program in Taiwan. J Clin Virol. 2011; 52(1): 116.	Taiwan (Province of China)	2004	2009
Chiaramonte M, Floreani A, Naccarato R. Hepatitis B virus infection in homes for the aged. J Med Virol. 1982; 9(4): 247-55.	Veneto	1980	1980
Chiaramonte M, Floreani A, Silvan C, Zampieri L, Trivello R, Renzulli G, Moschen M, Naccarato R. Hepatitis A and hepatitis B virus infection in children and adolescents in north-east Italy. J Med Virol. 1983; 12(3): 179-86.	Veneto	1979	1980
Chu F-Y, Su F-H, Cheng S-H, Lin Y-S, Li C-Y, Chien C-C, Lin Y-C, Chiang S-Y. Hepatitis B surface antigen confirmatory testing for diagnosis of hepatitis B virus infection in Taiwan. J Med Virol. 2011; 83(9): 151421.	Taiwan (Province of China)	2008	2008
Chunsuttiwat S, Biggs BA, Maynard J, Thamapalo S, Laoboripat S, Bovornsin S, Charanasri U, Pinyowiwat W, Kunasol P. Integration of hepatitis B vaccination into the expanded programme on immunization in Chonburi and Chiangmai provinces, Thailand. Vaccine. 1997; 15(6-7): 769-74.	Thailand	1993	1993
Clift A, Morgan C, Anderson D, Toole M. Alarming levels of hepatitis B virus detected among rural Tibetans. Trop Doct. 2004; 34(3): 156-7.	Tibet	1999	1999
Cunha L, Plouzeau C, Ingrand P, Gudo JPS, Ingrand I, Mondlane J, Beauchant M, Agius G. Use of replacement blood donors to study the epidemiology of major blood-borne viruses in the general population of Maputo, Mozambique. J Med Virol. 2007; 79(12): 1832-40.	Mozambique	2004	2004

D'Argenio P, Esposito D, Mele A, Ortolani G, Adamo B, Rapicetta M, Forte P, Pisani A, Soldo L, Sarrecchia B. Decline in the exposure to hepatitis A and B infections in children in Naples, Italy. Public Health. 1989; 103(5): 385-9.	Campania	1980	1980
Davaalkham D, Ojima T, Nymadawa P, Tsend N, Lkhagvasuren T, Wiersma S, Uehara R, Watanabe M, Oki I, Nakamura Y. Seroepidemiology of hepatitis B virus infection among children in Mongolia: results of a nationwide survey. Pediatr Int. 2007; 49(3): 368-74.	Mongolia	2004	2004
Dazza MC, Trebucq A, Gaudebout C, Jarretou A, Le Hesran JY, Josse R, Delaporte E, Bréchot C, Larouze B. Population-based study of serum hepatitis B virus DNA in Gabon. Trans R Soc Trop Med Hyg. 1993; 87(5): 539-40.	Mozambique	1991	1991
Denis F, Ranger-Rogez S, Alain S, Mounier M, Debrock C, Wagner A, Delpeyroux C, Tabaste JL, Aubard Y, Preux P-M. Screening of pregnant women for hepatitis B markers in a French Provincial University Hospital (Limoges) during 15 years. Eur J Epidemiol. 2004; 19(10): 973-8.	France	1984	1984
Dobrodeeva LK, Kornienko EB, Petrenya NN, Lutfalieva GT, Schegoleva LS, Demeneva LV, Duberman BL, Tkachev AV, Chiba H, Senoo H, Ito K, Mizoguchi E, Yoshida S, Tajima K. A unique seroepidemiological pattern of HBV, HCV and HTLV-I in Nenets and Komi in northwestern Russia. Asian Pac J Cancer Prev. 2005; 6(3): 342-5.	Russian Federation	2004	2004
Ferrer JF, Del Pino N, Esteban E, Sherman MP, Dube S, Dube DK, Basombrio MA, Pimentel E, Segovia A, Quirulas S, Poiesz BJ. High Rate of Infection with the Human T-Cell Leukemia Retrovirus Type II in Four Indian Populations of Argentina. Virology. 1993; 197(2): 576-84.	Argentina	1991	1991
Gacche RN, Kaid AMS. Epidemiology of viral hepatitis B and C infections in ibb city, yemen. Hepat Mon. 2012; 12(7): 4602.	Yemen	2010	2010
Grosheide PM, Wladimiroff JW, Heijtink RA, Mazel JA, Christiaens GC, Nuijten AS, Schalm SW. Proposal for routine antenatal screening at 14 weeks for hepatitis B surface antigen. Dutch Study Group on Prevention of Neonatal Hepatitis. BMJ. 1995; 311(7014): 1197-9.	Netherlands	1982	1983
Grosheide PM, Wladimiroff JW, Heijtink RA, Mazel JA, Christiaens GC, Nuijten AS, Schalm SW. Proposal for routine antenatal screening at 14 weeks for hepatitis B surface antigen. Dutch Study Group on Prevention of Neonatal Hepatitis. BMJ. 1995; 311(7014): 1197-9.	Netherlands	1983	1984
Hawkes RA, Boughton CR, Ferguson V, Vale TG. The Seroepidemiology of Hepatitis in Papua New Guinea Ii. a Long-Term Study of Hepatitis B. Am J Epidemiol. 1981; 114(4): 563-73.	Papua New Guinea	1963	1963
Hawkes RA, Boughton CR, Ferguson V, Vale TG. The Seroepidemiology of Hepatitis in Papua New Guinea Ii. a Long-Term Study of Hepatitis B. Am J Epidemiol. 1981; 114(4): 563-73.	Papua New Guinea	1964	1964
Hawkes RA, Boughton CR, Ferguson V, Vale TG. The Seroepidemiology of Hepatitis in Papua New Guinea Ii. a Long-Term Study of Hepatitis B. Am J Epidemiol. 1981; 114(4): 563-73.	Papua New Guinea	1972	1972
Hyams KC, al-Arabi MA, al-Tagani AA, Messiter JF, al-Gaali AA, George JF. Epidemiology of hepatitis B in the Gezira region of Sudan. Am J Trop Med Hyg. 1989; 40(2): 200-6.	Sudan	1986	1986
icddr,b. Impact of Hepatitis B vaccination programme in Bangladesh. Health Sci Bull. 2013; 2(1).	Bangladesh	2011	2012

Jang MK, Lee JY, Lee JH, Kim YB, Kim HY, Lee MS, Park CK, Yoo JY. Seroepidemiology of HBV infection in South Korea, 1995 through 1999. Korean J Intern Med. 2001; 16(3): 153-9.	Republic of Korea	1996	1996
Jang MK, Lee JY, Lee JH, Kim YB, Kim HY, Lee MS, Park CK, Yoo JY. Seroepidemiology of HBV infection in South Korea, 1995 through 1999. Korean J Intern Med. 2001; 16(3): 153-9.	Republic of Korea	1997	1997
Jang MK, Lee JY, Lee JH, Kim YB, Kim HY, Lee MS, Park CK, Yoo JY. Seroepidemiology of HBV infection in South Korea, 1995 through 1999. Korean J Intern Med. 2001; 16(3): 153-9.	Republic of Korea	1998	1998
Jang MK, Lee JY, Lee JH, Kim YB, Kim HY, Lee MS, Park CK, Yoo JY. Seroepidemiology of HBV infection in South Korea, 1995 through 1999. Korean J Intern Med. 2001; 16(3): 153-9.	Republic of Korea	1999	1999
Johnson DE, Snitbhan R, Scott RM, Pearlman EJ, Kennedy RS. Hepatitis B in the rural tropics. Int J Epidemiol. 1980; 9(2): 123-9.	Thailand	1975	1976
Kangin M, Turhanoglu M, Gulsun S, Cakabay B. Seroprevalence of Hepatitis B and C among Children in Endemic Areas of Turkey. Hepat Mon. 2010; 10(1): 36-41.	Turkey	2005	2008
Karatekin G, Kilinç M, Gulcan Öksuz B, Iğde M. Hepatitis B seroprevalence in children and women and the impact of the hepatitis B vaccination program in the Black Sea Region of Turkey. J Infect Dev Ctries. 2013; 7(12): 960-5.	Turkey	2007	2009
Kashiwagi S, Hayashi J, Nomura H, Kajiyama W, Ikematsu H, Noguchi A. Changing pattern of intrafamilial transmission of hepatitis B virus in Okinawa, Japan. Am J Epidemiol. 1988; 127(4): 783-7.	Japan	1980	1980
Katelaris PH, Robertson G, Bradbury R, Tippett G, Hoa DQ, Ngu MC. Seroprevalence of hepatitis viruses in children in rural Viet Nam. Trans R Soc Trop Med Hyg. 1995; 89(5): 487.	Viet Nam	1994	1994
King SD, Dodd RY, Haynes G, Wynter HH, Sullivan MT, Serjeant GR, Choo-Kang E, Michael E. Prevalence of antibodies to hepatitis C virus and other markers in Jamaica. West Indian Med J. 1995; 44(2): 55-7.	Jamaica	1991	1991
Li W-C, Lee Y-Y, Chen I-C, Sun C, Chiu F-H, Chuang C-H. Association between the hepatitis B and C viruses and metabolic diseases in patients stratified by age. Liver Int. 2013; 33(8): 1194202.	Taiwan (Province of China)	2008	2010
Liang X1, Bi S, Yang W, Wang L, Cui G, Cui F, Zhang Y, Liu J, Gong X, Chen Y, Wang F, Zheng H, Wang F, Guo J, Jia Z, Ma J, Wang H, Luo H, Li L, Jin S, Hadler SC, Wang Y. Epidemiological serosurvey of hepatitis B in China--declining HBV prevalence due to hepatitis B vaccination. Vaccine. 2009; 5(27): 6550-7.	Anhui	2006	2006
Liang X1, Bi S, Yang W, Wang L, Cui G, Cui F, Zhang Y, Liu J, Gong X, Chen Y, Wang F, Zheng H, Wang F, Guo J, Jia Z, Ma J, Wang H, Luo H, Li L, Jin S, Hadler SC, Wang Y. Epidemiological serosurvey of hepatitis B in China--declining HBV prevalence due to hepatitis B vaccination. Vaccine. 2009; 5(27): 6550-7.	Beijing	2006	2006
Liang X1, Bi S, Yang W, Wang L, Cui G, Cui F, Zhang Y, Liu J, Gong X, Chen Y, Wang F, Zheng H, Wang F, Guo J, Jia Z, Ma J, Wang H, Luo H, Li L, Jin S, Hadler SC, Wang Y. Epidemiological serosurvey of hepatitis B in China--declining HBV prevalence due to hepatitis B vaccination. Vaccine. 2009; 5(27): 6550-7.	Chongqing	2006	2006
Liang X1, Bi S, Yang W, Wang L, Cui G, Cui F, Zhang Y, Liu J, Gong X, Chen Y, Wang F, Zheng H, Wang F, Guo J, Jia Z, Ma J, Wang H, Luo H, Li L, Jin S, Hadler SC, Wang Y. Epidemiological serosurvey of hepatitis B in China--declining HBV prevalence due to hepatitis B vaccination. Vaccine. 2009; 5(27): 6550-7.	Fujian	2006	2006

Liang X1, Bi S, Yang W, Wang L, Cui G, Cui F, Zhang Y, Liu J, Gong X, Chen Y, Wang F, Zheng H, Wang F, Guo J, Jia Z, Ma J, Wang H, Luo H, Li L, Jin S, Hadler SC, Wang Y. Epidemiological serosurvey of hepatitis B in China--declining HBV prevalence due to hepatitis B vaccination. Vaccine. 2009; 5(27): 6550-7.	Gansu	2006	2006
Liang X1, Bi S, Yang W, Wang L, Cui G, Cui F, Zhang Y, Liu J, Gong X, Chen Y, Wang F, Zheng H, Wang F, Guo J, Jia Z, Ma J, Wang H, Luo H, Li L, Jin S, Hadler SC, Wang Y. Epidemiological serosurvey of hepatitis B in China--declining HBV prevalence due to hepatitis B vaccination. Vaccine. 2009; 5(27): 6550-7.	Guangdong	2006	2006
Liang X1, Bi S, Yang W, Wang L, Cui G, Cui F, Zhang Y, Liu J, Gong X, Chen Y, Wang F, Zheng H, Wang F, Guo J, Jia Z, Ma J, Wang H, Luo H, Li L, Jin S, Hadler SC, Wang Y. Epidemiological serosurvey of hepatitis B in China--declining HBV prevalence due to hepatitis B vaccination. Vaccine. 2009; 5(27): 6550-7.	Guangxi	2006	2006
Liang X1, Bi S, Yang W, Wang L, Cui G, Cui F, Zhang Y, Liu J, Gong X, Chen Y, Wang F, Zheng H, Wang F, Guo J, Jia Z, Ma J, Wang H, Luo H, Li L, Jin S, Hadler SC, Wang Y. Epidemiological serosurvey of hepatitis B in China--declining HBV prevalence due to hepatitis B vaccination. Vaccine. 2009; 5(27): 6550-7.	Guizhou	2006	2006
Liang X1, Bi S, Yang W, Wang L, Cui G, Cui F, Zhang Y, Liu J, Gong X, Chen Y, Wang F, Zheng H, Wang F, Guo J, Jia Z, Ma J, Wang H, Luo H, Li L, Jin S, Hadler SC, Wang Y. Epidemiological serosurvey of hepatitis B in China--declining HBV prevalence due to hepatitis B vaccination. Vaccine. 2009; 5(27): 6550-7.	Hainan	2006	2006
Liang X1, Bi S, Yang W, Wang L, Cui G, Cui F, Zhang Y, Liu J, Gong X, Chen Y, Wang F, Zheng H, Wang F, Guo J, Jia Z, Ma J, Wang H, Luo H, Li L, Jin S, Hadler SC, Wang Y. Epidemiological serosurvey of hepatitis B in China--declining HBV prevalence due to hepatitis B vaccination. Vaccine. 2009; 5(27): 6550-7.	Hebei	2006	2006
Liang X1, Bi S, Yang W, Wang L, Cui G, Cui F, Zhang Y, Liu J, Gong X, Chen Y, Wang F, Zheng H, Wang F, Guo J, Jia Z, Ma J, Wang H, Luo H, Li L, Jin S, Hadler SC, Wang Y. Epidemiological serosurvey of hepatitis B in China--declining HBV prevalence due to hepatitis B vaccination. Vaccine. 2009; 5(27): 6550-7.	Heilongjiang	2006	2006
Liang X1, Bi S, Yang W, Wang L, Cui G, Cui F, Zhang Y, Liu J, Gong X, Chen Y, Wang F, Zheng H, Wang F, Guo J, Jia Z, Ma J, Wang H, Luo H, Li L, Jin S, Hadler SC, Wang Y. Epidemiological serosurvey of hepatitis B in China--declining HBV prevalence due to hepatitis B vaccination. Vaccine. 2009; 5(27): 6550-7.	Henan	2006	2006
Liang X1, Bi S, Yang W, Wang L, Cui G, Cui F, Zhang Y, Liu J, Gong X, Chen Y, Wang F, Zheng H, Wang F, Guo J, Jia Z, Ma J, Wang H, Luo H, Li L, Jin S, Hadler SC, Wang Y. Epidemiological serosurvey of hepatitis B in China--declining HBV prevalence due to hepatitis B vaccination. Vaccine. 2009; 5(27): 6550-7.	Hubei	2006	2006
Liang X1, Bi S, Yang W, Wang L, Cui G, Cui F, Zhang Y, Liu J, Gong X, Chen Y, Wang F, Zheng H, Wang F, Guo J, Jia Z, Ma J, Wang H, Luo H, Li L, Jin S, Hadler SC, Wang Y. Epidemiological serosurvey of hepatitis B in China--declining HBV prevalence due to hepatitis B vaccination. Vaccine. 2009; 5(27): 6550-7.	Hunan	2006	2006
Liang X1, Bi S, Yang W, Wang L, Cui G, Cui F, Zhang Y, Liu J, Gong X, Chen Y, Wang F, Zheng H, Wang F, Guo J, Jia Z, Ma J, Wang H, Luo H, Li L, Jin S, Hadler SC, Wang Y. Epidemiological serosurvey of hepatitis B in China--declining HBV prevalence due to hepatitis B vaccination. Vaccine. 2009; 5(27): 6550-7.	Inner Mongolia	2006	2006

Liang X1, Bi S, Yang W, Wang L, Cui G, Cui F, Zhang Y, Liu J, Gong X, Chen Y, Wang F, Zheng H, Wang F, Guo J, Jia Z, Ma J, Wang H, Luo H, Li L, Jin S, Hadler SC, Wang Y. Epidemiological serosurvey of hepatitis B in China--declining HBV prevalence due to hepatitis B vaccination. Vaccine. 2009; 5(27): 6550-7.	Jiangsu	2006	2006
Liang X1, Bi S, Yang W, Wang L, Cui G, Cui F, Zhang Y, Liu J, Gong X, Chen Y, Wang F, Zheng H, Wang F, Guo J, Jia Z, Ma J, Wang H, Luo H, Li L, Jin S, Hadler SC, Wang Y. Epidemiological serosurvey of hepatitis B in China--declining HBV prevalence due to hepatitis B vaccination. Vaccine. 2009; 5(27): 6550-7.	Jiangxi	2006	2006
Liang X1, Bi S, Yang W, Wang L, Cui G, Cui F, Zhang Y, Liu J, Gong X, Chen Y, Wang F, Zheng H, Wang F, Guo J, Jia Z, Ma J, Wang H, Luo H, Li L, Jin S, Hadler SC, Wang Y. Epidemiological serosurvey of hepatitis B in China--declining HBV prevalence due to hepatitis B vaccination. Vaccine. 2009; 5(27): 6550-7.	Jilin	2006	2006
Liang X1, Bi S, Yang W, Wang L, Cui G, Cui F, Zhang Y, Liu J, Gong X, Chen Y, Wang F, Zheng H, Wang F, Guo J, Jia Z, Ma J, Wang H, Luo H, Li L, Jin S, Hadler SC, Wang Y. Epidemiological serosurvey of hepatitis B in China--declining HBV prevalence due to hepatitis B vaccination. Vaccine. 2009; 5(27): 6550-7.	Liaoning	2006	2006
Liang X1, Bi S, Yang W, Wang L, Cui G, Cui F, Zhang Y, Liu J, Gong X, Chen Y, Wang F, Zheng H, Wang F, Guo J, Jia Z, Ma J, Wang H, Luo H, Li L, Jin S, Hadler SC, Wang Y. Epidemiological serosurvey of hepatitis B in China--declining HBV prevalence due to hepatitis B vaccination. Vaccine. 2009; 5(27): 6550-7.	Ningxia	2006	2006
Liang X1, Bi S, Yang W, Wang L, Cui G, Cui F, Zhang Y, Liu J, Gong X, Chen Y, Wang F, Zheng H, Wang F, Guo J, Jia Z, Ma J, Wang H, Luo H, Li L, Jin S, Hadler SC, Wang Y. Epidemiological serosurvey of hepatitis B in China--declining HBV prevalence due to hepatitis B vaccination. Vaccine. 2009; 5(27): 6550-7.	Qinghai	2006	2006
Liang X1, Bi S, Yang W, Wang L, Cui G, Cui F, Zhang Y, Liu J, Gong X, Chen Y, Wang F, Zheng H, Wang F, Guo J, Jia Z, Ma J, Wang H, Luo H, Li L, Jin S, Hadler SC, Wang Y. Epidemiological serosurvey of hepatitis B in China--declining HBV prevalence due to hepatitis B vaccination. Vaccine. 2009; 5(27): 6550-7.	Shaanxi	2006	2006
Liang X1, Bi S, Yang W, Wang L, Cui G, Cui F, Zhang Y, Liu J, Gong X, Chen Y, Wang F, Zheng H, Wang F, Guo J, Jia Z, Ma J, Wang H, Luo H, Li L, Jin S, Hadler SC, Wang Y. Epidemiological serosurvey of hepatitis B in China--declining HBV prevalence due to hepatitis B vaccination. Vaccine. 2009; 5(27): 6550-7.	Shandong	2006	2006
Liang X1, Bi S, Yang W, Wang L, Cui G, Cui F, Zhang Y, Liu J, Gong X, Chen Y, Wang F, Zheng H, Wang F, Guo J, Jia Z, Ma J, Wang H, Luo H, Li L, Jin S, Hadler SC, Wang Y. Epidemiological serosurvey of hepatitis B in China--declining HBV prevalence due to hepatitis B vaccination. Vaccine. 2009; 5(27): 6550-7.	Shanghai	2006	2006
Liang X1, Bi S, Yang W, Wang L, Cui G, Cui F, Zhang Y, Liu J, Gong X, Chen Y, Wang F, Zheng H, Wang F, Guo J, Jia Z, Ma J, Wang H, Luo H, Li L, Jin S, Hadler SC, Wang Y. Epidemiological serosurvey of hepatitis B in China--declining HBV prevalence due to hepatitis B vaccination. Vaccine. 2009; 5(27): 6550-7.	Shanxi	2006	2006
Liang X1, Bi S, Yang W, Wang L, Cui G, Cui F, Zhang Y, Liu J, Gong X, Chen Y, Wang F, Zheng H, Wang F, Guo J, Jia Z, Ma J, Wang H, Luo H, Li L, Jin S, Hadler SC, Wang Y. Epidemiological serosurvey of hepatitis B in China--declining HBV prevalence due to hepatitis B vaccination. Vaccine. 2009; 5(27): 6550-7.	Sichuan	2006	2006

Liang X1, Bi S, Yang W, Wang L, Cui G, Cui F, Zhang Y, Liu J, Gong X, Chen Y, Wang F, Zheng H, Wang F, Guo J, Jia Z, Ma J, Wang H, Luo H, Li L, Jin S, Hadler SC, Wang Y. Epidemiological serosurvey of hepatitis B in China--declining HBV prevalence due to hepatitis B vaccination. Vaccine. 2009; 5(27): 6550-7.	Tianjin	2006	2006
Liang X1, Bi S, Yang W, Wang L, Cui G, Cui F, Zhang Y, Liu J, Gong X, Chen Y, Wang F, Zheng H, Wang F, Guo J, Jia Z, Ma J, Wang H, Luo H, Li L, Jin S, Hadler SC, Wang Y. Epidemiological serosurvey of hepatitis B in China--declining HBV prevalence due to hepatitis B vaccination. Vaccine. 2009; 5(27): 6550-7.	Tibet	2006	2006
Liang X1, Bi S, Yang W, Wang L, Cui G, Cui F, Zhang Y, Liu J, Gong X, Chen Y, Wang F, Zheng H, Wang F, Guo J, Jia Z, Ma J, Wang H, Luo H, Li L, Jin S, Hadler SC, Wang Y. Epidemiological serosurvey of hepatitis B in China--declining HBV prevalence due to hepatitis B vaccination. Vaccine. 2009; 5(27): 6550-7.	Xinjiang	2006	2006
Liang X1, Bi S, Yang W, Wang L, Cui G, Cui F, Zhang Y, Liu J, Gong X, Chen Y, Wang F, Zheng H, Wang F, Guo J, Jia Z, Ma J, Wang H, Luo H, Li L, Jin S, Hadler SC, Wang Y. Epidemiological serosurvey of hepatitis B in China--declining HBV prevalence due to hepatitis B vaccination. Vaccine. 2009; 5(27): 6550-7.	Yunnan	2006	2006
Liang X1, Bi S, Yang W, Wang L, Cui G, Cui F, Zhang Y, Liu J, Gong X, Chen Y, Wang F, Zheng H, Wang F, Guo J, Jia Z, Ma J, Wang H, Luo H, Li L, Jin S, Hadler SC, Wang Y. Epidemiological serosurvey of hepatitis B in China--declining HBV prevalence due to hepatitis B vaccination. Vaccine. 2009; 5(27): 6550-7.	Zhejiang	2006	2006
Lin C-C, Yang C-Y, Shih C-T, Chen B-H, Huang Y-L. Waning immunity and booster responses in nursing and medical technology students who had received plasma-derived or recombinant hepatitis B vaccine during infancy. Am J Infect Control. 2011; 39(5): 40814.	Taiwan (Province of China)	2007	2008
Lingao AL, Domingo EO, West S, Reyes CM, Gasmen S, Viterbo G, Tiu E, Lansang MA. Seroepidemiology of hepatitis B virus in the Philippines. Am J Epidemiol. 1986; 123(3): 473-80.	Batangas	1979	1982
Lingao AL, Domingo EO, West S, Reyes CM, Gasmen S, Viterbo G, Tiu E, Lansang MA. Seroepidemiology of hepatitis B virus in the Philippines. Am J Epidemiol. 1986; 123(3): 473-80.	Laguna	1979	1982
Lingao AL, Domingo EO, West S, Reyes CM, Gasmen S, Viterbo G, Tiu E, Lansang MA. Seroepidemiology of hepatitis B virus in the Philippines. Am J Epidemiol. 1986; 123(3): 473-80.	Capiz	1979	1982
Lingao AL, Domingo EO, West S, Reyes CM, Gasmen S, Viterbo G, Tiu E, Lansang MA. Seroepidemiology of hepatitis B virus in the Philippines. Am J Epidemiol. 1986; 123(3): 473-80.	Leyte	1979	1982
Lu W-P, Lin G-X, Shi S, Dong J-H. Simultaneously high prevalences of hepatitis B and C virus infections in a population in Putian County, China. J Clin Microbiol. 2012; 50(6): 2142-4.	Fujian	2009	2010
Lucas RE, Faoagali JL. The serological status of Solomon Island blood donors. Southeast Asian J Trop Med Public Health. 1999; 30(3): 542-5.	Solomon Islands	1994	1995
Madzime S, Adem M, Mahomed K, Woelk GB, Mudzamiri S, Williams MA. Hepatitis B virus infection among pregnant women delivering at Harare Maternity Hospital, Harare Zimbabwe, 1996 to 1997. Cent Afr J Med. 1999; 45(8).	Zimbabwe	1996	1997
Maher CP, Harris MS, Milne A, Johnston A, Stewart A, Waldon JA. Seroepidemiology of hepatitis B infection in children in Vanuatu. Implications for vaccination strategy. Med J Aust. 1991; 154(4): 249-53.	Vanuatu	1989	1989

Makuwa M, Souqui□re S, Telfer P, Apetrei C, Vray M, Bedjabaga I, Mouinga-Ondeme A, Onanga R, Marx PA, Kazanji M, Roques P, Simon F. Identification of hepatitis B virus subgenotype A3 in rural Gabon. J Med Virol. 2006; 78(9): 1175-84.	Gabon	2001	2001
Miller WC, Shao JF, Weaver DJ, Shimokura GH, Paul DA, Lallinger GJ. Seroprevalence of viral hepatitis in Tanzanian adults. Trop Med Int Health. 1998; 3(9): 757-63.	United Republic of Tanzania	1992	1992
Moore DJ, Bucens MR, Holman CD, Ott AK, Wells JI. Prenatal screening for markers of hepatitis B in aboriginal mothers resident in non-metropolitan Western Australia. Med J Aust. 1987; 147(11-12): 557-8.	Australia	1983	1984
Murhekar MV, Murhekar KM, Sehgal SC. Age-specific prevalence of hepatitis B infection among the Karen in the Andaman and Nicobar Islands, India. Trop Doct. 2004; 34(2): 117-8.	West Bengal, Rural	2002	2002
Murhekar MV, Murhekar KM, Sehgal SC. Seroepidemiology of hepatitis B infection among tribal school children in Andaman and Nicobar Islands, India. Ann Trop Paediatr. 2004; 24(1): 85-8.	West Bengal, Rural	2002	2002
Muro FJ, Fiorillo SP, Sakasaka P, Odhiambo C, Reddy EA, Cunningham CK, Buchanan AM. Seroprevalence of Hepatitis B and C Viruses Among Children in Kilimanjaro Region, Tanzania. J Pediatr Infect Dis Soc. 2013.	United Republic of Tanzania	2006	2008
Nelson KE, Suriyanon V, Taylor E, Wongchak T, Kingkeow C, Srirak N, Lertsrimongkol C, Cheewawat W, Celentano D. The incidence of HIV-1 infections in village populations of northern Thailand. AIDS. 1994; 8(7): 951-5.	Thailand	1990	1990
Ng KP, Saw TL, Baki A, Rozainah K, Pang KW, Ramanathan M. Impact of the Expanded Program of Immunization against hepatitis B infection in school children in Malaysia. Med Microbiol Immunol. 2005; 194(3): 163-8.	Malaysia	1999	1999
Ng KP, Saw TL, Baki A, Rozainah K, Pang KW, Ramanathan M. Impact of the Expanded Program of Immunization against hepatitis B infection in school children in Malaysia. Med Microbiol Immunol. 2005; 194(3): 163-8.	Malaysia	2000	2000
Ng KP, Saw TL, Baki A, Rozainah K, Pang KW, Ramanathan M. Impact of the Expanded Program of Immunization against hepatitis B infection in school children in Malaysia. Med Microbiol Immunol. 2005; 194(3): 163-8.	Malaysia	2001	2001
Ng KP, Saw TL, Baki A, Rozainah K, Pang KW, Ramanathan M. Impact of the Expanded Program of Immunization against hepatitis B infection in school children in Malaysia. Med Microbiol Immunol. 2005; 194(3): 163-8.	Malaysia	2002	2002
Ng KP, Saw TL, Baki A, Rozainah K, Pang KW, Ramanathan M. Impact of the Expanded Program of Immunization against hepatitis B infection in school children in Malaysia. Med Microbiol Immunol. 2005; 194(3): 163-8.	Malaysia	2003	2003
Ni Y-H, Chang M-H, Jan C-F, Hsu H-Y, Chen H-L, Wu J-F, Chen D-S. Continuing Decrease in Hepatitis B Virus Infection 30 Years AfterInitiation of Infant Vaccination Program in Taiwan. Clin Gastroenterol Hepatol. 2016; 14(9): 132430.	Taiwan (Province of China)	2014	2014
Ni Y-H, Chang M-H, Wu J-F, Hsu H-Y, Chen H-L, Chen D-S. Minimization of hepatitis B infection by a 25-year universal vaccination program. J Hepatol. 2012; 57(4): 7305.	Taiwan (Province of China)	2009	2009
Noguchi A, Hayashi J, Nakashima K, Ikematsu H, Hirata M, Kashiwagi S. Decrease of hepatitis A and B virus infections in the population of Okinawa, Japan. J Infect. 1991; 23(3): 255–62.	Japan	1980	1980

Nur YA, Groen J, Elmi AM, Ott A, Osterhaus AD. Prevalence of serum antibodies against bloodborne and sexually transmitted agents in selected groups in Somalia. Epidemiol Infect. 2000; 124(1): 137-41.	Somalia	1995	1995
Ochi S, Onji M, Shiraishi K, Ohtu K, Akao T, Yano Y, Takei N, Matsui H, Ohta Y, Umeda M. Prevalence of hepatitis C virus antibody in an area endemic for hepatitis B virus and human T cell leukaemia virus. J Gastroenterol Hepatol. 1991; 6(6): 599-602.	Ehime	1990	1990
Ochola E, Ocama P, Orach CG, Nankinga ZK, Kalyango JN, McFarland W, Karamagi C. High burden of hepatitis B infection in Northern Uganda: results of a population-based survey. BMC Public Health. 2013; 13: 727.	Uganda	2010	2010
Ohba K, Mizokami M, Kato T, Ueda R, Gurtsenvitch V, Senyuta N, Syrtsev A, Zoya K, Yamashita M, Hayami M. Seroprevalence of hepatitis B virus, hepatitis C virus and GB virus-C infections in Siberia. Epidemiol Infect. 1999; 122(1): 139-43.	Russian Federation	1994	1995
Paquet C, Babes VT, Drucker J, Sénémaud B, Dobrescu A. Viral hepatitis in Bucharest. Bull World Health Organ. 1993; 71(6): 781-6.	Romania	1990	1990
Qamer S, Shahab T, Alam S, Malik A, Afzal K. Age-specific prevalence of hepatitis B surface antigen in pediatric population of Aligarh, North India. Indian J Pediatr. 2004; 71(11): 965-7.	India	2002	2003
Quoilin S, Hutse V, Vandenberghe H, Claeys F, Verhaegen E, De Cock L, Van Loock F, Top G, Van Damme P, Vranckx R, Van Oyen H. A population-based prevalence study of hepatitis A, B and C virus using oral fluid in Flanders, Belgium. Eur J Epidemiol. 2007; 22(3): 195-202.	Belgium	2003	2003
Rapicetta M, Stroffolini T, Ngatchu T, Chionne P, Ciccaglione AR, Lantum D, Chiaramonte M. Age- and sex-related study of HBV-DNA in HBsAg asymptomatic children from an endemic area (Cameroon). Ann Trop Paediatr. 1991; 11(4): 325-9.	Cameroon	1989	1989
Ratanasuwan W, Sonji A, Tiengrim S, Techasathit W, Suwanagool S. Serological survey of viral hepatitis A, B, and C at Thai Central Region and Bangkok: a population base study. Southeast Asian J Trop Med Public Health. 2004; 35(2): 416-20.	Thailand	2000	2002
Reda AA, Arafa MA, Youssry AA, Wandan EH, Ati MA de, Daebees H. Epidemiologic evaluation of the immunity against hepatitis B in Alexandria, Egypt. Eur J Epidemiol. 2003; 18(10): 1007-11.	Egypt	2001	2001
Reshetnikov OV, Khryanin AA, Teinina TR, Krivenchuk NA, Zimina IY. Hepatitis B and C seroprevalence in Novosibirsk, western Siberia. Sex Transm Infect. 2001; 77(6): 463.	Russian Federation	1995	1999
Richard-Lenoble D, Traore O, Kombila M, Roingeard P, Dubois F, Goudeau A. Hepatitis B, C, D, and E markers in rural equatorial African villages (Gabon). Am J Trop Med Hyg. 1995; 53(4): 338-41.	Gabon	1991	1992
Robinson T, Bullen C, Humphries W, Hornell J, Moyes C. The New Zealand Hepatitis B Screening Programme: screening coverage and prevalence of chronic hepatitis B infection. N Z Med J. 2005; 118(1211): U1345.	New Zealand	1999	2002
Salleras L, Domínguez A, Bruguera M, Plans P, Costa J, Cardeñosa N, Batalla J, Plasència A. Declining prevalence of hepatitis B virus infection in Catalonia (Spain) 12 years after the introduction of universal vaccination. Vaccine. 2007; 25(52): 8726-31.	Spain	2002	2002
Sanaei-Zadeh H, Amoei M, Taghaddosinejad F. Seroprevalence of HIV, HBV and HCV in forensic autopsies, of presumed low risk, in Tehran, the capital of Iran. J Clin Forensic Med. 2002; 9(4): 179-81.	Iran (Islamic Republic of)	2000	2001

Sanders RC, Lewis D, Dyke T, Alpers MP. Markers of hepatitis B infection in Tari District, Southern Highlands Province, Papua New Guinea. P N G Med J. 1992; 35(3): 197-201.	Papua New Guinea	1990	1990
Sayed HA, El Ayyat A, El Dusoki H, Zoheiry M, Mohamed S, Hassan M, El Assaly N, Awad A, El Ansary M, Saad A, El Karim AA. A cross sectional study of hepatitis B, C, some trace elements, heavy metals, aflatoxin B1 and schistosomiasis in a rural population, Egypt. J Egypt Public Health Assoc. 2005; 80(3-4): 355-88.	Egypt	2003	2003
Scaraveli NG, Passos AM, Voigt AR, Livramento A do, Tonial G, Treitinger A, Spada C. Seroprevalence of hepatitis B and hepatitis C markers in adolescents in Southern Brazil. Cad Saude Publica. 2011; 27(4): 753-8.	Brazil	2008	2008
Seiji K, Inoue O, Liu SJ, Xu XP, Jin C, Cai SX, Nakatsuka H, Watanabe T, Uchida Y, Ikeda M. Prevalence of hepatitis B virus infection markers among factory workers in Beijing, China. Asia Pac J Public Health. 1991; 5(4): 345-9.	Beijing	1988	1989
Soeung SC, Rani M, Huong V, Sarath S, Kimly C, Kohei T. Results from nationwide hepatitis B serosurvey in Cambodia using simple and rapid laboratory test: implications for National Immunization Program. Am J Trop Med Hyg. 2009; 81(2): 252-7.	Cambodia	2006	2006
Su F-H, Cheng S-H, Li C-Y, Chen J-D, Hsiao C-Y, Chien C-C, Yang Y-C, Hung H-H, Chu F-Y. Hepatitis B seroprevalence and anamnestic response amongst Taiwanese young adults with full vaccination in infancy, 20 years subsequent to national hepatitis B vaccination. Vaccine. 2007; 25(47): 8085-90.	Taiwan (Province of China)	2006	2006
Subida RD, Zhang ZW, Agetano MC, Nakatsuka H, Watanabe T, Shimbo S, Higashikawa K, Ikeda M. Hepatitis B and C virus infection prevalence among women in Manila, the Philippines. Southeast Asian J Trop Med Public Health. 1997; 28(4): 683-8.	National Capital Region	1997	1997
Tao QM, Wang Y, Wang H, Chen WR, Sun Y, Meng Q, Watanabe J, Nishioka K. Seroepidemiology of HCV and HBV infection in northern China. Gastroenterol Jpn. 1991; 156-8.	Beijing	1989	1989
Tonial GC, Passos AM, Livramento A do, Scaraveli NG, Batschauer AP de B, Bueno EC, Largura A, Spada C, Treitinger A. Hepatitis B marker seroprevalence and vaccination coverage in adolescents in the City of Itajaí, State of Santa Catarina, Southern Brazil, in 2008. Rev Soc Bras Med Trop. 2011; 44(4): 416-9.	Brazil	2008	2008
Tsen YJ, Chang MH, Hsu HY, Lee CY, Sung JL, Chen DS. Seroprevalence of hepatitis B virus infection in children in Taipei, 1989: five years after a mass hepatitis B vaccination program. J Med Virol. 1991; 34(2): 96-9.	Taiwan (Province of China)	1984	1984
Tsen YJ, Chang MH, Hsu HY, Lee CY, Sung JL, Chen DS. Seroprevalence of hepatitis B virus infection in children in Taipei, 1989: five years after a mass hepatitis B vaccination program. J Med Virol. 1991; 34(2): 96-9.	Taiwan (Province of China)	1989	1989
Tuinakelo LR, Tayler-Smith K, Khogali M, Marks GB. Prevalence of anaemia, syphilis and hepatitis B in pregnant women in Nausori, Fiji. Public Health Action. 2013; 3(1): 725.	Fiji	2011	2011
Wilson N, Ruff TA, Rana BJ, Leydon J, Locarnini S. The effectiveness of the infant hepatitis B immunisation program in Fiji, Kiribati, Tonga and Vanuatu. Vaccine. 2000; 18(26): 3059-66.	Fiji	1998	1998
Wolff AP, Ruys AH, Dolmans WM, Van Loon AM, Pangalila PF. Hepatitis B virus infection in patients with chronic liver disease and healthy controls in north-Sulawesi, Indonesia. Trop Geogr Med. 1990; 42(3): 221-5.	Indonesia	1988	1988

Wu T-W, Lin HH, Wang L-Y. Chronic hepatitis B infection in adolescents who received primary infantile vaccination. Hepatology. 2013; 57(1): 3745.	Taiwan (Province of China)	2003	2008
Xia GL, Liu CB, Caoa HL, BP SL, Zhan MY, Sub CA, Nan JH, Qi XQ. Prevalence of hepatitis B and C virus infections in the general Chinese population: Results from a nationwide cross-sectional seroepidemiologic study of hepatitis A, B, C, D, and E virus infections in China, 1992. Int Hepatol Commun. 1996; 5: 62-73.	Zhejiang	1992	1992
Zacharakis G, Kotsiou S, Papoutselis M, Vafiadis N, Tzara F, Pouliou E, Maltezos E, Koskinas J, Papoutselis K. Changes in the epidemiology of hepatitis B virus infection following the implementation of immunisation programmes in northeastern Greece. Euro Surveill. 2009; 14(32).	Greece	1998	2006

Data sources listed in Supplemental Table 1 can be viewed as extracted ("Unadjusted") and as adjusted per section 3.5 ("Adjusted"), with excluded sources marked in red, on the GBD Epi Visualization for the HBsAg Seroprevalence – counterfactual model

Supplemental Table 2. Percentage change in HBsAg prevalence (%) and prevalence counts in all ages and under 5 years between 1990 and 2019 and 2015 and 2019, by location

	HBsAg prevalence (%), all ages		HBsAg prevalence counts, all ages		HBsAg prevalence (%), under 5		HBsAg prevalence counts, under 5	
**Location Name**	**1990 to 2019**	**2015 to 2019**	**1990 to 2019**	**2015 to 2019**	**1990 to 2019**	**2015 to 2019**	**1990 to 2019**	**2015 to 2019**
Global	-31.3 (-33.9 to -29.0)	-6.8 (-8.3 to -5.5)	-0.7 (-4.4 to 2.7)	-2.7 (-4.3 to -1.3)	-76.8 (-77.5 to -76.2)	-15.2 (-16.7 to -13.6)	-75.7 (-76.4 to -75.0)	-15.1 (-16.6 to -13.5)
African Region	-36.1 (-37.9 to -34.4)	-9.2 (-10.3 to -8.0)	38.0 (34.0 to 41.6)	0.4 (-0.8 to 1.7)	-65.3 (-66.6 to -64.1)	-9.4 (-11.6 to -7.1)	-37.3 (-39.7 to -35.1)	-5.4 (-7.7 to -3.0)
Eastern Mediterranean Region	-37.4 (-39.5 to -35.2)	-7.3 (-8.9 to -5.5)	20.1 (16.1 to 24.4)	0.0 (-1.8 to 1.9)	-74.3 (-75.1 to -73.2)	-19.3 (-22.2 to -16.5)	-65.6 (-66.7 to -64.2)	-19.6 (-22.5 to -16.8)
European Region	-28.1 (-31.1 to -25.0)	-6.9 (-8.6 to -4.9)	-22.2 (-25.4 to -18.9)	-5.9 (-7.7 to -3.9)	-90.7 (-91.4 to -90.2)	-34.1 (-36.9 to -31.1)	-92.3 (-92.9 to -91.9)	-36.1 (-38.8 to -33.2)
Region of the Americas	-28.8 (-32.8 to -23.8)	-4.7 (-6.9 to -2.5)	0.5 (-5.2 to 7.5)	-1.6 (-3.8 to 0.7)	-89.6 (-90.0 to -89.1)	-6.6 (-9.5 to -3.2)	-89.9 (-90.4 to -89.5)	-9.3 (-12.2 to -6.1)
South-East Asia Region	-24.2 (-27.8 to -21.1)	-4.6 (-7.0 to -2.7)	16.9 (11.4 to 21.7)	-0.5 (-2.9 to 1.5)	-80.0 (-80.7 to -79.2)	-22.9 (-26.1 to -20.1)	-81.6 (-82.3 to -80.9)	-26.7 (-29.8 to -24.1)
Western Pacific Region	-34.3 (-38.2 to -31.0)	-7.6 (-10.1 to -5.3)	-18.6 (-23.4 to -14.5)	-5.5 (-8.0 to -3.1)	-93.6 (-94.0 to -93.3)	-45.0 (-47.0 to -42.9)	-95.1 (-95.3 to -94.8)	-41.7 (-43.8 to -39.4)
Low SDI	-30.6 (-32.5 to -28.7)	-8.6 (-10.0 to -7.4)	48.3 (44.3 to 52.3)	0.7 (-0.7 to 2.2)	-61.9 (-63.4 to -60.4)	-10.1 (-12.6 to -7.7)	-32.5 (-35.3 to -30.0)	-6.5 (-9.0 to -4.0)
Low-middle SDI	-29.1 (-31.8 to -26.2)	-5.9 (-7.7 to -4.4)	10.6 (6.5 to 15.2)	-1.2 (-3.1 to 0.4)	-75.3 (-76.2 to -74.4)	-12.8 (-16.3 to -9.4)	-74.7 (-75.6 to -73.8)	-14.6 (-18.0 to -11.2)
Middle SDI	-39.2 (-42.3 to -36.5)	-8.0 (-9.9 to -5.9)	-15.2 (-19.4 to -11.4)	-4.6 (-6.6 to -2.4)	-89.8 (-90.2 to -89.4)	-35.0 (-36.4 to -33.6)	-90.8 (-91.1 to -90.4)	-35.4 (-36.9 to -34.1)
High-middle SDI	-28.7 (-32.6 to -25.2)	-6.5 (-9.5 to -3.8)	-11.4 (-16.2 to -7.0)	-4.6 (-7.6 to -1.8)	-93.7 (-94.0 to -93.3)	-13.3 (-16.4 to -10.7)	-95.0 (-95.2 to -94.7)	-13.3 (-16.4 to -10.6)
High SDI	-24.3 (-26.7 to -21.7)	-4.0 (-5.6 to -2.6)	-6.6 (-9.7 to -3.5)	-1.8 (-3.4 to -0.3)	-87.7 (-88.3 to -87.2)	-63.0 (-64.4 to -61.4)	-88.8 (-89.4 to -88.3)	-63.8 (-65.1 to -62.2)
Central Europe, eastern Europe, and central Asia	-25.4 (-29.4 to -21.3)	-7.4 (-10.2 to -4.2)	-25.6 (-29.6 to -21.4)	-6.9 (-9.6 to -3.6)	-88.6 (-89.5 to -87.9)	-30.2 (-34.3 to -25.9)	-91.2 (-91.9 to -90.7)	-32.7 (-36.7 to -28.5)
Central Asia	-33.2 (-38.7 to -25.8)	-9.2 (-13.6 to -4.1)	-9.9 (-17.3 to 0.2)	-4.2 (-8.8 to 1.1)	-90.1 (-91.2 to -88.9)	-14.4 (-21.4 to -6.2)	-90.0 (-91.1 to -88.8)	-14.9 (-21.8 to -6.8)
Armenia	-30.5 (-38.0 to -20.6)	-2.8 (-11.8 to 7.5)	-38.5 (-45.1 to -29.8)	-3.9 (-12.8 to 6.2)	-91.7 (-92.9 to -90.3)	-10.3 (-22.8 to 5.7)	-95.5 (-96.2 to -94.8)	-17.6 (-29.1 to -2.9)
Azerbaijan	-17.5 (-25.6 to -7.7)	-3.0 (-11.5 to 8.5)	15.7 (4.3 to 29.4)	0.9 (-8.0 to 12.8)	-78.7 (-81.1 to -76.0)	-3.6 (-15.4 to 8.5)	-82.2 (-84.2 to -79.9)	-11.1 (-22.0 to -0.0)
Georgia	-23.5 (-25.1 to -21.7)	-6.0 (-6.9 to -5.1)	-49.1 (-50.2 to -47.9)	-9.2 (-10.0 to -8.3)	-82.9 (-85.0 to -80.8)	-6.4 (-15.7 to 4.0)	-90.9 (-92.1 to -89.8)	-13.9 (-22.5 to -4.3)
Kazakhstan	-40.0 (-46.6 to -32.0)	-7.4 (-17.9 to 2.8)	-32.6 (-40.0 to -23.6)	-2.2 (-13.3 to 8.6)	-92.5 (-93.7 to -91.0)	-18.1 (-33.2 to 0.7)	-92.5 (-93.8 to -91.1)	-17.7 (-33.0 to 1.0)
Kyrgyzstan	-37.6 (-44.8 to -28.0)	-10.5 (-18.5 to 0.4)	-8.7 (-19.1 to 5.5)	-4.5 (-13.0 to 7.2)	-79.0 (-81.8 to -75.9)	-2.9 (-15.4 to 14.8)	-75.0 (-78.3 to -71.2)	-2.6 (-15.1 to 15.2)

Mongolia	-38.0 (-45.6 to -28.6)	-7.9 (-13.3 to -1.6)	-2.6 (-14.4 to 12.3)	-0.8 (-6.6 to 6.0)	-91.4 (-92.6 to -89.9)	-9.2 (-19.0 to 1.9)	-90.0 (-91.4 to -88.3)	-7.4 (-17.5 to 3.9)
Tajikistan	-39.0 (-45.9 to -32.5)	-11.3 (-19.1 to -2.3)	7.6 (-4.5 to 19.3)	-3.3 (-11.7 to 6.5)	-90.1 (-91.9 to -88.0)	-17.1 (-28.6 to -1.9)	-87.3 (-89.6 to -84.6)	-13.2 (-25.3 to 2.6)
Turkmenistan	-40.1 (-48.0 to -27.3)	-9.3 (-18.9 to 2.5)	-17.8 (-28.6 to -0.3)	-5.2 (-15.1 to 7.3)	-89.6 (-91.4 to -87.1)	-11.3 (-26.9 to 6.0)	-90.3 (-91.9 to -87.9)	-11.1 (-26.8 to 6.2)
Uzbekistan	-39.9 (-46.7 to -30.4)	-11.6 (-18.0 to -4.2)	-3.3 (-14.2 to 11.9)	-6.0 (-12.9 to 1.8)	-92.5 (-93.7 to -91.3)	-19.1 (-28.7 to -7.2)	-92.0 (-93.2 to -90.7)	-19.7 (-29.2 to -7.9)
Central Europe	-37.9 (-40.5 to -35.0)	-5.6 (-7.9 to -3.2)	-42.3 (-44.7 to -39.6)	-6.7 (-9.0 to -4.3)	-87.6 (-88.4 to -86.8)	0.3 (-5.1 to 6.1)	-92.2 (-92.6 to -91.6)	-2.7 (-7.9 to 2.9)
Albania	-35.1 (-42.5 to -25.3)	-6.4 (-12.6 to 1.8)	-46.7 (-52.8 to -38.6)	-8.4 (-14.4 to -0.3)	-94.2 (-95.1 to -93.1)	0.8 (-12.9 to 16.2)	-97.6 (-98.0 to -97.2)	-1.5 (-15.0 to 13.4)
Bosnia and Herzegovina	-17.1 (-23.8 to -7.8)	-3.5 (-10.0 to 4.1)	-39.7 (-44.6 to -33.0)	-9.0 (-15.1 to -1.8)	-73.5 (-76.7 to -69.7)	17.8 (4.0 to 32.3)	-89.1 (-90.4 to -87.6)	4.0 (-8.2 to 16.7)
Bulgaria	-39.5 (-41.6 to -37.3)	-8.4 (-11.1 to -5.9)	-51.7 (-53.3 to -49.9)	-11.1 (-13.7 to -8.6)	-88.8 (-90.3 to -87.2)	-13.5 (-24.0 to -2.8)	-93.5 (-94.3 to -92.5)	-18.0 (-27.9 to -7.8)
Croatia	-35.2 (-39.9 to -30.8)	-7.8 (-13.3 to -2.8)	-43.8 (-47.9 to -40.0)	-9.2 (-14.6 to -4.2)	-91.2 (-92.2 to -90.3)	9.6 (-1.4 to 20.6)	-94.6 (-95.2 to -94.0)	-0.1 (-10.2 to 9.8)
Czech Republic	-26.1 (-33.4 to -17.7)	-6.1 (-14.7 to 3.8)	-23.6 (-31.2 to -14.9)	-5.6 (-14.2 to 4.4)	-93.9 (-94.8 to -93.0)	9.8 (-5.4 to 27.0)	-94.7 (-95.4 to -93.8)	12.8 (-2.8 to 30.4)
Hungary	-8.3 (-14.5 to -2.0)	-1.2 (-6.8 to 5.6)	-14.6 (-20.4 to -8.8)	-2.6 (-8.1 to 4.1)	-16.4 (-28.8 to -2.8)	1.3 (-13.3 to 19.4)	-40.3 (-49.1 to -30.5)	-2.0 (-16.1 to 15.5)
Montenegro	-17.2 (-24.4 to -8.3)	-5.2 (-13.9 to 5.5)	-17.9 (-25.1 to -9.1)	-6.0 (-14.6 to 4.6)	-79.1 (-82.2 to -76.0)	-7.5 (-18.6 to 5.4)	-86.3 (-88.3 to -84.2)	-12.5 (-22.9 to -0.2)
North Macedonia	-19.7 (-26.8 to -11.3)	-4.3 (-13.2 to 6.0)	-14.2 (-21.8 to -5.2)	-3.8 (-12.7 to 6.7)	-85.9 (-87.4 to -83.6)	-0.2 (-11.2 to 15.2)	-90.9 (-91.8 to -89.4)	-2.5 (-13.2 to 12.6)
Poland	-52.0 (-55.4 to -48.6)	-7.1 (-9.7 to -4.3)	-51.7 (-55.0 to -48.3)	-7.2 (-9.8 to -4.4)	-94.5 (-94.8 to -94.1)	-8.2 (-11.1 to -5.5)	-96.3 (-96.5 to -96.1)	-10.3 (-13.1 to -7.6)
Romania	-31.1 (-37.4 to -23.7)	-0.6 (-8.7 to 9.7)	-43.3 (-48.5 to -37.2)	-3.5 (-11.4 to 6.4)	-84.2 (-86.3 to -82.0)	13.5 (-0.7 to 31.6)	-91.7 (-92.8 to -90.5)	9.7 (-4.0 to 27.2)
Serbia	-25.6 (-31.7 to -16.8)	-4.2 (-12.2 to 4.2)	-30.7 (-36.5 to -22.6)	-5.7 (-13.6 to 2.6)	-89.0 (-90.5 to -87.1)	4.3 (-7.7 to 18.1)	-92.3 (-93.4 to -91.0)	-2.2 (-13.5 to 10.8)
Slovakia	-29.3 (-36.1 to -21.2)	-7.0 (-15.1 to 1.7)	-27.2 (-34.3 to -18.9)	-6.8 (-14.8 to 1.9)	-93.3 (-94.2 to -92.3)	12.6 (-5.1 to 29.3)	-95.3 (-95.9 to -94.6)	13.9 (-4.0 to 30.8)
Slovenia	-25.3 (-32.6 to -16.4)	-5.9 (-14.6 to 3.8)	-21.4 (-29.1 to -12.1)	-5.7 (-14.3 to 4.1)	-87.7 (-89.4 to -85.6)	-9.4 (-23.7 to 6.8)	-89.9 (-91.3 to -88.2)	-15.7 (-29.1 to -0.7)
Eastern Europe	-31.3 (-35.0 to -27.7)	-11.1 (-13.6 to -8.3)	-36.3 (-39.8 to -32.9)	-11.6 (-14.1 to -8.8)	-90.9 (-91.5 to -90.3)	-61.7 (-64.9 to -58.6)	-93.5 (-93.9 to -93.0)	-64.0 (-67.0 to -61.0)
Belarus	-36.3 (-43.0 to -28.4)	-9.1 (-17.5 to 0.3)	-42.2 (-48.3 to -35.1)	-9.8 (-18.1 to -0.5)	-95.0 (-95.6 to -94.2)	-1.1 (-15.1 to 18.2)	-96.5 (-97.0 to -96.0)	-4.8 (-18.3 to 13.7)
Estonia	-28.0 (-36.4 to -17.2)	-4.9 (-13.6 to 5.5)	-39.8 (-46.8 to -30.7)	-5.1 (-13.7 to 5.3)	-93.4 (-94.4 to -92.2)	15.5 (-2.9 to 35.2)	-96.2 (-96.8 to -95.5)	12.0 (-5.8 to 31.2)
Latvia	-37.8 (-44.6 to -30.5)	-7.9 (-18.5 to 1.9)	-55.2 (-60.1 to -49.9)	-11.3 (-21.5 to -1.8)	-94.8 (-95.5 to -94.0)	-4.8 (-18.2 to 9.7)	-97.3 (-97.7 to -96.9)	-4.6 (-18.0 to 10.0)
Lithuania	-34.6 (-41.9 to -25.6)	-7.3 (-15.6 to 2.9)	-50.2 (-55.8 to -43.4)	-11.2 (-19.0 to -1.4)	-94.5 (-95.2 to -93.5)	0.7 (-12.1 to 16.3)	-97.2 (-97.6 to -96.7)	-4.3 (-16.5 to 10.5)

Moldova	-20.4 (-27.4 to -12.6)	-1.1 (-9.1 to 6.8)	-34.0 (-39.8 to -27.5)	-3.3 (-11.1 to 4.4)	-66.6 (-70.3 to -61.4)	-4.8 (-19.4 to 9.4)	-86.4 (-87.9 to -84.3)	-16.7 (-29.5 to -4.3)
Russia	-34.3 (-37.6 to -30.9)	-11.8 (-13.9 to -9.8)	-36.2 (-39.4 to -32.9)	-11.5 (-13.7 to -9.5)	-95.4 (-95.6 to -95.2)	2.3 (-0.5 to 5.3)	-96.4 (-96.5 to -96.2)	-2.5 (-5.2 to 0.4)
Ukraine	-22.4 (-28.3 to -16.1)	-9.9 (-15.6 to -3.1)	-35.1 (-40.1 to -29.8)	-12.4 (-17.9 to -5.7)	-80.7 (-82.7 to -78.2)	-71.0 (-74.0 to -67.7)	-89.0 (-90.1 to -87.5)	-74.3 (-77.0 to -71.4)
High income	-25.5 (-28.1 to -22.7)	-4.2 (-5.8 to -2.5)	-11.2 (-14.2 to -7.8)	-2.6 (-4.2 to -0.9)	-86.1 (-86.6 to -85.7)	-66.7 (-68.0 to -65.2)	-87.1 (-87.6 to -86.7)	-67.6 (-68.9 to -66.2)
Australasia	-22.4 (-27.9 to -15.4)	-5.2 (-11.7 to 2.3)	11.2 (3.4 to 21.2)	-0.4 (-7.3 to 7.4)	-91.2 (-92.0 to -90.2)	-9.9 (-17.9 to -0.8)	-89.6 (-90.6 to -88.5)	-8.7 (-16.7 to 0.5)
Australia	-22.4 (-28.2 to -15.0)	-5.8 (-12.7 to 2.2)	13.0 (4.6 to 23.9)	-0.6 (-7.9 to 7.8)	-92.2 (-93.0 to -91.4)	-10.6 (-19.5 to -0.6)	-90.6 (-91.6 to -89.6)	-9.1 (-18.2 to 1.2)
New Zealand	-30.5 (-36.7 to -23.0)	-0.7 (-7.2 to 6.3)	-8.5 (-16.8 to 1.3)	1.5 (-5.2 to 8.6)	-73.0 (-75.2 to -70.6)	-6.3 (-14.6 to 1.7)	-71.6 (-73.9 to -69.0)	-6.7 (-15.0 to 1.2)
High-income Asia Pacific	-21.0 (-24.3 to -17.6)	-2.5 (-5.0 to -0.1)	-14.8 (-18.2 to -11.0)	-2.3 (-4.8 to 0.1)	-91.3 (-91.9 to -90.9)	-88.2 (-88.9 to -87.6)	-93.8 (-94.2 to -93.5)	-89.0 (-89.6 to -88.4)
Brunei	-54.6 (-59.5 to -49.0)	-15.7 (-23.9 to -7.9)	-23.3 (-31.5 to -13.8)	-12.0 (-20.6 to -3.8)	-60.2 (-65.3 to -54.5)	-19.6 (-30.1 to -7.5)	-63.9 (-68.5 to -58.7)	-20.3 (-30.8 to -8.4)
Japan	-7.4 (-11.4 to -3.7)	-0.6 (-3.7 to 2.6)	-6.0 (-10.0 to -2.3)	-1.7 (-4.8 to 1.5)	-93.0 (-93.2 to -92.8)	-92.5 (-92.7 to -92.2)	-94.9 (-95.1 to -94.8)	-93.0 (-93.3 to -92.8)
South Korea	-43.9 (-47.7 to -39.8)	-7.1 (-10.7 to -2.6)	-32.5 (-37.0 to -27.5)	-4.2 (-7.9 to 0.5)	-87.1 (-89.1 to -85.1)	-0.4 (-13.8 to 14.7)	-91.7 (-93.0 to -90.4)	-6.1 (-18.8 to 8.1)
Singapore	-31.3 (-36.6 to -25.9)	1.6 (-3.6 to 7.4)	27.8 (18.0 to 37.8)	4.0 (-1.3 to 10.0)	-76.2 (-79.3 to -71.7)	-4.6 (-14.2 to 7.6)	-66.3 (-70.6 to -59.9)	-4.5 (-14.1 to 7.7)
High-income North America	-27.5 (-31.0 to -23.1)	-5.4 (-7.8 to -2.8)	-5.9 (-10.5 to -0.2)	-3.1 (-5.6 to -0.5)	-72.1 (-73.4 to -70.5)	-10.9 (-15.9 to -4.9)	-72.7 (-74.1 to -71.2)	-12.6 (-17.5 to -6.7)
Canada	-11.1 (-16.1 to -6.2)	-2.4 (-7.8 to 2.7)	19.1 (12.4 to 25.7)	0.8 (-4.7 to 6.0)	-68.2 (-71.3 to -64.7)	-18.8 (-27.7 to -8.0)	-68.2 (-71.3 to -64.7)	-19.9 (-28.7 to -9.3)
Greenland	-5.8 (-16.4 to 7.1)	-1.1 (-10.4 to 8.5)	-4.8 (-15.5 to 8.3)	-1.2 (-10.6 to 8.3)	-9.3 (-24.8 to 10.0)	-1.8 (-16.2 to 10.2)	-34.0 (-45.3 to -20.0)	0.6 (-14.2 to 12.8)
USA	-34.9 (-38.7 to -30.2)	-7.3 (-9.8 to -4.8)	-15.9 (-20.7 to -9.7)	-5.2 (-7.8 to -2.6)	-75.1 (-76.2 to -74.1)	-3.4 (-7.1 to 0.3)	-75.7 (-76.8 to -74.8)	-5.3 (-8.9 to -1.6)
Southern Latin America	-13.0 (-19.7 to -3.4)	-0.5 (-7.0 to 6.0)	17.2 (8.2 to 30.1)	3.3 (-3.4 to 10.1)	-86.6 (-88.4 to -82.7)	7.4 (-6.3 to 26.5)	-87.4 (-89.1 to -83.7)	2.9 (-10.2 to 21.3)
Argentina	-15.8 (-24.0 to -1.8)	-0.9 (-9.4 to 8.4)	14.7 (3.5 to 33.8)	2.9 (-6.0 to 12.5)	-85.2 (-88.0 to -78.3)	10.8 (-4.7 to 38.8)	-85.1 (-87.9 to -78.1)	5.8 (-9.0 to 32.5)
Chile	-7.1 (-15.7 to 2.6)	0.7 (-9.5 to 11.6)	27.3 (15.6 to 40.6)	5.2 (-5.5 to 16.5)	-89.6 (-91.3 to -87.1)	-0.7 (-21.9 to 26.0)	-91.7 (-93.1 to -89.7)	-4.2 (-24.6 to 21.6)
Uruguay	-22.6 (-29.6 to -14.7)	-7.5 (-16.0 to 0.8)	-15.2 (-22.9 to -6.7)	-6.6 (-15.2 to 1.7)	-89.0 (-90.7 to -86.7)	-9.9 (-24.0 to 10.5)	-90.7 (-92.1 to -88.8)	-11.1 (-25.1 to 9.0)
Western Europe	-22.5 (-24.8 to -19.7)	-4.7 (-6.3 to -3.2)	-12.0 (-14.7 to -8.9)	-3.5 (-5.1 to -2.0)	-84.6 (-85.5 to -83.9)	-49.6 (-51.4 to -47.9)	-85.2 (-86.1 to -84.6)	-50.7 (-52.5 to -49.1)
Andorra	-23.5 (-30.9 to -14.6)	-4.2 (-12.5 to 7.2)	17.5 (6.0 to 31.1)	2.2 (-6.7 to 14.3)	-92.9 (-93.9 to -91.5)	-25.4 (-38.7 to -9.8)	-92.9 (-93.9 to -91.5)	-38.2 (-49.2 to -25.2)
Austria	-21.1 (-29.1 to -12.6)	-5.6 (-15.2 to 3.4)	-9.4 (-18.7 to 0.4)	-2.8 (-12.7 to 6.4)	-86.8 (-89.8 to -83.4)	26.0 (2.1 to 54.8)	-87.0 (-90.0 to -83.7)	34.8 (9.3 to 65.6)

Belgium	-25.9 (-30.8 to -20.4)	-5.7 (-11.3 to 0.4)	-15.2 (-20.8 to -8.9)	-4.0 (-9.7 to 2.1)	-94.4 (-95.1 to -93.5)	-4.2 (-18.4 to 11.2)	-94.2 (-94.9 to -93.2)	-7.4 (-21.1 to 7.5)
Cyprus	-40.6 (-42.3 to -39.0)	-6.0 (-8.2 to -4.0)	0.3 (-2.5 to 3.0)	0.1 (-2.2 to 2.3)	-92.5 (-93.4 to -91.5)	-2.6 (-14.2 to 11.9)	-91.3 (-92.3 to -90.2)	4.4 (-8.0 to 20.0)
Denmark	-4.5 (-14.4 to 5.1)	-2.5 (-11.8 to 7.2)	7.8 (-3.5 to 18.5)	-0.3 (-9.8 to 9.7)	-2.9 (-18.7 to 15.1)	-4.3 (-19.0 to 17.3)	3.4 (-13.5 to 22.5)	-1.5 (-16.6 to 20.7)
Finland	-7.7 (-16.8 to 2.7)	-2.1 (-11.0 to 9.3)	2.0 (-8.1 to 13.5)	-1.4 (-10.4 to 10.1)	-6.6 (-20.1 to 12.3)	-4.3 (-17.9 to 16.2)	-22.1 (-33.3 to -6.3)	-14.9 (-27.0 to 3.3)
France	-21.0 (-23.6 to -17.8)	-4.0 (-6.8 to -1.1)	-9.4 (-12.5 to -5.8)	-2.8 (-5.6 to 0.2)	-88.5 (-89.9 to -87.0)	-27.2 (-36.4 to -17.8)	-89.2 (-90.5 to -87.8)	-31.5 (-40.1 to -22.6)
Germany	-17.4 (-24.4 to -8.6)	-3.5 (-11.0 to 4.4)	-12.3 (-19.7 to -3.0)	-1.2 (-8.8 to 6.9)	-89.4 (-91.1 to -87.2)	-16.2 (-33.6 to 11.8)	-90.7 (-92.2 to -88.8)	-9.7 (-28.5 to 20.4)
Greece	-19.1 (-24.3 to -11.7)	-0.8 (-8.0 to 5.7)	-19.5 (-24.7 to -12.1)	-3.1 (-10.2 to 3.2)	-93.5 (-94.2 to -92.7)	-4.2 (-17.7 to 11.4)	-94.7 (-95.3 to -94.1)	-8.8 (-21.6 to 6.0)
Iceland	-10.0 (-19.8 to 0.8)	-2.7 (-12.2 to 10.3)	22.2 (9.0 to 36.8)	1.8 (-8.2 to 15.3)	-15.4 (-31.3 to 7.1)	-6.8 (-24.9 to 22.2)	-15.4 (-31.3 to 7.1)	-12.8 (-29.8 to 14.3)
Ireland	-21.3 (-29.6 to -10.6)	-6.3 (-16.5 to 4.4)	7.3 (-4.0 to 21.9)	-4.4 (-14.8 to 6.6)	-92.0 (-93.4 to -90.3)	-13.1 (-28.9 to 17.9)	-91.2 (-92.7 to -89.3)	-21.1 (-35.4 to 7.2)
Israel	-36.2 (-41.9 to -28.9)	-10.3 (-18.5 to -1.0)	19.8 (9.0 to 33.4)	-3.3 (-12.2 to 6.7)	-90.7 (-91.8 to -89.4)	-2.0 (-13.6 to 15.5)	-83.0 (-84.9 to -80.5)	6.0 (-6.5 to 25.0)
Italy	-42.6 (-45.9 to -38.9)	-6.7 (-10.2 to -3.1)	-39.1 (-42.6 to -35.1)	-7.4 (-10.9 to -3.8)	-85.9 (-86.9 to -84.7)	-1.2 (-7.6 to 4.7)	-87.9 (-88.7 to -86.9)	-11.8 (-17.5 to -6.6)
Luxembourg	-18.7 (-26.9 to -8.6)	-4.5 (-12.1 to 4.6)	32.0 (18.7 to 48.3)	4.9 (-3.5 to 14.8)	-75.4 (-80.0 to -69.8)	39.9 (19.1 to 60.2)	-65.1 (-71.7 to -57.2)	43.5 (22.1 to 64.3)
Malta	-25.4 (-34.3 to -14.4)	-5.2 (-14.6 to 4.7)	-11.6 (-22.2 to 1.4)	-3.9 (-13.4 to 6.2)	-86.0 (-87.9 to -83.4)	-16.6 (-27.3 to -0.5)	-89.3 (-90.7 to -87.2)	-14.4 (-25.4 to 2.2)
Monaco	-26.7 (-33.7 to -17.9)	-7.4 (-17.6 to 2.2)	-9.5 (-18.2 to 1.3)	-5.7 (-16.0 to 4.1)	-95.2 (-96.0 to -93.9)	1.9 (-14.3 to 24.4)	-93.3 (-94.4 to -91.6)	4.6 (-12.0 to 27.8)
Netherlands	-7.5 (-14.9 to 1.4)	-6.0 (-14.5 to 2.6)	6.4 (-2.2 to 16.5)	-4.7 (-13.3 to 4.1)	-87.1 (-88.5 to -85.4)	-31.0 (-38.9 to -21.7)	-87.9 (-89.2 to -86.3)	-31.3 (-39.2 to -22.1)
Norway	-29.2 (-31.9 to -26.4)	-8.2 (-10.6 to -5.6)	-10.8 (-14.2 to -7.3)	-5.0 (-7.5 to -2.3)	-90.3 (-90.7 to -89.8)	-9.4 (-13.8 to -4.7)	-89.7 (-90.1 to -89.1)	-13.3 (-17.5 to -8.8)
Portugal	-27.9 (-35.0 to -19.5)	-7.6 (-15.9 to 2.5)	-24.2 (-31.7 to -15.5)	-8.4 (-16.6 to 1.6)	-93.6 (-94.7 to -92.3)	-6.1 (-21.7 to 17.4)	-95.4 (-96.2 to -94.5)	-11.1 (-25.8 to 11.3)
San Marino	-21.9 (-29.3 to -12.5)	3.1 (-7.2 to 15.2)	9.7 (-0.7 to 23.0)	5.6 (-5.0 to 18.0)	-73.9 (-79.2 to -68.8)	-1.4 (-20.5 to 22.3)	-69.7 (-75.9 to -63.9)	-4.4 (-22.9 to 18.7)
Spain	-28.8 (-35.2 to -21.9)	-7.2 (-13.9 to 0.1)	-15.5 (-23.2 to -7.3)	-8.7 (-15.2 to -1.4)	-92.9 (-94.2 to -91.6)	-7.9 (-22.8 to 12.7)	-93.2 (-94.4 to -91.9)	-17.4 (-30.7 to 1.0)
Sweden	-6.6 (-11.4 to -0.7)	-2.0 (-7.8 to 3.8)	11.2 (5.4 to 18.2)	1.6 (-4.4 to 7.6)	-72.0 (-75.1 to -68.8)	-41.7 (-47.6 to -35.6)	-70.4 (-73.7 to -67.0)	-40.5 (-46.5 to -34.2)
Switzerland	-6.5 (-14.8 to 2.2)	-2.5 (-11.8 to 7.7)	19.5 (8.9 to 30.6)	1.5 (-8.2 to 12.1)	-57.8 (-64.7 to -50.0)	-44.2 (-53.3 to -34.4)	-52.5 (-60.3 to -43.7)	-41.5 (-51.1 to -31.3)
UK	0.1 (-3.6 to 4.2)	-1.2 (-3.4 to 0.8)	17.1 (12.8 to 21.9)	0.9 (-1.3 to 3.0)	-79.9 (-80.7 to -79.2)	-78.3 (-79.0 to -77.7)	-79.6 (-80.3 to -78.8)	-78.5 (-79.1 to -77.9)
Latin America and Caribbean	-32.4 (-36.6 to -27.1)	-5.0 (-7.4 to -2.6)	1.2 (-5.1 to 9.1)	-1.5 (-4.0 to 1.0)	-90.8 (-91.2 to -90.3)	-5.5 (-8.8 to -1.5)	-91.1 (-91.6 to -90.7)	-8.7 (-11.9 to -4.8)

Andean Latin America	-9.3 (-15.2 to -2.6)	-4.1 (-8.9 to 0.4)	51.0 (41.2 to 62.2)	2.4 (-2.7 to 7.2)	-79.4 (-81.4 to -77.0)	-3.6 (-12.9 to 4.2)	-76.0 (-78.3 to -73.3)	-1.8 (-11.3 to 6.2)
Bolivia	-9.3 (-17.1 to -0.2)	-2.5 (-14.1 to 6.9)	69.7 (55.0 to 86.7)	4.9 (-7.6 to 15.0)	-87.4 (-89.2 to -85.7)	-20.6 (-35.8 to -9.0)	-81.9 (-84.4 to -79.5)	-17.0 (-32.8 to -4.8)
Ecuador	-12.7 (-20.9 to -3.0)	-4.4 (-12.1 to 4.0)	53.2 (38.8 to 70.2)	3.4 (-5.1 to 12.3)	-76.7 (-79.7 to -72.9)	0.5 (-14.5 to 20.1)	-71.3 (-75.0 to -66.6)	3.9 (-11.5 to 24.2)
Peru	-8.1 (-16.5 to 0.6)	-4.6 (-11.1 to 3.2)	43.8 (30.6 to 57.4)	0.9 (-5.9 to 9.2)	-76.5 (-79.6 to -73.0)	1.1 (-12.2 to 15.6)	-75.7 (-78.9 to -72.1)	0.9 (-12.4 to 15.4)
Caribbean	-11.0 (-17.6 to -4.8)	-1.2 (-7.4 to 5.3)	19.0 (10.2 to 27.3)	1.8 (-4.6 to 8.5)	-67.4 (-71.0 to -63.7)	-0.3 (-10.7 to 12.3)	-68.9 (-72.4 to -65.4)	-2.1 (-12.4 to 10.3)
Antigua and Barbuda	-28.4 (-36.1 to -20.3)	-5.5 (-15.1 to 4.0)	4.4 (-6.9 to 16.1)	-4.5 (-14.2 to 5.1)	-90.7 (-92.0 to -89.2)	6.2 (-11.8 to 23.3)	-92.7 (-93.7 to -91.6)	-1.8 (-18.4 to 14.0)
The Bahamas	-21.1 (-28.3 to -10.4)	-4.6 (-13.5 to 5.5)	16.0 (5.5 to 31.7)	-2.5 (-11.5 to 7.9)	-90.5 (-92.0 to -88.9)	-1.1 (-11.8 to 14.0)	-91.8 (-93.1 to -90.4)	-8.7 (-18.6 to 5.3)
Barbados	-20.5 (-29.2 to -9.9)	-1.9 (-10.1 to 8.0)	-6.7 (-17.0 to 5.7)	0.0 (-8.3 to 10.1)	-88.5 (-90.0 to -86.5)	-2.3 (-14.3 to 11.1)	-91.6 (-92.7 to -90.1)	-8.4 (-19.7 to 4.1)
Belize	-33.8 (-41.6 to -25.3)	2.1 (-8.4 to 15.0)	46.0 (28.9 to 64.8)	11.6 (0.1 to 25.7)	-87.0 (-88.9 to -85.1)	-15.8 (-26.8 to -2.8)	-82.9 (-85.4 to -80.3)	-14.1 (-25.4 to -0.8)
Bermuda	-6.4 (-14.3 to 2.8)	-0.7 (-10.0 to 10.7)	0.9 (-7.6 to 10.7)	-2.0 (-11.2 to 9.2)	-13.0 (-25.8 to 2.8)	-3.7 (-15.8 to 10.0)	-47.0 (-54.8 to -37.4)	-15.8 (-26.3 to -3.8)
Cuba	-28.7 (-36.4 to -20.6)	2.1 (-6.0 to 11.8)	-25.3 (-33.3 to -16.8)	1.3 (-6.7 to 10.9)	-49.7 (-57.0 to -40.4)	-2.3 (-17.5 to 16.1)	-68.4 (-73.0 to -62.6)	-12.8 (-26.4 to 3.6)
Dominica	-18.3 (-26.6 to -8.6)	-5.7 (-15.1 to 3.7)	-24.2 (-31.9 to -15.2)	-6.4 (-15.7 to 3.0)	-90.3 (-91.7 to -88.8)	7.8 (-8.7 to 26.8)	-95.0 (-95.8 to -94.3)	0.5 (-14.9 to 18.1)
Dominican Republic	-33.2 (-40.0 to -25.6)	-7.8 (-14.8 to -0.2)	0.9 (-9.4 to 12.4)	-3.5 (-10.8 to 4.4)	-72.6 (-76.0 to -68.6)	-20.5 (-31.9 to -10.0)	-71.2 (-74.8 to -67.0)	-18.4 (-30.2 to -7.7)
Grenada	-26.6 (-35.6 to -16.9)	2.5 (-7.1 to 11.5)	-11.5 (-22.5 to 0.2)	1.3 (-8.2 to 10.2)	-86.7 (-88.7 to -84.5)	10.0 (-6.3 to 27.7)	-91.2 (-92.6 to -89.7)	2.5 (-12.6 to 19.0)
Guyana	-26.8 (-34.5 to -17.5)	-10.0 (-18.3 to -0.3)	-26.8 (-34.4 to -17.4)	-5.8 (-14.5 to 4.3)	-89.2 (-91.1 to -87.6)	-14.4 (-26.6 to -2.3)	-92.6 (-93.8 to -91.4)	-9.8 (-22.7 to 2.9)
Haiti	-12.8 (-22.6 to -2.0)	-2.6 (-13.7 to 9.4)	70.1 (51.1 to 91.2)	5.7 (-6.3 to 18.8)	-74.8 (-78.9 to -70.7)	4.4 (-10.8 to 23.8)	-63.1 (-69.0 to -57.1)	7.4 (-8.3 to 27.3)
Jamaica	-32.9 (-39.3 to -25.8)	-7.2 (-13.2 to -0.3)	-20.2 (-27.9 to -11.8)	-6.5 (-12.6 to 0.5)	-94.2 (-95.0 to -93.1)	-16.9 (-27.2 to -4.9)	-96.1 (-96.7 to -95.4)	-22.2 (-31.9 to -10.9)
Puerto Rico	-29.3 (-37.9 to -19.2)	-2.2 (-11.9 to 7.5)	-31.1 (-39.4 to -21.2)	-7.1 (-16.4 to 2.1)	-67.2 (-71.0 to -62.4)	1.2 (-12.5 to 21.0)	-86.5 (-88.1 to -84.5)	-25.6 (-35.7 to -11.1)
Saint Kitts and Nevis	-25.3 (-33.9 to -14.7)	-5.1 (-14.3 to 4.1)	7.4 (-4.8 to 22.8)	-1.7 (-11.2 to 7.9)	-92.8 (-93.8 to -91.9)	-13.8 (-26.2 to 3.3)	-94.5 (-95.2 to -93.7)	-18.8 (-30.4 to -2.6)
Saint Lucia	-18.5 (-26.3 to -10.0)	-4.1 (-13.2 to 6.3)	3.6 (-6.3 to 14.4)	-3.0 (-12.3 to 7.5)	-85.5 (-87.8 to -83.3)	40.7 (16.0 to 65.5)	-92.7 (-93.9 to -91.6)	27.6 (5.2 to 50.2)
Saint Vincent and the Grenadines	-26.3 (-35.1 to -17.4)	-5.0 (-13.5 to 4.9)	-24.3 (-33.3 to -15.1)	-4.5 (-13.1 to 5.4)	-95.2 (-95.8 to -94.4)	-1.9 (-15.8 to 14.7)	-97.2 (-97.5 to -96.7)	-8.9 (-21.8 to 6.6)
Suriname	-28.4 (-35.1 to -20.7)	-8.2 (-14.3 to -0.9)	6.6 (-3.3 to 18.1)	-6.2 (-12.4 to 1.2)	-93.3 (-94.2 to -92.4)	5.4 (-6.7 to 25.3)	-93.0 (-93.9 to -92.1)	0.1 (-11.3 to 19.0)
Trinidad and Tobago	-19.3 (-29.3 to -9.0)	-4.7 (-12.7 to 4.3)	-6.9 (-18.5 to 4.9)	-3.9 (-11.9 to 5.3)	-82.0 (-85.4 to -78.5)	2.3 (-11.6 to 26.9)	-88.6 (-90.8 to -86.4)	-6.9 (-19.5 to 15.6)

Virgin Islands	-25.3 (-32.5 to -16.1)	-1.7 (-10.4 to 8.5)	-26.7 (-33.7 to -17.7)	-3.6 (-12.1 to 6.4)	-41.4 (-49.6 to -27.9)	7.5 (-7.9 to 32.5)	-65.5 (-70.3 to -57.6)	-1.9 (-16.0 to 20.9)
Central Latin America	-33.3 (-39.5 to -25.9)	-7.2 (-12.3 to -1.6)	1.6 (-7.8 to 12.9)	-4.2 (-9.5 to 1.6)	-85.1 (-86.1 to -83.9)	-11.4 (-17.1 to -4.3)	-85.9 (-86.9 to -84.8)	-14.8 (-20.3 to -8.0)
Colombia	-35.1 (-43.4 to -24.9)	-10.5 (-18.8 to -1.8)	-4.7 (-16.9 to 10.3)	-6.7 (-15.4 to 2.3)	-87.5 (-88.8 to -85.8)	-15.9 (-26.0 to -4.7)	-88.7 (-89.9 to -87.2)	-15.6 (-25.8 to -4.4)
Costa Rica	-26.3 (-34.7 to -15.9)	-0.0 (-9.0 to 10.1)	14.3 (1.4 to 30.6)	2.9 (-6.3 to 13.3)	-95.5 (-96.4 to -94.7)	-19.6 (-31.4 to -4.8)	-96.2 (-96.9 to -95.6)	-22.8 (-34.1 to -8.6)
El Salvador	-41.6 (-49.5 to -32.7)	-9.9 (-18.8 to 1.3)	-30.7 (-40.0 to -20.1)	-8.1 (-17.2 to 3.3)	-89.1 (-90.9 to -87.2)	-4.1 (-16.0 to 9.2)	-91.6 (-93.0 to -90.1)	-6.1 (-17.8 to 6.9)
Guatemala	-27.0 (-34.8 to -18.6)	-0.9 (-10.8 to 10.9)	62.8 (45.4 to 81.5)	7.7 (-3.0 to 20.6)	-79.8 (-82.6 to -76.2)	-1.3 (-17.8 to 18.8)	-72.3 (-76.0 to -67.3)	0.9 (-16.0 to 21.3)
Honduras	-36.2 (-42.6 to -27.8)	-0.4 (-9.7 to 8.9)	33.1 (19.6 to 50.5)	8.8 (-1.4 to 18.9)	-88.0 (-89.5 to -86.0)	-9.2 (-22.4 to 4.3)	-83.6 (-85.5 to -80.7)	-4.2 (-18.1 to 10.1)
Mexico	-39.4 (-42.7 to -36.3)	-5.2 (-7.4 to -2.8)	-11.4 (-16.3 to -6.8)	-1.7 (-4.0 to 0.7)	-82.7 (-84.1 to -81.6)	-19.1 (-22.6 to -15.0)	-84.6 (-85.8 to -83.6)	-22.4 (-25.7 to -18.4)
Nicaragua	-50.6 (-51.9 to -49.1)	-12.2 (-14.0 to -10.2)	-17.2 (-19.5 to -14.8)	-7.4 (-9.2 to -5.3)	-96.4 (-96.5 to -96.2)	-5.5 (-10.0 to -1.0)	-96.5 (-96.6 to -96.3)	-7.1 (-11.5 to -2.5)
Panama	-35.7 (-44.0 to -27.3)	-10.5 (-18.4 to -1.2)	12.0 (-2.4 to 26.6)	-2.7 (-11.3 to 7.5)	-84.0 (-86.5 to -81.3)	-48.6 (-56.1 to -40.3)	-78.5 (-81.9 to -74.9)	-46.4 (-54.2 to -37.7)
Venezuela	-20.5 (-29.7 to -7.6)	-2.6 (-11.6 to 8.9)	18.6 (4.8 to 37.8)	-8.4 (-16.9 to 2.4)	-71.7 (-75.5 to -67.0)	-2.9 (-17.1 to 11.7)	-74.5 (-78.0 to -70.3)	-20.6 (-32.2 to -8.7)
Tropical Latin America	-32.4 (-36.5 to -27.1)	-4.5 (-7.5 to -1.8)	-1.1 (-7.1 to 6.7)	-1.0 (-4.1 to 1.8)	-94.4 (-94.7 to -94.2)	0.1 (-3.5 to 4.1)	-94.8 (-95.1 to -94.6)	-5.0 (-8.4 to -1.2)
Brazil	-32.6 (-36.7 to -27.3)	-4.4 (-7.4 to -1.7)	-1.8 (-7.9 to 5.9)	-1.0 (-4.1 to 1.8)	-94.5 (-94.7 to -94.2)	1.0 (-2.4 to 4.9)	-94.9 (-95.1 to -94.7)	-4.3 (-7.6 to -0.6)
Paraguay	-20.2 (-29.3 to -7.5)	-5.9 (-16.0 to 5.8)	36.7 (21.2 to 58.4)	-0.8 (-11.4 to 11.6)	-92.6 (-93.9 to -91.1)	-17.4 (-28.9 to 2.9)	-92.4 (-93.7 to -90.9)	-18.9 (-30.2 to 1.0)
North Africa and Middle East	-40.8 (-43.2 to -38.6)	-6.8 (-8.3 to -4.9)	4.4 (0.2 to 8.3)	-0.8 (-2.5 to 1.2)	-82.1 (-83.5 to -80.9)	-24.7 (-28.5 to -20.7)	-80.0 (-81.5 to -78.6)	-26.1 (-29.9 to -22.2)
Afghanistan	-30.1 (-36.7 to -22.3)	-8.9 (-15.5 to -1.6)	134.4 (112.0 to 160.4)	3.8 (-3.7 to 12.2)	-72.8 (-76.2 to -68.6)	-30.2 (-37.4 to -21.1)	-10.4 (-21.8 to 3.3)	-21.9 (-30.0 to -11.7)
Algeria	-34.0 (-40.1 to -26.7)	-8.3 (-15.5 to -0.9)	9.2 (-0.9 to 21.3)	-2.5 (-10.2 to 5.3)	-89.9 (-91.3 to -88.6)	-13.7 (-24.3 to -2.7)	-88.4 (-90.0 to -86.9)	-13.0 (-23.7 to -1.9)
Bahrain	-27.9 (-36.1 to -14.8)	-3.3 (-14.0 to 8.2)	104.6 (81.4 to 142.0)	-0.4 (-11.3 to 11.5)	-94.4 (-95.2 to -93.6)	0.1 (-14.2 to 15.2)	-93.7 (-94.6 to -92.8)	-11.3 (-24.0 to 2.1)
Egypt	-34.2 (-37.6 to -29.9)	-8.1 (-13.5 to -3.5)	17.1 (10.9 to 24.7)	-1.7 (-7.4 to 3.3)	-93.3 (-93.8 to -92.8)	-13.2 (-18.9 to -5.2)	-91.5 (-92.2 to -90.8)	-16.6 (-22.1 to -9.0)
Iran	-39.2 (-44.0 to -33.7)	-5.9 (-8.5 to -3.4)	-12.5 (-19.4 to -4.5)	-2.1 (-4.8 to 0.6)	-94.6 (-94.9 to -94.3)	-9.6 (-12.8 to -6.2)	-95.8 (-96.0 to -95.6)	-12.6 (-15.7 to -9.3)
Iraq	-47.8 (-53.7 to -41.7)	-8.1 (-16.7 to 1.9)	24.9 (10.7 to 39.6)	-0.8 (-10.0 to 10.1)	-78.6 (-81.2 to -75.7)	-47.9 (-55.3 to -40.4)	-68.3 (-72.2 to -64.1)	-48.2 (-55.7 to -40.8)
Jordan	-62.7 (-63.8 to -61.4)	-10.6 (-12.5 to -8.4)	15.0 (11.7 to 19.0)	7.2 (4.9 to 9.8)	-90.3 (-90.8 to -89.9)	-7.9 (-12.3 to -3.3)	-81.3 (-82.1 to -80.4)	-0.6 (-5.4 to 4.4)
Kuwait	-41.4 (-48.6 to -32.2)	2.5 (-8.5 to 15.3)	47.6 (29.4 to 70.5)	19.3 (6.5 to 34.2)	-80.3 (-83.1 to -77.1)	-6.2 (-19.3 to 9.5)	-71.9 (-75.9 to -67.4)	-3.4 (-17.0 to 12.7)

Lebanon	-35.9 (-42.1 to -29.2)	-7.4 (-14.9 to 0.5)	1.3 (-8.5 to 11.9)	-4.3 (-12.1 to 3.8)	-80.5 (-83.0 to -77.5)	-12.9 (-23.4 to 0.2)	-79.7 (-82.3 to -76.6)	-19.5 (-29.2 to -7.4)
Libya	-37.0 (-42.8 to -30.3)	3.9 (-3.0 to 11.7)	0.2 (-9.0 to 10.8)	7.5 (0.4 to 15.5)	-92.0 (-93.2 to -90.6)	-7.4 (-19.2 to 6.0)	-95.1 (-95.9 to -94.3)	-17.3 (-27.9 to -5.4)
Morocco	-48.3 (-51.1 to -45.5)	-10.1 (-13.0 to -7.1)	-26.5 (-30.4 to -22.6)	-7.6 (-10.6 to -4.5)	-94.1 (-94.5 to -93.6)	-18.7 (-25.3 to -11.9)	-94.9 (-95.3 to -94.5)	-26.9 (-32.8 to -20.8)
Oman	-53.3 (-58.9 to -46.4)	0.3 (-11.4 to 13.9)	10.2 (-2.9 to 26.5)	11.5 (-1.6 to 26.6)	-60.6 (-64.7 to -56.7)	-8.3 (-15.6 to 0.8)	-50.9 (-56.0 to -46.0)	-3.3 (-10.9 to 6.4)
Palestine	-64.5 (-69.2 to -59.1)	-12.4 (-23.0 to -2.1)	-15.1 (-26.2 to -2.0)	-6.1 (-17.5 to 4.8)	-93.5 (-94.5 to -92.5)	-18.5 (-28.7 to -6.9)	-90.2 (-91.6 to -88.7)	-22.6 (-32.3 to -11.6)
Qatar	-35.8 (-41.8 to -30.5)	-18.3 (-24.9 to -12.3)	313.2 (274.6 to 347.5)	-4.4 (-12.1 to 2.6)	-44.0 (-50.6 to -36.9)	-0.6 (-13.7 to 12.0)	59.5 (40.7 to 79.9)	5.3 (-8.6 to 18.6)
Saudi Arabia	-63.7 (-69.2 to -58.7)	-8.3 (-16.2 to 1.3)	-19.2 (-31.5 to -8.0)	0.9 (-7.8 to 11.5)	-91.2 (-92.9 to -89.3)	-9.0 (-20.6 to 5.6)	-92.0 (-93.6 to -90.3)	-12.4 (-23.6 to 1.7)
Sudan	-50.4 (-54.1 to -46.0)	-10.5 (-14.8 to -6.0)	0.1 (-7.3 to 9.1)	-2.2 (-6.9 to 2.7)	-84.0 (-85.9 to -82.0)	-20.8 (-29.0 to -12.2)	-76.2 (-79.0 to -73.1)	-20.5 (-28.7 to -11.8)
Syria	-54.2 (-56.8 to -50.9)	-4.2 (-6.3 to -2.2)	-48.6 (-51.4 to -44.8)	-8.6 (-10.5 to -6.6)	-81.7 (-84.4 to -78.5)	-47.8 (-54.8 to -39.2)	-90.9 (-92.3 to -89.3)	-55.3 (-61.4 to -48.0)
Tunisia	-48.6 (-52.5 to -43.8)	-0.1 (-5.7 to 6.3)	-29.5 (-34.8 to -23.0)	2.6 (-3.1 to 9.3)	-92.4 (-93.2 to -91.5)	-11.9 (-19.7 to -1.1)	-94.2 (-94.8 to -93.5)	-18.2 (-25.5 to -8.2)
Turkey	-46.0 (-49.2 to -42.7)	-9.2 (-12.8 to -5.3)	-26.5 (-30.9 to -22.0)	-6.9 (-10.6 to -2.9)	-95.2 (-95.8 to -94.6)	-20.1 (-29.0 to -9.7)	-96.9 (-97.3 to -96.5)	-22.8 (-31.4 to -12.8)
United Arab Emirates	-12.2 (-22.0 to -1.0)	20.5 (7.5 to 34.8)	333.3 (285.1 to 388.7)	23.1 (9.8 to 37.6)	-13.8 (-23.5 to -0.4)	3.7 (-10.0 to 18.2)	25.8 (11.7 to 45.4)	-18.4 (-29.1 to -6.9)
Yemen	-47.6 (-51.6 to -43.3)	-4.4 (-10.3 to 2.0)	20.2 (11.1 to 30.1)	4.4 (-2.1 to 11.3)	-79.7 (-81.9 to -77.7)	-26.0 (-33.7 to -17.5)	-68.3 (-71.6 to -65.1)	-29.5 (-36.8 to -21.4)
South Asia	-23.6 (-26.9 to -20.4)	-5.2 (-7.5 to -3.0)	25.6 (20.3 to 30.9)	-0.1 (-2.5 to 2.2)	-78.4 (-79.2 to -77.4)	-21.5 (-24.9 to -18.8)	-78.0 (-78.9 to -77.0)	-24.3 (-27.5 to -21.6)
Bangladesh	-29.3 (-35.0 to -23.5)	-5.6 (-10.7 to 0.7)	3.2 (-5.1 to 11.7)	-1.7 (-7.1 to 4.8)	-92.3 (-93.1 to -91.4)	-17.1 (-24.6 to -7.1)	-94.2 (-94.8 to -93.5)	-21.5 (-28.6 to -12.1)
Bhutan	-54.6 (-57.0 to -49.5)	-11.7 (-14.1 to -8.8)	-44.0 (-47.0 to -37.8)	-12.7 (-15.1 to -9.9)	-96.5 (-96.8 to -96.0)	-8.2 (-16.0 to 2.5)	-97.6 (-97.8 to -97.3)	-17.4 (-24.5 to -7.8)
India	-20.9 (-24.5 to -17.4)	-4.2 (-6.8 to -1.7)	28.6 (22.7 to 34.3)	0.6 (-2.2 to 3.2)	-77.8 (-78.9 to -76.6)	-22.5 (-26.6 to -19.2)	-78.4 (-79.4 to -77.2)	-25.8 (-29.8 to -22.6)
Nepal	-34.2 (-38.8 to -28.7)	-6.9 (-12.0 to -1.2)	2.5 (-4.7 to 11.1)	-3.2 (-8.4 to 2.8)	-86.4 (-87.8 to -84.9)	-11.0 (-19.3 to -0.5)	-87.9 (-89.1 to -86.6)	-13.2 (-21.3 to -2.9)
Pakistan	-37.7 (-41.3 to -33.6)	-11.7 (-15.1 to -8.0)	23.7 (16.5 to 31.8)	-3.8 (-7.5 to 0.2)	-76.6 (-78.1 to -74.8)	-19.1 (-23.1 to -15.1)	-64.4 (-66.6 to -61.6)	-18.6 (-22.6 to -14.5)
Southeast Asia, east Asia, and Oceania	-34.8 (-38.5 to -31.6)	-7.6 (-9.9 to -5.4)	-17.1 (-21.8 to -13.1)	-5.3 (-7.7 to -3.0)	-92.6 (-93.0 to -92.2)	-39.1 (-40.8 to -37.3)	-94.2 (-94.5 to -93.9)	-37.1 (-38.8 to -35.2)
East Asia	-34.0 (-38.0 to -30.3)	-7.2 (-10.0 to -4.7)	-20.6 (-25.5 to -16.3)	-5.4 (-8.3 to -2.8)	-95.4 (-95.7 to -95.2)	-7.7 (-11.5 to -4.2)	-96.8 (-97.0 to -96.6)	0.9 (-3.2 to 4.8)
China	-34.3 (-38.4 to -30.6)	-7.3 (-10.1 to -4.6)	-21.0 (-26.0 to -16.6)	-5.5 (-8.3 to -2.7)	-95.4 (-95.7 to -95.2)	-7.3 (-11.3 to -3.5)	-96.8 (-97.0 to -96.6)	1.9 (-2.4 to 6.1)
North Korea	-27.9 (-37.3 to -14.2)	-5.9 (-16.6 to 7.7)	-10.1 (-21.9 to 6.9)	-4.8 (-15.6 to 9.0)	-94.6 (-95.3 to -93.7)	-18.4 (-28.8 to -6.2)	-96.8 (-97.3 to -96.3)	-23.3 (-33.1 to -11.9)

Taiwan (province of China)	-23.0 (-26.2 to -19.4)	-4.5 (-8.4 to -1.4)	-10.8 (-14.5 to -6.7)	-4.1 (-8.1 to -1.0)	-79.5 (-80.8 to -78.2)	-5.9 (-11.8 to 0.1)	-87.5 (-88.3 to -86.7)	-12.7 (-18.2 to -7.2)
Oceania	-36.5 (-40.4 to -27.0)	-6.5 (-10.0 to 2.0)	30.3 (22.4 to 49.8)	2.8 (-1.1 to 12.1)	-52.7 (-58.3 to -44.0)	8.8 (-7.2 to 45.2)	-11.0 (-21.5 to 5.4)	17.5 (0.2 to 56.7)
American Samoa	-4.9 (-15.0 to 6.8)	-4.2 (-14.1 to 7.8)	9.0 (-2.6 to 22.4)	-5.0 (-14.8 to 6.9)	-8.0 (-21.9 to 8.9)	-2.2 (-16.7 to 14.5)	-36.5 (-46.0 to -24.8)	-8.4 (-21.9 to 7.3)
Cook Islands	-34.7 (-45.0 to -24.4)	-13.0 (-21.7 to -1.4)	-38.3 (-47.9 to -28.5)	-13.2 (-21.9 to -1.6)	-77.3 (-80.6 to -72.5)	-18.0 (-28.7 to -7.1)	-87.1 (-89.0 to -84.4)	-24.4 (-34.2 to -14.4)
Fiji	-49.1 (-57.0 to -36.1)	-17.2 (-24.3 to -6.8)	-38.9 (-48.3 to -23.3)	-15.8 (-23.1 to -5.3)	-76.3 (-80.3 to -70.2)	-20.2 (-31.9 to -5.7)	-77.9 (-81.6 to -72.2)	-23.9 (-35.1 to -10.1)
Guam	-8.3 (-20.6 to 2.0)	-7.9 (-17.4 to 2.1)	14.4 (-0.9 to 27.3)	-6.2 (-15.9 to 3.9)	-12.6 (-27.3 to 1.0)	-8.0 (-20.0 to 5.0)	-14.3 (-28.7 to -0.9)	-8.9 (-20.8 to 4.0)
Kiribati	-28.0 (-39.2 to -10.5)	-11.7 (-21.3 to 3.8)	15.4 (-2.7 to 43.4)	-7.1 (-17.2 to 9.1)	-56.7 (-65.0 to -39.8)	-11.0 (-24.8 to 15.7)	-44.7 (-55.2 to -23.1)	-9.6 (-23.6 to 17.6)
Marshall Islands	-26.4 (-36.2 to -13.9)	-14.6 (-23.3 to -5.8)	-8.6 (-20.7 to 7.0)	-13.0 (-21.9 to -4.0)	-70.4 (-75.0 to -63.4)	-20.1 (-30.0 to -8.5)	-76.3 (-80.0 to -70.7)	-24.5 (-33.8 to -13.5)
Federated States of Micronesia	-37.1 (-43.3 to -28.3)	-8.5 (-16.6 to -0.0)	-38.4 (-44.5 to -29.8)	-9.7 (-17.6 to -1.3)	-30.1 (-38.3 to -16.1)	-4.9 (-15.7 to 10.0)	-59.3 (-64.1 to -51.1)	-11.4 (-21.4 to 2.5)
Nauru	-35.1 (-44.8 to -23.3)	-13.3 (-24.3 to -2.9)	-33.2 (-43.2 to -21.1)	-12.1 (-23.2 to -1.6)	-85.7 (-87.8 to -82.6)	-26.0 (-35.9 to -11.3)	-89.1 (-90.7 to -86.8)	-28.7 (-38.2 to -14.6)
Niue	-39.2 (-47.4 to -30.7)	-15.0 (-22.3 to -5.3)	-56.3 (-62.2 to -50.2)	-13.7 (-21.2 to -3.8)	-46.9 (-55.5 to -37.6)	-16.0 (-29.1 to -0.1)	-75.7 (-79.7 to -71.5)	-18.6 (-31.3 to -3.1)
Northern Mariana Islands	8.7 (-5.2 to 25.3)	-2.6 (-12.1 to 8.5)	1.8 (-11.3 to 17.3)	-10.7 (-19.4 to -0.5)	5.1 (-10.2 to 23.3)	-1.1 (-11.9 to 12.0)	-51.9 (-58.9 to -43.5)	-16.0 (-25.2 to -4.9)
Palau	-26.7 (-36.4 to -7.6)	-8.3 (-18.4 to 3.4)	-14.3 (-25.6 to 8.1)	-7.8 (-17.9 to 3.9)	-54.7 (-61.8 to -42.4)	-26.4 (-37.7 to -12.3)	-73.0 (-77.2 to -65.7)	-39.0 (-48.4 to -27.3)
Papua New Guinea	-38.2 (-41.8 to -28.0)	-5.7 (-9.6 to 3.8)	49.2 (40.6 to 73.9)	5.7 (1.4 to 16.4)	-55.7 (-61.2 to -47.2)	10.4 (-7.1 to 50.0)	0.2 (-12.3 to 19.4)	22.1 (2.7 to 65.8)
Samoa	-16.1 (-24.7 to -4.3)	-11.0 (-20.1 to -1.3)	8.4 (-2.7 to 23.6)	-5.3 (-14.9 to 5.1)	-28.5 (-38.8 to -14.2)	-14.5 (-24.2 to -0.1)	-26.0 (-36.6 to -11.2)	-10.8 (-21.0 to 4.2)
Solomon Islands	-48.2 (-55.6 to -32.1)	-7.5 (-14.8 to 7.5)	-0.3 (-14.5 to 30.8)	-0.6 (-8.5 to 15.5)	-69.9 (-74.3 to -59.9)	-14.7 (-27.0 to 23.6)	-52.8 (-59.7 to -37.0)	-14.0 (-26.4 to 24.6)
Tokelau	-9.3 (-19.6 to 1.0)	-7.7 (-19.8 to 3.8)	-24.1 (-32.8 to -15.5)	-1.3 (-14.3 to 11.0)	-15.1 (-26.0 to -1.4)	-8.4 (-23.0 to 13.9)	-50.0 (-56.4 to -41.9)	-7.5 (-22.3 to 15.0)
Tonga	-50.9 (-56.2 to -42.1)	-10.5 (-19.9 to 3.4)	-48.1 (-53.7 to -38.8)	-12.1 (-21.3 to 1.5)	-56.1 (-62.1 to -47.6)	-8.4 (-25.7 to 14.9)	-59.8 (-65.3 to -52.0)	-14.8 (-30.9 to 6.8)
Tuvalu	-27.8 (-37.7 to -17.1)	-11.4 (-20.9 to -1.4)	-8.9 (-21.4 to 4.7)	-7.7 (-17.5 to 2.8)	-63.8 (-69.6 to -56.7)	-17.8 (-29.2 to -3.5)	-70.9 (-75.5 to -65.1)	-17.4 (-28.8 to -3.0)
Vanuatu	-41.3 (-47.5 to -34.8)	-12.3 (-22.3 to -3.6)	14.2 (2.1 to 26.9)	-5.4 (-16.3 to 4.0)	-71.1 (-74.5 to -66.6)	-7.5 (-21.8 to 8.4)	-58.0 (-63.0 to -51.5)	-6.0 (-20.6 to 10.1)
Southeast Asia	-32.3 (-35.5 to -29.0)	-8.1 (-9.8 to -6.4)	-2.3 (-6.9 to 2.5)	-4.9 (-6.7 to -3.1)	-83.8 (-84.5 to -83.1)	-52.4 (-53.7 to -50.9)	-85.1 (-85.8 to -84.5)	-54.7 (-55.9 to -53.3)
Cambodia	-32.5 (-40.9 to -23.2)	-11.1 (-20.5 to 0.3)	8.0 (-5.5 to 22.9)	-5.6 (-15.7 to 6.4)	-91.2 (-92.3 to -90.0)	-22.1 (-31.8 to -8.9)	-91.8 (-92.8 to -90.7)	-21.6 (-31.4 to -8.3)
Indonesia	-23.1 (-26.7 to -19.5)	-4.1 (-7.0 to -1.5)	7.6 (2.6 to 12.7)	-1.3 (-4.3 to 1.4)	-76.8 (-77.7 to -76.1)	-27.9 (-30.3 to -25.7)	-79.9 (-80.6 to -79.2)	-33.8 (-36.1 to -31.8)

Laos	-26.2 (-33.7 to -18.4)	-8.1 (-16.0 to 0.9)	27.2 (14.3 to 40.7)	-3.1 (-11.4 to 6.4)	-76.7 (-79.9 to -72.8)	-14.1 (-24.5 to -0.6)	-73.1 (-76.8 to -68.6)	-12.8 (-23.5 to 0.9)
Malaysia	-37.3 (-42.9 to -31.9)	-9.0 (-15.1 to -3.0)	11.1 (1.2 to 20.8)	-4.7 (-11.1 to 1.5)	-74.8 (-78.5 to -71.2)	-16.7 (-27.3 to -4.5)	-72.9 (-77.0 to -69.1)	-14.3 (-25.2 to -1.7)
Maldives	-38.7 (-46.6 to -28.2)	0.9 (-9.0 to 11.3)	37.7 (19.9 to 61.3)	15.9 (4.5 to 27.8)	-86.6 (-88.3 to -84.5)	-17.4 (-29.9 to -6.5)	-86.6 (-88.4 to -84.6)	-18.7 (-31.0 to -8.0)
Mauritius	-28.7 (-37.7 to -20.2)	-5.9 (-16.6 to 5.7)	-17.3 (-27.7 to -7.4)	-5.8 (-16.5 to 5.9)	-81.9 (-84.7 to -78.6)	-10.6 (-27.1 to 6.7)	-88.9 (-90.6 to -86.9)	-15.6 (-31.2 to 0.8)
Myanmar	-25.0 (-31.9 to -17.1)	-6.5 (-15.7 to 1.8)	-0.2 (-9.5 to 10.2)	-3.3 (-12.9 to 5.2)	-71.8 (-75.3 to -67.7)	-19.9 (-30.8 to -7.0)	-75.6 (-78.6 to -72.0)	-22.2 (-32.8 to -9.7)
Philippines	-41.6 (-44.6 to -38.4)	-13.7 (-16.5 to -11.2)	3.5 (-1.8 to 9.1)	-8.0 (-10.9 to -5.3)	-86.8 (-87.3 to -86.4)	-72.7 (-73.4 to -72.1)	-82.3 (-82.9 to -81.7)	-72.2 (-72.9 to -71.5)
Seychelles	-24.3 (-36.2 to -11.9)	-1.3 (-11.2 to 8.3)	5.8 (-10.7 to 23.2)	2.4 (-8.0 to 12.3)	-87.1 (-89.5 to -84.6)	-18.3 (-29.4 to -7.0)	-88.3 (-90.4 to -85.9)	-19.9 (-30.8 to -8.7)
Sri Lanka	-35.2 (-42.7 to -26.7)	-7.9 (-14.7 to -1.7)	-17.8 (-27.3 to -7.0)	-5.7 (-12.6 to 0.8)	-95.2 (-95.8 to -94.5)	-9.6 (-18.7 to 0.9)	-95.9 (-96.4 to -95.3)	-17.8 (-26.1 to -8.3)
Thailand	-30.5 (-36.3 to -25.3)	-4.8 (-9.4 to 0.8)	-14.3 (-21.4 to -8.0)	-3.8 (-8.4 to 1.8)	-88.1 (-89.4 to -86.6)	-9.4 (-18.2 to 0.2)	-93.1 (-93.8 to -92.2)	-20.2 (-28.0 to -11.8)
Timor-Leste	-33.8 (-40.5 to -25.6)	-11.1 (-18.8 to -1.2)	12.9 (1.5 to 26.9)	-3.7 (-12.0 to 7.1)	-71.0 (-75.2 to -66.6)	-9.3 (-21.3 to 4.2)	-64.1 (-69.2 to -58.6)	-5.3 (-17.8 to 8.7)
Vietnam	-41.9 (-45.9 to -37.2)	-8.9 (-11.9 to -5.9)	-17.6 (-23.2 to -10.9)	-6.3 (-9.3 to -3.2)	-90.6 (-91.7 to -89.1)	-13.4 (-23.2 to -1.4)	-92.8 (-93.7 to -91.7)	-20.7 (-29.7 to -9.7)
Sub-Saharan Africa	-36.1 (-37.9 to -34.5)	-9.1 (-10.3 to -8.0)	40.4 (36.5 to 44.0)	0.7 (-0.5 to 2.0)	-65.0 (-66.3 to -63.8)	-9.6 (-11.7 to -7.4)	-35.2 (-37.7 to -33.0)	-5.3 (-7.5 to -3.0)
Central sub-Saharan Africa	-27.4 (-31.5 to -23.7)	-10.5 (-15.2 to -5.5)	72.0 (62.4 to 80.8)	-0.1 (-5.4 to 5.4)	-59.2 (-62.7 to -55.3)	-10.9 (-19.7 to -2.1)	-21.4 (-28.2 to -13.9)	-8.4 (-17.4 to 0.6)
Angola	-35.5 (-40.2 to -30.8)	-8.9 (-14.9 to -1.9)	88.3 (74.6 to 102.2)	4.3 (-2.6 to 12.3)	-54.8 (-61.1 to -48.7)	-9.1 (-21.0 to 4.8)	17.9 (1.4 to 33.7)	-3.9 (-16.5 to 10.8)
Central African Republic	-26.8 (-28.4 to -25.1)	-6.4 (-8.6 to -4.4)	41.4 (38.3 to 44.6)	2.2 (-0.1 to 4.4)	-58.0 (-61.3 to -54.6)	-17.0 (-23.4 to -10.6)	-30.1 (-35.4 to -24.3)	-12.0 (-18.7 to -5.1)
Congo (Brazzaville)	-43.2 (-48.7 to -36.9)	-9.0 (-17.1 to 0.2)	22.4 (10.5 to 36.0)	-0.4 (-9.2 to 9.6)	-75.3 (-78.6 to -71.9)	-7.4 (-19.9 to 6.8)	-59.9 (-65.4 to -54.4)	-10.3 (-22.4 to 3.4)
DR Congo	-23.7 (-30.3 to -15.7)	-13.7 (-20.4 to -6.5)	73.4 (58.3 to 91.5)	-4.2 (-11.6 to 3.8)	-71.3 (-75.0 to -66.7)	-17.3 (-28.2 to -5.9)	-48.5 (-55.0 to -40.1)	-15.7 (-26.8 to -4.1)
Equatorial Guinea	-42.8 (-47.3 to -37.7)	-8.4 (-14.0 to -2.3)	88.7 (73.8 to 105.5)	3.7 (-2.6 to 10.7)	-72.0 (-75.9 to -67.7)	-42.0 (-48.2 to -35.8)	-42.1 (-50.2 to -33.2)	-41.4 (-47.7 to -35.2)
Gabon	-46.8 (-50.5 to -42.5)	-10.9 (-15.4 to -5.7)	-6.1 (-12.6 to 1.5)	-4.9 (-9.8 to 0.6)	-84.2 (-86.2 to -81.9)	-17.5 (-27.7 to -7.2)	-81.0 (-83.4 to -78.2)	-17.4 (-27.7 to -7.2)
Eastern sub-Saharan Africa	-38.0 (-40.6 to -35.5)	-9.9 (-12.0 to -8.2)	34.2 (28.7 to 39.7)	-0.3 (-2.5 to 1.7)	-78.7 (-79.7 to -77.6)	-17.0 (-20.1 to -14.2)	-62.0 (-63.9 to -60.2)	-13.1 (-16.3 to -10.2)
Burundi	-46.2 (-48.6 to -43.6)	-14.3 (-16.0 to -12.6)	15.2 (10.2 to 20.9)	-0.7 (-2.6 to 1.3)	-92.6 (-93.5 to -91.5)	-4.4 (-15.5 to 7.3)	-85.9 (-87.6 to -83.8)	8.7 (-3.9 to 22.0)
Comoros	-28.2 (-35.7 to -19.2)	-8.2 (-16.6 to 1.8)	10.2 (-1.3 to 24.0)	-3.7 (-12.6 to 6.7)	-83.3 (-85.5 to -81.1)	-25.4 (-35.3 to -12.6)	-84.1 (-86.3 to -82.0)	-28.3 (-37.8 to -16.0)
Djibouti	-21.0 (-29.8 to -10.6)	-8.4 (-18.0 to 2.0)	95.4 (73.8 to 121.2)	2.0 (-8.7 to 13.7)	-59.8 (-65.9 to -51.0)	-11.8 (-25.2 to 6.2)	-29.6 (-40.4 to -14.3)	-12.2 (-25.5 to 5.8)

Eritrea	-45.1 (-51.1 to -37.5)	-15.7 (-24.6 to -6.7)	22.7 (9.4 to 39.7)	-8.3 (-18.0 to 1.6)	-91.7 (-92.8 to -90.6)	-19.0 (-27.7 to -8.3)	-86.2 (-88.0 to -84.4)	-17.5 (-26.4 to -6.7)
Ethiopia	-29.8 (-33.0 to -26.2)	-2.1 (-5.8 to 1.8)	46.9 (40.3 to 54.4)	8.0 (3.9 to 12.2)	-64.0 (-65.8 to -62.1)	-11.0 (-15.8 to -5.9)	-39.0 (-42.1 to -35.8)	-6.4 (-11.4 to -1.0)
Kenya	-25.5 (-29.2 to -21.4)	-7.4 (-9.4 to -5.6)	61.4 (53.4 to 70.1)	1.1 (-1.0 to 3.1)	-71.2 (-72.1 to -70.0)	12.0 (9.0 to 16.7)	-57.5 (-58.9 to -55.7)	11.9 (9.0 to 16.7)
Madagascar	-41.6 (-46.6 to -35.5)	-12.9 (-19.5 to -5.8)	30.5 (19.2 to 44.0)	-3.7 (-11.1 to 4.1)	-83.4 (-85.5 to -80.6)	-19.7 (-31.0 to -7.8)	-70.2 (-74.1 to -65.2)	-16.9 (-28.6 to -4.6)
Malawi	-31.9 (-39.0 to -24.5)	-13.4 (-22.0 to -3.6)	31.4 (17.7 to 45.7)	-3.3 (-13.0 to 7.6)	-81.1 (-83.8 to -78.1)	-0.0 (-13.4 to 20.7)	-72.9 (-76.8 to -68.7)	0.5 (-12.9 to 21.4)
Mozambique	-47.1 (-53.0 to -41.5)	-14.1 (-21.2 to -7.4)	19.6 (6.2 to 32.1)	-3.9 (-11.9 to 3.5)	-92.6 (-93.6 to -91.3)	-52.5 (-58.9 to -46.2)	-83.7 (-86.1 to -81.0)	-49.3 (-56.1 to -42.6)
Rwanda	-44.1 (-49.9 to -36.9)	-14.4 (-21.6 to -6.7)	-1.1 (-11.3 to 11.7)	-6.2 (-14.1 to 2.2)	-93.0 (-93.8 to -92.0)	-15.1 (-25.2 to 0.3)	-91.5 (-92.5 to -90.2)	-14.1 (-24.3 to 1.4)
Somalia	-15.2 (-18.9 to -11.2)	-6.1 (-10.0 to -2.0)	141.4 (130.8 to 152.7)	8.9 (4.3 to 13.7)	-48.3 (-52.2 to -43.9)	-15.2 (-21.5 to -7.9)	48.9 (37.8 to 61.4)	-3.3 (-10.5 to 4.9)
South Sudan	-19.8 (-27.0 to -12.5)	-8.5 (-17.8 to 1.2)	27.1 (15.7 to 38.7)	-7.4 (-16.9 to 2.3)	-56.3 (-61.7 to -49.3)	-15.5 (-27.0 to -2.6)	-34.2 (-42.4 to -23.8)	-17.9 (-29.0 to -5.4)
Uganda	-56.0 (-58.9 to -52.7)	-16.3 (-20.6 to -11.6)	4.4 (-2.5 to 12.3)	-7.2 (-12.0 to -2.0)	-89.0 (-90.3 to -87.5)	-22.7 (-32.5 to -12.6)	-78.3 (-80.8 to -75.4)	-19.1 (-29.4 to -8.6)
Tanzania	-50.0 (-52.3 to -48.1)	-16.1 (-18.0 to -14.2)	9.5 (4.6 to 13.7)	-6.9 (-9.0 to -4.7)	-90.7 (-91.9 to -89.3)	-23.0 (-33.0 to -12.7)	-82.2 (-84.6 to -79.5)	-19.0 (-29.5 to -8.2)
Zambia	-40.4 (-47.0 to -33.6)	-8.1 (-16.2 to 0.5)	36.9 (21.8 to 52.4)	3.9 (-5.2 to 13.7)	-88.9 (-90.2 to -87.4)	-28.8 (-37.1 to -18.8)	-79.4 (-81.8 to -76.6)	-25.1 (-33.8 to -14.5)
Southern sub-Saharan Africa	-46.8 (-49.2 to -44.4)	-5.5 (-7.9 to -3.2)	-20.3 (-24.0 to -16.8)	-1.1 (-3.6 to 1.3)	-76.2 (-77.7 to -74.5)	-1.6 (-7.5 to 4.3)	-73.1 (-74.9 to -71.2)	-3.4 (-9.2 to 2.4)
Botswana	-52.4 (-58.0 to -46.4)	-10.0 (-18.2 to -2.1)	-14.4 (-24.6 to -3.7)	-3.8 (-12.7 to 4.5)	-79.4 (-81.9 to -76.6)	16.7 (-0.9 to 32.3)	-75.0 (-78.0 to -71.6)	15.8 (-1.7 to 31.3)
eSwatini	-54.5 (-59.5 to -49.0)	-12.1 (-19.8 to -4.8)	-35.6 (-42.6 to -27.8)	-9.4 (-17.4 to -2.0)	-86.8 (-88.3 to -85.1)	-0.4 (-13.4 to 14.2)	-87.2 (-88.7 to -85.5)	-4.9 (-17.3 to 9.0)
Lesotho	-40.9 (-48.1 to -33.1)	-10.7 (-18.5 to -1.4)	-31.6 (-39.9 to -22.6)	-8.1 (-16.1 to 1.5)	-93.8 (-94.6 to -92.9)	-47.9 (-54.7 to -37.9)	-95.0 (-95.7 to -94.2)	-49.7 (-56.3 to -40.2)
Namibia	-45.0 (-46.0 to -43.8)	-13.5 (-14.3 to -12.3)	-6.2 (-7.9 to -4.2)	-8.4 (-9.3 to -7.2)	-92.6 (-93.2 to -92.1)	-23.0 (-28.8 to -16.1)	-90.1 (-90.8 to -89.4)	-21.2 (-27.1 to -14.2)
South Africa	-47.9 (-51.3 to -44.6)	-0.3 (-4.1 to 3.9)	-21.3 (-26.5 to -16.4)	3.7 (-0.3 to 8.1)	-70.3 (-72.8 to -67.2)	-0.6 (-7.3 to 6.7)	-67.0 (-69.7 to -63.5)	-3.2 (-9.7 to 3.9)
Zimbabwe	-43.2 (-45.3 to -40.8)	-12.2 (-14.5 to -10.0)	-17.5 (-20.6 to -14.0)	-6.4 (-8.8 to -4.0)	-81.8 (-83.7 to -79.2)	-1.4 (-11.8 to 11.1)	-78.2 (-80.5 to -75.2)	-1.7 (-12.0 to 10.7)
Western sub-Saharan Africa	-37.6 (-39.2 to -36.1)	-9.2 (-10.8 to -7.8)	47.8 (44.1 to 51.3)	1.5 (-0.3 to 3.1)	-60.8 (-62.8 to -59.2)	-8.4 (-11.5 to -5.4)	-20.1 (-24.1 to -16.8)	-2.8 (-6.1 to 0.4)
Benin	-45.7 (-52.1 to -39.1)	-12.4 (-21.7 to -1.5)	41.6 (25.0 to 59.1)	-0.2 (-10.9 to 12.2)	-77.1 (-80.9 to -73.3)	-15.6 (-30.8 to 0.2)	-48.5 (-57.0 to -39.8)	-7.8 (-24.4 to 9.6)
Burkina Faso	-26.4 (-33.5 to -18.2)	-13.1 (-21.4 to -4.3)	74.7 (57.8 to 94.3)	-1.1 (-10.6 to 8.8)	-86.8 (-88.4 to -84.4)	-6.8 (-24.3 to 8.3)	-71.3 (-74.9 to -66.1)	3.8 (-15.7 to 20.6)
Cape Verde	-37.7 (-45.5 to -28.7)	-9.2 (-19.8 to 1.0)	-0.2 (-12.6 to 14.3)	-5.5 (-16.5 to 5.1)	-92.0 (-93.2 to -90.5)	-16.9 (-28.6 to 0.8)	-92.6 (-93.7 to -91.2)	-21.0 (-32.2 to -4.2)

Cameroon	-43.0 (-48.2 to -39.1)	-10.0 (-14.5 to -4.1)	59.6 (45.1 to 70.6)	1.0 (-4.1 to 7.7)	-82.2 (-84.4 to -79.8)	-25.3 (-33.4 to -14.9)	-61.9 (-66.5 to -56.8)	-24.0 (-32.3 to -13.5)
Chad	-25.4 (-34.2 to -15.8)	-9.1 (-17.9 to 5.1)	103.2 (79.1 to 129.4)	5.5 (-4.8 to 21.8)	-46.8 (-57.7 to -36.3)	-10.3 (-22.6 to 9.1)	43.2 (13.8 to 71.5)	2.6 (-11.5 to 24.7)
Côte d'Ivoire	-51.5 (-53.1 to -49.7)	-14.9 (-16.0 to -13.6)	3.8 (0.4 to 7.8)	-7.4 (-8.6 to -6.0)	-88.6 (-89.5 to -87.0)	-30.9 (-36.5 to -20.9)	-80.3 (-81.9 to -77.6)	-29.0 (-34.8 to -18.7)
The Gambia	-59.3 (-60.9 to -57.6)	-2.1 (-3.6 to -0.5)	-7.9 (-11.5 to -3.9)	8.0 (6.3 to 9.8)	-63.4 (-68.5 to -57.5)	-8.5 (-19.1 to 2.7)	-38.1 (-46.7 to -28.2)	-6.9 (-17.7 to 4.5)
Ghana	-46.8 (-50.9 to -42.8)	-13.3 (-19.1 to -7.6)	11.6 (3.1 to 20.1)	-4.9 (-11.2 to 1.4)	-95.1 (-95.8 to -94.3)	-48.8 (-54.5 to -42.6)	-92.7 (-93.7 to -91.5)	-47.4 (-53.2 to -41.0)
Guinea	-31.1 (-38.8 to -20.8)	-11.5 (-21.3 to -2.3)	40.8 (25.1 to 61.9)	-1.6 (-12.6 to 8.5)	-55.1 (-61.6 to -47.9)	-10.2 (-22.3 to 5.3)	-21.0 (-32.4 to -8.3)	-4.2 (-17.1 to 12.3)
Guinea-Bissau	-35.3 (-41.3 to -24.7)	-9.3 (-18.2 to -0.1)	22.0 (10.9 to 42.1)	-1.2 (-10.9 to 8.8)	-72.5 (-76.5 to -67.3)	8.8 (-8.4 to 29.3)	-58.1 (-64.2 to -50.1)	10.4 (-7.1 to 31.1)
Liberia	-29.6 (-36.3 to -21.8)	-8.7 (-17.4 to 1.9)	71.6 (55.2 to 90.7)	-1.7 (-11.1 to 9.7)	-81.2 (-83.8 to -78.3)	-37.4 (-45.1 to -27.4)	-64.0 (-68.9 to -58.4)	-39.8 (-47.2 to -30.1)
Mali	-26.2 (-35.5 to -16.9)	-6.7 (-20.5 to 4.9)	86.5 (62.9 to 110.0)	8.0 (-8.1 to 21.3)	-56.0 (-69.0 to -46.3)	-9.5 (-34.5 to 9.0)	3.6 (-27.0 to 26.5)	3.4 (-25.2 to 24.5)
Mauritania	-42.4 (-43.5 to -41.2)	-7.9 (-9.1 to -6.8)	11.9 (9.7 to 14.3)	-0.4 (-1.7 to 0.8)	-77.6 (-79.8 to -75.4)	-22.8 (-30.6 to -14.4)	-68.8 (-71.8 to -65.6)	-24.1 (-31.8 to -15.8)
Niger	-41.1 (-43.1 to -38.7)	-13.8 (-16.1 to -11.4)	71.1 (65.4 to 77.9)	0.6 (-2.1 to 3.3)	-71.6 (-75.1 to -67.9)	-12.8 (-22.4 to -1.7)	-18.1 (-28.1 to -7.3)	0.2 (-10.8 to 13.0)
Nigeria	-35.1 (-36.7 to -33.5)	-6.6 (-8.5 to -4.6)	54.5 (50.7 to 58.3)	4.2 (2.0 to 6.3)	-44.2 (-46.3 to -41.8)	-5.2 (-8.5 to -1.8)	16.6 (12.3 to 21.6)	-0.5 (-4.0 to 3.0)
São Tomé and Príncipe	-45.6 (-51.9 to -38.7)	-11.0 (-19.7 to -3.0)	-8.0 (-18.6 to 3.5)	-4.9 (-14.2 to 3.5)	-91.1 (-92.5 to -89.4)	-14.0 (-29.9 to 0.9)	-90.0 (-91.6 to -88.1)	-19.3 (-34.2 to -5.3)
Senegal	-42.6 (-49.1 to -36.2)	-12.1 (-21.0 to -2.1)	13.9 (1.1 to 26.8)	-4.6 (-14.3 to 6.2)	-88.6 (-90.2 to -86.8)	-20.7 (-32.6 to -5.1)	-83.2 (-85.5 to -80.4)	-19.7 (-31.8 to -3.9)
Sierra Leone	-47.0 (-52.2 to -41.0)	-14.5 (-21.3 to -6.8)	20.3 (8.3 to 33.9)	-4.2 (-11.9 to 4.4)	-86.8 (-88.5 to -84.8)	-19.6 (-28.4 to -10.0)	-74.9 (-78.0 to -71.0)	-15.2 (-24.4 to -5.1)
Togo	-36.2 (-41.6 to -30.8)	-13.2 (-19.6 to -6.5)	38.0 (26.2 to 49.7)	-5.0 (-12.1 to 2.3)	-86.2 (-88.2 to -83.9)	-23.0 (-33.8 to -12.2)	-78.0 (-81.2 to -74.3)	-23.6 (-34.3 to -13.0)

Supplemental Table 3. Percentage change in HBV-related death rates and HBV-related death counts in all ages between 1990 and 2019 and 2015 and 2019, by location

	**Death rates, all ages**		**Death counts, all ages**	
**Location name**	**1990 to 2019**	**2015 to 2019**	**1990 to 2019**	**2015 to 2019**
Global	-26.8 (-34.7 to -17.6)	-1.4 (-9.9 to 6.6)	5.9 (-5.6 to 19.2)	2.9 (-5.9 to 11.3)
African Region	-37.2 (-48.8 to -23.4)	-7.8 (-16.5 to 2.1)	35.6 (10.5 to 65.4)	1.9 (-7.7 to 12.9)
Eastern Mediterranean Region	-34.7 (-49.5 to -18.7)	-0.6 (-12.3 to 12.0)	25.2 (-3.2 to 56.1)	7.2 (-5.4 to 20.8)
European Region	-6.0 (-12.6 to 1.0)	-3.5 (-8.9 to 2.2)	1.8 (-5.4 to 9.3)	-2.5 (-8.0 to 3.3)
Region of the Americas	-13.1 (-18.8 to -6.2)	2.8 (-2.1 to 8.0)	22.7 (14.6 to 32.3)	6.1 (1.1 to 11.5)
South-East Asia Region	-0.9 (-16.9 to 17.9)	-6.0 (-16.7 to 5.9)	52.7 (28.1 to 81.7)	-1.9 (-13.0 to 10.5)
Western Pacific Region	-37.5 (-48.8 to -22.8)	5.1 (-13.1 to 24.3)	-22.6 (-36.7 to -4.6)	7.5 (-11.2 to 27.1)
Low SDI	-34.0 (-44.6 to -22.3)	-8.0 (-14.7 to -0.7)	41.0 (18.4 to 66.2)	1.4 (-5.9 to 9.5)
Low-middle SDI	-14.9 (-25.4 to -1.2)	-6.0 (-14.2 to 3.2)	32.8 (16.5 to 54.3)	-1.3 (-9.9 to 8.4)
Middle SDI	-28.6 (-38.7 to -15.9)	1.1 (-11.0 to 13.9)	-0.4 (-14.4 to 17.4)	4.9 (-7.6 to 18.1)
High-middle SDI	-38.7 (-47.5 to -28.0)	3.1 (-9.3 to 16.5)	-23.8 (-34.7 to -10.5)	5.2 (-7.4 to 18.9)
High SDI	-9.5 (-16.5 to -2.2)	3.7 (-0.1 to 7.7)	11.5 (2.9 to 20.6)	6.1 (2.3 to 10.2)
Central Europe, eastern Europe, and central Asia	14.3 (4.4 to 25.6)	-3.7 (-10.8 to 3.8)	14.0 (4.2 to 25.3)	-3.2 (-10.3 to 4.4)
Central Asia	13.2 (-2.5 to 31.7)	-5.2 (-13.6 to 4.1)	52.9 (31.7 to 77.8)	-0.1 (-8.8 to 9.8)
Armenia	117.2 (74.5 to 169.0)	-4.1 (-19.6 to 12.3)	92.1 (54.3 to 137.9)	-5.2 (-20.5 to 11.0)
Azerbaijan	6.2 (-16.9 to 35.2)	-13.6 (-26.3 to 1.2)	48.9 (16.6 to 89.5)	-10.2 (-23.3 to 5.2)
Georgia	11.8 (-11.0 to 35.4)	0.9 (-15.9 to 18.7)	-25.6 (-40.8 to -9.9)	-2.6 (-18.8 to 14.7)
Kazakhstan	54.1 (26.0 to 91.2)	-7.6 (-20.6 to 6.4)	73.2 (41.5 to 114.8)	-2.4 (-16.1 to 12.4)
Kyrgyzstan	-15.1 (-30.7 to 4.4)	-5.9 (-18.1 to 8.2)	24.3 (1.4 to 52.9)	0.4 (-12.6 to 15.5)
Mongolia	21.7 (-10.5 to 63.0)	3.9 (-9.4 to 19.5)	91.5 (40.8 to 156.4)	11.9 (-2.4 to 28.7)
Tajikistan	-28.7 (-50.4 to -1.8)	0.5 (-17.9 to 22.1)	25.9 (-12.4 to 73.4)	9.6 (-10.5 to 33.1)
Turkmenistan	14.4 (-15.3 to 51.0)	-4.8 (-22.4 to 16.9)	56.9 (16.2 to 107.1)	-0.3 (-18.8 to 22.3)
Uzbekistan	2.1 (-19.0 to 30.5)	-6.3 (-22.3 to 11.5)	64.1 (30.2 to 109.7)	-0.5 (-17.4 to 18.5)

Central Europe	-28.1 (-38.1 to -18.0)	0.5 (-12.3 to 13.6)	-33.2 (-42.5 to -23.9)	-0.7 (-13.3 to 12.2)
Albania	-6.2 (-32.3 to 28.0)	9.9 (-12.1 to 36.4)	-22.9 (-44.3 to 5.2)	7.6 (-14.0 to 33.5)
Bosnia and Herzegovina	-3.7 (-28.1 to 24.8)	-0.4 (-20.7 to 25.5)	-30.0 (-47.7 to -9.3)	-6.0 (-25.2 to 18.3)
Bulgaria	-9.8 (-31.4 to 15.4)	3.6 (-18.6 to 30.5)	-28.0 (-45.2 to -7.8)	0.6 (-21.0 to 26.7)
Croatia	-50.4 (-62.8 to -35.8)	-3.5 (-23.8 to 21.5)	-57.0 (-67.8 to -44.3)	-4.9 (-24.9 to 19.7)
Czech Republic	-38.3 (-53.2 to -21.7)	-0.2 (-18.6 to 24.2)	-36.3 (-51.6 to -19.1)	0.4 (-18.1 to 24.9)
Hungary	-40.3 (-52.1 to -26.0)	-1.5 (-19.1 to 19.4)	-44.4 (-55.4 to -31.1)	-2.9 (-20.3 to 17.6)
Montenegro	10.8 (-14.8 to 42.9)	-24.3 (-37.9 to -9.5)	9.8 (-15.5 to 41.7)	-24.9 (-38.4 to -10.2)
North Macedonia	12.8 (-13.9 to 45.2)	-7.5 (-28.0 to 17.5)	20.5 (-8.0 to 55.1)	-7.0 (-27.5 to 18.2)
Poland	-31.4 (-44.9 to -16.0)	-0.0 (-17.7 to 20.3)	-30.9 (-44.5 to -15.4)	-0.1 (-17.8 to 20.2)
Romania	-8.1 (-26.1 to 13.3)	5.7 (-13.3 to 30.0)	-24.4 (-39.2 to -6.9)	2.5 (-15.9 to 26.1)
Serbia	-29.0 (-48.0 to -6.7)	-6.6 (-27.1 to 18.0)	-34.0 (-51.6 to -13.1)	-8.1 (-28.2 to 16.2)
Slovakia	-36.8 (-54.1 to -13.7)	-1.8 (-23.6 to 26.7)	-34.9 (-52.8 to -11.2)	-1.5 (-23.4 to 27.0)
Slovenia	-44.0 (-62.3 to -18.4)	3.0 (-20.1 to 36.0)	-41.0 (-60.4 to -14.2)	3.3 (-19.9 to 36.4)
Eastern Europe	74.3 (52.7 to 97.1)	-6.0 (-15.9 to 5.1)	61.6 (41.6 to 82.7)	-6.5 (-16.4 to 4.5)
Belarus	68.6 (26.7 to 121.1)	-4.8 (-27.1 to 22.9)	53.0 (15.0 to 100.6)	-5.5 (-27.6 to 22.0)
Estonia	35.1 (1.6 to 71.3)	-2.7 (-24.9 to 23.7)	13.0 (-15.0 to 43.3)	-2.9 (-25.1 to 23.5)
Latvia	6.6 (-14.5 to 33.3)	-2.6 (-19.9 to 19.8)	-23.2 (-38.4 to -4.0)	-6.1 (-22.9 to 15.5)
Lithuania	71.4 (37.3 to 115.0)	-12.2 (-29.2 to 7.9)	30.3 (4.4 to 63.5)	-15.8 (-32.1 to 3.5)
Moldova	-31.4 (-41.1 to -20.0)	-5.8 (-18.4 to 9.2)	-43.1 (-51.2 to -33.7)	-7.9 (-20.2 to 6.7)
Russia	71.4 (44.8 to 102.3)	-12.0 (-24.3 to 1.8)	66.5 (40.7 to 96.5)	-11.7 (-24.1 to 2.1)
Ukraine	131.4 (88.5 to 181.6)	8.5 (-9.2 to 30.4)	93.5 (57.6 to 135.5)	5.5 (-11.7 to 26.8)
High income	-9.6 (-17.1 to -1.1)	2.5 (-0.7 to 5.5)	7.8 (-1.1 to 17.9)	4.2 (1.0 to 7.3)
Australasia	13.3 (1.4 to 26.9)	-2.5 (-7.5 to 2.9)	62.3 (45.4 to 81.9)	2.4 (-2.8 to 8.0)
Australia	10.7 (-1.9 to 26.0)	-3.4 (-9.0 to 2.6)	61.3 (42.9 to 83.6)	1.9 (-3.9 to 8.2)
New Zealand	28.5 (17.0 to 40.8)	3.5 (-1.4 to 8.8)	69.1 (53.9 to 85.2)	5.8 (0.8 to 11.1)

High-income Asia Pacific	4.4<br>(-7.3 to 17.1)	8.1<br>(3.3 to 13.5)	12.6<br>(0.1 to 26.4)	8.3<br>(3.5 to 13.7)
Brunei	19.3<br>(-4.6 to 51.3)	8.3<br>(-3.0 to 20.2)	101.7<br>(61.3 to 155.9)	13.1<br>(1.3 to 25.5)
Japan	8.2<br>(-4.4 to 19.7)	8.7<br>(5.6 to 12.0)	9.8<br>(-2.9 to 21.5)	7.5<br>(4.4 to 10.7)
South Korea	-6.9<br>(-21.1 to 9.7)	5.3<br>(-2.5 to 14.1)	12.2<br>(-5.0 to 32.1)	8.6<br>(0.6 to 17.6)
Singapore	12.7<br>(0.2 to 26.0)	10.6<br>(3.2 to 18.2)	109.6<br>(86.5 to 134.3)	13.2<br>(5.7 to 21.0)
High-income North America	41.0<br>(27.7 to 55.8)	-4.3<br>(-10.6 to 1.7)	83.0<br>(65.7 to 102.2)	-2.0<br>(-8.5 to 4.2)
Canada	68.6<br>(49.0 to 90.4)	-4.3<br>(-10.6 to 2.5)	125.9<br>(99.6 to 155.1)	-1.1<br>(-7.6 to 5.9)
Greenland	80.5<br>(32.6 to 145.7)	1.8<br>(-10.7 to 16.0)	82.5<br>(34.1 to 148.4)	1.7<br>(-10.8 to 15.8)
USA	38.9<br>(25.2 to 53.7)	-4.3<br>(-11.1 to 2.3)	79.6<br>(61.9 to 98.8)	-2.1<br>(-9.0 to 4.7)
Southern Latin America	-30.8<br>(-37.2 to -23.5)	-1.1<br>(-6.3 to 4.0)	-6.8<br>(-15.4 to 3.1)	2.7<br>(-2.7 to 8.0)
Argentina	-27.1<br>(-34.5 to -19.3)	-2.5<br>(-8.9 to 3.6)	-0.7<br>(-10.8 to 9.9)	1.2<br>(-5.5 to 7.6)
Chile	-36.0<br>(-43.7 to -27.1)	0.7<br>(-5.4 to 6.9)	-12.3<br>(-22.9 to -0.1)	5.2<br>(-1.3 to 11.6)
Uruguay	-37.9<br>(-45.6 to -27.7)	2.4<br>(-4.2 to 9.3)	-32.0<br>(-40.5 to -20.8)	3.3<br>(-3.4 to 10.3)
Western Europe	-25.8<br>(-32.8 to -17.6)	-0.9<br>(-3.6 to 2.1)	-15.8<br>(-23.8 to -6.5)	0.3<br>(-2.4 to 3.4)
Andorra	4.8<br>(-30.5 to 54.4)	3.3<br>(-9.6 to 17.9)	60.9<br>(6.8 to 137.0)	10.3<br>(-3.6 to 25.8)
Austria	-46.7<br>(-53.5 to -37.6)	-6.4<br>(-12.5 to 1.0)	-38.9<br>(-46.7 to -28.4)	-3.6<br>(-9.9 to 4.0)
Belgium	-20.1<br>(-30.8 to -8.2)	-6.8<br>(-12.6 to -0.8)	-8.6<br>(-20.9 to 5.1)	-5.2<br>(-11.1 to 0.9)
Cyprus	-35.9<br>(-50.1 to -12.9)	1.8<br>(-7.9 to 12.4)	8.2<br>(-15.7 to 47.0)	8.5<br>(-1.9 to 19.7)
Denmark	22.8<br>(5.5 to 42.5)	-0.4<br>(-6.9 to 7.1)	38.5<br>(19.0 to 60.8)	1.9<br>(-4.8 to 9.5)
Finland	67.5<br>(45.8 to 91.6)	-4.0<br>(-11.2 to 3.5)	85.1<br>(61.1 to 111.6)	-3.3<br>(-10.5 to 4.2)
France	-24.8<br>(-35.1 to -10.9)	-7.5<br>(-14.1 to -0.1)	-13.8<br>(-25.6 to 2.1)	-6.3<br>(-12.9 to 1.2)
Germany	-35.7<br>(-45.0 to -24.3)	-1.4<br>(-7.6 to 4.9)	-31.7<br>(-41.6 to -19.6)	1.0<br>(-5.3 to 7.4)
Greece	15.7<br>(-3.0 to 38.7)	3.0<br>(-2.7 to 9.5)	15.1<br>(-3.5 to 38.0)	0.6<br>(-5.1 to 6.9)
Iceland	29.4<br>(11.3 to 50.7)	6.1<br>(-2.6 to 15.3)	75.7<br>(51.2 to 104.7)	10.9<br>(1.8 to 20.5)
Ireland	56.7<br>(36.4 to 80.1)	-21.4<br>(-26.9 to -15.3)	113.6<br>(85.9 to 145.6)	-19.8<br>(-25.4 to -13.6)
Israel	-30.8<br>(-39.9 to -18.9)	1.8<br>(-4.5 to 8.7)	29.8<br>(12.8 to 52.1)	9.7<br>(3.0 to 17.2)

Italy	-43.5 (-47.6 to -39.3)	5.1 (0.6 to 9.2)	-40.0 (-44.4 to -35.5)	4.3 (-0.2 to 8.4)
Luxembourg	-53.3 (-61.3 to -42.9)	-7.1 (-15.7 to 3.4)	-24.3 (-37.3 to -7.4)	2.0 (-7.5 to 13.6)
Malta	-13.4 (-29.1 to 5.7)	4.5 (-4.0 to 13.9)	2.6 (-16.0 to 25.3)	6.0 (-2.6 to 15.5)
Monaco	6.0 (-23.2 to 46.0)	-1.7 (-13.5 to 10.1)	30.8 (-5.1 to 80.2)	0.1 (-11.9 to 12.2)
Netherlands	27.5 (11.7 to 48.1)	3.9 (-2.0 to 10.4)	46.5 (28.4 to 70.3)	5.4 (-0.7 to 12.0)
Norway	-25.5 (-32.8 to -16.7)	-4.1 (-8.3 to 0.4)	-6.2 (-15.3 to 4.9)	-0.8 (-5.0 to 3.9)
Portugal	-44.7 (-54.7 to -30.3)	4.0 (-2.5 to 12.3)	-41.8 (-52.4 to -26.7)	3.1 (-3.3 to 11.3)
San Marino	-13.8 (-45.8 to 30.2)	7.0 (-10.1 to 26.2)	21.1 (-23.8 to 82.9)	9.5 (-8.0 to 29.2)
Spain	-45.6 (-54.6 to -33.9)	1.7 (-4.7 to 8.7)	-35.5 (-46.1 to -21.6)	0.1 (-6.2 to 7.0)
Sweden	7.8 (-1.0 to 16.6)	-2.0 (-7.4 to 3.4)	28.3 (17.9 to 38.7)	1.6 (-4.1 to 7.2)
Switzerland	28.3 (11.5 to 48.7)	-1.6 (-8.2 to 4.8)	63.9 (42.6 to 90.1)	2.5 (-4.4 to 9.1)
UK	110.1 (99.5 to 126.9)	1.5 (-0.7 to 3.6)	145.7 (133.4 to 165.4)	3.7 (1.5 to 5.9)
Latin America and Caribbean	-25.7 (-31.9 to -18.7)	6.4 (-0.4 to 13.9)	11.3 (2.0 to 21.6)	10.4 (3.3 to 18.1)
Andean Latin America	-23.4 (-41.1 to -1.0)	-1.3 (-16.9 to 16.3)	27.7 (-1.9 to 64.9)	5.3 (-11.3 to 24.1)
Bolivia	-17.3 (-41.1 to 21.0)	3.6 (-9.2 to 20.5)	54.8 (10.2 to 126.3)	11.4 (-2.4 to 29.6)
Ecuador	0.0 (-24.1 to 28.3)	2.0 (-21.4 to 32.9)	75.5 (33.2 to 125.1)	10.3 (-15.1 to 43.6)
Peru	-33.6 (-54.3 to -4.2)	-5.2 (-28.4 to 23.8)	3.8 (-28.4 to 49.8)	0.4 (-24.2 to 31.0)
Caribbean	-23.0 (-35.5 to -9.1)	8.2 (-3.6 to 23.3)	2.9 (-13.8 to 21.6)	11.5 (-0.7 to 27.0)
Antigua and Barbuda	-56.7 (-63.9 to -47.9)	10.1 (-2.1 to 23.0)	-36.9 (-47.5 to -24.1)	11.2 (-1.1 to 24.2)
The Bahamas	-40.4 (-52.4 to -23.8)	11.3 (-3.4 to 29.3)	-12.4 (-30.1 to 12.0)	13.8 (-1.2 to 32.2)
Barbados	-21.5 (-37.7 to -0.8)	13.5 (-1.7 to 30.3)	-8.0 (-27.0 to 16.3)	15.7 (0.2 to 32.9)
Belize	-16.6 (-31.9 to 2.3)	9.3 (-4.0 to 24.8)	84.0 (50.3 to 125.7)	19.5 (4.9 to 36.4)
Bermuda	-38.5 (-49.8 to -23.7)	7.4 (-3.3 to 19.7)	-33.8 (-45.9 to -17.8)	5.9 (-4.6 to 18.0)
Cuba	-28.1 (-43.3 to -8.6)	6.8 (-14.1 to 30.3)	-24.6 (-40.5 to -4.2)	5.9 (-14.8 to 29.3)
Dominica	-50.5 (-61.0 to -37.2)	-25.0 (-34.5 to -14.6)	-54.1 (-63.8 to -41.7)	-25.6 (-35.0 to -15.2)
Dominican Republic	3.8 (-29.4 to 46.3)	16.4 (-10.1 to 51.6)	56.8 (6.6 to 121.0)	21.8 (-5.9 to 58.7)

Grenada	-54.4<br>(-61.2 to -46.5)	7.9<br>(-3.6 to 19.5)	-45.0<br>(-53.3 to -35.5)	6.6<br>(-4.8 to 18.0)
Guyana	-34.3<br>(-52.5 to -12.2)	-1.2<br>(-20.2 to 20.8)	-34.2<br>(-52.5 to -12.1)	3.5<br>(-16.4 to 26.4)
Haiti	-26.3<br>(-47.6 to 3.4)	1.7<br>(-9.4 to 14.1)	43.8<br>(2.2 to 101.8)	10.4<br>(-1.6 to 23.9)
Jamaica	-27.9<br>(-45.1 to -7.3)	4.6<br>(-14.8 to 26.9)	-14.3<br>(-34.7 to 10.3)	5.4<br>(-14.1 to 27.9)
Puerto Rico	-44.4<br>(-58.1 to -27.2)	8.8<br>(-16.5 to 39.7)	-45.8<br>(-59.1 to -29.1)	3.4<br>(-20.7 to 32.7)
Saint Kitts and Nevis	-61.5<br>(-70.7 to -50.9)	29.4<br>(13.3 to 46.4)	-44.6<br>(-57.9 to -29.3)	34.1<br>(17.4 to 51.7)
Saint Lucia	-37.4<br>(-48.1 to -23.7)	13.5<br>(0.6 to 26.4)	-20.4<br>(-34.1 to -3.0)	14.7<br>(1.7 to 27.8)
Saint Vincent and the Grenadines	-26.4<br>(-38.9 to -10.0)	8.1<br>(-3.5 to 21.5)	-24.3<br>(-37.2 to -7.5)	8.7<br>(-3.1 to 22.1)
Suriname	-43.6<br>(-55.3 to -29.5)	2.8<br>(-12.8 to 20.6)	-15.9<br>(-33.4 to 5.0)	5.0<br>(-10.9 to 23.2)
Trinidad and Tobago	-42.9<br>(-58.2 to -23.1)	5.5<br>(-22.7 to 43.7)	-34.2<br>(-51.8 to -11.3)	6.5<br>(-22.0 to 45.0)
Virgin Islands	-0.6<br>(-26.1 to 30.2)	4.1<br>(-6.6 to 15.2)	-2.5<br>(-27.5 to 27.7)	2.1<br>(-8.4 to 13.0)
Central Latin America	-3.9<br>(-17.6 to 10.9)	17.3<br>(2.9 to 33.2)	46.4<br>(25.5 to 68.9)	21.0<br>(6.2 to 37.4)
Colombia	4.8<br>(-21.4 to 38.7)	6.5<br>(-17.6 to 36.8)	53.9<br>(15.4 to 103.6)	10.9<br>(-14.2 to 42.5)
Costa Rica	12.0<br>(-15.8 to 45.9)	11.5<br>(-13.5 to 42.9)	73.8<br>(30.6 to 126.5)	14.8<br>(-11.0 to 47.2)
El Salvador	-28.3<br>(-47.2 to -4.5)	2.9<br>(-21.2 to 33.7)	-14.8<br>(-37.2 to 13.4)	4.9<br>(-19.7 to 36.3)
Guatemala	-39.8<br>(-54.3 to -20.4)	-10.0<br>(-30.2 to 15.2)	34.2<br>(2.0 to 77.6)	-2.1<br>(-24.1 to 25.2)
Honduras	7.3<br>(-18.7 to 46.6)	-0.1<br>(-13.4 to 16.1)	123.7<br>(69.5 to 205.7)	9.1<br>(-5.4 to 26.8)
Mexico	0.9<br>(-15.6 to 20.5)	25.5<br>(5.2 to 47.8)	47.5<br>(23.3 to 76.1)	30.1<br>(9.1 to 53.3)
Nicaragua	10.6<br>(-13.3 to 38.5)	3.4<br>(-16.4 to 26.4)	85.2<br>(45.3 to 131.9)	9.2<br>(-11.8 to 33.4)
Panama	-14.3<br>(-35.4 to 13.2)	0.5<br>(-22.8 to 30.6)	49.2<br>(12.5 to 97.2)	9.3<br>(-16.1 to 42.0)
Venezuela	-21.5<br>(-42.1 to 4.4)	30.9<br>(0.5 to 69.9)	17.0<br>(-13.7 to 55.6)	23.1<br>(-5.5 to 59.7)
Tropical Latin America	-35.2<br>(-39.3 to -30.5)	2.1<br>(-2.3 to 7.0)	-5.2<br>(-11.2 to 1.7)	5.8<br>(1.3 to 10.9)
Brazil	-35.5<br>(-39.6 to -30.7)	2.1<br>(-2.5 to 6.9)	-6.1<br>(-12.0 to 0.9)	5.7<br>(1.0 to 10.7)
Paraguay	-5.6<br>(-33.6 to 30.1)	5.7<br>(-21.5 to 36.8)	61.7<br>(13.8 to 122.9)	11.4<br>(-17.3 to 44.2)
North Africa and Middle East	-39.4<br>(-55.1 to -22.0)	-0.7<br>(-12.4 to 12.7)	6.9<br>(-20.7 to 37.5)	5.6<br>(-6.9 to 19.9)
Afghanistan	-67.9<br>(-80.1 to -51.3)	-13.3<br>(-24.1 to -2.2)	7.6<br>(-33.2 to 63.1)	-1.2<br>(-13.5 to 11.5)

Algeria	-35.9<br>(-54.1 to -10.6)	-0.9<br>(-14.0 to 14.2)	6.1<br>(-24.1 to 47.9)	5.4<br>(-8.6 to 21.4)
Bahrain	-27.2<br>(-45.3 to -3.7)	26.3<br>(9.3 to 45.9)	106.8<br>(55.3 to 173.3)	30.2<br>(12.6 to 50.4)
Egypt	-38.5<br>(-59.3 to -16.7)	-0.5<br>(-21.8 to 24.1)	9.3<br>(-27.6 to 48.1)	6.5<br>(-16.3 to 32.8)
Iran	-13.2<br>(-26.2 to 5.5)	4.9<br>(-0.9 to 11.7)	25.1<br>(6.2 to 51.9)	9.2<br>(3.1 to 16.2)
Iraq	-25.2<br>(-47.5 to 2.7)	-0.9<br>(-11.2 to 10.9)	79.1<br>(25.8 to 145.8)	7.1<br>(-4.1 to 19.8)
Jordan	-46.1<br>(-61.7 to -27.6)	13.2<br>(-2.8 to 30.4)	66.2<br>(18.1 to 123.3)	35.7<br>(16.5 to 56.3)
Kuwait	-18.9<br>(-33.8 to 1.2)	12.4<br>(-6.6 to 34.1)	104.1<br>(66.5 to 154.6)	30.8<br>(8.7 to 56.0)
Lebanon	-24.0<br>(-48.4 to 14.7)	-0.0<br>(-11.2 to 11.4)	20.1<br>(-18.4 to 81.3)	3.4<br>(-8.2 to 15.1)
Libya	-20.1<br>(-48.5 to 25.2)	14.5<br>(-1.7 to 30.5)	27.1<br>(-18.1 to 99.1)	18.4<br>(1.7 to 35.0)
Morocco	-28.3<br>(-50.8 to 2.8)	3.9<br>(-8.5 to 19.2)	1.9<br>(-30.1 to 46.2)	6.8<br>(-5.9 to 22.5)
Oman	-42.5<br>(-58.5 to -18.8)	-9.2<br>(-21.5 to 4.6)	35.5<br>(-2.1 to 91.5)	0.9<br>(-12.7 to 16.2)
Palestine	-43.6<br>(-59.8 to -21.7)	6.9<br>(-6.0 to 20.3)	35.1<br>(-3.9 to 87.4)	14.5<br>(0.7 to 28.8)
Qatar	-37.5<br>(-58.8 to -7.2)	10.7<br>(-7.9 to 31.4)	302.1<br>(165.3 to 497.2)	29.5<br>(7.7 to 53.7)
Saudi Arabia	-53.0<br>(-69.6 to -27.1)	6.3<br>(-6.5 to 20.5)	4.7<br>(-32.2 to 62.2)	17.0<br>(2.9 to 32.7)
Sudan	-65.8<br>(-82.9 to -33.9)	-11.0<br>(-21.3 to 2.0)	-30.8<br>(-65.6 to 33.6)	-2.8<br>(-14.1 to 11.4)
Syria	-2.9<br>(-34.7 to 45.7)	12.6<br>(-5.0 to 32.9)	9.1<br>(-26.6 to 63.8)	7.5<br>(-9.3 to 26.9)
Tunisia	-23.5<br>(-54.0 to 26.9)	2.6<br>(-13.3 to 23.7)	4.8<br>(-36.9 to 73.9)	5.4<br>(-10.9 to 27.1)
Turkey	-32.1<br>(-50.7 to -2.8)	-3.9<br>(-20.4 to 15.3)	-7.6<br>(-32.9 to 32.3)	-1.5<br>(-18.4 to 18.3)
United Arab Emirates	23.6<br>(-27.0 to 114.1)	35.2<br>(12.3 to 60.8)	510.0<br>(260.3 to 957.0)	38.0<br>(14.7 to 64.2)
Yemen	-60.2<br>(-79.5 to -23.9)	3.4<br>(-10.3 to 18.9)	-8.7<br>(-53.0 to 74.7)	12.9<br>(-2.1 to 29.8)
South Asia	-0.2<br>(-18.3 to 21.9)	-8.5<br>(-20.4 to 5.4)	64.1<br>(34.4 to 100.5)	-3.6<br>(-16.1 to 11.0)
Bangladesh	-48.0<br>(-61.9 to -29.4)	-3.2<br>(-17.6 to 11.1)	-24.0<br>(-44.4 to 3.1)	0.7<br>(-14.3 to 15.7)
Bhutan	-12.9<br>(-41.6 to 35.0)	7.5<br>(-5.5 to 21.2)	7.3<br>(-28.1 to 66.3)	6.3<br>(-6.6 to 19.8)
India	12.6<br>(-10.9 to 40.5)	-10.3<br>(-24.5 to 5.6)	83.1<br>(44.8 to 128.4)	-5.8<br>(-20.7 to 10.9)
Nepal	-42.6<br>(-60.5 to -22.1)	17.5<br>(0.1 to 37.0)	-10.7<br>(-38.5 to 21.2)	22.3<br>(4.2 to 42.5)
Pakistan	-15.5<br>(-37.6 to 22.9)	-0.3<br>(-20.4 to 26.1)	67.8<br>(23.9 to 144.0)	8.6<br>(-13.3 to 37.4)

Southeast Asia, east Asia, and Oceania	-35.6<br>(-46.3 to -22.3)	4.7<br>(-11.8 to 22.4)	-18.1<br>(-31.8 to -1.3)	7.3<br>(-9.6 to 25.5)
East Asia	-40.2<br>(-52.7 to -23.9)	5.0<br>(-16.5 to 28.5)	-28.1<br>(-43.2 to -8.5)	7.0<br>(-14.9 to 31.0)
China	-41.0<br>(-53.8 to -24.2)	4.9<br>(-17.3 to 29.5)	-29.1<br>(-44.5 to -8.9)	7.0<br>(-15.7 to 32.1)
North Korea	-11.7<br>(-37.0 to 20.5)	0.0<br>(-11.2 to 11.9)	10.0<br>(-21.5 to 50.1)	1.3<br>(-10.1 to 13.2)
Taiwan (province of China)	-14.9<br>(-34.5 to 10.6)	14.9<br>(-9.6 to 47.1)	-1.5<br>(-24.1 to 28.1)	15.4<br>(-9.2 to 47.8)
Oceania	-25.0<br>(-41.5 to -3.8)	-1.4<br>(-8.9 to 6.9)	53.9<br>(20.0 to 97.4)	8.4<br>(0.1 to 17.5)
American Samoa	55.0<br>(20.7 to 94.3)	6.8<br>(-3.5 to 16.9)	77.6<br>(38.3 to 122.7)	5.9<br>(-4.3 to 15.9)
Cook Islands	16.1<br>(-10.5 to 49.5)	7.8<br>(-4.7 to 21.1)	9.7<br>(-15.4 to 41.3)	7.6<br>(-4.9 to 20.8)
Fiji	13.6<br>(-19.2 to 58.9)	2.1<br>(-15.0 to 22.6)	36.3<br>(-3.0 to 90.7)	3.7<br>(-13.6 to 24.6)
Guam	67.0<br>(32.1 to 105.7)	4.0<br>(-7.7 to 17.1)	108.3<br>(64.8 to 156.6)	5.9<br>(-6.1 to 19.2)
Kiribati	-38.1<br>(-59.6 to -11.0)	-2.5<br>(-11.3 to 6.9)	-0.9<br>(-35.2 to 42.5)	2.5<br>(-6.7 to 12.4)
Marshall Islands	1.9<br>(-29.3 to 35.9)	1.4<br>(-11.0 to 15.0)	26.7<br>(-12.2 to 69.0)	3.2<br>(-9.3 to 17.1)
Federated States of Micronesia	-4.5<br>(-40.4 to 39.1)	8.3<br>(-9.4 to 23.4)	-6.5<br>(-41.6 to 36.2)	7.0<br>(-10.5 to 21.9)
Nauru	-30.4<br>(-50.9 to -6.6)	-5.6<br>(-16.6 to 8.2)	-28.4<br>(-49.5 to -3.9)	-4.3<br>(-15.5 to 9.8)
Niue	-16.6<br>(-39.1 to 10.8)	1.0<br>(-7.6 to 10.9)	-40.1<br>(-56.2 to -20.4)	2.6<br>(-6.2 to 12.6)
Northern Mariana Islands	70.5<br>(26.3 to 134.1)	13.9<br>(2.9 to 27.3)	59.6<br>(18.2 to 119.1)	4.4<br>(-5.6 to 16.8)
Palau	36.1<br>(-3.2 to 87.2)	15.0<br>(0.2 to 30.9)	59.1<br>(13.2 to 118.9)	15.6<br>(0.8 to 31.6)
Papua New Guinea	-24.8<br>(-48.1 to 8.6)	0.9<br>(-9.8 to 13.8)	81.6<br>(25.2 to 162.0)	13.2<br>(1.1 to 27.6)
Samoa	-8.8<br>(-33.9 to 28.1)	-1.5<br>(-12.0 to 9.9)	17.8<br>(-14.6 to 65.4)	4.9<br>(-6.3 to 17.1)
Solomon Islands	-25.8<br>(-49.4 to 5.1)	-4.9<br>(-15.1 to 6.0)	42.9<br>(-2.5 to 102.4)	2.2<br>(-8.8 to 13.9)
Tokelau	-11.7<br>(-37.1 to 19.0)	0.6<br>(-9.6 to 11.5)	-26.2<br>(-47.4 to -0.5)	7.6<br>(-3.3 to 19.1)
Tonga	-8.4<br>(-32.3 to 24.2)	1.7<br>(-8.6 to 12.5)	-3.1<br>(-28.4 to 31.4)	-0.2<br>(-10.3 to 10.4)
Tuvalu	-29.9<br>(-48.6 to -2.7)	-2.0<br>(-12.8 to 11.5)	-11.5<br>(-35.2 to 22.8)	2.2<br>(-9.1 to 16.2)
Vanuatu	-17.8<br>(-46.3 to 25.8)	-2.4<br>(-13.5 to 10.3)	59.9<br>(4.5 to 144.7)	5.2<br>(-6.7 to 18.9)
Southeast Asia	-10.5<br>(-22.8 to 5.1)	4.4<br>(-6.3 to 16.7)	29.2<br>(11.4 to 51.7)	8.0<br>(-3.0 to 20.8)
Cambodia	-18.2<br>(-37.6 to 8.6)	0.2<br>(-10.8 to 12.1)	30.9<br>(-0.1 to 73.8)	6.3<br>(-5.4 to 19.0)

Indonesia	-11.7 (-30.5 to 12.9)	5.4 (-13.6 to 28.0)	23.5 (-2.8 to 58.1)	8.4 (-11.1 to 31.8)
Laos	-42.8 (-61.8 to -13.2)	-5.2 (-16.2 to 7.7)	-1.4 (-34.2 to 49.6)	-0.0 (-11.6 to 13.6)
Malaysia	33.9 (2.9 to 72.6)	7.4 (-12.1 to 30.9)	137.4 (82.5 to 205.9)	12.4 (-8.1 to 37.0)
Maldives	-43.8 (-60.2 to -16.0)	8.2 (-6.6 to 24.8)	26.1 (-10.5 to 88.6)	24.4 (7.3 to 43.3)
Mauritius	-38.5 (-52.0 to -23.5)	4.4 (-15.9 to 29.8)	-28.7 (-44.3 to -11.2)	4.5 (-15.8 to 29.9)
Myanmar	-18.4 (-43.9 to 18.1)	-2.5 (-13.6 to 10.5)	8.6 (-25.3 to 57.1)	0.8 (-10.7 to 14.2)
Philippines	-31.1 (-49.5 to -3.2)	-0.4 (-22.4 to 25.8)	22.0 (-10.5 to 71.5)	6.2 (-17.2 to 34.1)
Seychelles	-12.6 (-29.0 to 9.0)	12.7 (-0.3 to 27.2)	22.2 (-0.7 to 52.5)	16.9 (3.4 to 31.9)
Sri Lanka	-35.1 (-53.3 to -12.2)	-1.2 (-27.2 to 33.0)	-17.6 (-40.7 to 11.4)	1.3 (-25.4 to 36.3)
Thailand	53.8 (8.1 to 107.8)	9.5 (-19.8 to 44.7)	89.6 (33.3 to 156.2)	10.7 (-18.9 to 46.2)
Timor-Leste	-12.9 (-45.3 to 22.8)	-17.9 (-29.4 to -2.8)	48.5 (-6.8 to 109.3)	-11.0 (-23.5 to 5.3)
Vietnam	-28.0 (-52.4 to 6.3)	8.7 (-3.7 to 21.6)	2.1 (-32.5 to 50.8)	11.9 (-0.9 to 25.1)
Sub-Saharan Africa	-37.4 (-48.7 to -23.9)	-8.0 (-16.6 to 1.9)	37.6 (12.7 to 67.1)	1.9 (-7.6 to 12.9)
Central sub-Saharan Africa	-43.8 (-60.7 to -23.5)	-18.5 (-27.6 to -8.8)	33.1 (-6.9 to 81.2)	-9.0 (-19.2 to 1.8)
Angola	-48.8 (-67.0 to -21.4)	-15.7 (-27.0 to -3.4)	49.5 (-3.6 to 129.7)	-3.5 (-16.4 to 10.6)
Central African Republic	-37.1 (-59.4 to -11.3)	-16.3 (-27.3 to -3.9)	21.5 (-21.5 to 71.3)	-8.6 (-20.5 to 4.9)
Congo (Brazzaville)	-39.7 (-60.2 to -10.9)	-18.3 (-29.7 to -5.3)	29.9 (-14.2 to 92.0)	-10.6 (-23.1 to 3.6)
DR Congo	-42.9 (-62.1 to -19.6)	-20.1 (-31.0 to -8.2)	29.7 (-13.8 to 82.7)	-11.3 (-23.4 to 1.9)
Equatorial Guinea	-62.7 (-77.3 to -39.9)	5.3 (-11.1 to 24.4)	23.2 (-25.3 to 98.4)	19.2 (0.6 to 40.8)
Gabon	-40.3 (-61.8 to -14.6)	-9.9 (-21.7 to 2.8)	5.3 (-32.6 to 50.7)	-3.8 (-16.4 to 9.7)
Eastern sub-Saharan Africa	-34.8 (-47.7 to -17.2)	-5.5 (-12.5 to 1.6)	41.2 (13.2 to 79.2)	4.6 (-3.1 to 12.5)
Burundi	-53.7 (-70.0 to -28.9)	-11.8 (-22.2 to -0.1)	-0.9 (-35.8 to 52.2)	2.3 (-9.8 to 15.8)
Comoros	-9.2 (-38.8 to 84.8)	-3.0 (-22.4 to 11.0)	39.2 (-6.2 to 183.5)	1.7 (-18.7 to 16.4)
Djibouti	6.4 (-31.8 to 64.8)	-17.7 (-31.4 to -2.5)	163.3 (68.8 to 307.9)	-8.3 (-23.6 to 8.6)
Eritrea	-23.9 (-47.4 to 13.5)	1.8 (-11.2 to 16.9)	70.1 (17.7 to 153.7)	10.8 (-3.3 to 27.2)
Ethiopia	-32.7 (-57.4 to 24.2)	-9.4 (-24.2 to 5.6)	40.9 (-10.8 to 160.1)	-0.2 (-16.4 to 16.4)

Kenya	-9.8<br>(-29.7 to 21.0)	0.7<br>(-16.4 to 20.8)	95.3<br>(52.3 to 162.1)	10.0<br>(-8.7 to 32.0)
Madagascar	-48.5<br>(-64.9 to -27.7)	0.5<br>(-12.9 to 16.0)	15.1<br>(-21.5 to 61.4)	11.0<br>(-3.8 to 28.1)
Malawi	-56.6<br>(-69.4 to -39.9)	-1.8<br>(-14.1 to 10.5)	-16.2<br>(-41.0 to 16.0)	9.6<br>(-4.2 to 23.3)
Mozambique	-46.5<br>(-68.7 to -8.9)	-6.8<br>(-19.4 to 7.0)	20.8<br>(-29.3 to 105.9)	4.1<br>(-9.8 to 19.6)
Rwanda	-54.6<br>(-68.8 to -32.4)	-1.0<br>(-12.7 to 12.4)	-19.6<br>(-44.9 to 19.5)	8.4<br>(-4.4 to 23.1)
Somalia	-21.7<br>(-44.2 to 11.6)	-6.0<br>(-18.7 to 7.4)	122.7<br>(58.7 to 217.7)	9.0<br>(-5.8 to 24.5)
South Sudan	-4.5<br>(-34.3 to 40.0)	1.8<br>(-11.3 to 18.1)	51.3<br>(4.0 to 121.8)	3.0<br>(-10.3 to 19.4)
Uganda	-38.5<br>(-57.0 to -12.8)	-2.0<br>(-14.4 to 11.3)	46.1<br>(2.1 to 107.1)	8.6<br>(-5.1 to 23.3)
Tanzania	-46.4<br>(-61.7 to -26.9)	-5.1<br>(-16.8 to 8.2)	17.3<br>(-16.2 to 60.0)	5.4<br>(-7.6 to 20.2)
Zambia	-39.4<br>(-58.4 to -14.2)	-12.7<br>(-25.1 to -0.1)	39.2<br>(-4.6 to 97.0)	-1.2<br>(-15.2 to 13.0)
Southern sub-Saharan Africa	-25.4<br>(-45.1 to 2.0)	-3.9<br>(-10.3 to 2.0)	11.6<br>(-17.8 to 52.7)	0.5<br>(-6.1 to 6.7)
Botswana	-36.3<br>(-62.0 to 8.1)	2.9<br>(-12.8 to 20.7)	14.5<br>(-31.7 to 94.2)	9.9<br>(-6.9 to 28.9)
eSwatini	35.4<br>(-48.1 to 157.4)	-3.3<br>(-16.5 to 12.9)	91.7<br>(-26.5 to 264.4)	-0.4<br>(-13.9 to 16.4)
Lesotho	3.9<br>(-53.3 to 115.0)	-4.2<br>(-17.0 to 11.2)	20.3<br>(-46.0 to 148.9)	-1.4<br>(-14.5 to 14.5)
Namibia	-32.5<br>(-54.0 to -0.3)	-27.5<br>(-38.3 to -15.5)	15.1<br>(-21.6 to 70.0)	-23.2<br>(-34.7 to -10.5)
South Africa	-26.7<br>(-46.1 to -2.4)	-2.4<br>(-10.3 to 5.8)	10.6<br>(-18.7 to 47.4)	1.5<br>(-6.7 to 10.0)
Zimbabwe	-26.1<br>(-54.5 to 13.8)	-5.7<br>(-17.5 to 6.1)	7.3<br>(-33.9 to 65.2)	0.5<br>(-12.0 to 13.1)
Western sub-Saharan Africa	-41.1<br>(-54.4 to -23.7)	-7.9<br>(-21.1 to 6.9)	39.4<br>(8.1 to 80.8)	3.0<br>(-11.7 to 19.5)
Benin	-47.0<br>(-62.8 to -25.3)	-9.8<br>(-20.1 to 1.6)	38.4<br>(-2.9 to 95.1)	2.7<br>(-9.1 to 15.6)
Burkina Faso	-54.6<br>(-72.5 to -33.3)	-15.8<br>(-26.0 to -4.5)	7.8<br>(-34.8 to 58.3)	-4.2<br>(-15.8 to 8.6)
Cape Verde	24.0<br>(-18.3 to 75.0)	10.7<br>(-1.9 to 23.8)	98.8<br>(31.0 to 180.5)	15.3<br>(2.1 to 28.9)
Cameroon	-46.6<br>(-69.2 to -13.0)	-6.5<br>(-19.8 to 7.9)	49.4<br>(-13.8 to 143.6)	4.9<br>(-10.0 to 21.1)
Chad	-31.3<br>(-49.7 to -6.0)	-10.3<br>(-21.7 to 1.3)	87.2<br>(37.1 to 155.9)	4.0<br>(-9.3 to 17.5)
Côte d'Ivoire	-31.6<br>(-53.6 to -4.0)	-5.7<br>(-18.3 to 8.4)	46.4<br>(-0.7 to 105.4)	2.6<br>(-11.1 to 17.9)
The Gambia	4.9<br>(-31.8 to 60.9)	11.4<br>(-4.9 to 32.1)	137.5<br>(54.4 to 264.3)	22.9<br>(4.9 to 45.7)
Ghana	-36.5<br>(-56.9 to -8.0)	-2.4<br>(-14.0 to 10.7)	33.3<br>(-9.5 to 93.1)	7.1<br>(-5.6 to 21.5)

Guinea	-29.0<br>(-49.4 to -1.5)	-15.1<br>(-25.0 to -3.7)	45.2<br>(3.4 to 101.3)	-5.6<br>(-16.6 to 7.0)
Guinea-Bissau	-34.4<br>(-53.5 to -4.2)	-14.8<br>(-26.0 to -3.1)	23.8<br>(-12.3 to 80.9)	-7.2<br>(-19.4 to 5.6)
Liberia	-50.5<br>(-65.9 to -31.0)	-11.7<br>(-24.9 to 2.6)	20.8<br>(-16.9 to 68.3)	-4.9<br>(-19.2 to 10.4)
Mali	-53.6<br>(-69.8 to -33.5)	-0.5<br>(-11.0 to 12.7)	17.2<br>(-23.7 to 68.2)	15.1<br>(3.0 to 30.4)
Mauritania	-52.4<br>(-66.9 to -35.4)	-7.9<br>(-27.1 to 7.2)	-7.6<br>(-35.6 to 25.6)	-0.4<br>(-21.2 to 15.9)
Niger	-37.8<br>(-55.4 to -13.7)	-12.3<br>(-24.2 to 0.4)	80.6<br>(29.4 to 150.6)	2.4<br>(-11.5 to 17.2)
Nigeria	-41.2<br>(-58.9 to -15.7)	-7.9<br>(-32.1 to 24.9)	40.0<br>(-2.0 to 100.7)	2.7<br>(-24.3 to 39.3)
São Tomé and Príncipe	-41.4<br>(-65.2 to -6.9)	0.1<br>(-10.7 to 12.4)	-0.9<br>(-41.2 to 57.3)	6.9<br>(-4.6 to 20.0)
Senegal	-30.3<br>(-51.7 to -3.5)	-1.9<br>(-19.9 to 12.3)	38.4<br>(-4.1 to 91.6)	6.5<br>(-13.1 to 21.8)
Sierra Leone	-55.3<br>(-72.0 to -32.1)	-12.0<br>(-24.0 to 2.3)	1.5<br>(-36.5 to 54.0)	-1.5<br>(-14.9 to 14.6)
Togo	-25.2<br>(-50.6 to 12.3)	-9.6<br>(-20.9 to 2.9)	61.7<br>(6.8 to 142.8)	-1.1<br>(-13.5 to 12.6)

Supplemental Table 4. Achievement in 2019 of WHO-GHSS 2020 death impact target, WHO-GHSS new cases impact target, and under-5 prevalence proxy target, with high probability, by location. High probability is defined by at least 950 out of 1000 draws meeting target.

WHO region	WHO-GHSS 2020 death impact target	WHO-GHSS 2020 new cases impact target	Under-5 prevalence proxy target
African Region	Namibia	Equatorial Guinea, Mozambique, Lesotho, Ghana, Liberia	Algeria, Equatorial Guinea, Burundi, Comoros, Eritrea, Kenya, Mauritius, Rwanda, Seychelles, Tanzania, Lesotho, Namibia, Cape Verde, The Gambia, Ghana
Eastern Mediterranean Region	-	Iraq, Syria	Bahrain, Egypt, Iran, Iraq, Jordan, Kuwait, Lebanon, Libya, Morocco, Oman, Qatar, Saudi Arabia, Syria, Tunisia, United Arab Emirates, Pakistan
European Region	Montenegro, Ireland	Ukraine, Sweden, Switzerland, UK	Armenia, Azerbaijan, Georgia, Kazakhstan, Kyrgyzstan, Tajikistan, Turkmenistan, Uzbekistan, Albania, Bosnia and Herzegovina, Bulgaria, Croatia, Czech Republic, Hungary, North Macedonia, Montenegro, Poland, Romania, Serbia, Slovakia, Slovenia, Belarus, Estonia, Latvia, Lithuania, Moldova, Russia, Ukraine, Andorra, Austria, Belgium, Cyprus, Denmark, Finland, France, Germany, Greece, Iceland, Ireland, Israel, Italy, Luxembourg, Malta, Netherlands, Norway, Portugal, Spain, Sweden, Switzerland, UK, Turkey, Monaco, San Marino
Region of the Americas	Dominica	Panama	Argentina, Chile, Uruguay, Canada, USA, Antigua and Barbuda, The Bahamas, Barbados, Belize, Cuba, Dominica, Dominican Republic, Grenada, Guyana, Haiti, Jamaica, Saint Lucia, Saint Vincent and the Grenadines, Suriname, Trinidad and Tobago, Bolivia, Ecuador, Peru, Colombia, Costa Rica, El Salvador, Guatemala, Honduras, Mexico, Nicaragua, Panama, Venezuela, Brazil, Paraguay, Saint Kitts and Nevis
South-East Asia Region	-	Indonesia	North Korea, Indonesia, Maldives, Myanmar, Sri Lanka, Thailand, Bangladesh, Bhutan, India, Nepal
Western Pacific Region	-	Philippines, Japan	Bahrain, Egypt, Iran, Iraq, Jordan, Kuwait, Lebanon, Libya, Morocco, Oman, Qatar, Saudi Arabia, Syria, Tunisia, United Arab Emirates, Pakistan

Supplementary Table 5. Achievement in 2019 of WHO Interim Guidance's absolute mortality rate target and WHO-GHSS under-5 prevalence target for 2030, with high probability, by location. High probability is defined by at least 950 out of 1000 draws.

WHO region	All age mortality of less than or equal to 4 per 100,000	Under 5 prevalence of less than or equal to 0.1%
Western Pacific Region	Papua New Guinea, Australia, New Zealand	Australia, New Zealand
South-East Asia Region	Maldives	Sri Lanka
European Region	Austria, Belgium, Cyprus, Denmark, France, Germany, Iceland, Ireland, Israel, Italy, Luxembourg, Malta, Netherlands, Norway, Portugal, Spain, Sweden, Switzerland, UK, San Marino	Albania, Czech Republic, North Macedonia, Poland, Serbia, Slovakia, Slovenia, Belarus, Estonia, Latvia, Lithuania, Russia, Andorra, Austria, Belgium, Cyprus, France, Germany, Greece, Ireland, Israel, Italy, Malta, Netherlands, Norway, Portugal, Spain, Sweden, UK, Monaco
Region of the Americas	Argentina, Uruguay, Canada, USA, Antigua and Barbuda, The Bahamas, Barbados, Belize, Cuba, Dominica, Grenada, Jamaica, Saint Lucia, Saint Vincent and the Grenadines, Suriname, Trinidad and Tobago, Ecuador, Peru, Colombia, Costa Rica, El Salvador, Guatemala, Honduras, Mexico, Nicaragua, Panama, Venezuela, Brazil, Paraguay, Saint Kitts and Nevis	Argentina, Chile, Uruguay, USA, Antigua and Barbuda, The Bahamas, Barbados, Belize, Cuba, Dominica, Grenada, Guyana, Jamaica, Saint Lucia, Saint Vincent and the Grenadines, Suriname, Trinidad and Tobago, Bolivia, Ecuador, Peru, Costa Rica, El Salvador, Mexico, Nicaragua, Saint Kitts and Nevis
African Region	Algeria, Mozambique, Rwanda, Tanzania, Uganda	-
Eastern Mediterranean Region	Bahrain, Iran, Iraq, Jordan, Kuwait, Oman, Qatar, Saudi Arabia, Tunisia	Kuwait

Supplementary Table 6. Prevalence of HBsAg, all ages, both sexes, as reported by GBD and multiple other estimation groups

	**% (95% uncertainty interval or confidence interval, as reported by original authors)**				
**Location Name**	**IHME, 2019**	**Schweitzer, ~1965-2013**	**WHO, 2016**	**WHO, 2019**	**CDA, 2016**
**Global**	4·09 (3·66–4·53)	3.61 (3.61 to 3.61)	3.5 (2.7 to 5.0%)	-	3.9 (3.4 to 4.6)
**African Region**	6·49 (5·76–7·30)	8.83 (8.82 - 8.83)	6.1 (4.6 to 8.5)	7.5 (5.7-10.5)	7.2 (6.2 to 8.2)
**Eastern Mediterranean Region**	3·07 (2·80–3·36)	3.01 (3.01 to 3.01)	3.3 (2.6 to 4.3)	2.5 (2.0-3.3)	2.2 (1.9 to 3.0)
**European Region**	1·15 (1·05–1·24)	2.06 (2.06 to 2.06)	1.6 (1.2 to 2.6)	1.5 (1.1-2.4)	1.6 (1.1 to 2.1)
**Region of the Americas**	1·22 (1·07–1·37)	0.81 (0.81 - 0.81)	0.7 (0.4 to 1.6)	0.5 (0.3-1.2)	0.4 (0.3 to 0.6)
**South-East Asia Region**	3·06 (2·71–3·41)	1.90 (1.90 - 1.90)	2.0 (1.5 to 4.0)	3.0 (2.3-6.0)	3.5 (2.9 to 4.0)
**Western Pacific Region**	7·04 (6·30–7·82)	5.26 (5.26 - 5.26)	6.2 (5.1 to 7.6)	5.9 (4.9-7.3)	5.7 (5.1 to 6.6)

Supplemental Figure 4. Hepatitis-related deaths by virus and cause, global, 2019

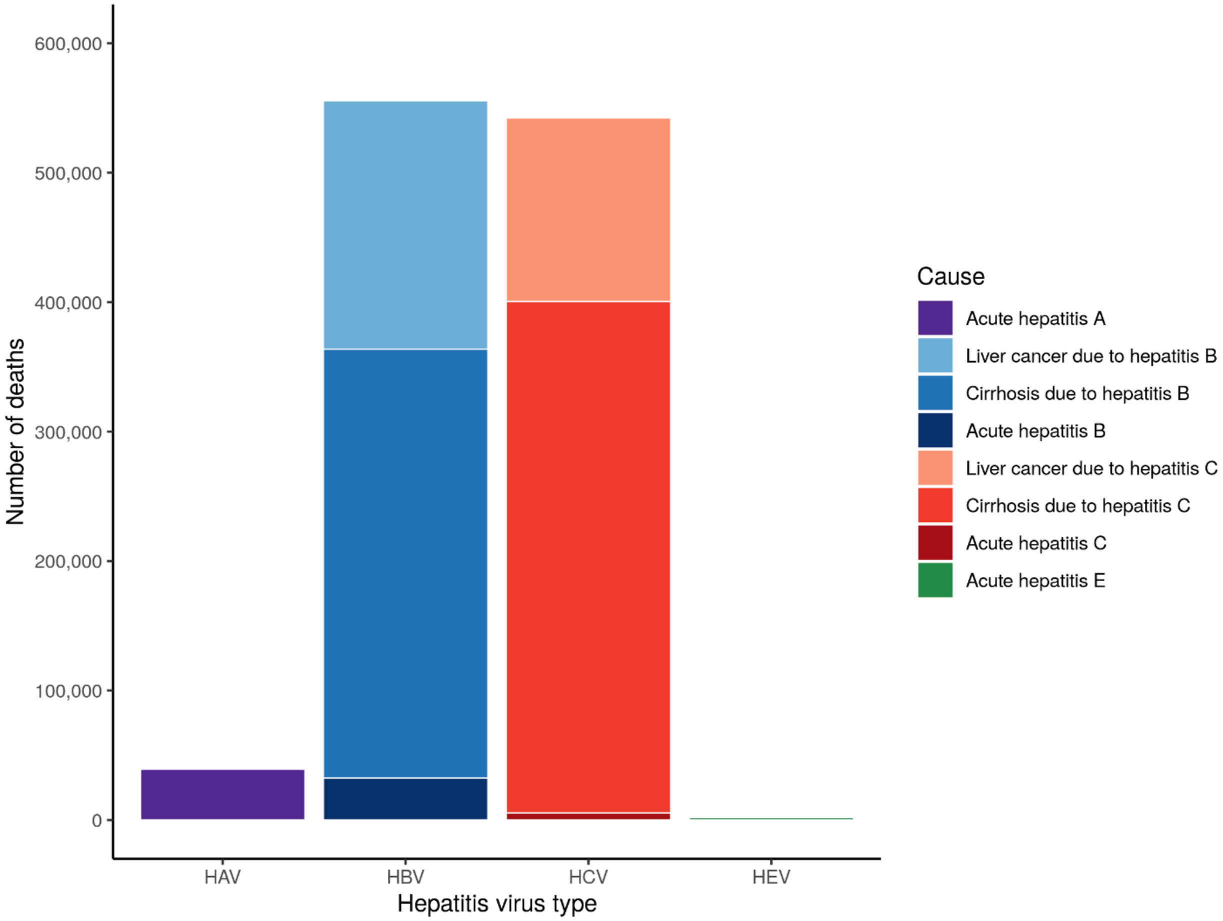

Supplemental Figure 5. Percentage of HBV-related disability-adjusted life-years (DALYs) in top burden countries (out of total HBV-related DALYs)

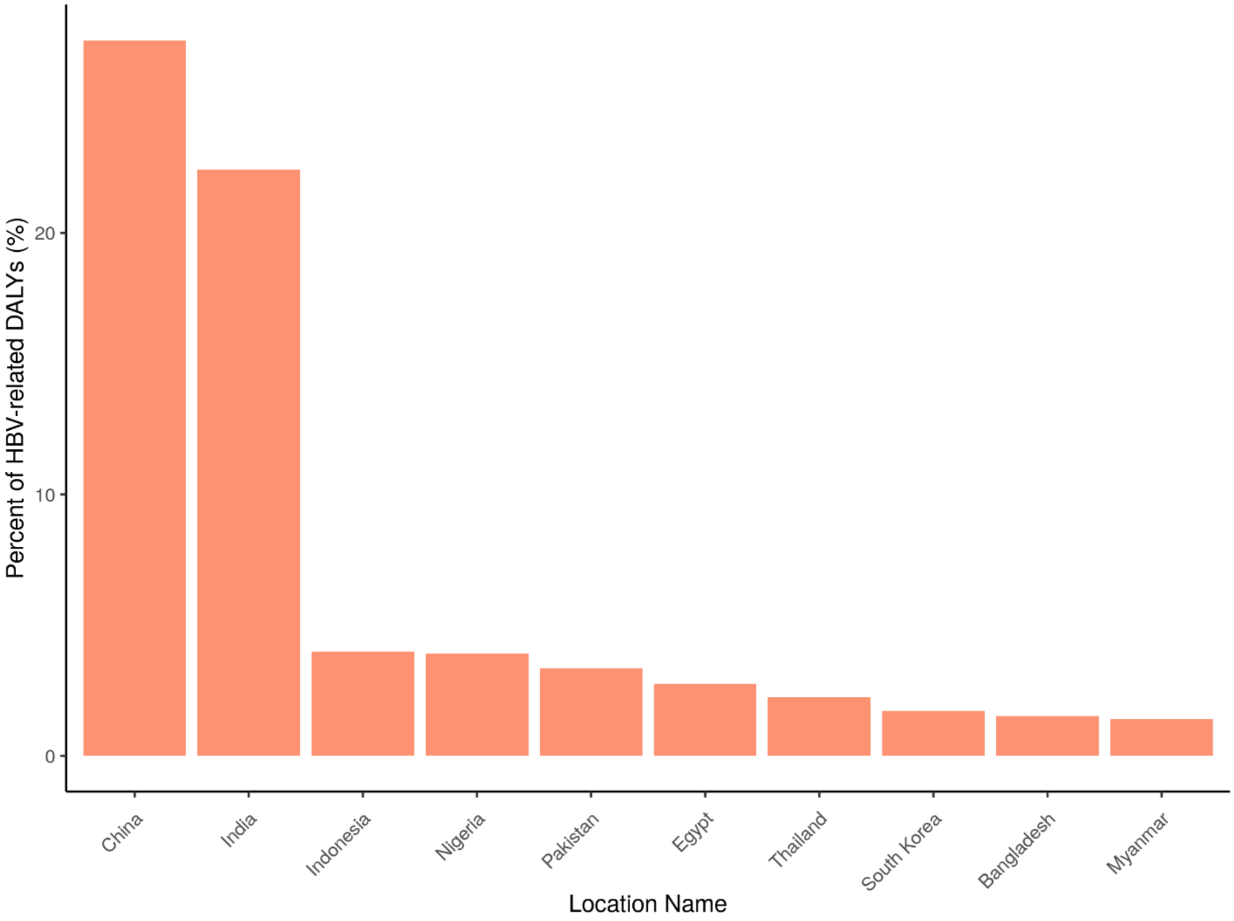

Supplemental Figure 6. Map of percentage change in all-age HBV-related death counts (A) and all-age HBV death rates (B) between 2015 and 2019 by country. Percentage change category was achieved with high probability (at least 950 out of 1000 draws of percentage change).

A

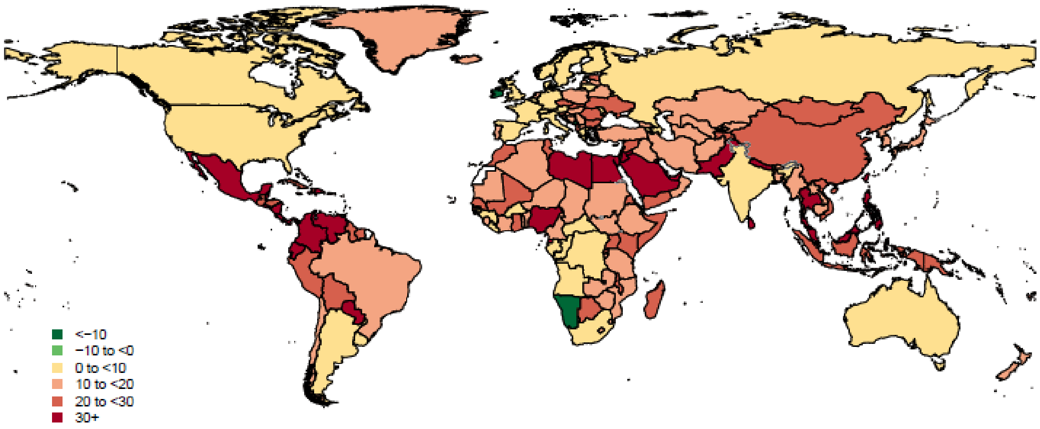

B

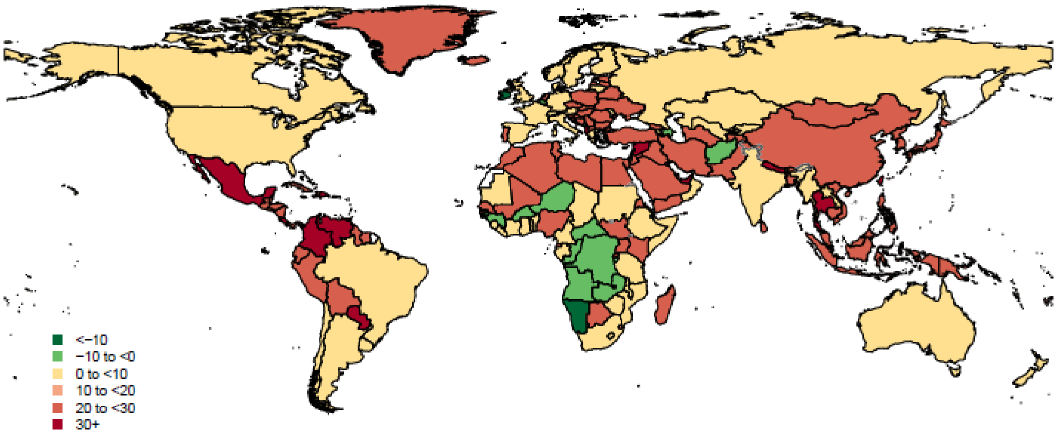

Supplemental Figure 4. Map of percentage change in under-5 HBsAg prevalence counts (A) and under-5 HBsAg seroprevalence (%) (B) between 2015 and 2019, by country. Percentage change category was achieved with high probability (at least 950 out of 1000 draws of percentage change).

A

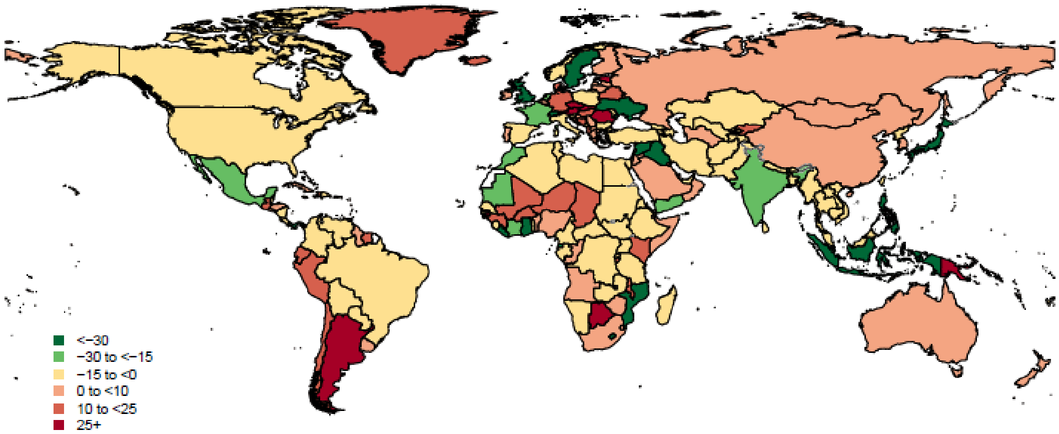

B

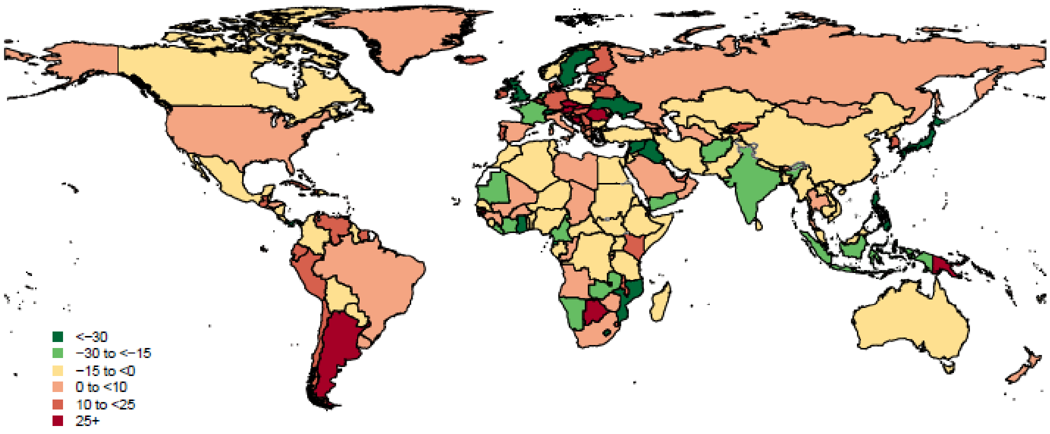

Supplemental Figure 5. HBV-related death counts by WHO region by cause

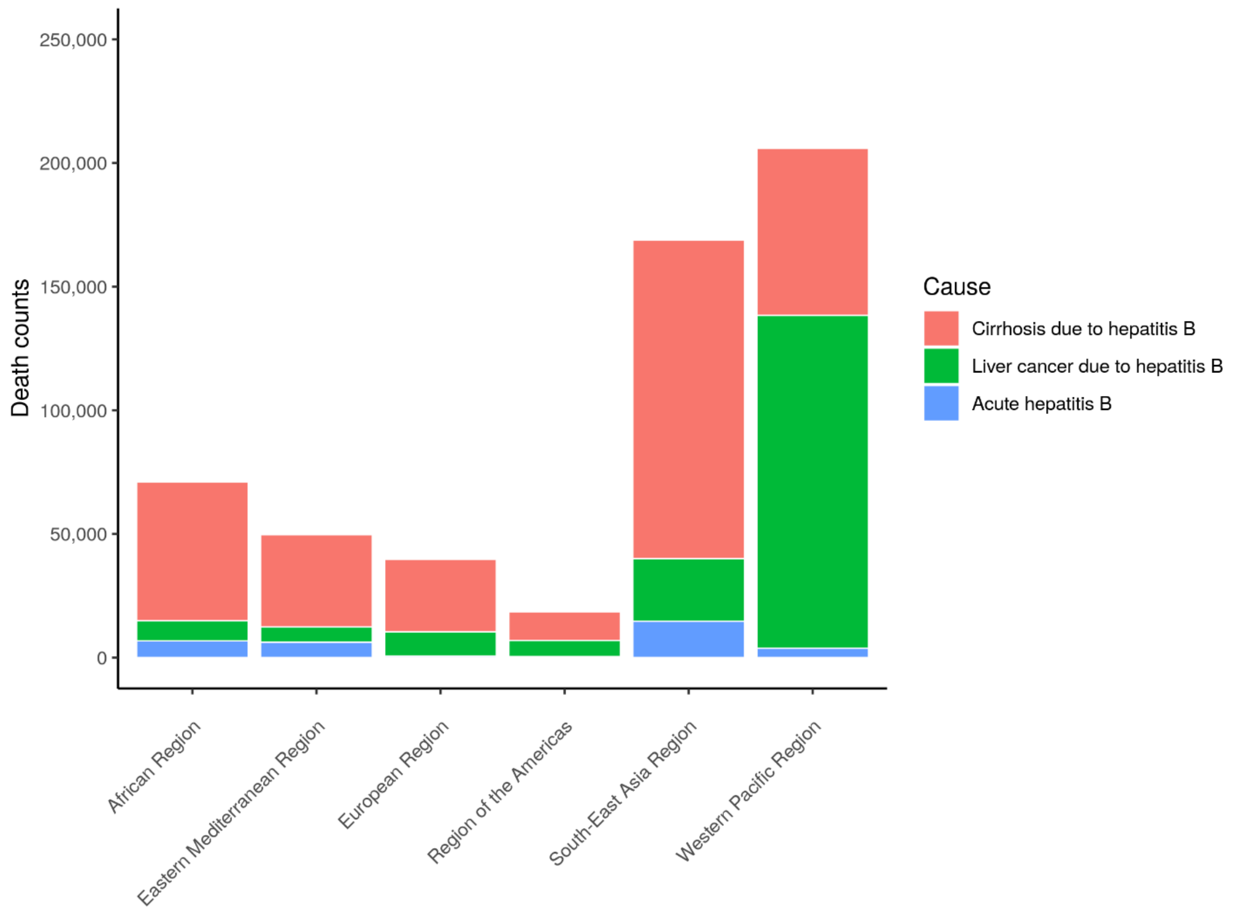

Supplemental Figure 6. Age-sex-specific death counts of HBV-related diseases in 2019, globally and by WHO region

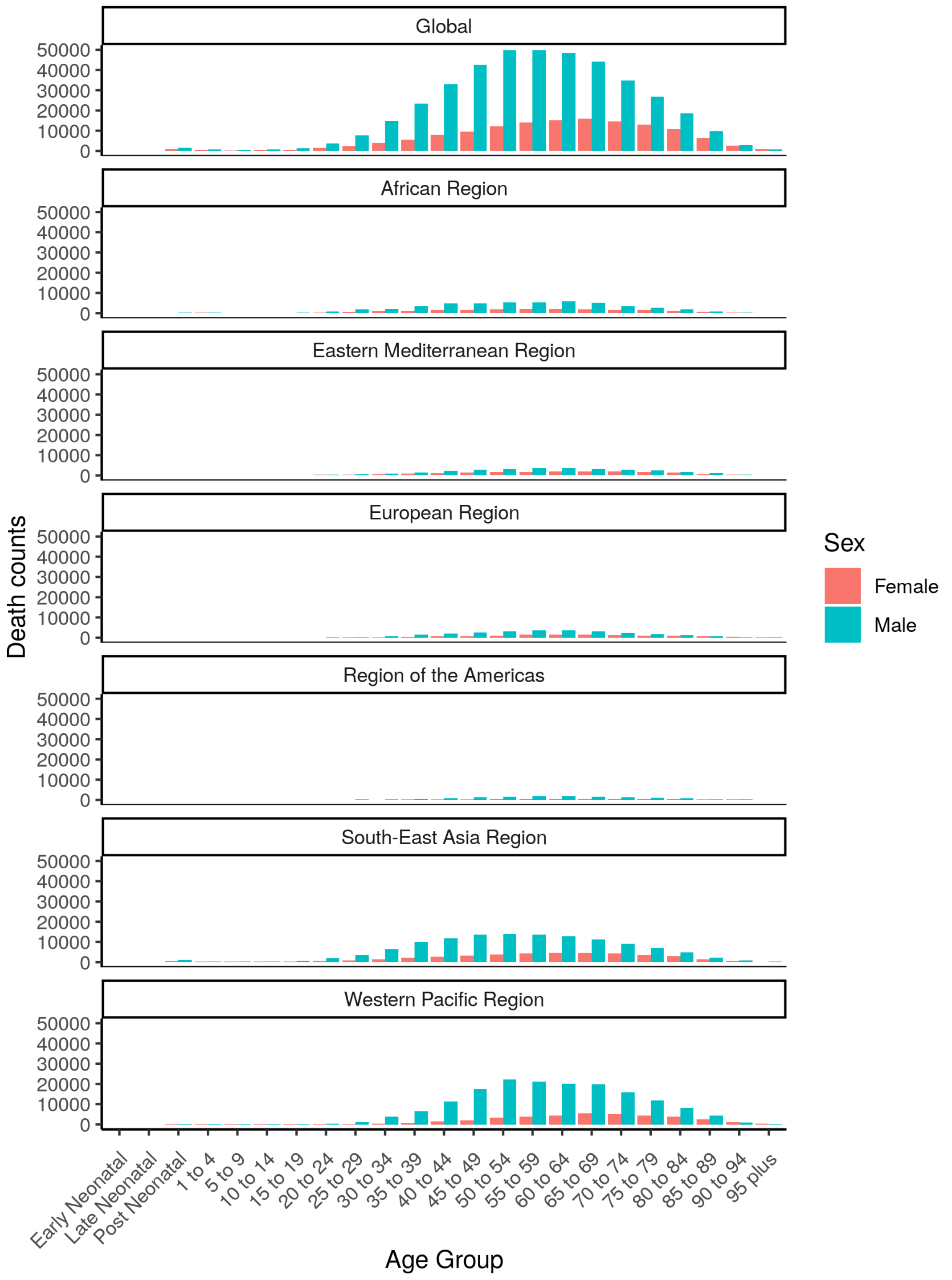

Supplemental Figure 7. Age-sex-specific death rates per 100,000 of HBV-related diseases in 2019, globally and by WHO region

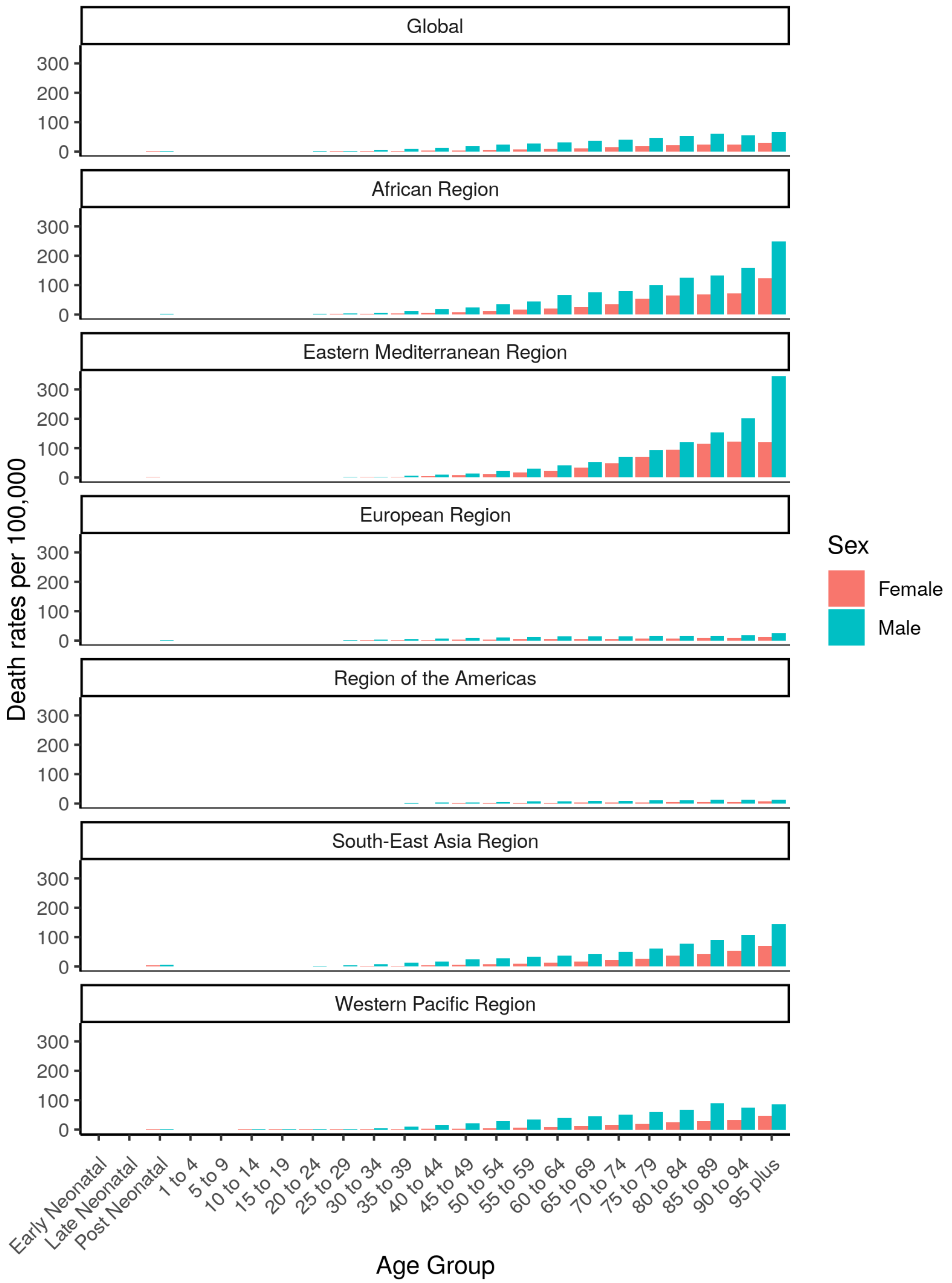

16 Authorship Contributions

16.1 Managing the estimation or publications process

M Ashworth Dirac and Theo Vos.

16.2 Writing the first draft of the manuscript

M Ashworth Dirac, Lindsey Hiebert, Brittney S Sheena, John W Ward, and Theo Vos.

16.3 Primary responsibility for applying analytical methods to produce estimates

Brittney S Sheena.

16.4 Primary responsibility for seeking, cataloguing, extracting, or cleaning data; designing or coding figures and tables

Brittney S Sheena, Hannah Han, and Helen Ippolito.

16.5 Providing data or critical feedback on data sources

Hedayat Abbastabar, Amir Abdoli, Hiwa Abubaker Ali, Qorinah Estiningtyas Sakilah Adnani, Muhammad Sohail Afzal, Saira Afzal, Bright Opoku Ahinkorah, Sajjad Ahmad, Tauseef Ahmad, Sepideh Ahmadi, Muktar Beshir Ahmed, Tarik Ahmed Rashid, Gizachew Taddesse Akalu, Addis Aklilu, Hanadi Al Hamad, Fares Alahdab, Dejene Tsegaye Alem, Robert Kaba Alhassan, Liaqat Ali, Motasem Alkhayyat, Sami Almustanyir, Saeed Amini, Hubert Amu, Jalal Arabloo, Ayele Mamo Argaw, Daniel Atlaw, Floriane Ausloos, Marcel Ausloos, Amirhossein Azari Jafari, Saeed Bahadory, Atif Amin Baig, Maciej Banach, Nastaran Barati, Amadou Barrow, Abdul-Monim Mohammad Batiha, Diana Fernanda Bejarano Ramirez, Devidas S Bhagat, Vijayalakshmi S Bhojaraja, Belay Boda Abule Bodicha, Archith Boloor, Dejana Braithwaite, Luis Alberto Cámera, Dinh-Toi Chu, Paolo Angelo Cortesi, Xiaochen Dai, Lalit Dandona, Rakhi Dandona, Fernando Pio De la Hoz, Feleke Mekonnen Demeke, Meseret Derbew Molla, Mandira Lamichhane Dhimal, Meghnath Dhimal, Mojtaba Didehdar, Linh Phuong Doan, Fariba Dorostkar, Thomas M Drake, Fatemeh Eghbalian, Michael Ekholuenetale, Maysaa El Sayed Zaki, Shymaa Enany, Ryenchindorj Erkhembayar, Sharareh Eskandarieh, Firooz Esmaeilzadeh, Hossein Farrokhpour, Getahun Fetensa, Mohamed M Gad, Shilpa Gaidhane, Natalie C Galles, Teferi Gebru Gebremeskel, Keyghobad Ghadiri, Kazem Ghaffari, Mansour Ghafourifard, Ahmad Ghashghaee, Amit Goel, Mahaveer Golechha, Pouya Goleij, Max G Griswold, Bhawna Gupta, Sapna Gupta, Veer Bala Gupta, Vivek Kumar Gupta, Rasool Haddadi, Rabih Halwani, Saeed S Hamid, Shafiul Haque, Harapan Harapan, Ahmed I Hasaballah, Soheil Hassanipour, Hadi Hassankhani, Claudiu Herteliu, Demisu Zenbaba Heyi, Kamal Hezam, Mehdi Hosseinzadeh, Mowafa Househ, Nawfal R Hussein, Segun Emmanuel Ibitoye, Helen Ippolito, Nahlah Elkudssiah Ismail, Kathryn H Jacobsen, Amirreza Javadi Mamaghani, Shubha Jayaram, Farahnaz Joukar, Billingsley Kaambwa, Zubair Kabir, Rohollah Kalhor, Himal Kandel, Tesfaye K Tesfaye Kanko, Rami S Kantar, Bekalu Getnet Kassa, Yousef Saleh Khader, Himanshu Khajuria, Imteyaz A Khan, Moien AB Khan, Amir M Khater, Mahalaqua Nazli Khatib, Abdullah T Khoja, Gyu Ri Kim, Hanna Kim, Min Seo Kim, Yun Jin Kim, Jonathan M Kocarnik, G Anil Kumar, Dharmesh Kumar Lal, Savita Lasrado, Sang-woong Lee, Muhammed Magdy Abd El Razek, Alaa Makki, Fariborz Mansour-Ghanaei, Mohammad Ali Mansournia, Francisco Rogerlândio Martins-Melo, Walter Mendoza, Ritesh G Menezes, Seyyedmohammadsadeq Mirmoeeni, Erkin M Mirrakhimov, Babak Moazen, Mokhtar Mohammadi, Shafiu Mohammed, Nagabhishek Moka, Ali H Mokdad, Jalal Moludi, Ghobad Moradi, Ebrahim Mostafavi, Sumaira Mubarik, Malaisamy Muniyandi, Christopher J L Murray,

Mohsen Naghavi, Mukhammad David Naimzada, Sreenivas Narasimha Swamy, Zuhair S Natto, Biswa Prakash Nayak, Javad Nazari, Ionut Negoi, Sandhya Neupane Kandel, Huong Lan Thi Nguyen, Robina Khan Niazi, Chukwudi A Nnaji, Jean Jacques Noubiap, Bogdan Oancea, Chimedsuren Ochir, Andrew T Olagunju, Emad Omer, Stanislav S Otstavnov, Bilcha Oumer, Jagadish Rao Padubidri, Adrian Pana, Anamika Pandey, Eun-Cheol Park, Fatemeh Pashazadeh Kan, Urvish K Patel, Zahra Zahid Piracha, Maarten J Postma, Hossein Poustchi, Mohammad Rabiee, Navid Rabiee, Alireza Rafiei, Sima Rafiei, Amir Masoud Rahmani, Chythra R Rao, Sowmya J Rao, Reza Rawassizadeh, Jefferson Antonio Buendia Rodriguez, Godfrey M Rwegerera, Siamak Sabour, Basema Saddik, Umar Saeed, Abdallah M Samy, Juan Sanabria, Milena M Santric-Milicevic, Arash Sarveazad, Brijesh Sathian, Monika Sawhney, Abdul-Aziz Seidu, Allen Seylani, Masood Ali Shaikh, Murad Ziyaudinovich Shakhmardanov, Mohammed Shannawaz, Brittney S Sheena, Suchitra M Shenoy, Jeevan K Shetty, Jae Il Shin, Parnian Shobeiri, Achintya Dinesh Singh, Jasvinder A Singh, Valentin Yurievich Skryabin, Anna Aleksandrovna Skryabina, Mircea Tampa, Ker-Kan Tan, Abdelghani Tbakhi, Seyed Abolfazl Tohidast, Marcos Roberto Tovani-Palone, Mai Thi Ngoc Tran, Irfan Ullah, Sahel Valadan Tahbaz, Pascual R Valdez, Shoban Babu Varthya, Bay Vo, Yasir Waheed, Adisu Birhanu Weldesenbet, Melat Woldemariam, Seyed Hossein Yahyazadeh Jabbari, Mehdi Yaseri, Birhanu Wubale Yirdaw, Naohiro Yonemoto, Chuanhua Yu, Ismaeel Yunusa, Leila Zaki, and Mohammad Zamani.

16.6 Developing methods or computational machinery

Hiwa Abubaker Ali, Saira Afzal, Muktar Beshir Ahmed, Tarik Ahmed Rashid, Tayyaba Akram, Atif Amin Baig, Nastaran Barati, Garland T Culbreth, Xiaochen Dai, Fatemeh Eghbalian, Firooz Esmaeilzadeh, Shilpa Gaidhane, Natalie C Galles, Kazem Ghaffari, Max G Griswold, Rasool Haddadi, Hannah Han, Mehdi Hosseinzadeh, Mowafa Househ, Amirreza Javadi Mamaghani, Mahalaqua Nazli Khatib, Jonathan M Kocarnik, Sang-woong Lee, Stephen S Lim, Hassan Magdy Abd El Razek, Muhammed Magdy Abd El Razek, Alaa Makki, Mokhtar Mohammadi, Nagabhishek Moka, Ali H Mokdad, Christopher J L Murray, Mohsen Naghavi, Robina Khan Niazi, Emad Omer, Bilcha Oumer, Zahra Zahid Piracha, Mohammad Rabiee, Navid Rabiee, Amir Masoud Rahmani, Reza Rawassizadeh, Umar Saeed, Abdallah M Samy, Brittney S Sheena, Mai Thi Ngoc Tran, Bay Vo, Theo Vos, and Leila Zaki.

16.7 Providing critical feedback on methods or results

Hedayat Abbastabar, Hiwa Abubaker Ali, Mesafint Molla Adane, Oyelola A Adegboye, Qorinah Estiningtyas Sakilah Adnani, Shailesh M Advani, Muhammad Sohail Afzal, Saira Afzal, Mohamad Aghaie Meybodi, Bahman Ahadinezhad, Bright Opoku Ahinkorah, Sajjad Ahmad, Tauseef Ahmad, Haroon Ahmed, Muktar Beshir Ahmed, Tarik Ahmed Rashid, Gizachew Taddesse Akalu, Addis Aklilu, Tayyaba Akram, Hanadi Al Hamad, Fares Alahdab, Adugnaw Zeleke Alem, Dejene Tsegaye Alem, Robert Kaba Alhassan, Liaqat Ali, Yousef Alimohamadi, Vahid Alipour, Motasem Alkhayyat, Sami Almustanyir, Rajaa M Al-Raddadi, Saeed Amini, Hubert Amu, Robert Ancuceanu, Catalina Liliana Andrei, Tudorel Andrei, Adnan Ansar, Anayochukwu Edward Anyasodor, Jalal Arabloo, Morteza Arab-Zozani, Zeleke Gebru Argaw, Anton A Artamonov, Tahira Ashraf, Daniel Atlaw, Floriane Ausloos, Marcel Ausloos, Sina Azadnajafabad, Mohammadreza Azangou-Khyavy, Amirhossein Azari Jafari, Ghasem Azarian, Sayna Bagheri, Atif Amin Baig, Maciej Banach, Nastaran Barati, Amadou Barrow, Abdul-Monim Mohammad Batiha, Diana Fernanda Bejarano Ramirez, Alemshet Yirga Berhie, Devidas S Bhagat, Nikha Bhardwaj, Pankaj Bhardwaj, Krittika Bhattacharyya, Vijayalakshmi S Bhojaraja, Ali Bijani, Antonio Biondi, Belay Boda Abule Bodicha, Hunduma Amensisa Bojia, Archith Boloor, Cristina Bosetti, Dejana Braithwaite, Zahid A Butt, Luis Alberto Cámera, Raja Chandra Chakinala, Promit

Ananyo Chakraborty, Jaykaran Charan, Shu Chen, Jee-Young Jasmine Choi, Sonali Gajanan Choudhari, Fazle Rabbi Chowdhury, Dinh-Toi Chu, Sheng-Chia Chung, Paolo Angelo Cortesi, Benjamin C Cowie, Omid Dadras, Xiaochen Dai, Lalit Dandona, Rakhi Dandona, Fernando Pio De la Hoz, Sisay Abebe Debela, Mohammed Gebre Dedefo, Takele Gezahegn G Demie, Getu Debalkie Demissie, Meseret Derbew Molla, Abebaw Alemayehu Desta, Deepak Dhamnetiya, Mandira Lamichhane Dhimal, Meghnath Dhimal, Mojtaba Didehdar, Linh Phuong Doan, Fariba Dorostkar, Thomas M Drake, Fatemeh Eghbalian, Michael Ekholuenetale, Iman El Sayed, Maysaa El Sayed Zaki, Muhammed Elhadi, Mohamed A Elmonem, Aisha Elsharkawy, Daniel Berhanie Enyew, Ryenchindorj Erkhembayar, Sharareh Eskandarieh, Firooz Esmaeilzadeh, Hossein Farrokhpour, Getahun Fetensa, Florian Fischer, Masoud Foroutan, Mohamed M Gad, Abhay Motiramji Gaidhane, Shilpa Gaidhane, Natalie C Galles, Teferi Gebru Gebremeskel, Eyob Alemayehu Alemayehu Gebreyohannes, Kazem Ghaffari, Mansour Ghafourifard, Ahmad Ghashghaee, Ali Gholami, Abdolmajid Gholizadeh, Amit Goel, Mahaveer Golechha, Davide Golinelli, Yitayal Ayalew Goshu, Bhawna Gupta, Veer Bala Gupta, Vivek Kumar Gupta, Rasool Haddadi, Rabih Halwani, Samer Hamidi, Hannah Han, Asif Hanif, Shafiul Haque, Harapan Harapan, Arief Hargono, Sanam Hariri, Ahmed I Hasaballah, S M Mahmudul Hasan, Soheil Hassanipour, Hadi Hassankhani, Simon I Hay, Khezar Hayat, Claudiu Herteliu, Demisu Zenbaba Heyi, Lindsey Hiebert, Ramesh Holla, Mohammad-Salar Hosseini, Mehdi Hosseinzadeh, Mihaela Hostiuc, Mowafa Househ, Nawfal R Hussein, Segun Emmanuel Ibitoye, Olayinka Stephen Ilesanmi, Irena M Ilic, Milena D Ilic, Lalu Muhammad Irham, Jessica Y Islam, Nahlah Elkudssiah Ismail, Kathryn H Jacobsen, Farhad Jadidi-Niaragh, Amirreza Javadi Mamaghani, Shubha Jayaram, Ranil Jayawardena, Rime Jebai, Ravi Prakash Jha, Nitin Joseph, Farahnaz Joukar, Billingsley Kaambwa, Ali Kabir, Zubair Kabir, Rohollah Kalhor, Himal Kandel, Tesfaye K Tesfaye Kanko, Rami S Kantar, Ibraheem M Karaye, Phillip M Kemp Bohan, Mohammad Keykhaei, Yousef Saleh Khader, Himanshu Khajuria, Imteyaz A Khan, Junaid Khan, Moien AB Khan, Javad Khanali, Amir M Khater, Mahalaqua Nazli Khatib, Mahmoud Khodadost, Abdullah T Khoja, Omid Khosravizadeh, Jagdish Khubchandani, Gyu Ri Kim, Hanna Kim, Min Seo Kim, Yun Jin Kim, Jonathan M Kocarnik, Ali-Asghar Kolahi, Rajasekaran Koteeswaran, G Anil Kumar, Carlo La Vecchia, Dharmesh Kumar Lal, Savita Lasrado, Jeffrey V Lazarus, Caterina Ledda, Doo Woong Lee, Sang-woong Lee, Yeong Yeh Lee, Jiarui Li, Stephen S Lim, Stany W Lobo, Joana A Loureiro, Jennifer H MacLachlan, Hassan Magdy Abd El Razek, Muhammed Magdy Abd El Razek, Azeem Majeed, Alaa Makki, Mohammad-Reza Malekpour, Reza Malekzadeh, Ahmad Azam Malik, Fariborz Mansour-Ghanaei, Mohammad Ali Mansournia, Francisco Rogerlândio Martins-Melo, Philippa C Matthews, Walter Mendoza, Ritesh G Menezes, Amanual Getnet Mersha, Tomislav Mestrovic, Ted R Miller, Le Huu Nhat Minh, Andreea Mirica, Seyyedmohammadsadeq Mirmoeeni, Erkin M Mirrakhimov, Sanjeev Misra, Prasanna Mithra, Babak Moazen, Ashraf Mohamadkhani, Mokhtar Mohammadi, Shafiu Mohammed, Nagabhishek Moka, Ali H Mokdad, Jalal Moludi, Sara Momtazmanesh, Ghobad Moradi, Maliheh Moradzadeh, Rahmatollah Moradzadeh, Ebrahim Mostafavi, Sumaira Mubarik, Malaisamy Muniyandi, Christopher J L Murray, Mohsen Naghavi, Mukhammad David Naimzada, Sreenivas Narasimha Swamy, Zuhair S Natto, Biswa Prakash Nayak, Javad Nazari, Ionut Negoi, Serban Mircea Negru, Seyed Aria Nejadghaderi, Sandhya Neupane Kandel, Huong Lan Thi Nguyen, Che Henry Ngwa, Robina Khan Niazi, Chukwudi A Nnaji, Jean Jacques Noubiap, Ali Nowroozi, Bogdan Oancea, Chimedsuren Ochir, Andrew T Olagunju, Ahmed Omar Bali, Emad Omer, Stanislav S Otstavnov, Bilcha Oumer, Jagadish Rao Padubidri, Adrian Pana, Anamika Pandey, Eun-Cheol Park, Fatemeh Pashazadeh Kan, Urvish K Patel, Uttam Paudel, Zahra Zahid Piracha, Richard Charles G Pollok, Maarten J Postma, Alireza Rafiei, Sima Rafiei, Pavan Manibettu Raghuram, Mosiur Rahman, Amir Masoud Rahmani, Setyaningrum Rahmawaty, Aashish Rajesh, Priyanga Ranasinghe, Chythra R Rao, Sowmya J Rao, Mahsa Rashidi, Mohammad-Mahdi Rashidi, David Laith Rawaf, Salman Rawaf, Reza Rawassizadeh, Negar Rezaei, Aziz Rezapour, Sahba Rezazadeh-Khadem, Jefferson Antonio Buendia Rodriguez, Godfrey M

Rwegerera, Siamak Sabour, Basema Saddik, Mohammad Reza Saeb, Umar Saeed, KM Saif-Ur-Rahman, Hamideh Salimzadeh, Chethan Sampath, Abdallah M Samy, Juan Sanabria, Milena M Santric-Milicevic, Arash Sarveazad, Brijesh Sathian, Monika Sawhney, Sadaf G Sepanlou, Allen Seylani, Saeed Shahabi, Masood Ali Shaikh, Murad Ziyaudinovich Shakhmardanov, Mohammed Shannawaz, Brittney S Sheena, Suchitra M Shenoy, Jeevan K Shetty, Pavanchand H Shetty, Kenji Shibuya, Jae Il Shin, Parnian Shobeiri, Migbar Mekonnen Sibhat, Achintya Dinesh Singh, Jasvinder A Singh, Surjit Singh, Valentin Yurievich Skryabin, Anna Aleksandrovna Skryabina, Suhang Song, Eyayou Girma Tadesse, Mircea Tampa, Ker-Kan Tan, Ahmad Tavakoli, Abdelghani Tbakhi, Belay Negash Tefera, Habtamu Molla Tesfaw, Rekha Thapar, Aravind Thavamani, Seyed Abolfazl Tohidast, Daniel Nigusse Tollosa, Marcos Roberto Tovani-Palone, Eugenio Traini, Mai Thi Ngoc Tran, Indang Trihandini, Biruk Shalmeno Tusa, Irfan Ullah, Marco Vacante, Sahel Valadan Tahbaz, Pascual R Valdez, Shoban Babu Varthya, Bay Vo, Theo Vos, Yasir Waheed, Adisu Birhanu Weldesenbet, Melat Woldemariam, Suowen Xu, Seyed Hossein Yahyazadeh Jabbari, Yigizie Yeshaw, Vahit Yiğit, Birhanu Wubale Yirdaw, Naohiro Yonemoto, Chuanhua Yu, Ismaeel Yunusa, Mazyar Zahir, Leila Zaki, Mohammad Zamani, Maryam Zamanian, and Mikhail Sergeevich Zastrozhin.

16.8 Drafting the work or revising is critically for important intellectual content

Mohsen Abbasi-Kangevari, Zeinab Abbasi-Kangevari, Oyelola A Adegboye, Qorinah Estiningtyas Sakilah Adnani, Shailesh M Advani, Muhammad Sohail Afzal, Saira Afzal, Bright Opoku Ahinkorah, Sajjad Ahmad, Sepideh Ahmadi, Muktar Beshir Ahmed, Gizachew Taddesse Akalu, Tayyaba Akram, Fares Alahdab, Adugnaw Zeleke Alem, Fadwa Alhalaiqa Naji Alhalaiqa, Liaqat Ali, Muhammad Ashar Ali, Motasem Alkhayyat, Sami Almustanyir, Haya Altawalah, Saeed Amini, Hubert Amu, Robert Ancuceanu, Amir Anoushiravani, Anayochukwu Edward Anyasodor, Jalal Arabloo, Morteza Arab-Zozani, Zeleke Gebru Argaw, Muhammad Arshad, Tahira Ashraf, Daniel Atlaw, Floriane Ausloos, Marcel Ausloos, Sina Azadnajafabad, Mohammadreza Azangou-Khyavy, Amirhossein Azari Jafari, Sayna Bagheri, Atif Amin Baig, Maciej Banach, Nastaran Barati, Amadou Barrow, Diana Fernanda Bejarano Ramirez, Uzma Iqbal Belgaumi, Krittika Bhattacharyya, Vijayalakshmi S Bhojaraja, Antonio Biondi, Belay Boda Abule Bodicha, Dejana Braithwaite, Nikolay Ivanovich Briko, Raja Chandra Chakinala, Fazle Rabbi Chowdhury, Dinh-Toi Chu, Paolo Angelo Cortesi, Benjamin C Cowie, Takele Gezahegn G Demie, Getu Debalkie Demissie, Meseret Derbew Molla, Deepak Dhamnetiya, Mandira Lamichhane Dhimal, Meghnath Dhimal, Mojtaba Didehdar, Linh Phuong Doan, Thomas M Drake, Fatemeh Eghbalian, Iman El Sayed, Maysaa El Sayed Zaki, Muhammed Elhadi, Mohamed A Elmonem, Aisha Elsharkawy, Sharareh Eskandarieh, Firooz Esmaeilzadeh, Sayeh Ezzikouri, Florian Fischer, Masoud Foroutan, Mohamed M Gad, Shilpa Gaidhane, Silvano Gallus, Teferi Gebru Gebremeskel, Kazem Ghaffari, Mansour Ghafourifard, Seyyed-Hadi Ghamari, Ali Gholami, Abdolmajid Gholizadeh, Aima Gilani, Davide Golinelli, Giuseppe Gorini, Max G Griswold, Mohammed Ibrahim Mohialdeen Gubari, Bhawna Gupta, Sapna Gupta, Veer Bala Gupta, Vivek Kumar Gupta, Saeed S Hamid, Shafiul Haque, Harapan Harapan, Sanam Hariri, Ahmed I Hasaballah, S M Mahmudul Hasan, Simon I Hay, Khezar Hayat, Claudiu Herteliu, Demisu Zenbaba Heyi, Kamal Hezam, Lindsey Hiebert, Ramesh Holla, Mohammad-Salar Hosseini, Mostafa Hosseini, Mowafa Househ, Junjie Huang, Nawfal R Hussein, Ivo Iavicoli, Segun Emmanuel Ibitoye, Olayinka Stephen Ilesanmi, Irena M Ilic, Milena D Ilic, Helen Ippolito, Lalu Muhammad Irham, Jessica Y Islam, Nahlah Elkudssiah Ismail, Kathryn H Jacobsen, Amirreza Javadi Mamaghani, Shubha Jayaram, Rime Jebai, Ravi Prakash Jha, Nitin Joseph, Ali Kabir, Himal Kandel, Rami S Kantar, Yousef Saleh Khader, Himanshu Khajuria, Gulfaraz Khan, Imteyaz A Khan, Junaid Khan, Moien AB Khan, Javad Khanali, Amir M Khater, Mahalaqua Nazli Khatib, Jagdish Khubchandani, Yun Jin Kim, Jonathan M Kocarnik, Carlo La Vecchia, Iván Landires, Savita Lasrado,

Jeffrey V Lazarus, Yeong Yeh Lee, Miriam Levi, Platon D Lopukhov, Joana A Loureiro, Jennifer H MacLachlan, Hassan Magdy Abd El Razek, Muhammed Magdy Abd El Razek, Mohammad-Reza Malekpour, Reza Malekzadeh, Ahmad Azam Malik, Francisco Rogerlândio Martins-Melo, Philippa C Matthews, Walter Mendoza, Ritesh G Menezes, Tuomo J Meretoja, Tomislav Mestrovic, Ted R Miller, Le Huu Nhat Minh, Seyyedmohammadsadeq Mirmoeeni, Prasanna Mithra, Babak Moazen, Shafiu Mohammed, Nagabhishek Moka, Ali H Mokdad, Jalal Moludi, Sara Momtazmanesh, Lorenzo Monasta, Paula Moraga, Ebrahim Mostafavi, Malaisamy Muniyandi, Mohsen Naghavi, Mukhammad David Naimzada, Sreenivas Narasimha Swamy, Zuhair S Natto, Biswa Prakash Nayak, Javad Nazari, Ionut Negoi, Serban Mircea Negru, Seyed Aria Nejadghaderi, Sandhya Neupane Kandel, Huong Lan Thi Nguyen, Che Henry Ngwa, Robina Khan Niazi, Ali Nowroozi, Virginia Nuñez-Samudio, Bogdan Oancea, Oluwakemi Ololade Odukoya, In-Hwan Oh, Andrew T Olagunju, Babayemi Oluwaseun Olakunde, Stanislav S Otstavnov, Bilcha Oumer, Jagadish Rao Padubidri, Adrian Pana, Urvish K Patel, Ionela-Roxana Petcu, Zahra Zahid Piracha, Richard Charles G Pollok, Maarten J Postma, Akram Pourshams, Mohammad Rabiee, Navid Rabiee, Priyanga Ranasinghe, Chythra R Rao, Sowmya J Rao, Mohammad-Mahdi Rashidi, David Laith Rawaf, Salman Rawaf, Sahba Rezazadeh-Khadem, Jefferson Antonio Buendia Rodriguez, Godfrey M Rwegerera, Siamak Sabour, Basema Saddik, Umar Saeed, Amirhossein Sahebkar, KM Saif-Ur-Rahman, Sarvenaz Salahi, Hamideh Salimzadeh, Abdallah M Samy, Juan Sanabria, Francesco Sanmarchi, Milena M Santric-Milicevic, Sadaf G Sepanlou, Allen Seylani, Saeed Shahabi, Elaheh Shaker, Murad Ziyaudinovich Shakhmardanov, Mohammed Shannawaz, Brittney S Sheena, Suchitra M Shenoy, Jeevan K Shetty, Pavanchand H Shetty, Kenji Shibuya, Parnian Shobeiri, Achintya Dinesh Singh, Jasvinder A Singh, Surjit Singh, Valentin Yurievich Skryabin, Anna Aleksandrovna Skryabina, Seidamir Pasha Tabaeian, Eyayou Girma Tadesse, Majid Taheri, Mircea Tampa, Ker-Kan Tan, Belay Negash Tefera, Arash Tehrani-Banihashemi, Seyed Abolfazl Tohidast, Maria Elena Tosti, Marcos Roberto Tovani-Palone, Mai Thi Ngoc Tran, Biruk Shalmeno Tusa, Irfan Ullah, Marco Vacante, Sahel Valadan Tahbaz, Shoban Babu Varthya, Theo Vos, Yasir Waheed, Adisu Birhanu Weldesenbet, Seyed Hossein Yahyazadeh Jabbari, Yigizie Yeshaw, Vahit Yiğit, Naohiro Yonemoto, Mazyar Zahir, Leila Zaki, Maryam Zamanian, and Mikhail Sergeevich Zastrozhin.

16.9 Managing the overall research enterprise

Lalit Dandona, Simon I Hay, Ali H Mokdad, Christopher J L Murray, and Mohsen Naghavi.

17 Supplementary References

1 Stevens G, Alkema L, Black R, *et al.* Guidelines for accurate and transparent health estimates reporting: the GATHER statement. *Lancet* 2016; **388**: 19–23.

2 Stanaway J, Flaxman A, Naghavi M, Mitzmaurice C, Vos T, Abubakar I. The global burden of viral hepatitis from 1990 to 2013: findings from the Global Burden of Disease Study 2013. *The Lancet* 2016; **388**: 1081–8.

3 Schweitzer A, Horn J, Mikolajczyk RT, Krause G, Ott JJ. Estimations of worldwide prevalence of chronic hepatitis B virus infection: a systematic review of data published between 1965 and 2013. *The Lancet* 2015; **386**: 1546–55.

4 GBD 2013 Mortality and Causes of Death Collaborators. Global, regional, and national age-sex specific all-cause and cause-specific mortality for 240 causes of death, 1990-2013: a systematic analysis for the Global Burden of Disease Study 2013. *Lancet* 2015; **385**: 117–71.

5 GBD 2015 Disease and Injury Incidence and Prevalence Collaborators. Global, regional, and national incidence, prevalence, and years lived with disability for 310 diseases and injuries, 1990-2015: a systematic analysis for the Global Burden of Disease Study 2015. *Lancet Lond Engl* 2016; **388**: 1545–602.

6 GBD 2016 Disease and Injury Incidence and Prevalence Collaborators. Global, regional, and national incidence, prevalence, and years lived with disability for 328 diseases and injuries for 195 countries, 1990-2016: a systematic analysis for the Global Burden of Disease Study 2016. *Lancet Lond Engl* 2017; **390**: 1211–59.

7 GBD 2017 Disease and Injury Incidence and Prevalence Collaborators. Global, regional, and national incidence, prevalence, and years lived with disability for 354 diseases and injuries for 195 countries and territories, 1990-2017: a systematic analysis for the Global Burden of Disease Study 2017. *Lancet Lond Engl* 2018; **392**: 1789–858.

8 Flaxman A, Vos T, Murray CJ. An integrative metaregression framework for descriptive epidemiology. Seattle: University of Washington Press, 2015.

9 GBD 2019 Diseases and Injuries Collaborators. Global burden of 369 diseases and injuries in 204 countries and territories, 1990–2019: a systematic analysis for the global burden of disease study 2019. *The Lancet* 2020; **396**: 1204–22.

10 Zheng P, Aravkin A, Barber R, Sorensen R, Murray C. Trimmed Constrained Mixed Effects Models: Formulations and Algorithms. *bioRxiv* 2020; : 2020.01.28.923599.

11 GBD 2019 Risk Factors Collaborators. Global burden of 87 risk factors in 204 countries and territories, 1990–2019: a systematic analysis for the Global Burden of Disease Study 2019. *The Lancet* 2020; **396**: 1223–49.

12 GBD 2019 Demographics Collaborators. Global age-sex-specific fertility, mortality, healthy life expectancy (HALE), and population estimates in 204 countries and territories, 1950–2019: a comprehensive demographic analysis for the Global Burden of Disease Study 2019. *Lancet Lond Engl* 2020; **396**: 1160–203.

13 GBD 2015 Healthcare Access and Quality Collaborators. Healthcare Access and Quality Index based on mortality from causes amenable to personal health care in 195 countries and territories, 1990-2015: a novel analysis from the Global Burden of Disease Study 2015. *Lancet Lond Engl* 2017; **390**: 231–66.

14 Galles NC, Liu PY, Updike RL, *et al.* Measuring routine childhood vaccination coverage in 204 countries and territories, 1980–2019: a systematic analysis for the Global Burden of Disease Study 2020, Release 1. *The Lancet* 2021; **398**: 503–21.

15 Viviani S, Jack A, Hall AJ, *et al.* Hepatitis B vaccination in infancy in The Gambia: protection against carriage at 9 years of age. *Vaccine* 1999; **17**: 2946–50.

16 Mendy M, Peterson I, Hossin S, *et al.* Observational Study of Vaccine Efficacy 24 Years after the Start of Hepatitis B Vaccination in Two Gambian Villages: No Need for a Booster Dose. *PLOS ONE* 2013; **8**: e58029.

17 Shen L, Wang F, Feng W, *et al.* Efficacy of yeast-derived recombinant hepatitis B vaccine after being used for 12 years in highly endemic areas in China. *Vaccine* 2012; **30**: 6623–7.

18 Pinkbook: Hepatitis B | CDC. 2021; published online Aug 17. https://www.cdc.gov/vaccines/pubs/pinkbook/hepb.html (accessed Oct 5, 2021).

19 Haagsma JA, Maertens de Noordhout C, Polinder S, *et al.* Assessing disability weights based on the responses of 30,660 people from four European countries. *Popul Health Metr* 2015; **13**: 10.

20 Salomon JA, Haagsma JA, Davis A, *et al.* Disability weights for the Global Burden of Disease 2013 study. *Lancet Glob Health* 2015; **3**: e712-723.

21 Vos T, Lim SS, Abbafati C, *et al.* Global burden of 369 diseases and injuries in 204 countries and territories, 1990–2019: a systematic analysis for the Global Burden of Disease Study 2019. *The Lancet* 2020; **396**: 1204–22.

22 GBD 2017 Disease and Injury Incidence and Prevalence Collaborators. Global, regional, and national incidence, prevalence, and years lived with disability for 354 diseases and injuries for 195 countries and territories, 1990-2017: a systematic analysis for the Global Burden of Disease Study 2017. *Lancet Lond Engl* 2018; **392**: 1789–858.

23 Haagsma JA, Maertens de Noordhout C, Polinder S, *et al.* Assessing disability weights based on the responses of 30,660 people from four European countries. *Popul Health Metr* 2015; **13**: 10.

24 Salomon JA, Vos T, Hogan DR, *et al.* Common values in assessing health outcomes from disease and injury: disability weights measurement study for the Global Burden of Disease Study 2010. *Lancet Lond Engl* 2012; **380**: 2129–43.

25 Naghavi M, Makela S, Foreman K, O'Brien J, Pourmalek F, Lozano R. Algorithms for enhancing public health utility of national causes-of-death data. *Popul Health Metr* 2010; **8**: 9.

26 Foreman KJ, Lozano R, Lopez AD, Murray CJ. Modeling causes of death: an integrated approach using CODEm. *Popul Health Metr* 2012; **10**: 1.

27 Friedman J, York H, Graetz N, *et al.* Measuring and forecasting progress towards the education-related SDG targets. *Nature* 2020; **580**: 636–9.

28 Global Burden of Disease Cancer Collaboration, Fitzmaurice C, Abate D, *et al.* Global, Regional, and National Cancer Incidence, Mortality, Years of Life Lost, Years Lived With Disability, and Disability-Adjusted Life-Years for 29 Cancer Groups, 1990 to 2017: A Systematic Analysis for the Global Burden of Disease Study. *JAMA Oncol* 2019; **5**: 1749–68.

29 Kocarnik J, Compton K, Dean F. Cancer Incidence, Mortality, Years of Life Lost, Years Lived with Disability, and Disability-Adjusted Life Years for 29 Cancer Groups from 2010 to 2019: A Systemic Analysis of Cancer Burden Globally, Nationally, and by Socio-demographic Index for the Global Burden Disease Study 2019. .

30 Surveillance, Epidemiology, and End Results (SEER) Program (www.Seer.Cancer.Gov) SEER*Stat Database: Incidence - SEER 18. .

31 R D, P P, JAH W. Cancer Incidence in Five Continents Volume I. https://publications.iarc.fr/Non-Series-Publications/Other-Non-Series-Publications/Cancer-Incidence-In-Five-Continents-Volume-I-1966 (accessed Feb 24, 2021).

32 Doll R, Muir C, Waterhouse J. Cancer Incidence in Five Continents: Volume II–1970. Springer Science & Business Media, 2012.

33 Waterhouse J, Muir C, Correa P, Powell J. Cancer Incidence in Five Continents III. Lyon: IARC; 1976. .

34 Waterhouse J, Muir C, Shanmugaratnam K, Powell J. Cancer Incidence in Five Continents IV. Lyon: IARC; 1982. .

35 Muir C, Mack T, Powell J, Whelan S. Cancer Incidence in Five Continents V. Lyon: IARC; 1987. .

36 Parkin D, Muir C, Whelan S, Gao Y, Ferlay J, Powell J. Cancer Incidence in Five Continents VI. Lyon: IARC; 1992. .

37 Parkin D, Whelan S, Ferlay J, Raymond L, Young J. Cancer Incidence in Five Continents VII. Lyon: IARC; 1997. .

38 Parkin D, Whelan S, Ferlay J, Teppo L, Thomas D. Cancer Incidence in Five Continents VIII. Lyon: IARC; 2002. .

39 Curado M, Edwards B, Shin H, et al. Cancer Incidence in Five Continents IX. Lyon: IARC; 2007. http://www.iarc.fr.

40 Forman D, Bray F, Brewster D, et al. Cancer Incidence in Five Continents X. http://ci5.iarc.fr. Published 2013. .

41 Bray F, Colombet M, Mery L, *et al.*, editors. Cancer Incidence in Five Continents. Lyon, France: International Agency for Research on Cancer, 2017.

42 Steliarova-Foucher E, O'Callaghan M, Ferlay J, *et al.* The European Cancer Observatory: A new data resource. *Eur J Cancer Oxf Engl 1990* 2015; **51**: 1131–43.

43 Engholm G, Ferlay J, Christensen N, *et al.* NORDCAN--a Nordic tool for cancer information, planning, quality control and research. *Acta Oncol Stockh Swed* 2010; **49**: 725–36.

44 Asadzadeh Vostakolaei F, Karim-Kos HE, Janssen-Heijnen MLG, Visser O, Verbeek ALM, Kiemeney LALM. The validity of the mortality to incidence ratio as a proxy for site-specific cancer survival. *Eur J Public Health* 2011; **21**: 573–7.

45 SEER*Stat Software. 2014 http://seer.cancer.gov/seerstat/.

46 SEER Cancer Statistics Review 1975-2011. http://seer.cancer.gov/csr/1975_2011/results_merged/topic_survival_by_year_dx.pdf.

47 de Martel C, Maucort-Boulch D, Plummer M, Franceschi S. World-wide relative contribution of hepatitis B and C viruses in hepatocellular carcinoma. *Hepatol Baltim Md* 2015; **62**: 1190–200.

48 Hong TP, Gow P, Fink M, *et al.* Novel population-based study finding higher than reported hepatocellular carcinoma incidence suggests an updated approach is needed. *Hepatol Baltim Md* 2016; **63**: 1205–12.

49 Edmunds WJ, Medley GF, Nokes DJ, Hall AJ, Whittle HC. The influence of age on the development of the hepatitis B carrier state. *Proc R Soc Lond B* 1993; **253**: 197–201.

50 McMahon BJ, Alward WLM, Hall DB, *et al.* Acute Hepatitis B Virus Infection: Relation of Age to the Clinical Expression of Disease and Subsequent Development of the Carrier State. *J Infect Dis* 1985; **151**: 599–603.

51 WHO. Draft global health sector strategies: Viral hepatitis, 2016-2021: Sixty-ninth world health assembly. Geneva, 2016 https://apps.who.int/gb/ebwha/pdf_files/WHA69/A69_32-en.pdf?ua=1 (accessed Jan 12, 2021).

52 World Health Organization. Interim Guidance for Country Validation of Viral Hepatitis Elimination. 2021 https://www.who.int/publications/i/item/9789240028395 (accessed Nov 8, 2021).
